# 2nd International Conference on Public Health and Health Sciences (PHHS2025)

**DOI:** 10.1097/MD.0000000000042787

**Published:** 2025-07-18

## PHHS25001: THE IMMEDIATE AND LONG-TERM SIDE EFFECTS OF MAJOR PUBLIC HEALTH EVENTS: MICRO-MODIFICATIONS IN HUMAN STRUCTURE AND EATING HABITS, AND THE MICRO-FLUCTUATIONS ON SOCIO-ECONOMICAL LEVELS

Jiameng Yuan^a^, Xiu Yu^b^, Yuhan Ba^c^, Hui Wang^d^, Xin Li^e^

^*a*^ *Duke Global Health Institute, Duke University, NC27701, Durham, North Carolina, USA,* ^*b*^ *International Business College, Leicester University, UKEC, LE2 1TH Leicester, UK,* ^*c*^ *School of Architecture & Fine Arts, Dalian University of Technology, 116024, Dalian, China,* ^*d*^ *First Center Hospital of Tianjin, 300380, Tianjin, China,* ^*e*^ *Physical Education Department, Luxun Academy of Fine Arts, 116650, Shenyang, China.*

**Background:** Major public events, such as pandemics, natural disasters, and economic crises, could profoundly impact human health, behaviors, and socio-economic systems, often with unknown long-term and subtle effects on human physiology, lifestyles, and economic stability. These events often trigger a cascade of changes that can have unknown long-term and subtle effects on human physiology, lifestyles, and economic stability. This paper explores the side effects of a recent major public event, the major public health events, from a biomedical and public health perspective.

**Subjects and Methods:** A comprehensive review of recent studies was conducted, examining the pandemic’s impacts on neurogenesis, brain microstructure, eating habits, and socio-economic fluctuations. Databases including PubMed, Scopus, and Google Scholar were searched using keywords such as “pandemic”, “neurogenesis”, “eating habits”, “food security”, “SES”, and “economic impacts”. A total of 150 studies were initially identified, and after a rigorous screening process based on inclusion and exclusion criteria, 50 studies were selected for critical analysis. The studies were reviewed to draw insights into the immediate and long-term pandemic effects on neurogenesis, brain microstructure, eating habits, and socio-economic fluctuations. The analysis involved a detailed examination of the methodologies, results, and conclusions of each study, with a focus on identifying common themes and discrepancies.

**Results:** The comprehensive review revealed several significant findings. Firstly, it has been found that major public health events induces changes in the microstructure of the human brain, reducing neurogenesis and altering neurological function, which in turn affects people’s physical health. At the same time, psychological stress and pandemic-related policies influence dietary behavior, facilitating shifts in emotional and nutrient-poor diets, as well as shifts in food purchasing patterns. In addition, at the socio-economic level, the pandemic triggered micro-fluctuations characterized by increased financial disparities, supply chain disruptions, and increased reliance on online and local food markets. Changes in educational patterns then further exacerbated health and economic inequalities, a feature that was particularly evident among marginalized populations.

**Conclusions:** The pandemic has induced significant modifications and micro-modifications in human physiology and socio-economic frameworks, with lasting implications for physical and mental health. Understanding these changes is critical for developing more resilient health systems, stable food policies, and equitable economic recovery strategies in future public health crises. Future research should focus on longitudinal studies to better understand the long-term effects of such events and to develop targeted interventions that can mitigate their negative impacts.

## PHHS25002: A STUDY ON THE CORRELATION BETWEEN DIFFERENT ORGANIZATIONAL FORMS OF TAI CHI AND MOBILE PHONE ADDICTION, MINDFULNESS, AND EMOTIONAL REGULATION IN COLLEGE STUDENTS

Xiaoxue Liu^a^, Yanfen Zhang^b^, Xuan Liu^c^, Hongbo Xu^a^, Lu Lai^a^, Yuxiao Liu^a^

^*a*^ *Department of Physical Education, China University of Geosciences (Beijing), Beijing 100083, China,* ^*b*^ *Cell Biology Research Laboratory, Xinxiang Medical University, Xinxiang 453004, China,* ^*c*^ *Universiti Sains Malaysia, Penang 11800, Malaysia.*

**Background:** This study aims to explore the effects of different organizational forms of Tai Chi on mobile phone addiction, mindfulness, emotional regulation abilities, and self-regulated learning among college students, in order to provide effective and feasible organizational strategies to mitigate mobile phone addiction and its associated impacts.

**Subjects and Methods:** A stratified random sampling method was used to select first- to third-year college students, who were asked to complete the Mobile Phone Addiction Index (MPAI) and the Physical Activity Rating Scale (PARS-3). Students with a mobile phone addiction score exceeding 40 points were randomly assigned to one of three Tai Chi intervention groups: a teacher-assigned leader Tai Chi group (72 students), a student-elected leader Tai Chi group (71 students), and a self-organized Tai Chi group with voluntary check-ins (71 students). Mindfulness and emotional regulation abilities were assessed using the Five Facet Mindfulness Questionnaire (FFMQ) and the Scale of Regulatory Emotional Self-Efficacy (SRESE), respectively. The tests were conducted three times: before the intervention, after the intervention, and two months following the intervention.

**Results:** The Tai Chi intervention with a student-elected leader significantly improved mobile phone addiction, mindfulness, and emotional regulation in college students (*P*<0.01). The student-elected leader group also outperformed both the teacher-assigned leader group (*P*<0.05) and the self-organized group (*P*<0.05). Follow-up research conducted six months post-intervention revealed that the student-elected leader group maintained lower levels of smartphone dependency for a longer period and showed sustained improvements in mindfulness and emotional regulation (*P*>0.05) compared to both the teacher-assigned and self-organized groups. Additionally, the self-organized Tai Chi group exhibited better results than the teacher-assigned group, especially in emotional regulation (*P*<0.05).

**Conclusions:** Tai Chi interventions led by a student-elected leader demonstrate superior organizational leadership, which helps promote the mental and physical health of college students, yielding more lasting and effective results. Furthermore, self-organized Tai Chi training also positively impacts mobile phone addiction, emotional regulation, and self-regulated learning in college students.

**Acknowledgments**

New Liberal Arts Project of China University of Geosciences (Beijing) (XWK202308).

## PHHS25003: KNOWLEDGE BASE, HOT TOPICS AND FRONTIER EVOLUTION OF PUBLIC HEALTH RESEARCH IN CHINA

Zaifeng Yang^a^, Ming Li^b^, Yanting Pan^a^

^*a*^ *Institute of Teacher Education, Wuzhou University, Wuzhou 543002, Guangxi, China,* ^*b*^ *Shaanxi Normal University, Xi’an 710062, Shanxi, China.*

**Background:** public health care social health important content. Public health is the cornerstone of social development, which is directly related to the economic prosperity and social stability of a country. In view of the progress of public health in China, its achievements provide many references for other countries. China’s successful experience in public health reform and innovation, especially in disease prevention and control, health education and health policy formulation, has provided valuable reference for developing countries. For example, the disease prevention and control system and sound health emergency response mechanism established in China have demonstrated the ability of rapid response and scientific management in response to public health emergencies such as COVID-19. These experiences have not only played a positive role in China’s own public health security, but also provided other countries with a viable model for responding to similar challenges. At the same time, China’s exploration in improving the health level of the whole people, promoting basic medical coverage and paying attention to the integration of traditional Chinese and western medicine has Chinese characteristics, but its policy ideas and practice methods can provide inspiration for other countries, especially developing countries, in realizing universal health coverage and improving the quality of medical services.

**Subjects and Methods:** CiteSpace was used to analyze 1432 research articles about public health in the core database of China National Knowledge Net.

**Results:** The results show that 14 scholars from Anlu, Zeng Ziming, etc. are highly cited authors, and 7 highly cited literatures do not determine the knowledge base of this field; the hot topics of research are reflected in public health and emergency management, risk society and personal privacy, modernity and information acquisition, medical treatment and social service in ethnic areas, national role and development. The evolution path presents a more diversified development direction from public health equity to focus on public health fund input, and then attention to public health network public opinion.

**Conclusions:** In the future, the research on public health will continue to increase and the trend of diversified development will promote the continuous renewal and continuous differentiation of research, thus forming a richer public health knowledge system.

**Acknowledgments**

This paper is one of the stage achievements of the teaching content construction and practice (2408030616) of the course “Education Research Methods” under the comprehensive literacy orientation National Social Science Fund Planning Project: Research on Practical Model of Health and Nursing Integration Services Based on Data Empowermentin Western Regions (24XGL046); 2024 Annual Guangxi Philosophy and Social Science Research Project: Study on the Ethical Risks and Prevention of Artifcial Intelligence in Public Health (24ZHC001).

## PHHS25004: A STUDY ON PREVENTIONS FROM SCHOOL-BULLYING BASED ON DUAL PERSPECTIVES OF RULE OF LAW AND MENTAL HEALTH IN DAZHOU CITY

Zhengquan Liu^a,b^, Peiling Liao^b^

^*a*^ *Hunan Normal University, Changsha 410081, Hunan, China,* ^*b*^ *Dazhou Institute of Administration, Dazhou 635000, Sichuan, China.*

**Background:** In recent years, the continuous emergence of school bullying on the Internet has aroused widespread heated discussions in all walks of life, especially in the education and legal circles. School bullying sometimes would develop into serious juvenile delinquency cases. The reason why such phenomena frequently occur in current time is mainly not only due to the mental health of juveniles not got well psychological counseled, but also due to the rule of law thinking not been widely spread. Both of the above caused juveniles in school encountering bullying even violence. Therefore, it is a unavoidable must that the thinking of rule of law and mental health in the group of juveniles should be severely taken in account.

**Subjects and Methods:** Health psychology study in the realm on how psychological factors influence health and well-being, focusing on understanding behaviors, emotions, and beliefs that might impact people’s physical health. We can see from the above occurrence that school bullying would not only undermines the stability of the family structure of one or more parties, but also causes the juveniles of one or more families to interrupt the physical and mental health growth of juveniles, or even causes some young children enter wrong approaches of life, participate criminal activities, such as causing death or injury of the victim, and they will be admitted to specialized schools. comparison and statistics methods would be used during the course of this research project.

**Results:** The healthy growth of juveniles should be physical and mental together in a synchronous way. How to carry out cultural education and psychological counseling as the goal on the rule of law, to prevent and reduce the occurrence of bullying on campus, so as to avoid teenagers going astray, should arouse the great attention of all sectors of society, such as the juveniles’ families, communities, schools, and government departments. A protection system coming from the whole society for the physical and mental health care of juveniles should be established.

**Conclusions:** To prevent, reduce, and ultimately eliminate bullying among juveniles, culture education and the thinking of rule of law, play an important role in the enlightenment of human civilization, which should be noticed from both of the physical and mental health of teenagers growth in school. The campus is the most important focus to achieve this function, but the starting point and the end point should not be limited to the campus. As a systematic social project, the lives of juveniles and all the growth environments they can come into contact with should be optimized, including communities, families, and recreational activities, and attention should be paid to juveniles’ legal literacy, even including the preschool education.

**Acknowledgments**

This research was supported by General Project of the National Social Science Fund “Research on the Judicial Application of the Principle of Public Order and Good Customs from the Perspective of Legal Methodology”(23BFX041); Research on Countervailing Risks and Countermeasures in the Construction of Key Fields of the “the Belt and Road” Scientific Research Project of the Education Department of Hunan Province (21A0037); 2025 Project of Southwest Jiaotong University Sichuan Provincial Key Research Base for Mental Health Education: “A Study on Rule of Law Approach for Collaborative Optimization of Youth Mental Health Education between Family, School and Society - Based on Artificial Intelligence”.

## PHHS25005: A STUDY ON THE ROLE OF MUSIC PERFORMANCE EDUCATION IN PROMOTING THE MENTAL HEALTH OF COLLEGE STUDENTS

Jing Liu^a^, Dan Mao^b^

^*a*^ *Chongqing College of Humanities, Science & Technology, Chongqing 401520, China,* ^*b*^ *Chongqing Preschool Education College, Chongqing 404000, China.*

**Background:** Music performance education, as a key component of art education, not only develops students’ professional skills but also profoundly impacts their mental health. College students face increasing mental health challenges, with music students experiencing higher rates of anxiety and depression due to the high-stress nature of performance. The integration of music education and psychology has gained academic attention, supported by theories of emotional expression and flow experience. However, current music performance education lacks a comprehensive psychological support system, emotional regulation training, and adaptable teaching methods. This study explores the unique characteristics and mechanisms of music performance education in promoting college students’ mental health, providing theoretical and practical insights for integrating art education and mental health.

**Subjects and Methods:** This study focused on university music students, employing a mixed-methods approach. A questionnaire survey collected data on students’ performance anxiety, emotional regulation, and psychological resilience, while in-depth interviews examined the mechanisms through which music performance education influences mental health. The research investigated emotional regulation strategies, flow experiences, and psychological interventions, such as cognitive restructuring, improvisational music therapy, and systematic desensitization therapy. Classroom observations and case studies were used to evaluate the effectiveness of curriculum design and teaching model optimization in supporting mental health.

**Results:** Music performance education significantly promotes the mental health of college students. Music serves as an emotional medium, helping students alleviate anxiety and depression while enhancing psychological resilience through emotional regulation. Flow experiences during music performance improve learning motivation, well-being, emotional management, and adaptability. Integrating cognitive behavioral therapy and improvisational music therapy into teaching effectively reduces performance anxiety, boosts self-confidence, and enhances stage performance. Systematic desensitization therapy helps students gradually adapt to stage pressure, improving performance skills and mental health.

**Conclusions:** Music performance education is not only a skill-oriented discipline but also a vital tool for enhancing college students’ mental health. Through emotional regulation, flow experiences, and psychological interventions, it provides a comprehensive psychological support system. However, current education models require further optimization to better integrate psychological support, adapt teaching methods, and enhance educational outcomes. Future efforts should focus on designing mental health-oriented music performance curricula. Leveraging digital technology and interdisciplinary collaboration, educators can create a model that prioritizes students’ psychological development, achieving the dual goals of art education and mental health promotion.

## PHHS25006: THE INFLUENCE OF SLEEP QUALITY ON THE EXPLOSIVE POWER OF BASKETBALL PLAYERS

Yu Tian^a^, Liu Yang^b^, Cheng Peng^c^, Fan Yang^d^, Xiangjun Miao^e^

^*a*^ *Tsinghua University High School, Beijing, China,* ^*b*^ *School of Psychology, Shanghai University of Sport, Shanghai, China,* ^*c*^ *China Athletics College, Beijing Sport University, Beijing, China,* ^*d*^ *Tsinghua University Primary School, Beijing, China,* ^*e*^ *China Basketball College, Beijing Sport University, Beijing, China.*

**Background:** Sleep is a vital recovery mechanism for athletes, playing a critical role in restoring physical capacity and fostering mental resilience after training and competition. As an essential pillar of overall health, sleep supports not only athletic performance but also long-term physical and psychological well-being. Despite the high prevalence of sleep disturbances among athletes and the often-underestimated impact of poor sleep quality, research in this area remains limited. This study aims to delve into the relationship between sleep quality and explosive power in basketball players, shedding light on the underlying mechanisms that link rest, recovery, and peak performance.

**Subjects and Methods:** Sleep quality will directly affect the physical health of athletes, therefore, by monitoring the sleep of 10 basketball players for a week, and their vertical and horizontal explosive power were assessed and analyzed the following day. Sleep parameters, including sleep time, heart rate and the root mean square of successive R-R intervals (RMSSD), were compared across different sleep quality groups. Vertical explosive power was assessed by vertical jump, while horizontal explosive power was evaluated through a standing long jump and a 20-meter sprint.

**Results:** Significant differences were observed in sleep duration, peak heart rate, minimum heart rate, and RMSSD between the high and medium sleep quality groups compared to the low sleep quality group (*P<*0.05). Athletes in the high sleep quality group demonstrated markedly better performance in standing high jump and maximum reach-with-jump height than those in the low sleep quality group (*P<*0.05), highlighting the strong association between quality sleep and vertical explosive power. However, no significant differences were observed in horizontal explosive power among the different sleep quality groups. These findings underscore the critical role of adequate sleep in fostering both physical performance and physiological recovery, which are essential for maintaining overall physical and mental health in athletes.

**Conclusions:** Improved sleep quality has a positive impact on the vertical explosive power of the subjects, underscoring the critical role of restful sleep in optimizing athletic performance. Beyond its direct influence on physical capabilities, adequate sleep also supports mental well-being, enabling athletes to recover holistically and perform at their peak. This study emphasizes the importance of prioritizing sleep as a fundamental component of training strategies, particularly for enhancing vertical power output and overall health. In addition, it can also intuitively help researchers understand the physical health status and sports performance ability of athletes.

**Acknowledgments**

The author would like to express gratitude to all the staff involved in the experiment, the coaches and players of the men’s basketball team, as well as the equipment providers. Finally, thanks are extended to all the students who assisted in data collection and processing.

## PHHS25007: RESEARCH ON MUSIC THERAPY FOR COLLEGE STUDENTS’ MENTAL HEALTH ISSUES

Haitao Yan^a^, Yangnan Yang^a^

^*a*^ *Hezhou University, Hezhou, China.*

**Background:** Contemporary college students face a variety of psychological pressures such as academic stress, social difficulties, confusion over career planning, and emotional problems, which often lead to common psychological issues like anxiety, depression, low self-esteem, and sensitivity in interpersonal relationships. The occurrence rate and severity of these issues are concerning. Traditional methods of psychological health education and therapy in schools have significant limitations in addressing these problems. Some students may resist facing psychological issues, struggle with pressures related to life, love, and career, and fail to properly deal with these challenges.

**Subjects and Methods:** This study systematically reviews the theoretical framework of music therapy for college students, including its psychological, musicological, and neuroscientific foundations, and clarifies the mechanisms through which music therapy affects the mental health of college students. It delves into the practical methods and techniques of music therapy for college students, including the design and implementation of various therapy models, interventions, and activity forms. The study aims to explore personalized music therapy plans suitable for college students. It also conducts a comprehensive evaluation of the effectiveness of music therapy, using empirical research methods such as surveys, psychological tests, and interviews, to quantitatively analyze improvements in mental health indicators, psychological adjustment abilities, and quality of life. This will provide scientific evidence for the effectiveness of music therapy. The research methods include literature review, case analysis, empirical research, and questionnaire surveys to understand students’ awareness of music therapy, willingness to accept it, and expectations regarding treatment methods and content. This information provides real data support and targeted suggestions for the study.

**Results:** The study systematically outlines the theoretical framework of music therapy for college students, including its psychological, musicological, and neuroscientific foundations, and clarifies the mechanisms through which music therapy influences students’ mental health. It further explores the practical methods and techniques of music therapy, including the design and implementation of various therapy models, interventions, and activity forms, as well as the development of personalized music therapy plans. The effectiveness of music therapy is comprehensively evaluated using empirical methods, such as surveys, psychological tests, and interviews. The study quantifies improvements in mental health indicators, psychological adjustment abilities, and quality of life, providing scientific evidence for the therapy’s effectiveness. The research also forecasts the future development trends of music therapy for college students and proposes strategies and recommendations for its broader application and professional development in mental health education at universities. This will promote academic research and practical innovation in this field.

**Conclusions:** This paper aims to explore the theoretical foundation, practical methods, application effects, and future prospects of music therapy for college students. Through comprehensive literature analysis, case studies, and empirical investigations, it reveals the unique role and value of music therapy in enhancing students’ mental health, improving psychological adjustment abilities, and boosting overall qualities. The study provides new ideas and methods for mental health education in universities and encourages the widespread application and professional development of music therapy for college students.

## PHHS25008: CONSTRUCTION AND PRACTICAL EXPLORATION OF AN EVALUATION MODEL FOR DIGITAL LITERACY AND MENTAL HEALTH AMONG STUDENTS IN HIGHER VOCATIONAL COLLEGES

Feng Wu ^a^

^*a*^ *Guangdong Science and Trade Vocational College, Guangzhou, Guangdong 510430, China.*

**Background:** Enhancing the digital literacy of students in higher vocational colleges is a crucial issue in current educational fields. Firstly, it is an intrinsic requirement for talent cultivation. With the rapid development of information technology, digital literacy has become an indispensable basic ability in the modern workplace. Secondly, the external demand for enterprise digital transformation also urgently requires higher digital literacy among students in higher vocational colleges to adapt to the ever-changing market environment. Thirdly, improving digital literacy is conducive to helping students in higher vocational colleges alleviate psychological pressure arising from improper technology use or inadequate information processing abilities in the digital age.

**Subjects and Methods:** To comprehensively enhance the digital literacy skills of students in higher vocational colleges, this study constructs an evaluation model with three levels: basic, intermediate, and advanced, based on Bloom’s Cognitive Model and the Global Digital Literacy Framework. The model encompasses twelve capabilities across four dimensions: digital cognition, digital application, digital management, and digital thinking. It focuses on analyzing the extent of fear among vocational college students towards learning with new digital devices, their level of convenience and satisfaction in acquiring digital information, the degree of fear regarding the disclosure of digital identity and personal privacy, as well as the level of psychological anxiety in digital thinking and creation. Simultaneously, a “six-degree” enhancement strategy through the first, second, and third classrooms is proposed, specifically including policy guarantee (height), collaboration breadth, ability level gradient, training method precision, assessment system validity, and technical literacy depth.

**Results:** By implementing the aforementioned “six-degree” enhancement strategy, the digital literacy level of students in higher vocational colleges can be significantly improved. Their digital cognition, application, management, and thinking abilities will all be enhanced, greatly reducing the physical and mental anxiety and job-seeking pressure brought by digital technological advancements. This will lay a solid foundation for their future career development and better adapt them to the workplace demands of the digital age.

**Conclusions:** In summary, enhancing the digital literacy of students in higher vocational colleges is a common requirement for talent cultivation and enterprise digital transformation. By constructing an evaluation model with four dimensions and twelve competencies and implementing the “six-degree” enhancement strategy, students’ digital literacy levels can be comprehensively improved, reducing anxiety arising from technology fear or information overload. This also has a positive guiding role in the practice of higher vocational education.

**Acknowledgments**

2023 Guangdong Provincial Education Science Planning Project (Higher Education Special) “Research on Strategies for Enhancing Digital Literacy of Students in Higher Vocational Colleges under the Background of New Business Education” (2023GXJK778); 2023 Provincial Higher Vocational Education Teaching Reform Research and Practice Project (2023JG268); 2024 School-level “Collaborative Innovation Center for Digital Commerce Technology” (GDKM2024-06).

## PHHS25009: FROM “INDIVIDUAL HEALTH” TO “PUBLIC HEALTH”: IMPACT OF NON-FARM EMPLOYMENT ON THE “ONE FAMILY, TWO SYSTEMS” PRODUCTION BEHAVIOR OF FARMERS

Chun Yang^a^, Yijin Li^a^, Xiang Chang^a^, Qiuyue Yuan^a^, Xianglin He^a^, Yixuan Zou^b^, Qian Chang^a^

^*a*^ *College of Management, Sichuan Agricultural University, Chengdu 611130, Sichuan, China,* ^*b*^ *Hong Kong Shue Yan University, Hong Kong, China.*

**Background:** As the internal and external environments for agricultural development change significantly, food safety incidents occur frequently, seriously affecting consumer trust and public health concerns. In this context, farmers, as important members of the economy and society, have the dual identity of “producer and consumer” of agricultural products, and usually adopt differentiated production strategies to cope with food health issues. With the rapid development of the economy, non-farm employment has become an important way for farming families to maintain their livelihoods and improve their standards of living. From the perspective of non-agricultural employment, it is important to explore the influence mechanism of “one family, two systems” (OF-TS) production behavior of farm households, in order to promote food safety from “individual health” to “public health”.

**Subjects and Methods:** Based on the China Rural Revitalization Survey (CRRS) Database, Logit, propensity score matching (PSM) and mediation effect models were employed in this study to investigate the influence of non-farm employment on the dual production behavior of farmers with a view to shifting from “individual health” to “public health”. In addition, how variations in the stability, location and intergenerational nature of non-farm employment shape these effects was further explored.

**Results:** Based on a public health perspective, the analysis yielded four key findings. First, non-farm employment disincentivizes OF-TS production behavior adopted by farmers to safeguard their health. This conclusion remains robust after endogeneity concerns are addressed. Second, non-farm employment has a more pronounced impact on farmers with lower educational attainment and higher income levels. Third, local non-farm employment and employment by older generations exert a stronger effect on the mitigation of dual production behavior compared with non-local employment and younger generational participation. Finally, non-farm employment curbs dual production behavior by reducing the dependence on outsourced agricultural services.

**Conclusions:** Against the backdrop of public health concerns arising from food safety incidents, it is recommended that policymakers should harness the potential of non-farm employment to enhance farmers’ income, strictly regulate agricultural production activities, promote a shift from safeguarding “individual health” to safeguarding “public health” and ensure the safety of agricultural products flowing into the market. Moreover, efforts should focus on strengthening the agricultural social service system to further support this transition.

**Acknowledgements:**

This study is sponsored by National Natural Science Foundation of China (NSFC) Youth Program: Research on the Mechanism through which Agricultural Service Outsourcing Influences the Eco-Efficiency of Grain Production under China’ s Carbon Peaking and Carbon Neutrality Goals (No. 72303168) and Natural Science Foundation of Sichuan Province: Research on the Assessment and Enhancement Mechanism of the Eco-Efficiency of Grain Production in the “Tianfu Granary” (No. 2024NSFSC1083).

## PHHS25010: RESEARCH ON THE CONSTRUCTION OF A TARGETED GUIDANCE MODEL FOR UNIVERSITY PUBLIC OPINION GOVERNANCE AND THE OPTIMIZATION OF NEGATIVE EMOTION REGULATION STRATEGIES

Chunhong Zhang^a^, Yali Liu^a^, Ruobing Liu^a^

^*a*^ *Faculty of Information Engineering, The College of Arts and Sciences·Kunming, Kunming, Yunnan 650222, China*.

**Background:** Due to long-standing structural issues within the campus governance system and unresolved specific needs, negative emotions have accumulated and erupted, ultimately evolving into online public opinion crisis. This paper aims to establish an effective management framework for university public opinion governance and optimize strategies for public opinion guidance, with the goal of preventing and addressing such crises and creating a harmonious and stable campus environment.

**Subjects and Methods:** The research object is the public opinion governance system in university campuses. The specific method proposed is to add a “Petition Office”, constructing a “0-1” progressive governance model with the Petition Office, Publicity Department, and Information Office as the core. In the initial stage of public opinion governance, the early warning mechanism of the Petition Office is used to identify problems, eliminate negative emotional risks, and detect and address potential public opinion risks in a timely manner. In the development stage of public opinion governance, for the Petition Office, Publicity Department, and Information Office, it is recommended to apply online questionnaire surveys platform, online message complaints system, agenda setting, gentle public opinion guidance, AI intelligent customer service, and other public opinion guidance strategies. This enables rapid response and precise information dissemination during public opinion incidents, effectively guiding the direction of public opinion.

**Results:** The constructed ‘0-1’ advanced governance model enables universities to respond swiftly and communicate effectively during public opinion crises. In daily management, it also plays a proactive role in prevention and correct guidance. For instance, the early warning mechanism of Petition Office successfully identified and mitigated multiple potential public opinion risks at germination stage, curbing negative emotions in their infancy; during public opinion development stage, the comprehensive application of various guiding strategies effectively directed public sentiment, minimizing the negative impact of public opinion incidents to the greatest extent.

**Conclusions:** The ‘0-1’ advanced governance model centered on the Petition Office, Publicity Department, and Information Office facilitates rapid response and effective communication for universities during crisis management of public opinion, and ensures proactive prevention and appropriate guidance in daily administration. This model provides robust support for creating a harmonious and stable campus environment. It offers feasible and effective solutions for addressing public opinion crises triggered by negative emotions in universities, with potential for promotion and further refinement within the field of campus governance.

**Acknowledgement:** This work was supported by a project grant from Multi-agent modeling in the model construction and application of public opinion management among college students, the Education Department of Yunnan Province (Grant No. 2023J1379).

## PHHS25011: THE DOUBLE-EDGED SWORD EFFECT OF INDUSTRY-EDUCATION INTEGRATION: THE SHAPING MECHANISM OF INTERNSHIP STRESS ON CAREER ANXIETY AND PROFESSIONAL IDENTITY

Xiao Peng^a^, Qiang Xie^a^, DeLong Tong^b^, Bo Hu^c^, Ting Pan^d^, XiaoKe Yin^a^

^*a*^ *Hunan Biological and Electromechanical Polytechnic, Changsha 410127, China,* ^*b*^ *Fushun Vocational Technical Institute. Fushun 113122, China,* ^*c*^ *Beiya Middle School of Changsha, Changsha 410005, China,* ^*d*^ *Hunan Dizhi Middle School, Changsha 410000, China.*

**Background:** Amidst China’s intensifying industry-education integration policy, internships have emerged as a pivotal link bridging educational systems and industrial demands. This connection exhibits a multifaceted and dynamic complexity, particularly in terms of the stress effects it induces. To delve into this phenomenon, the present study adopts a framework that integrates the Cognitive Appraisal Theory of Stress and the Career Ecosystems Perspective, aiming to explore the dual-edged sword effect of internship stress on career anxiety and professional identity.

**Subjects and Methods:** A mixed-methods approach was employed in this study. Quantitative data were collected from 1,268 interns across 12 applied universities in the Yangtze River Delta region. Additionally, qualitative insights were gathered through interviews with 32 interns and 23 mentors.

**Results:** The quantitative analysis revealed several key findings: (1) an inverted U-shaped relationship was observed between internship stress and career anxiety (β=-0.21, p<0.01), with a critical threshold identified at 52 weekly working hours. (2) A curvilinear mediation effect of professional identity was noted (indirect effect 95%CI = [0.08, 0.14]), where moderate levels of stress enhanced career commitment through pathways involving skill acquisition, self-efficacy, and identity reinforcement. (3) Significant moderating effects were found for institutional support (β=0.33, p<0.001) and enterprise mentoring efficacy (β=0.27, p<0.01). The qualitative analysis further uncovered that corporate subcultures emphasizing “performance supremacy” exacerbated role conflicts, while overprotective academic management posed “deskilling” risks.

**Conclusions:** Theoretically, this study introduces the “Stress Functional Zone” framework, which transcends traditional linear stress paradigms. Practically, it advocates for dual-path strategies: “progressive stress loading” and “elastic identity construction,” to optimize internship ecosystems. These findings offer valuable insights for policymakers, enabling them to refine risk governance mechanisms within the context of industry-education integration.

## PHHS25012: THE INFLUENCE OF OBE TEACHING MODE UNDER THE EMPOWERMENT OF NEW QUALITY PRODUCTIVITY ON THE MENTAL HEALTH OF HIGHER VOCATIONAL STUDENTS

Limin Xu ^a^

^*a*^ *School of Medicine, Huainan Union University, Huainan, Anhui 232000, China.*

**Background:** This study delves into the impact of integrating new productive forces and an artificial intelligence (AI)-supported outcomes-based education (OBE) teaching model on the mental health of higher vocational students, particularly those majoring in nursing and midwifery. The rapid advancements in technology and the evolving educational landscape necessitate a reevaluation of traditional teaching methods to better cater to the needs of contemporary students.

**Subjects and Methods:** To investigate this, a sample of 200 students from higher vocational colleges was selected. The study employed a control group that adhered to the conventional mixed teaching method, which combines various instructional strategies. In contrast, the experimental group embraced an OBE teaching approach enhanced by AI technologies. The AI-supported model was designed to personalize the learning experience, focusing on pre-class preparation, in-class engagement, and post-class reinforcement. The teaching effects of both groups were meticulously compared across multiple dimensions, including knowledge understanding and memory, skill strategy development, emotional attitude cultivation, and innovative thinking training. The AI-assisted personalized teaching path was seamlessly integrated throughout these stages, incorporating situational cases that reflect the demands of new productivity. This approach aimed to equip students with the skills and knowledge necessary to navigate the complexities of the modern workforce.

**Results:** The experimental group showed significant advantages over the group in scores related to understanding and memory, skills strategies, emotional attitude, and innovative thinking, and all differences were statistically significant.

**Conclusions:** The implementation of a teaching method that harmonizes new quality productivity with AI-enabled OBE in the nursing psychology course of higher vocational colleges has proven to be highly effective. This approach not only enhances students’ knowledge memory and coping strategies but also bolsters their innovative thinking abilities. By fostering a more personalized and engaging learning environment, the AI-supported OBE model contributes to improved mental health levels among students. Furthermore, it promotes the advancement of nursing psychology education in higher vocational colleges, aligning it with the demands of the modern healthcare industry. This study underscores the potential of integrating new technologies and innovative teaching methodologies to revolutionize education and support the holistic development of students.

## PHHS25013: THE INTERVENTION EFFECT AND MECHANISM OF SPORTS EXERCISE ON ANXIETY AMONG COLLEGE STUDENTS

Wu Chen^a^, Qian Xiong^a^

^*a*^ *Wuhan Railway Vocational College of Technology, Wuhan, China.*

**Background:** In recent years, the mental health of college students has attracted much attention. Factors such as academic pressure, employment pressure, and interpersonal relationship problems lead to a high incidence of psychological problems such as anxiety and depression among college students. As a low-cost, easy-to-implement and effective intervention, sports play an important role in promoting the mental health of college students. With the intensification of social competition, college students are faced with multiple stressors such as study and employment, and the detection rate of anxiety continues to rise. As a non-drug intervention means, sports has unique advantages in promoting mental health, and exploring its intervention mechanism is of great practical significance for improving the mental health education system in colleges and universities.

**Subjects and Methods:** This study aims to reveal the correlation characteristics of physical exercise and stress anxiety, analyze the anti-ytic mechanism of exercise from the dual perspectives of neurobiology and psychological regulation, and provide a theoretical basis for the development of scientific intervention programs. Using a cross-sectional study design, we surveyed 386 current students through stratified sampling. The stress Perception Scale (PSS) and physical activity rating scale (PARS-3) were used for quantitative evaluation, and the data were combined with Pearson correlation analysis and multiple regression model.

**Results:** The data showed that: 1) the average weekly exercise volume was significantly negatively related with anxiety level (r = -0.42, p < 0.01); 2) the high-intensity exercise score was significantly lower than the low-intensity group (t = 3.78, p < 0.001); 3) exercise played a mediation effect by regulating cortisol secretion (β = -0.31) and improving self-efficacy (β = 0.27).

**Conclusion:** Sports are an effective way to promote the mental health of college students. Colleges and universities and college students should pay attention to the role of sports, actively participate in physical exercise, and face study and life with a more positive and healthy attitude. Regular physical exercise can effectively improve the neuroendocrine regulation function of college students and enhance the psychological resilience, which is a feasible intervention strategy to prevent and treat anxiety disorders. It is suggested that colleges and universities should build a sports prescription system between inside and outside class, and incorporate physical education into the routine program of mental health promotion.

## PHHS25014: BRIDGING THE DIGITAL DIVIDE IN GLOBAL EDUCATION: HOW DIGITAL ECONOMY DEVELOPMENT INFLUENCES EQUITY IN HIGHER EDUCATION AND STUDENT MENTAL HEALTH

Ji Zhang^a^, Guofeng Zhao^b^

^*a*^ *Zhejiang Wanli University, Ningbo 315100, China,* ^*b*^ *Ningbo Polytechnic, Ningbo 315100, China.*

**Background:** The accelerated growth of the digital economy has transformed higher education by enabling new learning avenues, yet a persistent digital divide exacerbates inequalities. By 2023, global internet penetration reached 67% (5.36 billion people), per the International Telecommunication Union, while 33% (2.6 billion) remain offline, notably in least developed countries (LDCs), landlocked developing countries (LLDCs), and small island developing states (SIDS). This gap limits educational access, particularly in low-income regions, a challenge highlighted during the COVID-19 shift to online learning. Beyond access, this disparity may impact students’ mental health, increasing stress and isolation where digital resources are scarce. This study examines how digital economic development, proxied by Individuals Using Internet (IUI), influences higher education fairness, measured by gross enrollment ratios (GER), across a global sample, addressing its broader implications amidst globalization.

**Subjects and Methods:** The research uses panel data from over 200 countries (2014-2023), sourced from UNESCO (GER), ITU (internet usage), and the World Bank (controls: population, GDP per capita, income levels). A fixed-effects regression model, validated by a Hausman test, assesses the effect of internet usage on GER, controlling for country-specific heterogeneity. Robustness is ensured via lagged variables and instrumental techniques to address endogeneity, with standard errors clustered by country. Grouped regressions by income level (low, lower-middle, upper-middle, high) explore effect variations, supported by descriptive and correlation analyses. The study also considers how digital access might indirectly support student well-being by reducing educational barriers.

**Results:** Internet usage significantly boosts GER (coefficient=0.16, p=0.001), supporting human capital and information society theories linking digital access to equity. Grouped regressions show the effect peaks in lower-middle-income countries (0.230, p<0.001), followed by low-income (0.185, p<0.001), high-income (0.122, p<0.001), and upper-middle-income countries (0.089, p=0.052), with a Chow test (p=0.0433) confirming differences. GER ranges from 0.66% to 166.67% (mean=46.43%), and internet usage from 0.99% to 100% (mean=58.98%), reflecting disparities. These gains suggest digital access may enhance student well-being, including mental health benefits, by fostering inclusion and reducing learning-related stress.

**Conclusions:** Digital economic development promotes higher education fairness, most notably in lower-middle-income countries, offering a global perspective absent in prior studies. Policy should prioritize digital infrastructure in lower-middle-income nations, foundational support (e.g., electricity) in low-income settings, and skill initiatives in high-income countries to tackle the second-level digital divide. Such efforts could also support student well-being, aligning with UNESCO’s inclusive education goals. Limitations include potential endogeneity and GER’s quantitative focus, suggesting future research with digital literacy data and advanced methods.

**Acknowledgements:** This work was supported by 2024 Annual Ningbo Philosophy and Social Sciences Planning Project (Education Special Project), the Seventh Batch, titled “Concept, Mechanism, and Pathway: Research on Applied Undergraduate Talent Cultivation in Local Universities Based on German Experience” (Grant No. G24-7-JY10).

## PHHS25015: MID-INFRARED SPECTROSCOPY-BASED RAPID DETECTION OF SULFUR RESIDUES AND TOXICOLOGICAL SAFETY ASSESSMENT IN SULFUR-FUMIGATED GASTRODIA ELATA

Yan Cai^a^, Nan Xu^a^, Dianxu Ma^a^, Yuanzhao Shen^a^, Zhengyang Li^a^

^*a*^ *School of Physics and Information Engineering, Zhaotong University, Zhaotong 657000, China.*

**Objective:** This study aims to develop a rapid, non-destructive detection method based on Attenuated Total Reflection Mid-Infrared (ATR-MIR) spectroscopy, driven by analytical technology, for identifying sulfur residues in sulfur-fumigated Gastrodia elata and analyzing its pharmacochemical differences compared to non-fumigated samples. By addressing the limitations of traditional chromatographic methods in toxicological analysis, such as high complexity and low efficiency, this approach provides technical support for precise monitoring of sulfur residues and pharmaceutical quality control systems in traditional Chinese medicines, enhancing clinical medication safety and therapeutic stability.

**Methods:** An ATR-MIR spectroscopy platform integrated with difference spectrum method and exponential amplification method was employed to systematically collect pharmaceutical spectral fingerprints of sulfur-fumigated and non-fumigated Gastrodia elata. Preprocessing steps, including baseline correction, smoothing, and normalization, were applied to extract characteristic differences in pharmaceutical components using the difference spectrum method. The exponential amplification method was utilized to enhance sulfur-related toxicological markers (e.g., S=O and C-S vibrational peaks). Additionally, samples from multiple regions (Yunnan, Hubei, Guizhou) and counterfeit samples were analyzed to validate the method’s universality in pharmaceutical detection and clinical discrimination efficacy.

**Results:** Sulfur-fumigated Gastrodia elata exhibited specific toxicological absorption peaks at 1050 cm^-1^ (S=O stretching vibration) and 700 cm^-1^ (C-S vibration), confirming the formation of sulfate ester groups or thioether bonds as pharmaceutical by-products. A significant reduction in absorbance of natural active components (polysaccharides, lipids) was also observed (P<0.05). The difference spectrum and exponential amplification methods enhanced sulfur residue signals to ΔA≥0.3, enabling precise discrimination between samples from different regions and counterfeit products, with detection sensitivity (≥95%) and specificity (≥92%) meeting clinical pharmaceutical detection standards.

**Conclusions:** The developed ATR-MIR analytical technology provides an innovative solution for high-throughput screening and quantitative toxicological analysis of sulfur residues in sulfur-fumigated Gastrodia elata, offering advantages such as non-destructiveness, high efficiency, and clinical applicability. This method can be integrated into traditional Chinese medicine quality control systems and pharmaceutical safety monitoring networks. Future research should optimize sulfur residue quantification using pharmaceutical chemometric models, expand detection capabilities with pharmacometabolomics techniques (e.g., LC-MS/MS), and explore its potential in heavy metal toxicity assessment and pesticide residue monitoring, thereby promoting the standardization and safety management of the traditional Chinese medicine industry.

**Acknowledgements**

This work was supported by Yunnan Province Base Undergraduate University Basic Research Joint Special General Project (202301BA070001-094) and Yunnan Province Base Undergraduate University Basic Research Joint Special Youth Project (202301BA070001-122).

## PHHS25016: ACTION RESEARCH ON ENHANCING ENGLISH NARRATIVE COMPETENCE AND REDUCING LANGUAGE ANXIETY BASED ON “UDIG” DIAGNOSTIC ASSESSMENT

Yan Zou^a^, Jin Liang^a^

^*a*^ *College of Foreign Languages, Wuzhou University, Wuzhou 543000, Guangxi, China.*

**Background:** This study aims to enhance undergraduates’ English narrative skills while alleviating language anxiety through targeted instructional strategies, fostering confidence and fluency in expression. Using the “UDig” assessment tool, the study analyzes students’ narrative abilities, identifying key factors affecting narrative quality, such as language organization, logical coherence, and information coverage. Additionally, it explores integrating elements of Chinese traditional culture with the “Three-S” retelling strategy to optimize instructional interventions and strengthen language output. Beyond narrative skills, the study emphasizes the importance of motivating learners. Ultimately, this research provides empirical support for data-driven pedagogical improvements and lays the foundation for culturally responsive teaching models.

**Subjects and Methods:** This study involved 97 first-year law students from Wuzhou University. The “UDig” diagnostic assessment tool and a structured questionnaire were used to evaluate their English narrative competence, showing that most students were at a lower-intermediate level. To enhance their skills, an action research approach was adopted, integrating traditional Chinese cultural stories into English instruction. The intervention applied the “Three-S” retelling strategy and creative retelling techniques to strengthen narrative abilities. Additionally, the teaching activities focused on creating a supportive learning environment to alleviate language anxiety and boost students’ confidence in oral expression.

**Results:** The results indicate that integrating Chinese traditional cultural stories with systematic retelling strategies significantly enhances students’ English narrative abilities. Through narrative training within a familiar cultural context, students not only demonstrate notable improvements in linguistic fluency and coherence but also experience a substantial reduction in language anxiety. Moreover, the approach fosters increased motivation for language learning and cultivates a more positive attitude toward English acquisition. These results suggest that culturally embedded pedagogical strategies can serve as an effective means of facilitating second language development by leveraging learners’ prior knowledge and emotional engagement. Future studies could further explore the long-term effects of this approach and its applicability across diverse linguistic and educational settings.

**Conclusions:** This study demonstrates that an action research approach integrating the “Three-S” retelling strategy with creative narrative techniques effectively enhances students’ English narrative abilities. The incorporation of Chinese traditional cultural elements not only strengthens students’ narrative skills but also alleviates language anxiety to some extent, thereby fostering both linguistic development and psychological well-being. The findings suggest that culturally responsive pedagogy serves as an effective strategy to improve English learning outcomes while simultaneously promoting a more positive emotional state among students. Future research could further investigate the long-term efficacy of this approach, its adaptability across different learner profiles, and its potential applications in various educational contexts.

**Acknowledgements**

This work was supported by a project grant from Guangxi Education Science “14^th^ Five-Year Plan” 2023 Special Research Project on Foreign Language Teaching Reform under the “Three Integrations” Initiative (Grant No.2023ZJY2401).

## PHHS25017: A STUDY ON THE STRUCTURAL EQUATION MODEL OF MOTHERS’ EDUCATIONAL LEVEL, MEDICAL RESOURCE UTILIZATION AND RURAL CHILDREN’S HEALTH

Xiaoxue Cai^a^, Ronghui Liu^b^, Xiaoli Yi^b^, Wenyi Xu^b^

^*a*^ *Jiangsu Huaihai Technician Institute, Jiangsu, China,* ^*b*^ *Huaihua normal college, Hunan, China.*

**Background:** Persistent rural-urban pediatric health disparities necessitate investigation of modifiable pathways linking maternal education to child health. While maternal education is recognized as a critical social determinant, the mechanisms mediating its effects in resource-limited settings remain underexplored. This study examines medical service utilization as a mediator and satisfaction with healthcare resources as a moderator in rural China—a context marked by educational and healthcare access inequalities. The investigation addresses a critical knowledge gap regarding how healthcare system factors interact with individual socioeconomic advantages to shape intergenerational health outcomes.

**Subjects and Methods:** A cross-sectional design employed multi-stage stratified sampling to recruit 652 mother-child dyads across 12 Chinese provinces, ensuring geographic and socioeconomic representativeness. Data collection utilized validated instruments: 1) Maternal education categorized per UNESCO’s ISCED framework, 2) Medical Utilization Index quantifying service access frequency/appropriateness, 3) Multidimensional Medical Satisfaction Scale assessing infrastructure/staffing/affordability, and 4) Pediatric health outcomes measured through standardized physical/mental health assessments. Structural equation modeling (SEM) with maximum likelihood estimation evaluated direct/indirect pathways and moderation effects, controlling for household income and regional development indices.

**Results:** Firstly, Maternal education directly predicted medical utilization (β=0.48, p<0.001), explaining 34% of variance (R²=0.34). Medical utilization mediated 61.7% of education’s total effect on physical health (β=0.32, p<0.01) and 57.9% on mental health (β=0.27, p<0.05), with significant indirect effects (Sobel test Z=4.12, p<0.001).

Thirdly. Satisfaction levels moderated the education-utilization relationship (ΔR²=0.11, p=0.003), intensifying education’s impact in high-satisfaction contexts (βhigh=0.63 vs. βlow=0.31, pinteraction=0.008). Each educational year completed reduced children’s preventable hospitalization risk by 19% (OR=0.81, 95%CI=0.70-0.94).

**Conclusions:** This study elucidates a dual mechanism where maternal education enhances healthcare navigation capabilities, while medical satisfaction amplifies these advantages through improved service accessibility. The findings provide empirical support for integrated health equity strategies: (1) Targeted health literacy programs emphasizing preventive care and system navigation for less-educated mothers; (2) Equity-focused medical resource allocation prioritizing underserved regions; (3) Community-driven quality improvement initiatives addressing service affordability and provider-patient communication. The demonstrated moderation effect suggests that enhancing medical satisfaction could potentiate existing education-based interventions, offering a multiplier strategy for health disparity reduction. Future research should employ longitudinal designs to examine temporal dynamics and evaluate policy interventions simultaneously targeting maternal education and healthcare system optimization. These evidence-based insights contribute to global efforts in achieving Sustainable Development Goal 3 (health equity) within rural populations.

**Acknowledgements:** This work was supported by a project grant from Research on the Development of Inclusive Kindergartens in Rural Areas under the Background of Population Changes“ [XJK24CJC008] in the “14th Five-Year Plan” for Educational Sciences in Hunan Province. & Research on the Sustainable Development Strategies of Preschool Education Majors under the Background of Negative Population Growth” [XJK24BGD049] in the “14th Five-Year Plan” for Educational Sciences in Hunan Province.

## PHHS25018: FUNCTIONAL, AESTHETIC, AND SAFETY EVALUATION OF INVISIBLE BRACKET-FREE ORTHODONTIC TREATMENT IN FEMALE PATIENTS WITH SKELETAL CLASS I MALOCCLUSION

Dapeng Liu ^a^

^*a*^ *Department of Stomatology, Handan Aiyan Ophthalmic Hospital, Handan 056000, Hebei, China.*

**Background:** Orthodontic treatments have evolved significantly over the years, with advancements aiming to improve functionality, aesthetics, and patient comfort. One such innovation is invisible bracket-free orthodontic treatment, which has garnered attention for its potential benefits in female patients with skeletal class I malocclusion. This study was undertaken to comprehensively evaluate the effectiveness of this novel approach compared to traditional self-ligating bracket orthodontics in terms of functionality, aesthetics, and safety.

**Subjects and Methods:** A total of 108 female patients diagnosed with skeletal class I malocclusion were selected from those treated at Handan Aiyan Ophthalmic Hospital. The patients were randomly divided into two groups using a random number table method: an observation group and a control group, each comprising 54 patients. The observation group underwent invisible bracket-free orthodontic treatment, while the control group received self-ligating bracket orthodontic treatment. The evaluation criteria included functionality (masticatory function and speech function), comfort, and aesthetic aspects of the orthodontic appliances.

**Results:** The results indicated that the observation group scored higher in masticatory function and speech function compared to the control group, with statistically significant differences (P<0.05). Post-treatment assessments revealed improvements in both groups concerning occlusal contact time, center of occlusal force position, and occlusal force, but the observation group exhibited better improvements than the control group, showing statistically significant differences (P<0.05). In terms of gingival health, the gingival papilla index grade in the observation group was lower than that in the control group post-treatment, with statistically significant differences (P<0.05). Additionally, the observation group demonstrated superior oral aesthetics, oral hygiene, gingival aesthetics, and overall aesthetics compared to the control group, with statistically significant differences (P<0.05). The incidence of adverse reactions was also lower in the observation group than in the control group, with statistically significant differences (P<0.05).

**Conclusions:** The findings of this study suggest that invisible bracket-free orthodontic treatment offers several advantages over traditional self-ligating bracket orthodontics in female patients with skeletal class I malocclusion. These advantages include improved masticatory and speech functions, enhanced aesthetic outcomes, higher patient satisfaction, and fewer adverse reactions. Therefore, invisible bracket-free orthodontics can be considered a worthy clinical application for this specific patient demographic. Further research may be needed to generalize these findings to other types of malocclusions and patient populations.

## PHHS25019: APPLICATION OF MICRO-IMPLANTS FOR ORTHODONTIC ANCHORAGE IN FEMALE PATIENTS WITH ANTERIOR INCLINATION MALOCCLUSION OF THE UPPER LIP TEETH

Zhenyu Ji ^a^

^*a*^ *Department of Stomatology, Shijiazhuang Aiyan Ophthalmic Hospital, Shijiazhuang 050000, Hebei, China.*

**Background:** To observe the application effect of micro-implants for orthodontic anchorage in female patients with anterior maxillary incisor proclination and malocclusion.

**Subjects and Methods:** A total of 109 female patients with anterior maxillary incisor proclination and malocclusion were selected from the hospital and divided into an observation group (53 cases) and a control group (56 cases) using a random number table method. The observation group underwent orthodontic treatment using micro-implants for anchorage, while the control group was treated with transpalatal arches and Nanc bows for additional support. After treatment, the overbite (OB), overjet (OJ), aesthetic component scale (IOTN-AC), periodontal microenvironment, patient satisfaction, and complication rates were compared between the two groups before and after treatment.

**Results:** Before treatment, there were no significant differences between the two groups in OB/OJ levels, aesthetic scores, MMP-2 and MMP-9 levels, periodontal index scores, X-ray indicator levels, IL-6, IL-1β, and TNF-α levels (P>0.05). After treatment, both groups showed a decrease in OB and OJ levels, with the observation group showing lower levels than the control group (P<0.05). After treatment, the aesthetic scores of both groups were lower than before treatment, and the observation group had lower scores than the control group (P<0.05). After treatment, the levels of MMP-2 and MMP-9 in both groups increased, but the increase was smaller in the observation group compared to the control group (P<0.05). Following treatment, both groups improved in periodontal index scores, with the observation group showing greater improvement than the control group (P>0.05). Both groups showed a reduction in X-ray indicators after treatment, with the observation group having lower levels than the control group (P<0.05). The satisfaction level of patients in the observation group was higher than that of the control group (P<0.05). After treatment, both groups showed an increase in IL-6, IL-1β, and TNF-α levels, but these levels were lower in the observation group compared to the control group (P<0.05).

**Conclusions:** The use of micro-implants for orthodontic anchorage in female patients with anterior maxillary incisor proclination and malocclusion shows good clinical effects and is worthy of widespread clinical application.

## PHHS25020: EXCESS CONTROL RIGHTS OF LARGE SHAREHOLDERS ON THE PSYCHOLOGICAL ANXIETY OF SHAREHOLDERS, BASED ON THE PERSPECTIVE OF STOCK PRICE CRASH RISK

Weichao Cheng^a^, Yi Zhang^b^

^*a*^ *West Anhui University, Anhui, China,* ^*b*^ *Sichuan University Jinjiang College, Sichuan, China.*

**Background:** The collapse of stock price will quickly evaporate the wealth of shareholders, greatly increasing their anxiety. Anxiety can have a dual impact on people’s physical and mental well-being, Anxiety can bring complex and unpleasant emotions such as tension, unease, worry, annoyance, Insomnia and dreaminess to people, thereby affecting family harmony and happy life. A sound and stable capital market is of great significance for promoting the rapid, sustainable and high-quality development of China’s economy. Although China’s capital market has achieved leapfrog development, it started relatively late, and there is still room for improvement in aspects such as market supervision mechanisms and corporate legal systems. This has led to the frequent occurrence of behaviors such as fraudulent issuance by listed companies, stock price manipulation, insider trading, major shareholders’ embezzlement of corporate interests, financial fraud, and violations of information disclosure. These illegal acts have persisted despite repeated bans, resulting in relatively insufficient stability in China’s capital market and a higher risk of stock price collapses of listed companies.

**Subjects and Methods:** This paper using the Shapley-Shubik power index, which is based on cooperative game theory, and investigates the impact of excess control rights of major shareholders on the psychological anxiety of shareholders. This study takes A-share listed companies in China from 2003 to 2018 as the research sample. And uses regression methods such as least squares to empirically test the research hypotheses proposed in this paper.

**Results:** The study finds that: (1) the excess control rights of major shareholders significantly increase the stock price crash risk, increased the anxiety of shareholders. Excess control rights have a significant “crash effect” and act as an “accelerator” of stock price crashes. (2) Excess control rights have a significant “crash effect” on both state-owned and non-state-owned enterprises, and the “crash effect” is more significant in the non-state-owned enterprise group. (3) Regardless of whether the stock market experiences a bullish or a bearish market, excess control rights have a significant “crash effect”, and it is more significant in the bearish market group.

**Conclusions:** The excess control rights significantly increased the anxiety of shareholders, the findings of this paper could provide useful theoretical and practical guidance for optimizing firms’ shareholding structure, suppressing the risk of share price collapse, improving the quality of listed companies, promoting the stability of capital markets, and alleviating the anxiety of shareholders.

**Acknowledgments**

This work was supported by the West Anhui University Talent Introduction School Research Start-up Fund Project (No. 00701092302).

## PHHS25021: EXPLORING THE PROMOTION OF STUDENTS’ MENTAL HEALTH BY CONSTRUCTING A VOCATIONAL CORE COMPETENCY-ORIENTED CURRICULUM SYSTEM OF HIGHER VOCATIONAL PUBLIC ELECTIVE COURSES

Li Xiang ^a^

^*a*^ *Zhenjiang Branch of Jiangsu Union Technical Institute, Zhenjiang, Jiangsu, China.*

**Background:** With the continuous development of society, vocational education faces increasingly complex challenges. In higher vocational colleges and universities, cultivating students’ vocational core competence is one of the core tasks of cultivating qualified skilled personnel. However, as the pressure of students’ employment increases and the psychological challenges in their careers become more and more obvious, students’ mental health problems have gradually become the focus in education management. Therefore, how to pay attention to students’ mental health while cultivating their vocational core competencies has become a key issue in the educational reform of higher vocational colleges and universities. The construction of a public elective course system oriented to vocational core competence can not only effectively improve students’ professional competence, but also promote the improvement of students’ psychological quality, laying a solid foundation for students’ future career development.

**Subjects and Methods:** This study designed a set of public elective course system oriented to vocational core competence for the characteristics of higher vocational colleges and universities, combined with the needs of mental health education. The content of the courses covers three aspects: vocational skills training, vocational literacy enhancement and mental health education. In terms of research methodology, questionnaire surveys, in-depth interviews and case studies were used to evaluate the implementation effect of this curriculum system. The questionnaire survey focuses on students’ vocational ability and mental health status, while the interviews further understand students’ feedback and evaluation of the course content, teaching methods, and mental health modules. Through the comprehensive analysis of these data, the effectiveness of the course system in enhancing students’ vocational ability and psychological quality was assessed.

**Results:** The results of the study show that the public elective course system oriented on vocational core competencies has achieved significant results in enhancing both students’ vocational skills and mental health. First, the curriculum system enhanced students’ professional knowledge, teamwork ability, communication skills and problem solving ability and other occupational core competencies through diversified teaching contents and methods. Secondly, the mental health education module helped students enhance their self-knowledge and improve their ability of emotion management and stress regulation. In particular, when facing workplace pressure and challenges, students’ stress resistance, resilience and self-confidence were significantly improved. In addition, students generally reflected that the course content helped them better understand and deal with psychological problems in their careers and enhanced their mental health.

**Conclusions:** Constructing a public elective course system oriented to vocational core competencies can effectively enhance students’ vocational literacy and mental health. By systematically designing a curriculum system that combines vocational skills and mental health education, it not only helps students enhance their core vocational competencies, but also strengthens their psychological adjustment ability, which provides a solid support for them to face various challenges in the workplace in the future. Therefore, it is recommended that higher vocational colleges and universities further strengthen the integration of vocational competency training and mental health education in curriculum design to create a more comprehensive and sustainable educational environment and lay the foundation for students’ individualized development and career.

## PHHS25022: CONSERVATION PATHWAYS IN NATURE RESERVES BASED ON PSYCHOLOGICAL EXPECTATIONS: A MENTAL HEALTH PERSPECTIVE ANALYSIS

Wei Hu ^a^

^*a*^ *Anyang Normal University, Anyang 455000, Henan, China.*

**Background:** The current vertical management model of nature reserves, which is often implemented in the context of the dual challenges of environmental protection and economic development, has been observed to ignore the psychological expectations of local residents regarding their right to live. This has been identified as a contributing factor to the emergence of mental health problems such as anxiety and panic, which in turn threatens the sustainability of the reserves. The objective of this study is to examine and enhance management strategies from a mental health standpoint, with the aim of achieving the dual objectives of environmental conservation and the promotion of residents’ mental wellbeing.

**Subjects and Methods:** This study focuses on the subjectivity of local residents in the context of a co-management model for protected areas, and the impact of their psychological expectations regarding the right to exist on the management process. Psychological theories and empirical analyses were employed to evaluate the existing management model and to investigate the residents’ vision of their participation in conservation management. The relationship between residents’ psychological expectations and protected area management is analyzed through the use of quantitative surveys and qualitative interviews.

**Results:** The study demonstrates that the co-management model can effectively reconcile the rights to a healthy environment and to survival, while also enhancing residents’ motivation to participate in conservation by meeting their psychological expectations, such as those related to exploitative conservation and compensation. This model enhances management efficiency, mitigates residents’ anxiety, and bolsters psychological well-being.

**Conclusions:** This study underscores the vital importance of addressing the psychological expectations of residents with regard to their right to subsistence, as a means of alleviating tensions between management agencies and residents, and of enhancing the efficiency of conservation efforts. It is therefore evident that management strategies must give greater consideration to the psychological health of residents if the harmonious development of environmental protection and residents’ well-being is to be promoted.

**Acknowledgments**

This paper has been selected for funding by the 2012 National Social Science Foundation of China (NSFC): “Research on the Legal Mechanism of Community Co-management of Nature Reserves” (12BFX123). This paper has been selected for funding by the 2019 Henan Provincial Philosophy and Social Science Planning Program: “Justice and Revolution: a Study of the Tensions and Limits of Justice in the Central Soviet Union Region” (2019BFX001). This paper has been selected for funding by the 2024 Henan Provincial Philosophy and Social Science Planning Basic Program (Cultural Research Direction): “A Study of the 'Honor Harmony’ Litigation Source Governance Culture in the Ancient Central Plains Region” (2024XWH098).

## PHHS25023: DIGITAL TRANSFORMATION IN MUSIC COLLEGES: ENHANCING MENTAL HEALTH THROUGH TECHNOLOGICAL INNOVATION: A CASE STUDY OF ZHEJIANG CONSERVATORY OF MUSIC

Guohua Wang ^a^

^*a*^ *Zhejiang Conservatory of Music, Hangzhou, Zhejiang, China.*

**Background:** The rapid advancement of digital technology has revolutionized higher education, offering unprecedented opportunities to address contemporary challenges, including mental health. Music colleges, with their unique artistic and pedagogical characteristics, face distinct hurdles in integrating digital tools to support student well-being. This study examines the digital transformation at Zhejiang Conservatory of Music, focusing on how technological innovations can mitigate mental health issues by improving educational quality, accessibility, and personalized support. The research highlights the intersection of digitalization and mental health in art education, emphasizing the need for tailored solutions in music institutions.

**Subjects and Methods:** The study employs a mixed-methods approach, combining qualitative case analysis of Zhejiang Conservatory’s digital initiatives with quantitative data from student and faculty feedback. Key initiatives include the development of a big data warehouse for student performance tracking, a mobile app (“Zheyin on palm”) for streamlined academic and mental health services, and digital platforms like “Zheyin Academy” and “Zheyin Growth” to monitor and support student well-being. These tools integrate real-time data analytics to identify at-risk students, provide early interventions, and foster a supportive learning environment.

**Results:** The digital transformation significantly enhanced mental health support for students. Platforms like “Zheyin Growth” enabled continuous monitoring of academic and behavioral patterns, facilitating timely counseling and reducing stress. The “Happy Classroom” program extended mental health benefits to rural primary schools by providing accessible music education, alleviating anxiety among young learners. Online concerts and community engagement initiatives also promoted emotional well-being, reaching over 15 million viewers. These outcomes demonstrate the efficacy of digital tools in creating inclusive, mentally supportive educational ecosystems.

**Conclusions:** The study underscores the critical role of digital transformation in addressing mental health challenges in music education. By leveraging data-driven platforms and mobile technologies, Zhejiang Conservatory has set a precedent for integrating mental health support into digital frameworks. Future efforts should focus on bridging the digital divide and enhancing teacher training to maximize these benefits. This research provides a model for other art institutions to harmonize technological innovation with mental health advocacy, fostering holistic student development.

**Acknowledgments**

“Exploration of Digital Music Education Pathways in the New Era, “a development and independent research project funded by the Key Laboratory of Digital Music Intelligent Processing Technology of the Ministry of Culture and Tourism in 2023 (2023DMKLC008).

## PHHS25024: RESEARCH ON THE DEVELOPMENT OF COLD CHAIN AGRICULTURAL PRODUCTS SHIPPING AND DISTRIBUTION UNDER THE PERSPECTIVE OF CONSUMER PSYCHOLOGICAL NEEDS

Lei Jiang^a^, Xiaoyu Chen^b^

^*a*^ *Shandong Institute of Commerce and Technology, Jinan 250103, Shandong, China,* ^*b*^ *Shandong Management University, Jinan 250100, Shandong, China.*

**Background:** With the continuous expansion of the cold chain logistics market, the number of consumers of cold chain agricultural products also continues to grow. According to data, China’s cold chain logistics market has exceeded 380 billion yuan and is expected to exceed 550 billion yuan by 2025.This trend indicates that cold chain agricultural products have become an important part of consumers’ daily lives. However, with the expansion of the market scale, consumer demand for cold chain agricultural products is not only concerned about the products themselves, but also gradually shifts to the quality of logistics services behind them. Consumers’ psychological needs, including emotional needs, trust needs and security needs, have gradually become important factors influencing their purchasing decisions. Therefore, seizing consumers’ psychological needs will become the key for cold chain logistics enterprises to stand out in the fierce competition. Starting from the psychological needs of consumers in general, the article analyzes the decision-making behaviours of enterprises based on the AHP-grey correlation model, combines the psychological needs of consumers for cold chain agricultural products with their development paths, and focuses on the analysis of consumers’ emotional labourer and work pressure. The article focuses on solving the problems encountered in the development process of agricultural products distribution, such as high cost due to small and scattered logistics enterprises, poor food quality due to unsound regulatory system, etc., and discusses and concludes the high-quality development path of cold chain agricultural products distribution under the psychology of consumers.

**Subjects and Methods:** In order to deeply explore the influence of consumers’ psychological needs on cold chain agricultural products distribution, this paper is based on AHP (hierarchical analysis) and grey correlation model, combined with the knowledge of psychology and behavioural science, to analyse the decision-making behaviours of cold chain distribution enterprises. By identifying consumers’ emotional fluctuations, pressure feelings, and trust changes when purchasing cold-chain agricultural products, it examines how enterprises can optimise their cold-chain distribution services according to these psychological needs.

**Results:** Through discussion and investigation, this paper provides a path for the future development of cold chain agricultural enterprises that better meets the various psychological needs of consumers and their requirements for logistics quality.

**Conclusions:** This paper combines psychological needs and emotional behavioural changes to explore the cold chain agricultural products enterprises in the future in order to meet the psychological needs of consumers need chain logistics enterprises need to take measures to improve the level of technology, strengthen the infrastructure construction, optimize the quality of service, improve the construction of the regulatory system, and promote the whole process of digital integration and other measures.

## PHHS25025: CLINICAL EFFICACY ANALYSIS OF VERICIGUAT IN MIDDLE-AGED AND ELDERLY PATIENTS WITH HFREF CAUSED BY ISCHEMIC CARDIOMYOPATHY

Zhijie Song^a,b^, Jia Liu^c^, Zhihui Yan^d^, Nannan Leng^e^, Lihua Cao^a^, Wenzhong Zhang^a^

^*a*^ *Department of Cardiology, Affiliated Hospital of Qingdao University, Shandong, China,* ^*b*^ *Department of Electrocardiogram, Yiyuan County People’s Hospital, Shandong, China,* ^*c*^ *Department of Cardiology, Laizhou People’s Hospital, Shandong, China,*^*d*^ *Department of Cardiology, Qidu Hospital (Linzi District Maternal and Child Health Hospital) of Zibo City, Shandong, China,*^*e*^ *Department of Cardiology, West Coast New Area People’s Hospital of Qingdao, Shandong, China.*

*ZS and JL contributed equally to this study.*

**Background:** The primary goal of the research was to assess the efficacy of vericygide in conjunction with GDMT in managing HFrEF among middle-aged and elderly patients.

**Subjects and Methods:** A total of 386 HFrEF patients diagnosed at Qingdao University Affiliated Hospital were enrolled and stratified via a randomized method into an observation group (GDMT + vericiguat, n = 193) and a control group (GDMT alone, n = 193). The observation group was further categorized by etiology (ischemic vs. non-ischemic cardiomyopathy) and revascularization status. Complete revascularization subgroups were identified in both the observation and control groups. Efficacy parameters, including indices of cardiac structure and function such as N-terminal pro-B-type natriuretic peptide (NT-proBNP), left ventricular ejection fraction (LVEF), left ventricular end-diastolic diameter (LVDD), and left ventricular end-systolic diameter (LVDS), were assessed at 3- and 6-month follow-ups. Major adverse cardiovascular events (MACE) were recorded as a safety endpoint.

**Results:** At both 3- and 6-month follow-ups, the observation group demonstrated significantly greater improvements in NT-proBNP levels, LVEF, LVDD, and LVDS compared to the control group (all P < 0.05). Ischemic cardiomyopathy patients in the observation group showed more pronounced improvements than non-ischemic counterparts. Among patients with complete revascularization, the observation group exhibited superior cardiac structural and functional recovery. Moreover, as the drug exposure time prolongs, the therapeutic effect shows a trend of gradually improving over time. Additionally, the incidence of MACE was significantly lower in the observation group.

**Conclusions:** The addition of vericiguat to GDMT significantly amplifies therapeutic efficacy in HFrEF, particularly in ischemic cardiomyopathy. The differential benefit observed in ischemic patients may stem from vericiguat’s capacity to ameliorate microvascular dysfunction—a common residual pathology even after revascularization—through its action on the nitric oxide-soluble guanylate cyclase pathway. By enhancing cyclic guanosine monophosphate signaling, vericiguat likely promotes vasodilation, reduces myocardial fibrosis, and attenuates ventricular stiffness, mechanisms that align with the observed improvements in cardiac biomarkers and remodeling. These findings position vericiguat as a valuable adjunct for ischemic HFrEF, offering a targeted strategy to address microcirculatory deficits that conventional therapies often fail to modify. Future studies should explore long-term outcomes and validate these benefits in broader populations, including those with advanced microvascular disease.

## PHHS25026: THE SUSTAINABLE IMPACT OF BAODING CLIMATE ON MENTAL HEALTH

Lijun Wang ^a^

^*a*^ *School of Geography and Tourism, Baoding University, Baoding 071000, Hebei, China.*

**Background:** Climate plays a pivotal role in shaping our mental well-being. The fluctuations in temperature, humidity, and other atmospheric conditions can significantly influence our mood, stress levels, and overall psychological state.

**Subjects and Methods:** The study reveals several significant findings regarding the impact of climate on mental health. Firstly, extreme weather events such as floods, hurricanes, and droughts have been linked to increased levels of psychological stress and anxiety. These events often lead to displacement, loss of property, and disrupted social networks, which can have profound effects on mental health.

**Results:** This study draws the following conclusions: First, the reasons for the formation of two different climates in the eastern and western regions of Baoding City are mainly affected by the two major factors of terrain, altitude and water body. Second, the altitude of the western region is significantly higher than that in the east, and the wetland area in the eastern region is large. Third, through data comparison, it is concluded that the number of windy days in the west is significantly more than that in the east, and the average winter temperature in the west is generally lower than that in the east, which also has an impact on mental state.

**Conclusion:** It is important to note that while the impact of climate on mental health is significant, there are coping strategies that can help mitigate these effects. These include maintaining a healthy lifestyle with regular exercise, a balanced diet, and adequate sleep. Seeking social support through community engagement and staying connected with friends and family can also provide a sense of comfort and belongingness. Additionally, practices like mindfulness and meditation can help individuals stay calm and resilient during stressful times.

**Acknowledgements**

This research was supported by the Programme: JG202019, Baoding University. I offer my sincerest gratitude to all those who have been a part of my research journey.

## PHHS25027: CLINICAL ANALYSIS OF 31 CASES OF GENITAL TRACT MALFORMATION COMPLICATED WITH ENDOMETRIOSIS

Yanhua Huo^a^, Shuxian Li^a^, Juan Wang^a^

^*a*^ *Department of Gynecology, Shanxi Children’s Hospital (Maternal and Child Health Hospital), Taiyuan 030006, Shanxi, China.*

**Background:** The study summarized the reproductive tract abnormalities concurrent endometriosis clinical data, investigate the association between the genital tract anomalies.

**Subjects and Methods:** From October 2013 to October 2023, a total of 215 cases of genital tract anomalies undergoing treatment in the Department of Gynecology, Shanxi Maternal and Child Health Hospital were studied retrospectively, of which 31 cases coexisting with endometriosis, and all patients were diagnosed by surgery and pathology.

**Results:** (1) The proportion of endometriosis in genital tract anomalies, 215 cases of genital tract anomalies patients, of which 31 cases with endometriosis.32 cases of obstructive genita tract anomalies in patients, where 10 cases with endometriosis accounted for: 10/32, 183 cases of non-obstructive genita tract anomalies in patients, including 21 cases with endometriosis accounted for 21/183, which reached statistical difference(P<0.05). (2) The mean age were 26 years (15-39 years old) in obstructive genita tract anomalies coexisting with endometriosis patients, the mean age were 37 years (24-54 years old) in non-obstructive genital tract anomalies coexisting with endometriosis patients, the difference was statistically significant(P<0.05). The major symptoms were dysmenorrheal or chronicpelvic pain (7/10) and primary amenorrhea (2/10) in obstructive genita tract anomalies coexisting with endometriosis, However, in non-obstructive genital tract anomalies coexisting with endometriosis the major symptoms were infertility (9/21), dysmenorrheal or chronic pelvic pain (8/21) and abortion (5/21). (3) Obstructive genita tract anomalies mild endometriosis was seven cases (7/10), moderate or severe endometriosis was three cases (3/10), in non—obstructive genita tract anomalies, mild endometriosis was nine cases (9/21), moderate or severe endometriosis was twelve cases (12/21), which did not show statistical difference(P>0.05). (4) Type of genita tract anomalies coexisting with endometriosis: peritoneal endometriosis (obstructive genital tract anomalies7/10, non-obstructive genital tract anomalies 14/21), ovarianl endometriosis (obstructive genital tract anomalies 3/10, non-obstructive genital tract anomalies9/21), The proportion of peritoneal endometriosis, ovarian endometriosis did not show significant difference between two groups(P>0.05). (5) The proportion of endometriosis coexist with duplex uterus, uterus bicornis and uterus septus, respectively (2/8, 1/2, 16/116), there were no statistical difference (P>0.05).

**Conclusions:** (1) The proportion of endometriosis in obstructive genital tract anomalies compare with the proportion of endometriosis in non-obstructive genital tract anomalies increased. (2) The patients of obstructive genital tract anomalies coexisting with endometriosis are young, the major symptoms were dysmenorrheal or chronic pelvic pain and primary amenorrhea. The patients of non-obstructive genital tract anomalies coexisting with endometriosis are childbearing age, the major symptoms were infertility, dysmenorrheal or chronic pelvic pain and abortion. (3) The degree of endometriosis coexisting with obstructive genital tract anomalies and non-obstructive genital tract anomalies is no significant difference. (4) The type of endometriosis coexisting with obstructive genital tract anomalies and non-obstructive genital tract anomalies is no significant difference. In different types of non-obstructive genital tract anomalies, the proportion of duplex uterus, uterus bicornis and uterus Septus coexisting with endometriosis was not significant difference.

**Acknowledgments**

This work was supported by a project grant from Project of Shanxi Provincial Department of Health (Grant No. 2024ZYY2C096).

## PHHS25028: RESEARCH ON THE PRACTICE PATH OF INTEGRATION BETWEEN INDUSTRY AND EDUCATION OF INFORMATION COLLECTION AND WRITING FROM THE PERSPECTIVE OF PSYCHOLOGICAL NEEDS

Jingjing Cao ^a^

^*a*^ *School of Culture and Media, Xi’an Eurasia University, Xi’an, Shaanxi, China.*

**Background:** With the rapid development and continuous integration of AI writing and other AI technologies, the content production of the media industry is undergoing great changes. In this background, the integration between industry and education has become one of the important paths for universities to deal with the challenges and innovations of the media industry landscape. Although the integration between industry and education has achieved initial results, there are still difficulties in promoting school-enterprise cooperation. This uncertainty is easy to bring psychological pressure to all parties involved and affect mental health. College teachers may be anxious because of teaching adjustment, enterprises are worried about the results and benefits of cooperation, and students are confused about the future. All these need to pay attention to the psychological needs and mental health problems of all parties in the integration of industry and education.

**Subjects and Methods:** In this article, the author designs interview questionnaire, and attempts to analyze the similarities and contradictions between the demands and expectations of the psychological needs of teachers, students, and enterprises in the process of school-enterprise cooperation through interviews, thereby providing a reference path for achieving efficient integration between industry and education.

**Results:** First, in the practice of integration between industry and education, teachers’ psychological needs have been partially satisfied. However, due to increased workloads, an imperfect evaluation system, and an unsatisfactory teaching staff, teachers’ enthusiasm has significantly diminished. The author believes that it is necessary to promptly optimize the curriculum management system, talent introduction policies, and teacher evaluation systems to enhance teacher enthusiasm.

Second, the difference of the core objectives between enterprises and schools and the investment costs in practice that often fail to yield the expected returns lead to an increased perception of psychological distance. The author believes that this requires schools and enterprises to seek commonly recognized values, pursue shared interests, and achieve mutual satisfaction of psychological needs in a virtuous cycle of interaction.

Finally, students’ psychological needs in practice are generally being met. However, there remains a problem that students’ interest does not persist long. The author believes that this requires schools and teachers to fully explore students’ positive qualities through designing diverse practical tasks and provide timely rewards, thereby enhancing students’ enthusiasm for practice.

**Conclusions:** As key participants in the integration of industry and education, the willingness and engagement of teachers, enterprises and students play a significant role in the effectiveness of this practice. By understanding the psychological needs of these three main groups and how they are being met, the author analyzes the issues arising during the integration process, aiming to provide a reference path for integration between industry and education.

**Acknowledgments**

This research is under the project of the 14th Five-Year Plan “Research on the Practice of Mixed Teaching Mode for News and Communication Courses in the Digital Era”, which is supported by Shaanxi Academy of Educational Sciences (Grant No. SGH21Y0388), and the research project on higher education teaching reform “Research and Practice on Teaching Reform of Industry Education Integration Courses in Applied Universities: Taking Information Collection and Writing Courses as an Example”, which is supported by Xi’an Eurasia University (Grant No. 2023YB009).

## PHHS25029: ANALYZING THE LITERARY WORKS OF CHEN XU, A REPRESENTATIVE WRITER OF THE MANDARIN DUCKS AND BUTTERFLIES SCHOOL: FROM A MENTAL HEALTH PERSPECTIVE

Yanping Li^a^, Yao Jin^a^

^*a*^ *Jiaxing Nanhu University, Jiaxing 314001, Zhejiang, China.*

**Background:** The literary works of Chen Xu, a representative writer of the Mandarin Ducks and Butterflies School in modern Chinese literature, deeply reflect the social features and psychological state of the people during a specific historical period. The purpose of this study is to analyze Chen Xu’s works from the perspective of mental health, to experience the rich emotional world and complex psychological state contained in his works, to more comprehensively understand the inner world of people in that era, and to explore the potential impact of literary works on public mental health.

**Subjects and Methods:** Using Chen Xu’s literary works as the research object, this study employs close textual reading and emotional analysis to investigate the emotional expressions and psychological depictions in his works. By comparing themes, emotional types, and modes of expression across different works, the study examines their potential positive impacts on readers’ mental health.

**Results:** The findings reveal that Chen Xu’s works display a rich spectrum of human emotions and psychological needs, which remain relevant in modern society. Through delicate emotional portrayals, his works provide readers with a space for emotional resonance, helping them gain psychological support and solace when facing stress and challenges. Furthermore, the themes of emotional connection, familial bonds, and friendship presented in his works offer positive insights into maintaining mental health.

**Conclusions:** Chen Xu’s literary works not only hold literary value but also provide valuable references for modern mental health research. Literature, as a “poetic dwelling place” for the human soul, has a therapeutic function. The emotional wisdom and psychological depictions embedded in his works remind us of the importance of addressing emotional needs and valuing interpersonal relationships in promoting mental health. Future research could further explore the practical application and value of literary works in promoting mental health. Finally, we also hope that more scholars and researchers will conduct in-depth interpretation and research on Chen Xu’s works from different perspectives and find more mental health elements and hidden insights.

**Acknowledgments**

This research was supported by the Jiaxing Nanhu University 2023 Student Research Training (SRT) Program Project: “Development and Utilization of the Literary Value of *Mandarin Ducks and Butterflies School*—A Case Study of Chen Xu’s Literary Works.”

## PHHS25030: MULTILINGUAL NEWS CLUSTERING BASED ON CROSS-LANGUAGE WORD EMBEDDING FOR MENTAL HEALTH TREND MONITORING

Haocheng Huang^a^, Yuhao Long^b^, Lin Wu^b^, Ziyv Fan^b^

^*a*^ *State Key Laboratory of Media Convergence and Communication, Communication University of China, Beijing, China,* ^*b*^ *School of Computer and Cyberspace Security, Communication University of China, Beijing 100024, China.*

**Background:** Today, with the full development of the Internet and self-media, daily news emerges in an endless stream, and the difficulty of supervision is increasing. Although news gathering is not a problem, in the face of massive news, how to classify and process it is a difficult problem, especially for non-English foreign languages. If completely dependent on manual efforts, some languages have less talent reserves and low efficiency. This paper focuses on cross-language news clustering, proposing a solution for the bilingual clustering scenario of English and French, with applications in monitoring and analyzing global mental health trends.

**Subjects and Methods:** This paper proposes a new approach for cross-language news representation by using sentence vector embeddings in a mixed semantic space, combined with the topic probability distribution of news content. Knowledge distillation techniques are employed to align two semantic spaces. To enhance news clustering efficiency, the paper replaces traditional static methods like K-Means and AGNES with an improved SinglePass incremental clustering algorithm, making it more suitable for cross-language news. This approach is particularly valuable for monitoring global mental health trends, emotional behavior, and mood disorders, as it enables dynamic analysis of news across different languages and cultures. By clustering news based on psychological context and emotional tone, the system can detect emerging psychological needs, identify regions at risk of mental health issues, and provide targeted support for global mental health intervention.

**Results:** This study proposes a cross-language model trained via knowledge distillation, utilizing the English BERT as the teacher model and XLM-Roberta as the student model. The model effectively represents bilingual texts in English and French, making it particularly useful for monitoring global emotional trends, including mood disorders and psychological well-being shifts. By integrating the LDA topic model, the news content is represented through cross-language vectors of headlines and topic probability distributions, addressing the challenge of cross-language content representation and facilitating the monitoring of mood disorders, emotional labor, and the psychological impacts of global events. Furthermore, the SinglePass clustering algorithm is enhanced to better accommodate news clustering tasks, incorporating adjustments to the sample-cluster distance, cluster merging operations, and the inclusion of temporal parameters. These optimizations improve the system’s adaptability and efficiency, particularly in tracking emotional behavior changes, work-related stress, and mood-modulating trends across multiple languages and regions. This approach provides a robust framework for analyzing and addressing emerging psychological and emotional needs on a global scale.

**Conclusions:** In the experiment, evaluation metrics such as accuracy, regression rate, F1 score, and Kappa coefficient were used to verify that the proposed scheme can effectively address the cross-language news clustering problem in English and French. The accuracy reached 75.6% for coarse-grained topic-level news clustering and 75% for fine-grained event-level clustering, with a Kappa coefficient of 0.7069. This demonstrates the model’s capability to effectively monitor changes in emotional behavior and psychological needs in news content related to mood disorders, work stress, and other mental health issues.

## PHHS25031: THE IMPACT OF INTEGRATING INTANGIBLE CULTURAL HERITAGE INTO UNIVERSITY DIGITAL PUBLISHING PROGRAMS ON STUDENTS’ PSYCHOLOGICAL WELL-BEING

Lihong Liu^a^, Chatupol Yongson^b^, Li Ding^a^

^*a*^ *Xi’an Eurasia University, Xi’an, Shaanxi, China,* ^*b*^ *Department of Educational Administration and Higher Education, Faculty of Education, Srinakharinwirot University, Bangkok 10110, Thailand.*

**Background:** The rapidly evolving social environment and increasingly fierce social competition have given rise to mental health issues such as anxiety and depression among college students. The learning environment of college students profoundly influences their mental health. How professional programs can effectively take advantage of the nurturing power of culture to promote the psychological well-being of college students is a topic worthy of exploration.

**Subjects and Methods:** To investigate the impact of integrating intangible cultural heritage (ICH) into digital publishing programs on students’ mental health, this study proposes a theoretical hypothesis based on new constructivism. Utilizing a mixed-methods approach that combines quantitative and qualitative research, this study focuses on surveying the talent development programs of digital publishing majors in seven local universities in China, as well as the attitudes and perspectives of faculty and students towards the integration of ICH into professional development.

**Results:** Through the research and analysis, four pathways for integrating ICH into digital publishing programs are proposed. These pathways specifically include the following four aspects: seeking government and enterprise cooperation, cultivating dual-qualified ICH teachers, integrating ICH into the curriculum, and incorporating ICH into scientific research. In addition, the study places a strong emphasis on the positive impact of ICH education on the mental health of college students. The results indicate that ICH education effectively enhances students’ cultural identification, sense of learning achievement, and overall happiness, thereby fundamentally promoting their psychological well-being.

**Conclusions:** Both faculty and corporate stakeholders affirm the necessity of integrating ICH into digital publishing programs. More than half of the students reported a noticeable increase in their cultural and psychological sense of belonging to ICH, and they actively participate in ICH-related projects. During the learning process, students experience positive teacher-student relationships and derive pleasure and satisfaction from their studies. The findings of this research offer valuable insights and references for local universities in China on how to influence students’ mental health through the development of digital publishing programs.

**Acknowledgments**

This article is a phased achievement of the “2023 Shaanxi Province Higher Education Teaching Reform Research Key Project ‘AIGC Background Digital Publishing Innovation Compound Talent Cultivation Reform and Exploration’” (23BZ080) and “Research on the Design of Shaanxi Intangible Cultural Heritage Database Platform for Arts and Crafts” (Project Approval Number: 2023J018).

## PHHS25032: THE PSYCHOLOGY OF AI IN EDUCATION: EMPOWERING VOCATIONAL LEARNERS WHILE ADDRESSING DEPENDENCE

Liliang Qi ^a^

^*a*^ *Xinyang International Vocational and Technical College, Xinyang, Henan 465500, China.*

**Background:** In order to improve the application of artificial intelligence tools in education and bring great challenges to vocational education reform, the deep application of artificial intelligence in teaching has transformed the way people utilize educational resources and changed the cognitive state of learners’ psychology.

**Subjects and Methods:** Analyze the role of artificial intelligence in promoting vocational education from the perspective of educational availability, and analyze the characteristics, functions, and types of intelligent tools to construct an intelligent learning space that includes intelligent interaction modules, feedback modules, learning processes, and learning analysis modules. By studying the supply of resources, the interactivity of intelligent platforms, the integration of virtual and real spaces, immersive services, and the psychological changes of learners, combined with the application of artificial intelligence in learners, and through data analysis, implementation strategies for the reconstruction of artificial intelligence and vocational education space are proposed.

**Results:** The application of artificial intelligence tools in vocational education has restructured the learning space, enabling learners to access resources from multiple sources, transforming their learning concepts, improving their abilities, and more effectively supporting vocational education reform. At the same time, it also emphasizes reducing learners’ psychological dependence on learning space reconstruction.

**Conclusions:** With the continuous development of artificial intelligence iteration technology, the connection between virtual and real spaces has become more multidimensional, and learners can utilize more diverse resources and receive vocational skills training in more diverse and convenient ways, enabling learners to psychologically recognize the role of artificial intelligence. Exploring the difficulties and strategies of empowering vocational education curriculum teaching with artificial intelligence is of great practical significance for promoting vocational education reform.

## PHHS25033: DISCUSSION ON THE VALUE RATIONALITY OF THE HIGH-QUALITY DEVELOPMENT OF INDUSTRIAL COLLEGES IN HIGHER VOCATIONAL COLLEGES FROM THE PERSPECTIVE OF MENTAL HEALTH

Shu Zhou^a^, Yinggen He^b,c^, Kang Liu^b,d^, Ronghui Wang^e^

^*a*^ *Chongqing College of International Business and Economics, Chongqing 401520, China,* ^*b*^ *Faculty of Education, Southwest University, Chongqing 400715, China,* ^*c*^ *College of Vocational Teacher Education, Chongqing Normal University, Chongqing 430331, China,* ^*d*^ *Academic Affairs Office, Chongqing Wuyi Vocational and Technical College, Chongqing 400715, China,* ^*e*^ *Scientific Research Department, Chongqing Vocational University of Electronic Science and Technology, Chongqing 430331, China.*

**Background:** In China, the industrial colleges jointly established by higher vocational colleges and industry enterprises are in a period of vigorous development. However, from the perspective of students’ mental health, their development shows a tendency of pragmatism thinking with the output of achievements as the core, the breakthrough of technical indicators as the key, and the economic indicators as the fundamental support, lacking humanistic care and having the risk of deviating from the fundamental goal of talent cultivation.

**Research Objects and Methods:** This study takes the industrial colleges in higher vocational colleges as the research objects and comprehensively uses methods such as literature research, field investigation, questionnaire survey and on-site interview. Starting from the perspective of the mental health of students in industrial colleges, it aims to comprehensively analyze the development status and problems of industrial colleges and then explore the value rationality of their high-quality development.

**Results:** The study finds that there are four misunderstandings in the current industrial colleges in higher vocational colleges in terms of talent cultivation: instrumentalism with priority given to interests, market orientation with priority given to benefits, survivalism with priority given to quantity, and pragmatism with technology supremacy. Analyzed from the mental health level, these misunderstandings are respectively manifested as one-sided understanding of the talent cultivation goal, leading to overly utilitarian talent cultivation, neglecting the quality of talent cultivation, and restricting the all-round development of students.

**Conclusion:** Industrial colleges should, from the perspective of students’ mental health and based on the value rationality requirements of talent cultivation in four dimensions: moral character, all-round development, comprehensive quality and scientific spirit, calibrate the value rationality of talent cultivation with the standards of four dimensions: high quality, people-centered, fairness and justice, and all-round development, establish correct concepts, pay attention to the all-round development and individual differences of students, and cultivate high-quality talents.

## PHHS25034: HOW TECHNOSTRESS AFFECTS JOB PERFORMANCE: AN EMPIRICAL STUDY BASED ON EGO-DEPLETION THEORY

Zhongze Guo^a^, Mengmeng Xu^a^, Jiwen Chen^a^, Hao Dong^a^

^*a*^ *Beijing Information Science and Technology University, Beijing 100192, China.*

**Background:** In the era of technological self-reliance and self-improvement, the continuous iteration and upgrading of Information and Communication Technologies (ICTs) have imposed significant technostress on employees. Technostress often manifests as anxiety and discomfort associated with technology use. Although some research has begun to address the issue of technostress, there is still a lack of in-depth exploration regarding its effects on job performance within the specific context of the communications industry. The communications industry stands at the forefront of technological change and is significantly impacted by technostress, making the understanding of this relationship quite representative. This study aims to fill this research gap by investigating how technostress influences job performance of employees through psychological factors such as dedication and absorption.

**Subjects and Methods:** Based on ego-depletion theory, this research highlights the importance of limited psychological resources in determining job performance. Using the Credamo platform, we generated a questionnaire link and randomly selected 415 full-time employees from three major communications companies in China as our research sample. We constructed a moderated mediation model to analyze the mediating roles of dedication and absorption in the relationship between technostress and job performance, as well as the moderating role of resilience. The model was empirically tested using confirmatory factor analysis, hierarchical regression analysis, and the bootstrap method.

**Results:** The findings found that (1) dedication and absorption are important mediating variables between technostress and job performance. Specifically, higher levels of technostress were associated with decreased dedication and absorption, which in turn negatively affected job performance; (2) resilience plays a moderating role in the relationship between technostress, dedication, and absorption. Employees with higher resilience can mitigate the negative effects of technostress on dedication and absorption; (3) employees with higher resilience exhibited higher job performance by mitigating the negative correlation between technostress and the mediating variables of dedication and absorption.

**Conclusions:** The findings shed intrinsic mechanism of action and boundary conditions of technostress affecting job performance of employees, offering valuable insights for future research. The findings provide empirical evidence for the academic community and practical implications for management. Organizations can enhance job performance by fostering employee resilience, creating supportive work environments, and promoting dedication and absorption.

**Acknowledgements**

This research was funded by the National Natural Science Foundation of China Project (Grant No. 72002017, 72002035, 72402232), Humanities and Social Science Fund of Ministry of Education (Grant No. 24YJC630063), the Key Project of Beijing Municipal Education Commission (Grant No. 22JDGLB032), and the Education Reform Project of Beijing Information Science and Technology University (Grant No. 2024JGSZ17).

## PHHS25035: ANALYSIS AND INVESTIGATION PATH OF CRYPTIC LANGUAGE IN CYBERCRIME FROM THE PERSPECTIVE OF MENTAL HEALTH

Wenbo Fu^a^, Zhihui Ban^a^

^*a*^ *The National Police University for Criminal Justice, Baoding 071000, China.*

**Background:** The escalating prevalence of cybercrime has emerged as a significant global threat, with cryptic language in cybercrime serving as a covert communication tool for perpetrators, further complicating investigative efforts. To effectively address this challenge, this study aims to delve into the characteristics of cryptic language in cybercrime and its correlation with the psychological profiles of the offenders from the perspective of mental health. By uncovering the deep-seated psychological motivations behind the use of cryptic language, this study seeks to provide new investigative insights for law enforcement agencies, aiming to enhance the efficiency of cybercrime investigations through theoretical support and practical guidance.

**Subjects and Methods:** The primary focus of this study is the cryptic language in cybercrime and its users, namely the offending entities. Employing methods such as psychological analysis and status quo examination, this study conducts an in-depth analysis of the cryptic language in cybercrime. Firstly, through extensive collection and analysis of cryptic language usage in cybercrime cases, the study summarizes the trends towards diversification, complexity, and concealment. Secondly, integrating psychological theories, the study deeply analyzes the psychological motivations behind the use of cryptic language by offending entities, exploring their underlying mental health conditions and psychological characteristics. Finally, based on the aforementioned analysis, targeted investigative strategies and recommendations are proposed to improve the efficiency of cybercrime investigations.

**Results:** The findings indicate a close correlation between the use of cryptic language in cybercrime and the mental health conditions and psychological characteristics of offending entities. Offenders often use cryptic language to evade detection, enhance their sense of belonging, and showcase their criminal skills and experiences. These cryptic languages not only possess anti-investigative functions but also reflect mental health issues such as self-confidence, risk-taking, and antisocial tendencies among offending entities. Furthermore, the study reveals differences in the use of cryptic language among offending entities involved in different types of crimes, reflecting their distinct mental health conditions and psychological characteristics to some extent, and providing valuable clues and evidence for law enforcement agencies.

**Conclusions:** In summary, the use of cryptic language in cybercrime is an indication of the mental health issues of offending entities, posing new challenges for investigative work. To effectively combat cybercrime, law enforcement agencies should strengthen their research and analysis of cryptic language in cybercrime, deeply analyze the mental health conditions and psychological characteristics of offending entities, and formulate targeted investigative strategies. Additionally, cooperation should be enhanced to jointly combat cybercrime, safeguarding cybersecurity and social stability. Furthermore, sufficient attention and intervention should be given to the mental health issues of offending entities to reduce the occurrence of cybercrime.

**Acknowledgments**

This research was supported by the 2021 Annual Central Judicial Police Academy School of Prison Studies “Criminal Language Investigation” Young Teaching Research Team (Project No.: JYXQNJYTD202104) Phase Achievements.

## PHHS25036: ANALYSIS OF PRAGMATIC PHENOMENA OF BANTER FROM THE PERSPECTIVE OF MENTAL HEALTH

Guizhi Zhang^a^, Zhanyu Cui^a^

^*a*^ *School of Foreign Languages, Henan University of Technology, Zhengzhou 450001, Henan, China.*

**Background:** With the proliferation of online communication, the presence of banter in online contexts has increasingly drawn attention. Bantering has some effects on human’s mental health. Analyzing it from the perspective of mental health is helpful to understand the role and value of bantering better in interpersonal interactions, as well as the potential risks it may bring. This paper takes TikTok comments as a case study to investigate the pragmatic phenomena of banter from the perspective of mental health.

**Subjects and Methods:** This research takes the comments from TikTok popular videos as the research subjects, applies the qualitative research method approach to collect and analyse data with the discourse analysis method. The focus is placed on analyzing banter discourse from the perspective of false politeness to explore the pragmatics of banter within the framework of false politeness.

**Results:** Banter in these comments is categorized into positive and negative aspects. The former includes non-strategic, agreement-oriented, magnanimous, and praising banters, while the latter includes strategic, stingy, oppositional, sympathetic, and derogatory banters. There are two common response styles of banter: aggressive and defensive responses. These two styles have different purposes and effects. It reveals that malicious banter serves to construct the identity of the ironist by belittling others and asserting their own superiority. Moreover, adjusting the degree of linguistic proximity acts as a self-protective mechanism, allowing individuals to utilize banter as a means to safeguard their image and interests by bantering others.

**Conclusions:** This study explores the pragmatic phenomena and strategies of banter within the framework of deceptive impoliteness, taking TikTok short video comments as a case study. Banter serves not only as a tool for group entertainment but also as a means to facilitate harmonious interaction, playing a significant role in social life. The findings shed light on the category and application of banter within the context of deceptive impoliteness and delve into the motivations underlying its usage. This research intends to enrich readers’ understanding of banter pragmatics in online contexts and expand the scope of banter research.

**Acknowledgments**

This work was supported by a project grant from Department of Social Sciences, Ministry of Education (No. 24YJA740009).

## PHHS25037: ANALYSIS OF ONLINE REVIEWS BASED ON THE PSYCHOLOGICAL NEEDS OF SPORTING GOODS CONSUMERS-TAKING LI NING BASKETBALL SHOES ON JINGDONG MALL AS AN EXAMPLE

Hanqing Hu^a^, Yuesong Tian^a^, Qiong He^a^, Yuanyuan Jin^b^, Yi Sun^a^

^*a*^ *Beijing Information Science and Technology University, Beijing 100096, China.* ^*b*^ *Beijing Information Technology College, Beijing 100015, China.*

**Background:** This study analyzes online reviews of Li Ning basketball shoes to gain insights into consumer concerns and emotional tendencies. By combining these insights with consumers’ intrinsic needs, we aim to provide targeted data support and strategic recommendations for the product research and development of Li Ning basketball shoes, as well as for understanding market trends. The goal is to better address the psychological needs of consumers and enhance product-market adaptability.

**Subjects and Methods:** We employed web scraping technology to collect online review data of Li Ning basketball shoes from Jingdong Mall. After preprocessing the data—such as removing stop words, tokenization, and lemmatization—we utilized the TF-IDF algorithm for feature extraction and the LDA topic model for feature analysis. Finally, we conducted sentiment analysis using the BiLSTM model to assess consumer satisfaction and dissatisfaction, analyze the underlying issues, and explore the potential impact of consumer psychology on purchasing decisions and brand loyalty.

**Results:** The study revealed that consumers’ primary concerns regarding Li Ning basketball shoes encompass several dimensions: product experience, logistics service, shopping experience, perceived shoe quality, and performance technology. Notably, product experience emerged as the most significant dimension, particularly in terms of design aesthetics and comfort, which are closely linked to consumers’ psychological needs for a healthy and enjoyable sports experience. Additionally, efficient and convenient logistics services significantly influence consumers’ purchasing decisions, aligning with their psychological demand for efficiency in daily life.

**Conclusions:** This study comprehensively applies online review text mining techniques, using Li Ning basketball shoes as a case study to elucidate consumer demand themes in the context of online consumption. By integrating these themes with consumers’ psychological health needs, we provide an empirical foundation for optimizing product design and market strategy. In product design, it is crucial to prioritize consumers’ psychological needs for health by focusing on comfort and aesthetic design to fulfill their desires for a healthy and enjoyable sports experience. Furthermore, optimizing logistics processes and other aspects can enhance efficiency, improve the overall shopping experience, and promote consumers’ psychological well-being, thereby increasing the market competitiveness of the product.

**Acknowledgements**

This work was supported by a project grant from Promoting the Development of Universities and Colleges-Base Construction-Innovative Practice Base Construction of School of Management Science and Engineering, Beijing University of Information Science and Technology. (Grant No.5112410821).

## PHHS25038: A NOVEL POTASSIUM CHANNEL ACTIVATOR ON KCNQ CHANNELS

Qian Zhao^a^, Yihuang Liu^b^, Pingping Chen^a,b,c^, Shunxin Fan^a^

^*a*^ *Pharmacy, Hospital of Hebei University of Chinese Medicine, Shijiazhuang 050011, China,* ^*b*^ *Hebei University of Chinese Medicine, Shijiazhuang 050200, China,* ^*c*^ *Hebei International Cooperation Center for Ion Channel Function and Innovative Traditional Chinese Medicine, Shijiazhuang 050200, China.*

**Background:** By means of whole cell patch clamp system, to record KCNQ(Kv) and M currents in HEK293B cells as expression systems and DRG neurons as primary culture cells By means of currents clamp technology, to observe the effect of compound C15 on the action potential and analysis the excitability changes.

**Subjects and Methods:** (1) Channel expression: cDNA coding Kv7.2, Kv7.3 were inserted into the pcDNA 3.0 plasmid vector, cDNA coding GFP was inserted into pEGFP-N1 plasmid vector and transformed in TOP10 competent germ. After extract and amplification, plasmids were detected with agarose gel electrophoresis and ultraviolet light spectrophotometer. The Kv7.2, Kv7.3 and GFP plasmids were co-expressed in HEK293 cells, to observe the green fluorescence cells with the help of fluorescence microscope which set up the motivation wave 450~490nm. (2) Potassium channel currents recording: with Axon 700B system, Kv currents was produced from HEK293 cells, co-transfected Kv7.2/7.3 plasmids and perforated whole-cell patch clamp by Amphotericin B. We also observed the regulation of compound C15 for M- currents in DRG neurons. (3) Observe the effect of C15 for neuronal M current in primary cultures of DRG neurons, by the whole-cell patch-clamp technique. (4) Recording membrane potential: using Axon 700B system, we recorded the action potential of DRG neurons by current clamp method, and observe the effects of C15 and XE991 on neurons excitability.

**Results:** Observations have revealed that C15 has a certain prolonging effect on the activation and deactivation kinetics of Kv7 channel subtypes, but it has a more pronounced impact on deactivation time constants compared to activation time constants. Specifically, it has the most significant effect on Kv7.1, Kv7.2, and Kv7.2/7.3 within the Kv7 channel family.

**Conclusions:** C15 has a regulatory effect on Kv7.1, Kv7.2, Kv7.4, Kv7.5, and Kv7.2/7.3 channels. Additionally, it was found that C15 enhances Kv7.2/7.3 channel current in a concentration-dependent manner, with an EC50 of 0.59±0.03 µmol. It shifts the I-V curve of Kv7.2/7.3 channels towards hyperpolarizing voltages, while also prolonging the activation and deactivation time constants, affecting channel kinetics characteristics. Compounds C15 as a new chemical compound had a potential activity to open Kv channels, discovered by high-throughput screening method. The project studied the detail, the mechanism and the characteristics about the new chemical compound C15, in regulation Kv channels. The aim is to provide some valuable experiment data for the next exploration.

**Acknowledgments**

This work was supported by a project grant from Scientific research project of Hebei Provincial Administration of Traditional Chinese Medicine (Grant No.2021067).

## PHHS25039: PHARMACOLOGICAL AND COMPUTATIONAL EVALUATION OF FO-SHOU-SAN FOR THERAPEUTIC POTENTIAL IN VASCULAR DEMENTIA BY REGULATING FERROPTOSIS

Jueyu Wang^a,b^, Xinqian He^b^, Shuzhen Lu^a^, Yiwen Sun^a^, Zelong Ma^a^, Deyi Wang^a^, Qi Wang^b^

^*a*^ *GuangDong Mechanical & Electrical Polytechnic, Guangzhou 510515, Guangdong, China,* ^*b*^ *Guangzhou University of Chinese Medicine, Guangzhou 510405, Guangdong, China.*

**Background:** To investigate the pharmacological mechanism of Fo-Shou-San (FSS), a traditional Chinese medicine (TCM) herbal formula against vascular dementia (VD), providing a reference for new drugs and clinical applications of traditional Chinese medicine prescriptions.

**Subjects and Methods:** Firstly, the main chemical constituents of Danggui (DG, Angelica sinensis, AS) and Chuanxiong (CX, Ligusticum wallichii, LW) in Fo-Shou-San were obtained through TCMSP database, and their corresponding targets were screened according to ADME. Secondly, the main pathological targets of vascular dementia were retrieved from GenCards, OMIM, TDD and DRUGBANK databases. Take the intersection between FSS components targets and vascular dementia disease targets. Thirdly, based on the common targets of the two, the protein interaction (PPI) network was constructed through protein interaction analysis on the STRING platform, and then potential protein functional modules were analyzed and attained via MCODE plug-in of CytoScape3.9.1. Metascape data platform was adopted to investigate the signaling pathways and biological processes of targets related to the FSS treatment against vascular dementia, and further CytoScape3.9.1 was utilized to build the drug ingredients-target-pathway network of FSS against VD, with molecular docking verification simulation via Autodock 4.2.6 software. Finally, the genes regulating ferroptosis were gained and scrutinized by searching FerrDb database, and the correlation among the FSS active components, vascular dementia disease and ferroptosis phenotype was further comprehensively analyzed and predicted, which experiments in vivo and in vitro verified the results.

**Results:** The main active components of FSS against VD were beta-sitosterol, stigmasterol, myricanone, etc. and the core targets of FSS in the treatment of VD as CASP3, CASP9, CASP8 and so on, with good molecular docking simulation results of components-targets and involved pathways including cancer, neurodegeneration-multiple diseases, etc. Finally, based on synthetic analysis between ferroptosis regulatory genes and intersection genes of FSS against VD, FSS active compounds could regulate ferroptosis thereby delaying the occurrence and progression of VD by mediating cancer, neurodegeneration-multiple diseases and other signaling pathways through 8 genes such as PTGS2, NOS2. The score results of molecular docking verification also showed that the docking result was satisfying. Furthermore, experimental researches showed that FSS could improve VD by regulating ferroptosis-related genes.

**Conclusions:** Multiple component-target-pathways of FSS against VD were discovered by means of network pharmacology. The overlap between the core targets and signaling pathways of FSS against VD, and the target protein networks and signaling pathways of FSS active components regulating ferroptosis, suggested that FSS played an important role in ameliorating VD by regulating ferroptosis, with experiments verification.

## PHHS25040: RESEARCH ON THE CHARACTERISTICS OF TRADITIONAL CHINESE INTERIOR SPACE DESIGN FROM THE PERSPECTIVE OF ENVIRONMENTAL PSYCHOLOGY

Lu Zhang^a^, Ke Li^b^

^*a*^ *Taiyuan Normal University, Department of Design, Jinzhong 030619, Shanxi, China,* ^*b*^ *Professor, School of Literature and Journalism, Shandong University, Shandong, China.*

**Background:** This study aims to explore the characteristics and methods of traditional Chinese interior space design from an environmental psychology perspective. It deeply investigates the impact of social and physical environments on aesthetic consensus and its external manifestations in interior space design. The study also examines the behavioral and psychological effects of these design characteristics on individuals. This research seeks to reveal the intrinsic design logic of traditional Chinese interior spaces, thereby providing new design ideas and innovative directions for contemporary residential design in the new Chinese style.

**Subjects and methods:** Based on environmental psychology theory, this study conducts spatial analysis of traditional Chinese paintings and documented interior spaces, considering the environmental and aesthetic consensus of the time. It summarizes the characteristics, causes, and emotional experiences of traditional Chinese interior space design. Through case studies of contemporary residential spaces, the study investigates the current state and design trends of modern residences and discusses modern applications of traditional Chinese interior space design characteristics.

**Results:** The study finds that interior spaces have psychological expression and suggestive functions. Traditional Chinese residential interior space design not only meets life and safety needs but also embodies the needs for love and belonging. Its unique design logic provides richer and deeper emotional experiences for users. This research not only provides theoretical support and innovative directions for new Chinese-style residential space design but also has interdisciplinary implications for art and psychology, contributing to further innovation and development in the field of modern interior design.

**Conclusions:** Through specific case analysis, it is proven that superficial Chinese element stacking cannot meet higher psychological needs of users. The design logic hidden beneath superficial features is the key to emotional resonance in space.

**Acknowledgments**

The research is supported by: Shanxi Province philosophy and social science planning topic (Project number: 2022YY140).

## PHHS25041: ANALYSIS OF ANTIVIRAL TREATMENT FOR HIV/AIDS AND ITS INFLUENCING FACTORS IN THE CONTEXT OF MEDICAL INSURANCE COVERAGE

Liujie Tang^a^, Shaoping Kang^a^

^*a*^ *School of Humanities and Management, Kunming Medical University, Kumming 650500, China.*

**Background:** To understand the medical insurance medication of HIV/AIDS antiviral therapy patients in a hospital and the influencing factors, and to provide scientific reference for other medical institutions to promote the medical insurance medication of HIV/AIDS antiviral therapy.

**Methods:** From the AIDS information System of the national health insurance system, information of HIV/AIDS patients currently being treated in a hospital was selected. SPSS 20.0 software was used to conduct χ2 test and logistic regression model to analyze the drug use and influencing factors of HIVAIDS antiviral therapy in medical insurance.

**Results:** In 2023, a hospital was treating 650 patients with HIV/AIDS, 53.54% of which were medicated by medical insurance. Multivariate logistic regression analysis showed that age was less than 20 years old (OR < 20 years old =0.32, 95%CI: 0.17-1.26) and 30 to 39 years old (R30-39 years old =0.66, 95%CI: 0.27 ~ 1.32), 40 ~ 49 years old (OR40 ~ 49 years old =0.44,95%CI: 0.16 ~ 1.22), cadre/worker/retired (OR=0.76,95%CI: 0.23 ~ 2.38), married with spouse (OR=0.27,95%CI: 0.08-0.82), college degree OR above (OR=0.73, 95%CI: 0.25-1.62), homosexual transmission (OR=0.82, 95%CI: 0.10-2.42), and baseline CD4+T lymphocytes less than or equal to 200/μL (OR=0.15, 95%CI: 0.06 ~ 1.12) is the positive factor of medical insurance drug treatment; Male, age over 50 years old, farmers, service industry, divorced or widowed, Han nationality, elementary school, middle and high school, heterosexual transmission, injecting drug use, baseline CD4+T lymphocytes greater than 351/μL, and the most recent VL value greater than 0 copies/ml were risk factors for medicated drugs.

**Conclusion:** The proportion of medical insurance drugs for antiviral treatment of HIV/AIDS in a hospital reached 53.54%. In mobilizing medical insurance drugs for antiviral treatment of HIV/AIDS, Attention should be paid to patients over 50 years old, divorced or widowed, primary, middle and high schools, heterosexual transmission, injecting drug use transmission, CD4+T lymphocytes greater than 351/mm3 and the most recent VL value greater than 0 copies/ml. It is necessary to strengthen the knowledge of HIV antiviral treatment and the education and publicity of medical insurance drugs. Provide the necessary social support system and psychological care, optimize the treatment plan, and strive to improve the effectiveness of AIDS antiviral treatment in the area, and improve the quality of life of HIV/AIDS.

**Acknowledgements**

This work was supported by a project grant from Philosophy and Social Sciences of Yunnan Province: Research on the Generation Mechanism and Prevention Path of Medical Financial Protection on Excessive Debt and Poverty Risk of micro-subjects (Grant No. YB2023016). The 2023 Undergraduate Education and Teaching Reform Research Project of Yunnan Province “Research and Practice on the Construction of a Simulation Teaching Platform for New Liberal Arts Mathematics and Intelligence under the Background of Medical and Cultural Integration” (Grant No. JG2023019), Medical Psychology and Health Management Team, Kunming Medical University (Grant No. 2024XKTDPY18).

## PHHS25042: PSYCHOLOGICAL DEPENDENCY AND ANXIETY OF EMPLOYEES: FROM THE PERSPECTIVE OF GOVERNMENT SUBSIDY

Liwei Chen ^a^

^*a*^ *College of Management, Fujian University of Technology, Fujian, China.*

**Background:** The government subsidies refer to the economic support provided by the government to specific enterprises for the purposes of encouraging the development of certain industries. In today’s complex and ever-changing commercial environment, companies are facing many challenges and uncertainties. In China, government subsidies play an important role in driving development of industrials. Previous studies have primarily examined the pros and cons of government subsidies from the perspective of financial performance. However, the relation between operational efficiency of corporate and the impact of government subsidies has never been definitively established. In light of limited government finances, the efficiency of subsidy policies of government deserves further study. This study focuses on the relationship between psychological dependence of managers on government subsidies, employee anxiety and risk tolerance in corporate operations, aiming to deeply analyze how these factors work together to affect the development strategy and performance of the company.

**Subjects and Methods:** I observe the industry of new-energy vehicles and take a different point of view on the government subsidies to explore whether the psychological dependence of the corporate managers and anxiety of employees can indeed have a negative impact on the green-energy industry. By observing 26 new-energy vehicle companies listed in China between 2013 and 2023 as samples, we exclude companies with special treatment (ST) and incomplete data. Finally, a total of 263 valid data were finally selected for this study. The OLS regression model is built to test the relationship between the psychological dependence of managers on government subsidies and anxiety of employees and its financial performance.

**Results:** There are three main results. First, as the proportion of government subsidies to a company’s total revenue increases, the greater the psychological dependence effect, and the lower the company’s operating efficiency. The government subsidies are not a master key to improving corporate operating performance. Second, the uncertainty about future career development caused by the probable cease of government subsidies may cause serious anxiety among employees. Anxiety not only affects employees’ mental health and job satisfaction, but may also have a negative impact on the company’s innovation ability and operating performance. Third, companies should improve their equity structure, reduce agency costs, and optimize asset allocation, rather than merely increase scale.

**Conclusions:** In the long run, the government subsidies may be regarded as unfair competition by other countries and lead to trade disputes among nations of the world. Enterprises should reduce their dependence on government subsidies, pay attention to mental health of employees, and create a good working atmosphere to ease anxiety of employees. The enterprises should evaluate and improve their risk tolerance according to their actual situation, formulate scientific and reasonable R&D expenditure strategies, and achieve sustainable development through continuous innovation.

## PHHS25043: RESEARCH ON THE RELATIONSHIP AMONG SELF-EFFICACY, FLOW EXPERIENCE AND LEARNING ENGAGEMENT OF COLLEGE STUDENTS IN ENGLISH LEARNING

Du Cheng ^a,b^

^*a*^ *Hunan Polytechnic of Environment and Biology, Hengyang, Hunan, China,* ^*b*^ *School of Education, Sehan University, Mokpo, Jeollanam-do, Republic of Korea.*

**Background:** Relative researches have proven that self-efficacy is an important prerequisite for flow experience, and the self-motivation mechanism of flow experience makes individuals unconsciously invest more time and energy. This study aims to investigate the effect and impact among self-efficacy, flow experience and learning engagement of college students in English learning, expanding the understanding of the relationship of these three factors and providing substantial theoretical and practical implications for educator and relevant researchers.

**Subjects and Methods:** This study used the questionnaire method to select 780 Chinese college students for testing the relationship and impact among self-efficacy, flow experience and learning engagement by using four scales: General Perceived Self-Efficacy Scale (GSES), Short Flow State Scale, Learning Motivation Scale, and College Learning Engagement Questionnaire. This study has adopted software of SPSS 26.0 and Amos 26.0 to analyze the results of the above scales and questionnaire to explore the relationship of self-efficacy, flow experience and learning engagement.

**Results:** It was found that self-efficacy had a positive impact on flow experience while flow experience mediated the relations between self-efficacy and learning engagement with statistical significance.

**Conclusions:** The higher the self-efficacy of college students when learning English, the more likely they are to experience flow, which in turn promotes the level of learning engagement, enhance the understanding of the relationship among self-efficacy, flow experience and learning engagement for educators and English teachers, and provide corresponding insights for future research on emotional and learning engagement interventions and strategies.

## PHHS25044: THE SUPPLY LEVEL, EFFECTIVENESS AND ACTION FRAMEWORK OF RURAL PUBLIC SERVICES IN CHINA FROM THE PERCEPTION OF FARMERS’ PSYCHOLOGICAL NEEDS

Qiqi Chen^a,b^, Jun Li^a^, Chuang Tian^a^

^*a*^ *Zhongyuan University of Technology, Zhengzhou, Henan, China,* ^*b*^ *System and Industrial Engineering Technology Research Center, Zhongyuan University of Technology, Zhengzhou, Henan, China.*

**Background:** The rural revitalization strategy is a major decision to build a well-off society in an all-round way, which will help promote rural economic development, enhance agricultural modernization and increase farmers’ income. This paper studied the factors, mode, level, effectiveness and action framework of rural public service supply under the background of rural revitalization based on the perspective of farmers’ psychological needs. We attempted to explore the level and satisfaction of rural public services from both supply and demand perspectives.

**Subjects and Methods:** The paper constructed an evaluation index system including six aspects, such as, medical and health service capabilities, social security service capabilities, cultural and educational service capabilities, ecological environment service capabilities, infrastructure service capabilities, and public safety service capabilities. We also calculated the public level of rural public enlistment based on the data of China’s National Statistical Yearbook. It quantified the specific effectiveness based on the questionnaire data from the perspective of farmers’ psychological needs to consider the satisfaction of farmers.

**Results:** From 2011 to 2020, the supply level of rural public services showed an overall upward trend with an average annual growth rate of 15.80%. The supply effectiveness of rural public services was above the medium level, and the satisfaction of farmers for different subcategories was different based on farmers’ psychological needs. The demand of the rural grassroots for medical and health services, social security services, infrastructure services, ecological and environmental services, cultural and educational services, public security services and other services accounted for 36%, 28%, 13%, 9%, 8%, 4% and 2% respectively.

**Conclusions:** The supply factors of rural public service were mainly composed of subjective factors, institutional factors, subjective factors and strategic factors. The current supply mode mainly included administrative supply mode, market oriented mode and socialization mode. Based on the research of the factors, mode, level, effectiveness and action framework of rural public service supply and considering the farmers’ psychological needs, this paper puts forward the action framework to promote the development of rural public service supply from five aspects, such as, we could guide the value orientation of rural public service supply, optimize the pattern of rural public service supply entities, improve the evaluation system for the effectiveness of rural public service supply, strengthening the policy and legal protection of rural public service supply, improve the organizational construction level of rural public service supply.

**Acknowledgments**

The authors received financial support for the research, authorship, and publication of this article. It was funded by Henan Philosophy and Social Sciences Planning Project in 2024 (2024CJJ136) and Henan Province Higher Education Philosophy and Social Science Innovation Talent Support Program in 2023 (2023-CXRC-21). We also would like to thanks for the support from Zhongyuan University of Technology Major Cultivation Plan in 2021.

## PHHS25045: GLOBAL RESEARCH LANDSCAPE AND TRENDS OF EGFR MUTATION NON-SMALL-CELL LUNG CANCER: A BIBLIOMETRIC ANALYSIS FROM 2000 TO 2022

Li Gan^a^, Gang Mai^b^, Yanghua Fei^a^, Chunmei Jiang^a^

^*a*^ *Department of Operations Management, People’s Hospital of Deyang City, Deyang 618000, Sichuan, China,* ^*b*^ *Hospital Office of Deyang People’s Hospital, Deyang 618000, Sichuan, China.*

**Background:** Epidermal growth factor receptor (EGFR) mutations are among the most common driver mutations in non-small-cell lung cancer (NSCLC), occurring in approximately 15–20% of cases. These mutations play a critical role in tumor development and response to targeted therapies, making them a significant focus of research. Despite advances in understanding and treating EGFR-mutant (EGFRm+) NSCLC, a comprehensive bibliometric analysis summarizing the vast body of research remains scarce. Such analyses are vital for identifying research hotspots, trends, and emerging directions, thereby guiding future studies effectively.

**Subjects and Methods:** This study systematically evaluated EGFRm+ NSCLC research by analyzing 27,427 publications retrieved from the Web of Science Core Collection. Bibliometric and visualization tools, including keyword analysis and network mapping, were used to assess publication trends, key institutions, influential authors, and primary research areas. The study also examined global collaborative networks and research growth trends spanning the past two decades.

**Results:** The number of publications on EGFRm+ NSCLC has increased steadily over the last 22 years, following a second-order polynomial growth trend. China led with 9,033 publications, followed by the USA (8,078), Japan (3,261), Italy (1,875), and Germany (1,682). Prominent academic institutions in the USA, China, and France significantly contributed, with the USA at the center of global collaboration networks. Major research focus areas included therapeutic strategies such as first-line treatment options, anticancer therapies, and PD-L1 expression, as well as diagnostic approaches like liquid biopsy for gene mutation testing. Additionally, early prevention and screening methods, including low-dose CT imaging and plasma biomarker identification, have gained increasing attention as critical tools for improving early detection and outcomes in high-risk populations.

**Conclusions:** The growing global interest in EGFRm+ NSCLC research underscores the need for enhanced international collaboration, particularly among countries outside the USA, to amplify research impact and foster innovation. Future studies are expected to emphasize advanced therapeutic modalities, innovative diagnostic tools, and preventive strategies, including lifestyle modifications and early detection programs. Strengthened partnerships between academia, industry, and international collaborators are essential for advancing scientific discovery, accelerating clinical translation, and ultimately improving patient outcomes worldwide. These collective efforts promise to transform EGFRm+ NSCLC research and treatment, addressing one of the most pressing challenges in oncology today.

## PHHS25046: THE IMPACT OF DIGITAL EMPOWERMENT ON FOOD AND AGRICULTURE OCCUPATIONAL COMPETENCE FROM THE PERSPECTIVE OF OCCUPATIONAL STRESS

Song You^a^, Jilan Bian^a^, Shaotong Ning^a^, Chenchen Wang^b^, Yu Tan^a^

^*a*^ *Heilongjiang Bayi Agricultural University, Daqing 163319, China,* ^*b*^ *Central State Grain Reserve Tangyuan Subordinate Warehouse Co., Tangyuan 154700, China*

**Background:** At present, China’s economic development has entered a new stage, and the main contradiction in society has transformed into the contradiction between the growing needs of the people for a better life and the unbalanced and inadequate development of the country. With the rise of per capita income, grain farmers cannot rely solely on increased output and prices to increase their income. They should also improve product quality and provide production protection to ensure food security and promote income growth. Heilongjiang Province is located in Northeast China and is one of the main grain producing areas in China. And the research object in this paper is the absolute subject of grain production - farmers, and as the digital transformation is updated, and the relationship between farmers’ professional growth and grain production is found to further solve the current.

**Subjects and Methods:** Using moderated mediation effects, the research used the AMOS27 software to examine the relationship between the four sub-variables of digital empowerment, social empowerment, transactional empowerment, and psychological empowerment, as well as the variable of professionalization intentions, and career growth in Heilongjiang Province, with 668 questionnaires. Furthermore, the research included the variable of risk perception to further alleviate the problem of occupational stress and improve mental and physical health.

**Results:** There is a significant positive correlation between digital empowerment and vocational ability. The vocational intention of food and agriculture workers plays a mediating role in the influence path of digital empowerment on the vocational ability of food and agriculture workers, and has a positive effect on the vocational ability of food and agriculture workers. Risk perception plays a moderating role in the mediating role between digital empowerment and vocational competence in food and agriculture.

**Conclusions:** To effectively mitigate occupational stress in the food and agriculture sectors, it is crucial to enhance the quality of digital empowerment and nurture the professional will it supports. By exploring the potential value of professional roles and aligning with the vocational call and orientation, we can increase risk awareness and encourage career advancement. This approach not only improves the physical and mental well-being of individuals but also alleviates the occupational stress that is inherent in these industries. Through these measures, we can foster a more resilient and competent workforce in food and agriculture, better equipped to face the challenges of their occupations.

**Acknowledgements**

This work was supported by a project grant from Soft Science Research Project of Heilongjiang Provincial Department of Science and Technology (Project No.: GC12D412).

## PHHS25047: APPLICATION OF TRADITIONAL ENDOCRINE THERAPY IN METASTATIC PROSTATE CANCER

Chongjian Zhang^a^, Yu Bai^b^, Haiyang Jiang^c^, Longguo Dai^d^, Ying Bi^e^

^*a*^ *Depatment of Urology, Peking University Cancer Hospital Yunnan Hospital Yunnan Provincial Cancer Hospital The Third Affiliated Hospital of Kunming Medical University in China., Kunming, 519 Kunzhou Road, 650118, Yunnan, China,* ^*b*^ *Depatment of Urology, Peking University Cancer Hospital Yunnan Hospital Yunnan Provincial Cancer Hospital The Third Affiliated Hospital of Kunming Medical University in China., Kunming, 519 Kunzhou Road, 650118, Yunnan, China,* ^*c*^ *Depatment of Urology, Peking University Cancer Hospital Yunnan Hospital Yunnan Provincial Cancer Hospital The Third Affiliated Hospital of Kunming Medical University in China. Kunming, 519 Kunzhou Road, 650118, Yunnan, China,* ^*d*^ *Depatment of Urology, Peking University Cancer Hospital Yunnan Hospital Yunnan Provincial Cancer Hospital The Third Affiliated Hospital of Kunming Medical University in China., Kunming,519 Kunzhou Road, 650118, Yunnan, China,* ^*e*^ *Depatment of Urology, Peking University Cancer Hospital Yunnan Hospital Yunnan Provincial Cancer Hospital The Third Affiliated Hospital of Kunming Medical University in China., Kunming,519 Kunzhou Road, 650118, Yunnan, China.*

**Background:** Prostate cancer is a hormone-dependent disease. When metastasis occurs, most patients undergo traditional first-line endocrine therapy. Initially, the disease is highly responsive to androgen deprivation therapy. However, after a median duration of 14-30 months, most patients transition to castration-resistant prostate cancer, rendering traditional endocrine therapy progressively ineffective. Selecting an appropriate follow-up treatment plan for patients is a challenging issue that urologists frequently encounter. Currently, there are numerous treatment options available for castration-resistant prostate cancer, including systemic chemotherapy, novel endocrine therapies, and second-line endocrine therapies within traditional treatments. Second-line endocrine therapies encompass the addition of anti-androgen drugs, substitution of anti-androgen drugs, cessation of anti-androgen drugs, and the inclusion of adrenal androgen inhibitors and estrogen compounds.

**Subjects and Methods:** By combining the clinical conditions of patients with metastatic prostate cancer and adopting a targeted individualized diagnosis and treatment plan that alternates between first-line drugs (castration + anti - androgen) and second-line drugs (estrogen blocker) in traditional endocrine therapy, the progression of the disease can be better controlled, allowing patients to achieve long-term survival.

**Results:** Based on the patient’s condition, the metastatic prostate cancer is treated using a traditional endocrine therapy approach that involves alternating between first-line drugs (castration + anti-androgen therapy) and second-line drugs (estrogen). Regular reviews and monitoring of the patient’s condition are conducted. As of February 2020, the patient’s condition remains stable, with no clinical symptoms such as bone pain, hematuria, or difficulty urinating observed. The testosterone levels are all below 20 ng/dl.

**Conclusions:** Through the study of individualized treatment options for this patient with metastatic prostate cancer, including surgical treatment (radical prostatectomy, external radiation therapy or close range radiation therapy), first-line or second-line traditional endocrine therapy, intermittent endocrine therapy, traditional endocrine therapy and intermittent endocrine alternating therapy, systemic chemotherapy, targeted therapy, immunotherapy, etc., it is hoped that urologists and oncologists can have more treatment ideas and strategies to choose from when treating patients with metastatic prostate cancer (hormone sensitive prostate cancer or castration resistant prostate cancer) in clinical practice, providing first-line treatment opportunities for metastatic prostate cancer patients, prolonging their survival time, and improving their quality of life.

**Acknowledgments**

National Natural Science Foundation of China (No.82160511); Young and Middle-aged Academic and Technical Leaders Reserve Talent Program of Yunnan Province (202305AC160053); Medical and Healthcare Professionals Project of Xingdian Talent Support Program in Yunnan Province (XDYC-YLWS-2023-0023); Yunnan Province Dong Jintang Expert Workstation.

## PHHS25048: IMPROVING TECHNICAL EFFICIENCY FOR COMMERCIAL BANKS FROM THE PERSPECTIVE OF BOLDLY EMBRACING UNCERTAINTY

Haitao Duan^a^, Muquan Zou^a,b,c^, Shuangfeng Li^d^, Vanha Tran^e^, Rong Ye^a^, Jiankun Yang^b^

^*a*^ *Fudian Bank Co. LTD, Kunming 650200, Yunnan, China,* ^*b*^ *Kunming University, Kunming 650214, Yunnan, China,* ^*c*^ *Yunnan Key Laboratory of Intelligent Logistics Equipment and Systems, Kunming 650214, Yunnan, China,* ^*d*^ *China Mobile Communications Group Yunnan Co. LTD, Kunming 650200, Yunnan, China,* ^*e*^ *FPT University, Hanoi 11517, Vietnam.*

**Background:** Management of uncertainty has become a chronic disease afflicting enterprises especially for commercial banks. Given a certain input, output is almost predictable in deterministic operation, so it can be said that uncertainty management may seriously affect output expectations, and then affect the technical efficiency of commercial banks. However, the traditional technical efficiency measurement method does not pay enough attention to the management level of uncertainty, which leads to the cognitive anxiety of management level. Meanwhile, it also takes the frontier as reference and then ignores the overall development level of the industry, which is easy to lead to learned helplessness of non-frontier commercial banks.

**Subjects and Methods:** The technical efficiency for commercial banks has been improved on the overall industry instead of the frontier for rational estimation to avoid learned helplessness. Meanwhile, both the time effect and individual effects are eliminated to highlight uncertainty management for commercial banks. Additionally, liquidity coverage ratio and provision coverage ratio are introduced to verify the impact of bank risk control on the improved technical efficiency. Thus, the management of uncertainty is taken into account in technical efficiency to avoid cognitive anxiety.

**Results:** The empirical results show that the improved technical efficiency can better reflect the innovation level of China’s main commercial banks, showing heterogeneity among large state-owned banks, joint-stock banks and city commercial banks. Moreover, the liquidity coverage ratio shows a negative effect on technical efficiency and the provision coverage ratio vice versa. That is to say, the improved technical efficiency can fully reflect the real level of uncertainty management and provide strong support for alleviating decision-making anxiety.

**Conclusions:** Accordingly, the strategies to improve technical efficiency with little cognitive anxiety for commercial banks are suggested on management of risk and multi-endowments. With the certainty of management to deal with the uncertainty of risk to achieve the scientific and healthy development of commercial banks.

**Acknowledgments**

This work is a research result of Yunnan Province Philosophy and Social Science Planning Project (grant NO. ZK2024YB15), and was funded by the Special Basic Cooperative Research Programs of Yunnan Provincial Undergraduate Universities Association (grant NO. 202101BA070001-152, NO. 202101BA070001-155), the Science and Technology Projects of Yunnan Universities Serving Key Industries (grant NO. FWCY-QYCT2024016), and the Cai Yun Postdoctoral Program-Innovation Project (grant NO. ynbh23024). The authors have no competing interests to declare that are relevant to the content of this article.

## PHHS25049: A CASE REPORT OF DUAL IMMUNOTHERAPY FOR ADVANCED GALLBLADDER CARCER

Jinfang Li^a^, Zhixin Zhang^b^, Xiangwang Zhang^b^, Keying Bi^b^, Jianying Cai^b^, Guizhi Dong^b^, Yaohua Li^b^, Shujing Cui^c^, Yanling Tan^c^

^*a*^ *Department of Ultrasound, Weihai Municipal Hospital of Shandong Province, Weihai, Shandong, China 264200,* ^*b*^ *Weihai Medical Service Center, No. 970 Hospital, Joint Logistic Center of Chinese People’s Liberation Army, Weihai, China 264200.* ^*c*^ *Huakang Bayerbeck Cell Health Technology (Shandong) Co., Ltd., Weihai, Shandong, China 264200.*

*JL and ZZ contributed equally to this work.*

**Background:** There is a complex association between gallstones, cholecystitis, and gallbladder cancer. Gallstones are a significant risk factor for gallbladder cancer, and long-term gallbladder inflammation, especially chronic cholecystitis caused by gallstones, is considered a major contributing factor in the development of gallbladder cancer. Gallbladder cancer is the most common malignancy of the biliary tract with a poor prognosis. The diagnosis and treatment of gallbladder cancer are challenging, requiring a comprehensive evaluation of clinical, imaging, and pathological information for accurate diagnosis, especially in the context of coexisting cholecystitis or gallstones, and appropriate treatment options should be selected according to the specific situation of the patient. And it’s a poor prognosis, and conventional treatments such as surgery and chemotherapy have limited efficacy.

**Subjects and Methods:** A patient underwent surgery after anti-infection treatment for suppurative cholecystitis and gallstones. Postoperative diagnosis was “1. Moderately differentiated adenocarcinoma of the gallbladder with lymph node metastasis; 2. Gallstones with chronic cholecystitis.” The patient had a history of cholecystitis and gallstones for 8 years. Gene and immune tests suggested no clear targeted drug; microsatellite stability, low tumor mutation burden, and low benefit from PD-1/PD-L1 immunotherapy. The patient received 6 cycles of durvalumab combined with gemcitabine and cisplatin for 4 months postoperatively, a total of 22 doses of durvalumab immunotherapy, 9 treatments of cord blood NK (Natural killer cell) immunotherapy, and 9 treatments of autologous Tcm (central memory T cell) immunotherapy. Immunotherapy was combined with each dose of durvalumab and after imaging examinations. The patient underwent routine follow-up examinations every 3 months, followed by immunotherapy.

**Results:** Multiple follow-up abdominal MRI and PET-CT scans showed stable disease. The last follow-up was at the end of October 2024, and all indicators suggested stable disease, evaluated as PR, with an ECOG performance status score of 0.

**Conclusions:** Gallbladder cancer (GBC) is the most common and lethal biliary malignancy, lacking effective treatments. However, the combination of chemotherapy, immunotherapy, and cell therapy has maintained stable disease for 2 years, which is inseparable from cord blood NK and autologous Tcm immunotherapy.

**Acknowledgments**

We would like to express our sincere gratitude to all those who have contributed to this study. First and foremost, we thank the patients who generously provided their medical records. We are also deeply grateful to Professor Zhou Aiping, Director of the Department of Oncology, Cancer Hospital, Chinese Academy of Medical Sciences, for her invaluable support. Finally, we would like to acknowledge Huakang Bayerbeck Cell Health Technology (Shandong) Co., Ltd., and the research team of Dr. Li Yaohua, comprising experts in medicine, immunology, cell biology, and molecular biology, for providing the cell therapy and their dedicated efforts.

## PHHS25050: RESEARCH ON THE IMPACT OF REAL ESTATE INVESTMENT PYCHOLOGICAL NEEDS ON URBAN INNOVATION EFFICIENCY

Mi Zhang^a^, Ying Du^a^, Mingxiu Yang^a^, Yueying Zhou^a^

^*a*^ *School of Humanities and Management, Hunan University of Chinese Medicine, Changsha, Hunan, China.*

**Objectives:** With the development of globalization and informationization, urban innovation has become an important driver for economic growth and enhancing competitiveness. The sustained rapid increase in real estate prices in China has become a significant factor affecting urban innovation and development, with the prosperity of the real estate market often accompanied by fluctuations in investment psychology. Factors such as investors’ expectations, risk preferences, and pychological needs all influence investment behaviors in the real estate market. Moreover, changes in the real estate market affect urban innovation efficiency through multiple aspects such as financing constraints, cost effects, and industrial structure. Starting from the current background of excessive reliance on real estate investment to drive the economy across various regions in China, this study explores the impact mechanism and its operational mechanism on urban innovation efficiency from the perspective of real estate investment psychology.

**Methods:** Based on panel data from 244 prefecture-level and above cities in China from 2005 to 2019, the study examines the impact and mechanism of real estate investment on regional innovation efficiency from the three core elements of entrepreneurs pychological needs, capital, and technology, combined with the “cognitive–behavioral” psychological perspective.

**Results:** The study finds that real estate investment significantly reduces urban innovation efficiency; the higher the proportion of real estate investment in GDP, the lower the urban innovation efficiency. Heterogeneity analysis shows that the inhibitory effect of real estate investment on urban innovation efficiency is greater in the eastern regions, coastal cities, and after the 2008 international financial crisis. This remains valid after multiple robustness tests and using instrumental variables to overcome endogeneity issues. The research results further enrich the analysis of the constraints on the impact of real estate investment on urban innovation efficiency from the perspective of real estate investment pychological needs.

**Conclusions:** Through analyzing the impact mechanism of real estate investment on regional innovation efficiency, it is found that the impact of real estate investment on urban innovation efficiency is a complex and multidimensional issue to some extent. Real estate investment mainly negatively affects urban innovation efficiency through channels such as fiscal expenditure bias, industrial hollowing-out, and distorted human capital allocation. The data results indicate that excessive real estate investment is an important reason hindering the improvement of urban innovation efficiency. Promoting the investment psychology of real estate developers and the stable and healthy development of the real estate market is of significant importance for enhancing urban innovation efficiency.

**Acknowledgements**

This work was supported by 2024Hunan Provincial College Students’ Innovative Entrepreneurial Training Plan Program (NO. 2993).

## PHHS25051: EXPLORATION OF THE QUALITY MANAGEMENT PATHWAY FOR UNIVERSITY-LEVEL ENTREPRENEURSHIP AND INNOVATION EDUCATION BASED ON OBE-CDIO

Yan Cui^a^, Qiang Liu^a^, Ying Xi^a^

^*a*^ *Xi’an Mingde Institute of Technology, Xi’an, Shaanxi, 710124, China.*

**Background:** This scholarly investigation is designed to construct and execute a quality management framework for higher education entrepreneurship and innovation (E&I) programs, grounded in the OBE-CDIO educational paradigm. The primary goals are to bolster students’ innovation and enterprise capabilities, ensure their comprehensive competencies, and fortify their psychological resilience against stress. This initiative seeks to cultivate a well-rounded educational environment that primes students for the complexities of the contemporary professional landscape.

**Subjects and Methods:** The study centers on Xi’an Mingde Institute of Technology, deploying a multifaceted quality assessment strategy with a focus on outcome-based evaluation. We have integrated engineering quality management techniques with psychological resilience assessments, instituting data-driven, standardized metrics. A teaching management model for continuous improvement has been crafted to periodically reassess the validity of assessment indicators and fine-tune evaluation parameters, ensuring a flexible and adaptive educational approach.

**Results:** The findings indicate a notable escalation in student satisfaction with the quality of teaching, coupled with marked enhancements in academic performance, psychological well-being, and overall student development. There is a significant strengthening of faculty teaching skills and a marked improvement in teaching outcomes. The standardization of teaching management has led to more efficient resource allocation, optimized utilization, and heightened operational efficiency. Remarkably, the engagement of students in competitive events has set new benchmarks in terms of participation rates and the number of projects undertaken.

**Conclusions:** The OBE-CDIO-informed teaching quality management trajectory has proven effective in elevating the caliber of E&I education, playing a pivotal role in the cultivation of enterprising and innovative talents, and in augmenting the psychological stress tolerance of university student

**Acknowledgments**

This work was supported by the following projects: the Shaanxi Provincial Higher Education Association’s 2021 Higher Education Science Research Project, “Research on the Mechanism of Industry-Education Integration in New Era Private Undergraduate Colleges” (Project Number: XGH21299); Xi’an Mingde Institute of Technology’s 2023 Mingde Innovation Fund Project, “The Influence Mechanism of Scientific Literacy on College Students’ Innovation and Entrepreneurship Abilities” (Project Number: 2023MDN12); and Xi’an Mingde Institute of Technology’s 2023 Key Project for Education and Teaching Reform, “Exploration and Practice of Building an Applied Undergraduate College Innovation and Entrepreneurship College (College Student Innovation and Entrepreneurship Incubation Park) from the Perspective of Four Innovation Integration” (JG2023ZD07).

## PHHS25052: EVALUATION OF THE APPLICATION EFFECT OF HYBRID LEARNING MODEL IN INTERNAL MEDICINE NURSING COURSES BASED ON MENTAL HEALTH PERSPECTIVE

Di Zhu^a,b^, Xinxin Liu^c^, Jian Song^d^, Ya Meng^e^, Jian Zhang^f^, Yan Shi^g^

^*a*^ *Henan Health Cadre Institute, Department of medicine, Zhengzhou 450008, China,* ^*b*^ *Henan Health Cadre Institute, Academic Affairs Office, Zhengzhou 450008, China,* ^*c*^ *Medical School of Jingchu University of Technology, Jingmen 448000, China,* ^*d*^ *Jingzhou Institute of Technology, Department of nursing, Jingzhou 434000, China,* ^*e*^ *Zhengzhou Health Vocational College, Zhengzhou 450008, China,* ^*f*^ *Nanyang Vocational College of Agriculture, Mental Health Education Center, Nanyang 473000, China,* ^*g*^ *Zhengzhou University, School of Nursing and Health, Zhengzhou 450001, China.*

*DZ and XL contributed equally to this research.*

**Objective:** This study aims to analyze the main issues in implementing the Hyflex learning model in internal medicine nursing courses during and after a major health event. From the perspective of mental health, it endeavors to enhance students’ independent learning and critical thinking abilities, and promote their psychological well-being through innovative teaching methods, thus providing references for future curriculum development.

**Subjects and Methods:** A convenience sampling method was used to investigate nursing students in a health college. Self-designed questionnaires included a general situation questionnaire covering aspects that may affect learning and psychological state, such as age, gender, and network signals, as well as a feasibility questionnaire for the Hyflex learning model of internal medicine nursing. Purposive sampling and data saturation methods were used for in-depth interviews to supplement the quantitative research. SPSS 25.0 was used for statistical analysis of the data, and tables with more than 2/3 unanswered items were excluded, and double entry was used to check errors.

**Results:** A total of 500 student questionnaires were distributed, with 418 valid ones recovered (83.6%). The data showed students’ diverse demographics. Regarding the Hyflex model, most students had some interest in internal medicine nursing courses (27.51% with great interest, 68.42% with general interest), and many were familiar with online teaching platforms (27.99% knew more, 62.92% generally knew). They mainly solved learning problems through web search, teacher consultation, and peer communication. Interviews revealed themes of improving learning ability, avoiding constraints, and enhancing family support. Online learning resources helped some students relieve anxiety and build confidence, but technical issues caused anxiety in those overly dependent on platforms. Offline interactions reduced students’ loneliness. However, the rich and diverse tasks of hybrid learning led to time management stress for some students.

**Conclusions:** The Hyflex learning model, chosen due to the major health event, is generally accepted. It combines online and offline learning, with online platforms enabling interaction when students can’t attend school and facilitating independent learning. After returning to school, students can switch methods. To improve teaching quality and students’ mental health, schools should enhance platform support and training, and teachers should guide students in time management and learning strategies. By optimizing the hybrid learning model and addressing mental health issues, we can cultivate nursing students with both psychological health and strong professional abilities.

**Acknowledgments**

This research was funded by the 2024 annual survey project of the Federation of Social Sciences of Henan Province (number: SKL-2024-777), the 2024 annual scientific research project of Jingmen City’s “14th Five-Year” Education Science Planning (number: JMG2024008), and the science and technology project of Jingchu Technology University (number: QN202420).

## PHHS25053: RESEARCH ON THE TEACHING REFORM PATH OF LOGISTICS COURSE UNDER CONCEPTS OF THREE-SCIENTIFIC EDUCATION AND OBE BASED ON ANALYSIS OF STUDENTS’ PSYCHOLOGICAL NEEDS

Wei Chang^a^, Jianzhou Wang^b^

^*a*^ *Equipment Management and Support College, Engineering University of PAP, Xi’an 710086, China,* ^*b*^ *Yichun Detachment, Heilongjiang Provincial Corps of PAP, Yichun 153000, China.*

**Background:** This research aims to analyze teaching and learning status of logistics course at a certain university in Xi’an, and to promote the effective implementation of the teaching reform of logistics courses that matches psychological needs of students at similar universities.

**Subjects and Methods:** With the rapid development of the digital economy, logistics industry has undergone great changes, and the teaching reform of logistics courses at universities is particularly crucial. Three-scientific education concept emphasizes that in the process of teaching and learning, we should not only pay attention to the cultivation of scientific spirit and scientific and technological literacy, but also open up the road to students’ success through the improvement of scientific research and innovation ability. The outcome-based education concept is a student-centered and output-oriented education model that can better prepare students for the future. These two educational concepts point out the important direction of the teaching reform of logistics courses at universities. Based on analyses of students’ psychological needs and university course evaluations, pain points of teaching and learning are discovered. Then targeted implementation path of teaching reform under the guidance of three-scientific education and outcome-based education is developed.

**Results:** Three pain points in logistics teaching are discovered including weak timeliness, teacher centeredness, and reduced practicality. Under the guidance of three-scientific education and outcome-based education, a targeted reform path aiming for modernization, practicalization, and individuality can foster the effective implementation of the teaching reform and deepen students’ positive psychological experience of logistics courses at similar universities.

**Conclusions:** Reconstruction of logistics course at universities is an inevitable choice to achieve dual improvements of teaching quality and student abilities, and is also a basic requirement for the development of three-scientific education and outcome-based education. It suggests that in the process of logistics course reconstruction, measures such as project-based teaching, school-enterprise joint practical teaching, virtual simulation teaching, establishing student feedback mechanisms, promoting learning through competitions, incorporating resources to establish learning community etc should be carried out to overcome challenges of weak timeliness, teacher centeredness, and reduced practicality, cultivate students’ practical application ability and technological innovation ability, meet students’ psychological needs, and provide high-quality professional talents with both innovative consciousness and practical ability for China’s modernization construction.

## PHHS25054: EXPLORING THE INTERPLAY OF TRANSFORMATIONAL LEADERSHIP, TRUST, AND INNOVATION IN EDUCATION: A PSYCHOLOGICAL HEALTH PERSPECTIVE

Tian Wang^a^, Jianbang Lin^b^

^*a*^ *Guangdong University of Foreign Studies South China Business College, Guangzhou, China,* ^*b*^ *Nanfang College • Guangzhou, Guangzhou, China.*

**Objective:** This study investigates the intricate relationships between transformational leadership, teacher trust, and teacher innovative behavior by integrating insights from psychological health theories. The primary objective is to explore how these elements interplay within educational settings and to propose actionable strategies to cultivate innovation among educators and school leaders.

**Subjects and Methods:** Drawing from extensive literature, this study applies three psychological health theories—Emotional Intelligence Theory, Attachment Theory, and Flow Theory—to the constructs of leadership, trust, and innovation. Emotional Intelligence Theory is employed to examine the emotional competencies of transformational leaders in inspiring trust and motivating innovation among teachers. Attachment Theory provides a framework for understanding teacher trust, focusing on the development of secure and supportive relationships between teachers and their leaders or colleagues. Flow Theory is utilized to analyze teacher innovative behavior, highlighting how optimal engagement and intrinsic motivation drive creativity and the implementation of innovative practices. By synthesizing theoretical and empirical findings, this study identifies pathways to enhance organizational and educational outcomes.

**Results:** The analysis reveals that transformational leadership, underpinned by emotional intelligence, plays a pivotal role in fostering an environment conducive to trust and innovation. Leaders with high emotional intelligence build strong relationships and communicate effectively, creating a climate of psychological safety that encourages teachers to take creative risks. Attachment Theory underscores the importance of secure, trusting relationships among teachers and between teachers and leaders, which are critical for collaboration and knowledge sharing. Flow Theory illustrates that teachers are most innovative when they experience deep engagement in tasks, achieved through a balance between task difficulty and their personal capabilities. Together, these theories provide a comprehensive understanding of the factors that drive teacher innovation.

**Conclusions:** This study emphasizes the value of integrating psychological health theories into educational leadership practices. Transformational leaders equipped with emotional intelligence foster trust and innovation by nurturing psychologically safe and supportive environments. Secure attachments among educators, as explained by Attachment Theory, strengthen collaboration and collective creativity. Meanwhile, Flow Theory demonstrates that fostering engagement and intrinsic motivation among teachers significantly enhances their capacity for innovation. The findings offer a multidimensional framework for educational leaders seeking to align leadership practices with psychological insights, enabling schools to meet the demands of a rapidly evolving educational landscape.

**Acknowledgments**

This work was supported by a key project grant from SCBC: Research on the Impact of Transformational Leadership in Private Universities on Teacher Innovative Behavior (Grant No. 23-002A), Guangdong Provincial Department of Education Accounting Professional Course Ideological and Political Teaching Team (Grant No. 202121056), and Key Research Base of Social Sciences in Universities of Guangdong Province: Guangdong Research Base of Digital Development and Management Innovation of Service Industry (Grant No. 2022WZJD013), and a project grant from 2021 Guangdong Key discipline research ability improvement project (Grant No. 2021ZDJS129).

## PHHS25055: EXPLORATION OF THE POSITIVE IMPACT AND MECHANISM OF MARTIAL ARTS ON COLLEGE STUDENTS’ MENTAL HEALTH

Fei Yang^a^, Yanfang Dong^a^, Lei Lu^a^

^*a*^ *Sports Teaching and Research Section, Basic Teaching Department, Xinjiang University of Political Science and Law, Tumushuke 843900, Xinjiang, China.*

**Background:** In recent years, with the frequent occurrence of campus bullying, suicides, and poisoning incidents, the mental health issues of college students have increasingly garnered societal attention. As a traditional sport of the Chinese nation, Martial Arts, while strengthening the body, has also increasingly highlighted its positive effects on mental health.

**Subjects and Methods:** This study employed an experimental research method, conducting an in-depth analysis of the collected data through questionnaires and statistical analysis. It focuses on the positive impact of Martial Arts education on college students’ mental health and its underlying mechanisms, aiming to scientifically assess the physical and mental health benefits of Martial Arts and promote its widespread dissemination and application in universities.

**Results:** The research results indicate that the mental health qualities of college students primarily encompass six aspects: wisdom and knowledge, courage, benevolence, fairness, moderation, and excellence. Martial Arts significantly enhances the physical and mental health of college students, while also exerting a positive influence on the shaping of their internal and external characters, effectively bolstering their psychological resilience. Mental health education should not be limited to troubled students alone; rather, it should be extended to all students, inspiring their potential and positive emotions.

**Conclusions:** Therefore, Martial Arts training is not merely a physical exercise and shaping process, but also a profound transmission of culture and cultivation of values. Through receiving Martial Arts education, students can gain a deeper understanding of themselves, uncover their inner values, and subsequently significantly enhance their sense of self-efficacy. With its unique charm, Martial Arts training effectively alleviates the psychological pressure and burdens that students face due to academic workloads and challenges in adapting to life, thereby establishing a solid defense for their mental health. This modern educational approach, integrated with traditional culture, provides a firm cultural foundation and spiritual support for college students seeking a sense of belonging and identity in modern society. It not only promotes the comprehensive development of students’ physical and mental health but also equips them with the mental strength to tackle the challenges of modern society.

## PHHS25056: THE IMPACT OF TRANSPORTATION INFRASTRUCTURE IMPROVEMENT ON THE DEVELOPMENT OF TOURISM INDUSTRY AND TOURISTS’ MENTAL HEALTH ETHNIC REGIONS OF WESTERN SICHUAN, CHINA

Chunsheng Mu^a^, Noor Hafiza Ismail^b^

^*a*^ *ABA Teachers College, Wenchuan 623002, Sichuan, China,* ^*b*^ *Faculty of Information Sciences and Engineering, Management & Science University, Shah Alam 40100, Selangor, Malaysia.*

**Objective:** Transportation infrastructure plays a crucial role in regional economic development, particularly in the tourism industry, where accessibility and convenience significantly impact tourist experiences, satisfaction, and well-being. This paper examines how the construction of the Sichuan-Qinghai Railway influences tourism development in the ethnic regions of western Sichuan. It explores how improved transportation accessibility, reduced travel costs, and alleviated traffic pressure during peak seasons enhance regional tourism resources and promote industrial coordination. Additionally, the study investigates the complex effects of transportation infrastructure on tourists’ mental health, including emotional well-being, anxiety, and stress management. Through this research, we aim to provide a theoretical foundation for the development of ethnic tourism resources and optimize tourists’ travel experiences in western Sichuan, offering practical guidance for regional economic development and rural revitalization.

**Subjects and Methods:** This study employs the Analytic Hierarchy Process (AHP) to develop an evaluation model that incorporates four core elements: transportation accessibility, transportation costs, tourism attractiveness, and discovery benefits. These elements are analyzed in-depth to assess their direct and indirect effects on tourism development and tourists’ mental health. To ensure the reliability of the results, expert weights were assigned, with a sample of 15 experts from transportation planning, the tourism industry, ethnic studies, and government policy-making. This methodology allows for a comprehensive understanding of the impact of transportation infrastructure on both tourism development and the psychological experience of tourists, particularly in terms of anxiety, emotional satisfaction, and stress during peak seasons.

**Results:** The construction of the Sichuan-Qinghai Railway has significantly enhanced transportation accessibility, reduced travel time, and lowered travel costs. These improvements have alleviated congestion during peak tourist seasons, reducing the psychological stress of long journeys and concerns about road safety. Improved transportation access allows tourists to spend more time enjoying scenic spots, contributing to better mood management and reduced anxiety. Moreover, the enhanced infrastructure has facilitated the preservation and exploration of ethnic cultures, boosting the visibility of regional cultural heritage and supporting the growth of related tourism industries.

**Conclusions:** The construction of the Sichuan-Qinghai Railway has not only promoted tourism development but also positively impacted tourists’ mental health. By addressing stressors such as long travel times and congestion, the railway offers tourists a better emotional experience, helping them manage anxiety, stress, and negative emotions. These positive psychological effects have contributed to greater overall satisfaction and increased tourist retention. This study underscores the importance of considering mental health in the planning of transportation infrastructure in tourism-centric regions. The findings provide valuable insights for policymakers, highlighting the dual role of transportation infrastructure in fostering economic growth and enhancing tourists’ psychological well-being. A holistic approach to infrastructure development is essential for the sustainable growth of tourism and the overall prosperity of the region.

**Acknowledgements**

This work was supported by a project grant from the Sichuan Nationalities and Mountain Economy Development Research Centre, a key research base of humanities and social sciences of the Sichuan Provincial Department of Education, for the project “Research on the Promotion of Tourism Industry Development through Transportation Infrastructure Construction” (No. SDJJ202420), and the research project of the Sichuan Outdoor Camp Education Development Research Centre for the study “Research on the Design and Branding of Outdoor Camps in Tibetan and Qiang Ethnic Areas of Sichuan” (No. HWYD2024B12).

## PHHS25057: DISCRIMINATION AND ANALYSIS OF MENTAL DISORDERS BASED ON PROFESSOR ZHOU ZHONG-YING’S LATENT TOXIN

Yating Liao^a,b^, Xiaoshuo Jing^a,b^, Ning Yuan^c^, Ping Yang^c^, Liang Li^a^, Junfeng Yan^a,b^

^*a*^ *School of Information Science and Engineering, Hunan University of Chinese Medicine, Changsha 410208, Hunan, China,* ^*b*^ *Hunan Province Wisdom Engineering Technology Research Center, Changsha 410208, Hunan, China,* ^*c*^ *Hunan Provincial Brain Hospital, Changsha 410208, Hunan, China.*

**Objective:** This study introduces the concept of latent toxins proposed by Professor Zhou Zhong-ying, a master of traditional Chinese medicine, into the field of mental disorders. The purpose is to provide a new perspective and theoretical support for the diagnosis and treatment of mental disorders. Mental disorders are traditionally associated with imbalance of yin and yang, visceral dysfunction, abnormal qi, blood and body fluids, and impaired brain and mental function.

**Subjects and Methods:** Mental disorders include a series of mental and psychological diseases of traditional Chinese medicine, which is the focus of this study. In view of these diseases, the concept of latent toxin is discussed from the perspective of traditional Chinese medicine. According to the characteristics of latent toxin latency, specificity, variability, crosslinking and complexity, the influence and therapeutic effect of latent toxin in mental disorders are analyzed and explained by traditional Chinese medicine theory.

**Results:** The introduction of latent toxins into the field of mental disorders in traditional Chinese medicine reveals new insights into the pathogenesis of these diseases. By examining the syndrome differentiation and treatment of diseases, it was found that latent toxin may be an important factor leading to mental disorders. This study provides a new perspective and theoretical support for the diagnosis and treatment of mental disorders in traditional Chinese medicine.

**Conclusions:** This study concludes that Latent toxin, as proposed by Professor Zhou Zhong-ying, offers a valuable new perspective for understanding and treating mental disorders. By incorporating this concept into the framework of traditional Chinese medicine, it is possible to gain a deeper understanding of the pathogenesis of mental disorders and to develop more effective treatment strategies. The study emphasizes the importance of considering Latent toxin in the diagnosis and treatment of mental disorders, contributing to the advancement of traditional Chinese medicine in this field.

**Acknowledgements**

The research was supported by the General Program of the National Natural Science Foundation of China (Grant No. 82374323), the Key Project of Scientific Research of Hunan Provincial Department of Education (Grant No. 23A0312), the Research Center of Hunan Provincial Department of Science and Technology (Grant No. 2023SK4020), and the Hunan Provincial Health Commission (Grant No. 20230060).

## PHHS25058: THE EFFECT OF HIGH-ENERGY RADIAL SHOCK WAVE THERAPY COMBINED WITH ACUPOINT INJECTION ON THE TREATMENT OF KNEE OSTEOARTHRITIS: A CLINICAL RANDOMIZED CONTROLLED TRIAL

Tian Zhang^a^, Hongju Liu^a^, Hua Li^a^, Lu Huang^a^

^*a*^ *Department of Rehabilitation Medicine, Beijing Jishuitan Hospital Guizhou Hospital, Guiyang, 550000, Guizhou, China.*

**Background:** The purpose of this study was to analyze the efficacy of high-energy radial shock wave therapy (RSWT) combined with acupoint injection for Knee Osteoarthritis (KOA) and to summarize the application experience.

**Subjects and Methods:** A total of 156 patients with KOA were enrolled in this trial and randomly divided into two groups. The control group (n=75) was treated with acupoint injection only (Zu shi ma, 4ml per injection, 4 points for each treatment, injecting 1ml into each point after strict disinfection, once a day, 5 times a week, for a continuous 2-week treatment period). The research group (n=81) was treated with acupoint injection and high-energy RSWT (the output pressure was 2.5-3.5bar, the frequency was 10-13Hz, and the energy was 0.18-0.25mj/mm². Three treatment points were selected for each treatment, with each point impacted by 1000 shocks, totaling 3000 shocks; treatment was administered once every 3 days, with 5 sessions constituting one course of treatment). The visual analogue pain scale (VAS), Activities of Daily Living (ADL), and knee function score (Lysholm) were used to evaluate clinical efficacy before and after treatment, as well as 4 and 8 weeks after treatment. The adverse effects of the two groups were observed during the treatment period.

**Results:** The clinical efficacy of the enrolled cases was assessed at the end of treatment, and 4 and 8 weeks post-treatment. Post-treatment, the ADL scores of patients in both groups significantly improved compared to pre-treatment scores, with statistical significance (P<0.05), while the pain scores of the research group were significantly better than those of the control group at 8 weeks post-treatment (P<0.05). The Lysholm score in both groups at the end of treatment and 4 weeks post-treatment significantly improved compared to pre-treatment scores (P<0.05), with similar effects observed between groups (P>0.05). However, the Lysholm score of the research group was significantly better than the control group at 8 weeks post-treatment (P<0.05).

**Conclusions:** The application of high-energy radial shock wave therapy in conjunction with acupoint injection can alleviate knee pain, improve knee function, and enhance daily living abilities in patients with knee osteoarthritis. Our data suggest that this combination therapy is a good option, characterized by its safety, effectiveness, long-lasting curative effect, and minimally invasive nature. Therefore, we conclude that it is a useful method for KOA patients.

**Acknowledgments**

This research was supported by a project grant from Chinese Medicine and Ethnic Medicine Science and Technology Projects of Guizhou Provincial Administration of Traditional Chinese Medicine (Grant No: QZYY-2023-041).

## PHHS25059: EMPIRICAL STUDY ON DIGITAL ECONOMY BOOSTING THE HIGH-QUALITY DEVELOPMENT OF CULTURAL TOURISM UNDER TOURISTS’ PSYCHOLOGICAL NEEDS

Zuozhi Li^a^, Yunke Feng^a^

^*a*^ *Tiangong University, Tianjin 300387, China.*

**Background:** Against the backdrop of the surge in demand for cultural tourism, issues such as crowded tourism, the spread of negative public opinion, and the discrepancy between the level of public service provision and tourists’ psychological needs is occasionally appearing, which affects the development of high-quality cultural tourism. The cultural tourism industry and digital economy have a high degree of adaptability, and the development of the digital economy provides a new opportunity for the integration and transformation of cultural tourism, changes the traditional factors of production, and promotes the formation of high-quality cultural tourism.

**Subjects and Methods:** Based on the current situation of culture and tourism integration in China and the panel data of provincial areas from 2011 to 2021, this study calculates the level of high-quality development of culture and tourism using the entropy weight method and coupled coordination degree model. It examines the indicators of the digital economy, high-quality culture, and tourism development and establishes a model of the two to analyze the influence of the digital economy development levels and trends on the advancement of high-quality culture and tourism in the setting of tourists’ psychological needs owed to cultural tourism innovation.

**Results:** The level of high-quality development of cultural tourism (HCT) in China’s regions has changed to varying degrees over the past decade. The marginal impact of the digital economy (Dig) on HCT is 1.0399, demonstrating that the digital economy can directly improve the high-quality development of cultural tourism. The channel mechanism of action model has a marginal impact of 0.634 with the addition of the mediator variable, which suggests that the GDP plays a partial mediating role. The empirical model of the moderating mechanism of action has a marginal impact of 0.692 with the addition of the moderating variable, the level of public service supply to meet tourists’ psychological needs, which has a positive moderating effect. The endogeneity discussion and robustness test significantly support the above findings.

**Conclusions:** The direct, channel, and regulating action mechanisms are all established, which can effectively promote the development of high-quality cultural tourism under the condition of meeting the psychological expectations of tourists’ psychological needs. Therefore, it is of great significance to explore the intrinsic logic of the digital economy, characterized by digitization and intelligence, to promote the high-quality development of cultural tourism.

**Acknowledgments**

This work was supported by a project grant from Tianjin Art and Science Planning Project (Grant No. B20009).

## PHHS25060: HEZHOU UNIVERSITY SHIJING MOUNTAIN TEA GARDEN: A STUDY ON HEALING LANDSCAPE DESIGN CENTERED ON PSYCHOLOGICAL NEEDS

Yile Sun^a,b^, Longping Ye^a,b^

^*a*^ *Hezhou University, Guangxi, Hezhou, China,* ^*b*^ *Guangxi University Key Laboratory of Big Data for Innovative Design, Guangxi, Hezhou, China.*

**Background:** As modern life accelerates, university students face increasing psychological pressures and emotional challenges, demanding more from the healing functions of campus environments. The Shijing Mountain Tea Garden at Hezhou University, a vital green space on campus, holds potential for enhancing students’ psychological health and providing psychological support. From the perspective of psychological needs, exploring the significance of campus landscape design in promoting student psychological health has emerged as a new trend within the field.

**Subjects and Methods:** This study focuses on the Shijing Mountain Tea Garden at Hezhou University, aiming to explore how landscape design can satisfy students’ psychological needs and promote psychological health. The research methods include data collection and analysis, field surveys, and case studies, integrating theories from landscape ecology and aesthetics for a comprehensive analysis. The study will analyze space composition elements, features of the landscape, and the integration of cultural activities from the users’ perspective, with the goal of creating a healing landscape environment that is both highly functional and genuinely effective. Through field surveys, the study explores design methods for healing landscapes in terms of space, plants, and details, providing insights for creating healing spaces that meet students’ psychological needs, alleviate stress, and promote psychological health.

**Results:** The study will identify healing landscape elements in Shijing Mountain Tea Garden, such as plant species, structures, and paving, and discuss how these elements positively affect psychological states. A series of healing landscape design principles and strategies based on psychological needs will be proposed, offering optimization suggestions for the current spatial layout of the tea garden. This aims to enhance students’ emotional states, alleviate stress, and improve overall well-being.

**Conclusions:** The research identifies the strengths and weaknesses of Shijing Mountain Tea Garden in meeting students’ psychological needs, clarifying which design elements positively influence student psychological health. Specific spatial optimization suggestions are proposed, integrating local cultural elements into healing landscape design to enhance students’ sense of identity and belonging, thereby improving the healing potential of the tea garden to better serve students’ psychological needs.

**Acknowledgments**

This work was supported by the Guangxi University Key Laboratory of Innovative Design Big Data Project (GXUIDDL-2024001).

## PHHS25061: EXPLORATION OF THE SPATIAL IMPACT OF HUIZHOU-STYLE RESIDENCES ON ENVIRONMENTAL HEALING AND MENTAL HEALTH

Bowen Yu^a^, Ke Li^b^

^*a*^ *Weihai Vocational College, Weihai 264210, Shandong, China,* ^*b*^ *School of Literature and Journalism, Shandong University, China.*

**Background:** Huizhou architecture, as a gem in traditional Chinese architectural culture, is known for its unique design features and profound cultural connotations. Originating from Anhui Province in China, it is characterized by exquisite wood, brick, and stone carvings, as well as a deep understanding and respect for the natural environment. With the increasing emphasis on mental health in modern society, the concept of spatial healing has also gained attention. Spatial healing refers to the enhancement of mental health and well-being through architectural and environmental design. This paper aims to explore the design characteristics of Huizhou-style residences and their potential value in spatial healing, providing new ideas for modern architectural design.

**Subjects and Methods:** This study selected representative Huizhou-style residences as research subjects. Through literature review, case analysis, and field visits, the design elements of Huizhou-style residences were systematically analyzed. The research team went deep into the birthplace of Huizhou architecture to make detailed observations and records of local residences, while combining environmental psychology theory to explore the impact of these design elements on the mental health of residents. Environmental psychology is a discipline that studies the interaction between people and their environment, emphasizing the impact of the environment on psychological and behavioral aspects. In the study of Huizhou architecture, environmental psychology theory provides a framework for analyzing and understanding how Huizhou architecture promotes the mental health of residents through its unique design.

**Results:** The layout principles, architectural structures, and decorative arts of Huizhou-style residences not only reflect the harmonious coexistence with the natural environment but also play a significant role in promoting the health and well-being of users while providing a comfortable living environment. The layout principles of Huizhou architecture emphasize the adaptation and utilization of the natural environment, such as achieving good ventilation and lighting through reasonable orientation and spatial layout, which not only improves the comfort of living but also helps with the emotional regulation and mental health of residents. In terms of architectural structure, Huizhou architecture uses a wooden structural framework, which not only has good seismic performance but also gives a warm and comfortable feeling with the natural texture and color of wood. In terms of decorative arts, Huizhou architecture is known for its exquisite carvings, which not only have high aesthetic value but also stimulate the imagination and creativity of residents, thus positively affecting mental health.

**Conclusion:** The design characteristics of Huizhou-style residences have important implications for modern spatial healing. The design concepts and practices of Huizhou architecture provide valuable references for modern architectural design, especially in the field of spatial healing. The research in this paper not only provides theoretical support for the protection and inheritance of Huizhou architecture but also offers new ideas for modern architectural design. Future research should further explore the spatial healing effects of Huizhou architecture in different cultural backgrounds to promote its global promotion and application. This will not only enrich the diversity of global architectural culture but also help improve the mental health and quality of life of people worldwide.

## PHHS25062: A STUDY ON THE RELATIONSHIP BETWEEN COLLEGE STUDENTS’ INNOVATION ABILITY AND MENTAL HEALTH: A CASE STUDY OF SCIENTIFIC RESEARCH PROJECT

Hengzheng Li^a^, Zichen Tao^a^, Yinghe Qin^a^, Yanyan Meng^a^

^*a*^ *Suzhou University, Suzhou 234000, China.*

**Background:** Against the backdrop of the current emphasis on cultivating innovative talents in higher education, improving the scientific research and innovation capabilities of college students is one of the important goals of university education. Whether a college student has good scientific research and innovation capabilities is not only an important indicator to measure their eligibility but also a psychological need for them to prove their abilities. Self-efficacy is an individual’s belief and judgment about their ability to successfully complete a certain behavior. It plays a vital role in the scientific research activities of college students. It affects students’ choices of research tasks, their level of commitment, and the way they deal with difficulties, thus having a profound impact on the development of their scientific research and innovation capabilities. Moreover, self-efficacy is closely linked to students’ mental health, as it can influence their stress levels, motivation, and overall well-being during the research process.

**Subjects and Methods:** Taking the implementation of a laser surface strengthening project among college students as an example, this study delves deeply into the influence of self-efficacy on college students’ scientific research and innovation capabilities. Through the investigation and analysis of the students participating in the project, this study elaborates on the important roles of self-efficacy in stimulating students’ interest in scientific research, enhancing their scientific research skills, promoting teamwork, and dealing with setbacks in scientific research. Additionally, the study incorporates mental health assessments to evaluate the psychological impact of self-efficacy and research activities on students.

**Results:** The research results show that self-efficacy plays an irreplaceable role in stimulating students’ interest in scientific research, enhancing their scientific research skills, promoting teamwork, and strengthening their resilience in the face of setbacks. When cultivating college students’ scientific research and innovation capabilities, colleges and universities should attach great importance to the cultivation of students’ self-efficacy. By adopting various strategies such as providing successful experiences, strengthening skill training, and creating a good atmosphere, colleges and universities can enhance students’ self-efficacy, thus promoting the comprehensive improvement of college students’ scientific research and innovation capabilities. This, in turn, contributes to better mental health and overall well-being among students.

**Conclusions:** The study concludes that there is a strong relationship between college students’ innovative abilities and their mental health, mediated by self-efficacy. Enhancing self-efficacy not only boosts students’ scientific research and innovation capabilities but also positively impacts their mental health. Universities should integrate mental health support into their research training programs, providing students with resources and strategies to manage stress and anxiety. Future research should further explore the long-term effects of self-efficacy and mental health on students’ career development and overall life satisfaction.

**Acknowledgments**

This work was supported by the Scientific Research Foundation for Doctoral of Suzhou University (2020BS009), the Collaborative Education Project of the Ministry of Education (230703879265905, 230706071265258), Teaching Research Project of Suzhou University (szxy2024jyjf55), Anhui Province Excellent Talent Support Program for Universities (gxyq2020059), the Natural Science Research Project in Universities of Anhui Province in China (2022AH051386, 2023AH010055), Anhui Higher Education Quality Engineering Project (2022jyxm1595, 2020gnxm070, 2021jyxm1502, 2020mooc566).

## PHHS25063: EXPLORING THE MECHANISM OF MONGOLIAN MEDICINE MAQIANZI-2 IN TREATING PERIPHERAL FACIAL PARALYSIS VIA INTEGRATED SERUM PHARMACOCHEMISTRY AND NETWORK PHARMACOLOGY

Xiaohong Bai^a^, Ruhan A^b,c^, Meng Meng^a^, Biligetu Wang^d^, Sachula Baoyin^e^, Hongyan Sheng^f^, Tegexibaiyin Wang^b,c^, Lan Wu^g^

^*a*^ *College of Mongolian Medical, Inner Mongolia Medical University, China,* ^*b*^ *Affiliated Hospital, Inner Mongolia Minzu University, China,* ^*c*^ *Laboratory of Medical Research and Innovation Center, Affiliated Hospital of Inner Mongolia Minzu University, China,* ^*d*^ *College of Traditional Chinese Medicine, Beijing University of Chinese Medicine, China,* ^*e*^ *Mongolian Medicine Research Laboratory, Xilingol League Mongolian Medicine Hospital,* ^*f*^ *College of Mongolian Medical, Inner Mongolia Minzu University, China,* ^*g*^ *Inner Mongolia Collaborative Innovation Center for Mongolian Medicine, Inner Mongolia Medical University, China.*

*XB and RA contributed equally to this study.*

**Background:** Peripheral facial paralysis (PFP), a common neurological disorder with an annual incidence of 20-25 cases per 100,000 individuals, results from facial nerve dysfunction and manifests as unilateral facial muscle weakness, impaired eyelid closure, and orofacial asymmetry. While corticosteroids and antivirals remain first-line therapies, their inconsistent clinical outcomes and adverse effects have spurred interest in alternative approaches. Among these, the traditional Mongolian Medicine Maqianzi-2 (MM-2) has demonstrated clinical benefits in PFP management, though its pharmacological mechanisms remain poorly characterized. This study employed an integrated metabolomics and network analysis approach to systematically identify bioactive components of MM-2 and elucidate its therapeutic mechanisms against PFP.

**Subjects and Methods:** Untargeted metabolomics was conducted on serum samples from MM-2-treated rats using UHPLC-QE-MS to identify systemically absorbed compounds. Potential targets of these compounds were predicted via Swiss Target Prediction and cross-referenced with PFP-related targets from the DisGeNET, GeneCards, and OMIM databases. Protein-protein interaction (PPI) networks were constructed using STRING and further analyzed with Cytoscape (v3.9.1) for network visualization, topology analysis, and hub gene identification. Subsequent GO/KEGG enrichment analysis of the refined network elucidated key biological processes and pathways. Molecular docking simulations (AutoDock Vina) validated interactions between key active ingredients (quinic acid and caffeic acid) and critical targets (NF-κB and TLR4), with binding affinities visualized using PyMOL.

**Results:** In this study, we employed untargeted metabolomics analysis to identify 16 active components from MM-2 and 1,231 potential targets associated with PFP, including NF-κB, TLR4, IκBα, IL-1β, IL-6, and TNF-α. Further intersection analysis allowed us to pinpoint 21 potential targets for MM-2 in the treatment of PFP. Our findings suggest that quinic acid and caffeic acid may be key active ingredients in MM-2. Additionally, our enrichment analysis indicated that the PI3K-Akt signaling pathway, TNF signaling pathway, Toll-like receptor signaling pathway may all be related to the therapeutic effects of MM-2. The study also highlighted the strong binding affinity between these key active ingredients (quinic acid and caffeic acid) and key targets (NF-κB and TLR4), providing crucial insights into the molecular mechanisms by which MM-2 treats PFP.

**Conclusions:** MM-2 exerts therapeutic effects against PFP via a multi-compound, multi-target, and multi-pathway mechanism. The primary bioactive ingredients, quinic acid and caffeic acid, modulate critical signaling pathways—including the PI3K/Akt, TNF, and Toll-like receptor pathways—by targeting key nodes such as NF-κB, TLR4, IκBα, IL-1β, IL-6, and TNF-α. These interactions synergistically suppress neuroinflammation, enhance neuronal survival, and restore facial nerve function, underscoring MM-2’s potential as a holistic therapeutic strategy for PFP.

**Acknowledgments**

This research was supported by the Project for Pharmacodynamic Evaluation and Molecular Mechanism Research of Mongolian Medicine Compound Preparations (No. 2017-009), the Inner Mongolia Grassland Talent Program, the Qi Huang Scholars Support Project (2021), and the Mongolian Medical Doctoral Research Program (No. MYYXTBS202306) from the Inner Mongolia Collaborative Innovation Center for Mongolian Medicine.

## PHHS25064: PSYCHOLOGICAL NEEDS ENDORSEMENT OF DIGITAL AND INTELLIGENT TRANSFORMATION: A CATALYST FOR DISRUPTIVE INNOVATION IN SPECIALIZED, REFINED, DISTINCTIVE, AND INNOVATIVE SMES

Sen Lu^a^, Yiwen Chen^a^

^*a*^ *Business College, Nanjing Normal University, Nanjing, China.*

*SL and YC contributed equally to this research.*

**Background:** In the context of the rapid expansion of the global economy, SMEs that are specialized, refined, distinctive, and innovative—serving as a critical component in China’s economic transformation and enhancement—are confronted with challenges related to insufficient innovation and constrained technology. To address these challenges, it is imperative for companies to overcome the psychological inertia within their organizations that frequently hinders disruptive innovation. Concurrently, enterprises must cultivate the capacity for self-reinvention and have the courage to discontinue or enhance existing products or services to introduce innovations that have the potential to disrupt the market. Digital and intelligent transformation is a pivotal strategy in the contemporary era, offering novel approaches for organizations to overcome development constraints and actualize their disruptive innovation intentions. Moreover, enterprises must address psychological challenges, such as trepidation regarding new technologies and resistance to change. To this end, companies must prioritize employees’ mental health and engagement through training and culture-building to foster continuous innovation and development.

**Subjects and Methods:** The present study develops a negative binomial regression model with two-way fixed effects grounded in the theoretical examination of the internal mechanisms of digital and intelligent transformation affecting the disruptive innovation of specialized, refined, distinctive, and innovative SMEs. It methodically analyzes the influence of digital and intelligent transformation on disruptive innovation within enterprises and on the mental health of employees.

**Results:** The research indicates that digital and intelligent transformation can markedly enhance the disruptive innovation capabilities of specialized, refined, differentiated, and innovative SMEs. The finding demonstrates robustness following the substitution of the independent variable measurement method, the dependent variable measurement method, and the PSM test, with this effect being more pronounced in firms with more favorable geographical locations and younger management teams. This is related to the fact that the industrial structure in the eastern region is more developed, and the psychological needs of younger managers are more inclined to accept innovation. The mechanism test indicates that digital and intelligent transformation favorably influences disruptive innovation by fostering technical advancement and enhancing dynamic capacities within enterprises.

**Conclusions:** The study’s findings provide critical insights on leveraging digital and intelligent transformation to enhance the disruptive innovation capabilities of specialized, refined, distinctive, and innovative SMEs. This is significant for advancing China’s economy towards a higher quality development stage. The support of national policies, particularly the empowerment of specialized, special, and new SMEs in the eastern region, aims to maximize their regional advantages. This, in turn, will drive enterprises in the western and central regions to achieve digital and intelligent transformation and disruptive innovation. From a psychological standpoint, the initiative emphasizes nurturing the psychological readiness of senior managers to embrace innovation. This is facilitated by the implementation of a mentorship framework within the enterprise, which fosters the development of junior managers and fosters knowledge and expertise transfer across generations. These measures are designed to enhance the acceptance of digital and intelligent transformation among senior managers, while concurrently providing a platform for junior managers to evolve and flourish, thereby collectively propelling enterprises in the digital era.

**Acknowledgments**

This project is supported by the National College Student Innovation and Entrepreneurship Training Program (Project No. 202410319032Z).

## PHHS25065: FRAMEWORK FOR DIGITAL TRUST: MITIGATING CHALLENGES OF DATA DISTRUST

Linrui Han^a,b^, Ri Zheng^a^, Zuwei Du^a,b^

^*a*^ *The Institute for Data Law, China University of Political Science and Law, Beijing 100088, China,* ^*b*^ *The CUPL Data Law Lab, China University of Political Science and Law, Beijing 100088, China.*

*LH and RZ contributed equally to this study.*

**Background:** The rapid acceleration of digital transformation has exposed traditional trust mechanisms as increasingly inadequate for addressing the challenges of a digital society. Data risks, which fuel recurrent trust crises, highlight the need for digital trust frameworks that not only address technological and regulatory gaps but also consider the psychological well-being of individuals. This study explores digital trust through the lens of mental health, focusing on the psychological impacts of data risks and proposing strategies for building digital trust while safeguarding emotional and cognitive well-being.

**Subjects and Methods:** This article employs a multidimensional approach that includes literature review and logical analysis to explore the interrelationship between digital trust and mental health. Beginning with the traditional foundation of trust, the study analyzes how data risks lead to psychological distress, particularly in relation to privacy concerns, loss of autonomy, and emotional discomfort. The research contrasts these emotional impacts with the evolving demands of the digital society, where constant exposure to data risks amplifies the psychological toll. The article synthesizes various definitions of digital trust, framing it not only as a technical and regulatory necessity but also as a means of alleviating the emotional burden on individuals. The challenges in constructing digital trust are explored across technological, regulatory, and psychological dimensions, with particular attention to the mental health implications of each.

**Results:** The study finds that the breakdown of traditional trust patterns in the digital era is not only a technical and regulatory issue but also a profound psychological challenge. Technologically, biases in data collection, processing, and algorithmic training contribute to a sense of insecurity and anxiety among users. Regulatory gaps, such as inadequate data protection laws, exacerbate feelings of vulnerability and mistrust. Psychologically, individuals are deeply affected by data risks, leading to stress, fear, and uncertainty about their privacy and identity. The mental health effects of these risks are seen in the erosion of confidence, heightened anxiety over data misuse, and emotional distress related to perceived loss of control over personal information.

**Conclusions:** To build digital trust in a way that supports both technological progress and mental well-being, it is essential to address psychological concerns. At the technical level, improving data protection through transparent practices and trustworthy technologies can alleviate emotional distress. Normatively, the implementation of robust data security laws and clearer definitions of AI responsibility can reduce anxiety about privacy violations. Psychologically, fostering digital literacy and providing support for individuals to cope with the emotional toll of data risks are critical strategies for building a resilient digital trust framework. Ensuring the mental health of individuals within the digital ecosystem is a foundational aspect of creating a trustworthy and sustainable digital society.

**Acknowledgements**

This work was supported by the National Key R&D Program of China (Grant No.2022YFC3321001), the Natural Science Foundation of China (Grant No. J2424005).

## PHHS25066: AN IMPROVED COMPREHENSIVE SCREENING TECHNIQUE FOR MAIN INFLUENCING FACTORS OF LIFE CYCLE COST IN MENTAL HEALTH EQUIPMENT SYSTEMS

Yidan Li^a^, Juqin Fan^b^, Guowei Chen^a^, Chuyan Cao^a^, Jin Pang^a^

^*a*^ *Department of Management Engineering and Equipment Economics, Naval University of Engineering, 430033, Wuhan, China,* ^*b*^ *Teaching and Research Support Center, Naval University of Engineering, 430033, Wuhan, China.*

*YL and JF contributed equally to this study.*

**Background:** In the domain of mental health services, the reliability and sustainability of specialized equipment play a critical role in ensuring continuous care. However, the estimation of Life Cycle Cost (LCC) for mental health equipment is often hindered by an excessive number of influencing factors. This not only increases the difficulty and uncertainty of LCC estimation models but also places an additional burden on data collection efforts. To address these challenges, this study proposes an improved comprehensive screening technique aimed at identifying the primary cost-driving factors in the life cycle of mental health-related equipment, such as therapeutic monitoring systems or diagnostic tools.

**Subjects and Methods:** The improved screening technique integrates multiple existing screening methods to form a more robust and balanced approach. Rather than relying on a single method, which may yield incomplete or biased results, this technique synthesizes their strengths while offsetting individual limitations. Particular emphasis is placed on correcting for multicollinearity issues commonly encountered in stepwise regression analyses. This methodological innovation ensures that the main influencing factors are not only statistically significant but also practically relevant to the mental health field.

**Results:** By applying the improved technique to the LCC analysis of mental health support equipment—specifically those used in long-term psychological monitoring and intervention—the main cost-influencing factors were effectively identified. When compared with traditional single-method and previously proposed comprehensive methods, the improved approach demonstrated enhanced feasibility and reliability. This improvement is especially significant in mental health contexts, where equipment downtime or cost misestimation can directly affect patient outcomes and institutional trust.

**Conclusions:** This paper introduces a refined comprehensive screening approach that is highly suitable for analyzing life cycle costs within mental health equipment systems. It addresses the critical issue of factor redundancy and omission while resolving statistical pitfalls such as multicollinearity. Importantly, it alleviates psychological concerns among stakeholders regarding the reliability of LCC estimations. The technique has been successfully validated through practical application in mental health care environments, delivering results with high credibility. Its adoption promises to improve decision-making confidence and facilitate smoother progress in subsequent mental health planning and resource allocation efforts.

**Acknowledgments**

This work was supported by the Military Science of National Social Science Foundation (2023-SKJJ-B-029), the Independent Research and Development Project by Naval University of Engineering (202350A040).

## PHHS25067: DYNAMIC IMPACT OF URBAN SHRINK HETEROGENEITY ON URBAN EFFICIENCY IN THE YELLOW RIVER BASIN: THE MEDIATION ROLE OF MENTAL HEALTH

Xiaoyu Jin^a,b^, Zhan Gao^a,b^

^*a*^ *School of Business, Henan University of Engineering, Zhengzhou 451191, Henan, China,* ^*b*^ *Research Center for the High-Quality Economic and Social Development of the Central Plains, HAUE, Zhengzhou 451191, Henan, China.*

**Background:** Through some literature reading, we know that the heterogeneity of urban shrinkage in the Yellow River Basin has different impacts on urban efficiency. Therefore, this paper analyzes the difference of the impact of urban shrinkage at different levels on urban efficiency, and summarizes the mediating role of mental health between urban shrinkage and urban efficiency.

**Subjects and Methods:** The BCC model of DEA was selected to calculate the efficiency of shrinking cities. Select the data of cities in the Yellow River Basin from 2006 to 2018, and empirically analyze the impact of urban shrink of different scales on urban efficiency by establishing VAR model. And it further confirms the mediating role of mental health.

**Results:** First, urban shrink is an important factor affecting urban efficiency. The contribution degree of urban shrinkage to urban efficiency exceeds 78% in megacities, medium-sized cities and small cities, and the contribution proportion in large cities exceeds 60%. Second, the lower the urban hierarchy is, the stronger the impact of urban shrinkage on urban efficiency in the short term will be. In the short term, one unit of urban shrinkage will lead to a 0.003436-unit decrease in the efficiency of megacities, a 0.027624-unit change in the efficiency of large cities, a 0.028921-unit decrease in the efficiency of medium-sized cities, and a 0.095701-unit decrease in the efficiency of small cities. The continuous impact of urban shrinkage on urban efficiency tends to slow down in the long run. For megacities, large cities and small cities, the impact of urban shrinkage on urban efficiency basically drops to zero in the 10th period. Finally, mental health plays a mediation role through aspects such as labor supply, innovation activities, social capital, social participation, and public services, influencing cities of different scale grades and causing differences in the impact, volatility, and persistence of urban shrinkage on urban efficiency.

**Conclusions:** Mental health is an important mediator variable in the impact of urban shrinkage on urban efficiency. Paying attention to the mental health of residents in cities of different levels will help shrinking cities improve urban efficiency.

**Acknowledgments**

This work was supported by a project grant from 20FJYB009 (Research on Heterogeneity of Efficiency, Influencing Factors, and System Construction for Improvement in Shrinking Cities along the Yellow River Basin), 2024-YYZD-09 (Research on Spatial Heterogeneity of Urban Shrinkage in the Yellow River Basin and High-Quality Development Paths for New Urbanization).

## PHHS25068: CRYOTHERAPY COMBINATION THERAPY FOR THE TREATMENT OF ANKLE INJURIES IN AEROBICS ATHLETES

Jian Li^a^, Xin Li^b^, Hui Wang^c^, Chuan He^d^, Puzhu Han^e^, Chengpeng Dong^a^

^*a*^ *Sport Institute, Liaoning Institute of Science and Engineering, Jinzhou 121000, China,* ^*b*^ *Basic Teaching Department, Luxun Academy of Fine Arts, Shenyang 110000, China,* ^*c*^ *First Center Hospital of Tianjin, Tianjin 300380, China,* ^*d*^ *Health Sciences of Kobe University Graduate School, Kobe 650-0034, Japan,* ^*e*^ *Arts and Science College, Hongkong City University, Hongkong 999077, China.*

**Background:** Ankle injuries are prevalent among aerobics athletes due to the nature of their sport, which involves frequent jumps, twists, and landings. Traditional treatment methods for ankle injuries in aerobics athletes, such as simple rest, ice compress, and general rehabilitation exercises, have certain limitations. They may not fully address the complex biomechanical issues and muscle imbalances caused by these injuries. There is an urgent need to explore more effective treatment approaches to help athletes recover quickly and reduce the risk of recurrence. This study aimed to address this gap by investigating the impact of the ice combination therapy on ankle injuries in aerobics athletes.

**Subjects and Methods:** The subjects of this study were aerobics athletes who had suffered ankle injuries. The research combined cryotherapy with targeted muscle strength training. Based on the anatomical and physiological characteristics of the ankle joint and its injury mechanisms, a comprehensive treatment plan was developed. Cryotherapy was applied to reduce inflammation and swelling in the acute stage of the injury. It was administered at specific intervals and for a set duration. Meanwhile, targeted muscle strength training was designed to enhance the strength and stability of the ankle joint muscles, aiming to improve the overall function of the ankle. Exercises included resistance band training for the peroneal and tibialis muscles, as well as balance - training on unstable surfaces. The treatment process was carefully monitored, and relevant data such as pain levels, range of motion, and muscle strength were collected for further analysis.

**Results:** The results showed that the ice combination therapy had a significant positive impact on the recovery of ankle injuries in aerobics athletes. After a certain period of treatment, the athletes’ ankle pain was significantly reduced, with a decrease of up to 50% in the pain scale scores. Their joint range of motion was improved, with an average increase of 20% in both dorsiflexion and plantarflexion. Moreover, the muscle strength around the ankle joint was enhanced, which effectively improved the stability of the ankle. The stability improvement was demonstrated by a significant reduction in the number of ankle-related instability incidents during training.

**Conclusions:** In conclusion, this study provides a new and effective treatment method for ankle injuries in aerobics athletes. The combination of cryotherapy and targeted muscle strength training can accelerate the recovery process of injured ankles, improve the athletic performance of athletes, and reduce the likelihood of secondary injuries. It is hoped that this research can be widely applied in the field of sports medicine to benefit more athletes. This approach could potentially be extended to other sports that are prone to ankle injuries, contributing to the overall well-being and performance of athletes across different disciplines.

## PHHS25069: EXPLORATION OF THE TRADITIONAL VILLAGE ENVIRONMENT CREATION CONCEPT AND PERFORMANCE UNDER THE INFLUENCE OF REGIONAL PRECIPITATION METEOROLOGY ON THE PSYCHOLOGICAL BEHAVIOR OF THE QIANG ETHNIC GROUP

Zhizheng Liu^a^, Jun Zheng^b^

^*a*^ *Southeast University, Nanjing 210096, Jiangsu, China,* ^*b*^ *Southwest Minzu University, Chengdu 610041, Sichuan, China.*

**Background:** The biopsychological dynamics influenced by the natural environment and their corresponding behavioral outcomes represent the crystallization of human thought and spirit. The meteorological characteristics in the Qiang ethnic region of China exhibit a vertical variation, with precipitation being a major factor affecting the psychology and actions of the Qiang people. By discussing the annual average precipitation in relation to key aspects of the living environment in the Qiang region and analyzing the precipitation factors in the microclimate of the area, it is revealed that meteorological factors have created unique expressions of their psychology and behavior, directly impacting the environment of their living spaces.

**Subjects and Methods:** Currently, there is limited research on the psychological dynamics of people in the Qiang ethnic region of China based on precipitation environments, and even less from a medical perspective on architecture. This study explores design concepts and manifestations of traditional Qiang villages from a psychological behavior perspective related to regional precipitation meteorology. Specifically, methods like site measurement, literature investigation, graphical representation, and inferential simulation will analyze local people’s daily behavior patterns and psychological representations regarding precipitation factors of the microclimate at Qiang architecture sites. The research aims to infer the environmental context and investigate passive adaptation and morphological presentation of this ethnic group’s living environment.

**Results:** This study explores two outcomes based on meteorological issues and their corresponding psychological representations and behaviors in the Qiang ethnic context. From the perspective of village environment selection, layout, and spatial environment construction, a questionnaire survey was conducted to investigate the psychological reflexes of the Qiang people. The findings reveal various psychological behaviors and ideologies related to precipitation shielding during the construction process. Analyzing the psychological representations associated with building design to shield against rain reveals innovative ideas and practices inherent in their rain protection strategies. By integrating relevant case studies, this research demonstrates how the psychological dynamics of the Qiang people, driven by climate adaptation, have led to the development and successful implementation of their construction concepts. This holds significant scientific value for contemporary understanding of the traditional architectural environment of the Qiang ethnic group in China.

**Conclusions:** Exploring psychology can provide deeper insights into the wisdom of traditional ethnic villages and their construction practices adapted to local conditions. This offers applicable theoretical methods for contemporary architectural design and planning in rural areas of Southwest China, as well as reference for other similar countries and regions around the world.

**Acknowledgements**

This work was supported by a project grant from the 2023 Sichuan Philosophy and Social Sciences Fund Project (Grant No. SCJJ23ND382).

## PHHS25070: RESEARCH ON THE EVALUATION MODEL OF LEARNING PERFORMANCE OF CHINESE VOCATIONAL COLLEGE STUDENTS FROM THE PERSPECTIVE OF LEARNING AND PSYCHOLOGICAL NEEDS: AN EXPLORATION BASED ON GROUNDED THEORY

Lizhi Tao^a,b^, Fei luo^a,b^, Xuyun Peng^a^, Hengliang Cheng^a^, Wentao Sun^a^, Dianwang Pan^a^

^*a*^ *School of Sino-German Intelligent Manufacturing, Shenzhen City Polytechnic, Shenzhen, 518116, China,* ^*b*^ *Faculty of Education, Mahasarakham University, Mahasarakham 44150, Thailand.*

**Background:** With the vigorous development of the global digital economy, there is a significant increase in the demand for high-quality vocational and technical talents. However, Chinese vocational and technical students show a lack of sustained learning motivation, and the existing singular learning performance evaluation methods and inefficient talent cultivation cannot meet the needs of social and economic development. This study aims to explore the evaluation model of learning performance of Chinese vocational college students from the perspective of learning and psychological needs, with the objective of providing theoretical references for enhancing talent cultivation efficiency in vocational colleges.

**Subjects and Methods:** Grounded theory methodology was adopted to conduct an intrinsic logical analysis and model construction of the learning performance evaluation of Chinese vocational college students. Literature analysis was utilized to explore the factors, characteristics, and evaluation dimensions of learning performance, focusing on the influence of learning and psychological needs on evaluation results. Data were collected from two major literature databases, Web of Science and China National Knowledge Infrastructure (CNKI), and analyzed through open coding, axial coding, and selective coding to construct a theoretical framework.

**Results:** The research findings indicate that the strength of learning and psychological needs among Chinese vocational college students significantly influences their learning performance evaluation results. The rational selection of evaluation methods aligned with these needs can enhance collaborative teaching, optimize resources, and improve the efficiency of cultivating technical talents in vocational colleges. Specifically, the study identified three levels of learning needs: basic, intermediate, and advanced, which correspond to different dimensions of learning performance evaluation, including learning behavior, learning achievement, teaching factors, and environmental factors.

**Conclusions:** This study provides a comprehensive exploration of learning and psychological needs in the context of learning performance evaluation. By considering these needs, vocational colleges can develop more effective evaluation models to better meet the demands of talent cultivation. The findings of this study offer valuable theoretical insights for the development of learning performance evaluation models in both Chinese and international vocational colleges. Future research should focus on validating the proposed model through empirical studies and expanding its application to other student groups.

**Acknowledgments**

This work was supported by the Science Foundation of Shenzhen City Polytechnic (grant numbers BS22024007).

## PHHS25071: RESEARCH ON THE INNOVATIVE PRACTICE AND MENTAL HEALTH PROMOTION OF THANGKA MURAL ART IN HIGHER ART EDUCATION

Peng Zhang ^a^

^*a*^ *Sichuan Minzu College, Kangding 626001, Sichuan, China.*

**Background:** Amid global cultural diversity and a revival of traditional art, higher art education faces dual challenges: preserving traditional skills and integrating modern educational methods. Sichuan Nationalities University has leveraged its deep regional cultural heritage to explore innovative models in teaching thangka mural art. Integrating elements of mental health theory, the university seeks to cultivate artistic creativity while bolstering students’ psychological resilience and emotional regulation. Given the creative pressure and emotional fluctuations encountered in art learning, art therapy and behavioral interventions have been employed to inspire creativity and foster a positive psychological state, infusing contemporary vitality into traditional culture.

**Subjects and Methods:** This study examines the thangka mural teaching practices at Sichuan Nationalities University through a combination of case analysis and comparative research. Two innovative teaching models are explored. First, the studio-style teaching model optimizes classroom space and faculty resources by integrating psychological counseling and behavioral observation. Group discussions and case tracking help alleviate creative anxiety and enhance self-expression and emotional management. Second, the project-driven teaching model, developed in collaboration with cultural institutions and enterprises, facilitates industry-education integration. Through practical projects, this model employs a behavioral science evaluation system to monitor students’ emotional fluctuations and team dynamics, leading to improvements in both artistic skills and psychological quality.

**Results:** Practical teaching experiences have demonstrated that both models effectively cultivate thangka mural art talent. The studio-style approach not only improves artistic techniques and creative thinking but also fosters higher psychological resilience when facing setbacks. Meanwhile, project-driven teaching enhances practical skills and teamwork, with students reporting significant improvements in emotional management and interpersonal communication. Continuous curriculum development and diverse evaluation methods have supported professional skill growth and contributed positively to behavioral intervention and psychological counseling, although challenges remain in resource integration, technological updates, and international cultural exchanges.

**Conclusions:** This study offers practical experience and theoretical insights for integrating traditional thangka mural art with modern higher art education while incorporating mental health strategies. By employing studio-based and project-driven teaching models, Sichuan Nationalities University has promoted a balanced development of artistic creation and psychological well-being. Future efforts should focus on further integrating interdisciplinary resources, strengthening technical support, and expanding international exchanges to enhance both cultural inheritance and innovative educational practices, while providing new avenues for mental health support in art education.

**Acknowledgements**

Funded by the Sichuan Provincial Department of Education’s 2024-2026 Sichuan Higher Education Talent Training Quality and Teaching Reform Project: Research on the Dynamic Optimization Path of Industry-Education Integration of Thangka Mural Courses Driven by Industry Iteration from the Perspective of New Quality Productivity (General Project), Project No. JG2024-1164, one of the results.

## PHHS25072: ANALYSIS OF THE PSYCHOLOGICAL INFLUENCES OF RCEP TRADE FACILITATION ON MANAGERS OF TRADE EXPORTING ENTERPRISES IN SHANDONG PROVINCE

Qiang Gao^a^, Zihan Niu^a^, Jie Chang^a^

^*a*^ *Shandong Management University, Jinan, China*

**Background:** Since the implementation of RCEP, through tariff concessions, the promotion of trade facilitation and investment facilitation, the trade of goods between members has become closer, and more favorable trade conditions have been created for trade export enterprises. Trade-exporting enterprises have an important position in international economic activities, and the psychological attitudes and demand conditions of their managers affect trade decision-making, cross-border investment cooperation and other aspects, which further affect international economic cooperation and development. This study aims to better understand and satisfy the psychological needs of managers of trade export enterprises, and to better analyze and plan by studying the factors affecting the psychological needs of managers of trade export enterprises by trade facilitation.

**Subjects and Methods:** This paper analyzes the attitudes and psychological needs of managers of trade export enterprises in Shandong Province, takes trade export enterprises in Shandong Province as an example, and establishes a system of indicators of satisfaction of managers of trade export enterprises, and constructs a hierarchical system of indicators of satisfaction of managers of trade export enterprises, which are divided into four dimensions, namely, tariff concessions, utilization of policies, customs facilitation, and trade cooperation. On the basis of SERVQUAL model, we divided them into four dimensions, namely tariff concessions, policy utilization, customs clearance facilitation and trade cooperation, established the index system of psychological needs of managers of trade and export enterprises, constructed the hierarchical analysis method and fuzzy comprehensive analysis and evaluation model (AHP-FCE), and solved the evaluation results by using excel, spss, spsspro, etc.

**Results:** The results of the study show that customs clearance facilitation has the highest weight in the hierarchical analysis method, followed by tariff concessions, policy utilization, and trade cooperation. Based on the weight of the hierarchical analysis method, the fuzzy comprehensive evaluation score is 4.1665 points, and the comparison results in the evaluation of the satisfaction of the psychological needs of the managers of the trade and export enterprises in Shandong Province are satisfactory. Among them, the comprehensive score of tariff concessions is the lowest, and the satisfaction evaluation result of customs clearance facilitation is the highest.

**Conclusions:** In view of the results of this study, this paper suggests that tariff concessions should be maintained to reduce the burden on enterprises and to satisfy the psychological needs of trade and export enterprise managers for tariff concessions. At the same time, the policy promotion should be strengthened, so that all major enterprises, including small and medium-sized enterprises, can eat the dividends of the RCEP preferential policies, and improve the psychological positive attitude of managers towards the RCEP. It should also continue to optimize the customs clearance procedures, improve the efficiency of customs clearance, further improve the investment cooperation mechanism, strengthen the psychological identity of enterprise managers, so as to enhance the effectiveness of trade cooperation.

**Acknowledgments**

Shandong Social Science Planning Fund Program“ RCEP member states trade convenience to the development of the export trade development effectiveness evaluation and improvement path of the export trade of Shandong Province” 24CKFJ18.

## PHHS25073: KNOWLEDGE GRAPH RESEARCH OF DIABETIC RETINOPATHY BASE ON NEO4J

Yuxin Yang^a^, Qinghua Peng^b^, Xiaoxia Xiao^a,c^, Jiangtao Zhangsun^a^, Yang Li^a,c^, Beji Zou^d^

^*a*^ *School of Informatics, Hunan University of Chinese Medicine, Changsha 410208, Hunan, China,* ^*b*^ *School of Chinese Medicine, Hunan University of Chinese Medicine, Changsha 410208, Hunan, China,* ^*c*^ *AI TCM Lab Hunan, Hunan University of Chinese Medicine, Changsha 410208, Hunan, China,* ^*d*^ *School of Computer Science and Engineering, Central South University, Changsha 410083, Hunan, China.*

**Background:** This research employs Neo4j to amalgamate data from various sources in order to develop a knowledge graph pertaining to Diabetic Retinopathy (DR) in clinical practice. The objective is to improve the utility of the knowledge graph and to facilitate intelligent diagnosis and treatment options.

**Subjects and Methods:** This research employs the diagnostic and therapeutic principles of traditional Chinese medicine in the treatment of diabetic retinopathy (DR). It utilizes methodologies for evidence identification and analyzes a dataset consisting of 725 clinical records to extract relevant information. The study adopts an inductive-deductive framework to elucidate the relationship between knowledge and employs Cypher statements for knowledge querying and visualization purposes. Additionally, the research investigates the correlation between syndrome elements and the characteristics of fundus images through the application of a centrality algorithm. Knowledge reasoning is further enhanced by integrating community discovery techniques with similarity algorithms.

**Results:** The constructed knowledge graph consisted of 360 nodes and 1,330 relationships, which included 53 nodes of syndrome factors and 42 nodes related to the characteristics of fundus images. Utilizing the PageRank algorithm to assess the correlation between the features of fundus images and the syndrome factors, it was determined that diabetic retinopathy (DR) is primarily associated with deficiencies in qi and yin deficiency syndrome factors, with the implicated organs being the kidney, spleen, and liver. Further investigation into the relationship between discriminatory typing and diagnostic grading revealed that the association of yin deficiency syndrome with internal heat and mild-to-moderate non-proliferative diabetic retinopathy (NPDR) indicates that this stage of NPDR is also characterized by syndrome types such as qi and yin deficiency syndrome, liver and kidney deficiency syndrome, and yin deficiency with blood stasis syndrome. The knowledge predictions derived from the knowledge graph regarding diabetic retinopathy provided supporting evidence that aligns with clinical outcomes, thereby validating the methodology employed in this study.

**Conclusions:** The incorporation of multi-source data utilizing Neo4j is able to enhance the structural organization of the knowledge graph, thereby significantly increasing its applicability. This integration facilitates the synthesis and identification of patterns related to diagnosis and treatment, supports intelligent assistance in the diagnosis of diabetic retinopathy, and offers innovative approaches and methodologies for clinical research.

**Acknowledgements**

This work was supported by Hunan Provincial Administration of Traditional Chinese Medicine Key Project (A2023048); Hunan Provincial Department of Education Key Project of Scientific Research (23A0273); Hunan Provincial Natural Science Foundation Youth Fund (2024JJ6338); Changsha Natural Science Foundation (kq2402173); National Natural Science Foundation of China Youth Fund (62402180).

## PHHS25074: APPLICATION OF A NEW THREE IN ONE DEFOGGER FOR DEEP DUST REMOVAL IN DESULFURIZATION TOWERS OF THERMAL POWER PLANTS

Ting Wang^a^, Dan Sun^a^, Changwei An^a^, Tong Liu^a^, Te Wang^a^

^*a*^ *School of Biomedical and Chemical Engineering, Liaoning Institute of Science and Technology, Benxi, Liaoning, China.*

**Background:** Environmental sanitation is an important component of public health, which directly affects people’s health status. With the rapid development of industry, air pollution caused by emissions has become increasingly serious. With the gradual improvement of national requirements for environmental protection and air pollution control, traditional dust removal technologies are no longer able to meet current environmental standards. Therefore, the development of new and efficient dust removal equipment is particularly important. As one of the main sources of air pollution, the improvement and innovation of desulfurization tower dust removal technology in thermal power plants play a crucial role in improving the atmospheric environment and safeguarding public health. The emergence of the new three in one demister is a positive response and technological breakthrough to this demand. By using the emerging three in one demister to enhance the comprehensive treatment capability of flue gas dust removal systems, it will become an important part of safeguarding public health and safety.

**Subjects and Methods:** A comparative and comprehensive analysis is conducted on the advantages and disadvantages of ordinary folding plate defoggers, bundle type dust removal defoggers, and ridge type defoggers in the current market. A new type of three in one defogger is proposed, which achieves the highest efficiency of defogging through the initial defogging of the defogger plate, the secondary defogging of the defogging chamber, and the tertiary defogging of the top defogger plate, effectively reducing the height of the defogger and lowering various cost expenditures. This article focuses on a comprehensive explanation of its simple model structure, the working principle of three-stage defogging, and its main characteristics of high efficiency, stability, and low cost.

**Results:** The new three in one defogger is efficient, stable and reliable, occupies a small space, has low installation and maintenance costs, and has a low overall cost. However, installation may cause a slight increase in equipment operating resistance, but the value is not very significant and will not have a significant impact on the final emission results. The high efficiency is mainly reflected in its unique three-stage defogging design, which ensures the maximization of dust removal effect through a layer by layer progressive defogging method. Stability and reliability stem from its sturdy and durable materials and operational requirements, ensuring excellent performance even in harsh working environments. The characteristic of occupying small space is due to its highly integrated optimized design, which makes the layout of equipment in the desulfurization tower more compact and effectively improves space utilization. The low installation and maintenance cost reduces unnecessary maintenance steps, lowers labor and time costs, and is a significant advantage of the new three in one defogger.

**Conclusions:** The three in one defogger, with its excellent performance and low cost, will undoubtedly become the preferred choice in the defogger industry and a strong defender of public health safety.

**Acknowledgments**

2025 Liaoning University of Science and Technology College Student Innovation and Entrepreneurship Training Program Project, Research on Dust Reduction Measures in Desulfurization Tower (202511430147).

## PHHS25075: CAMPUS REHABILITATION LANDSCAPE DESIGN BASED ON MENTAL HEALTH INTERVENTIONS FOR COLLEGE STUDENTS

Zhiqin Zhang^a^, Wenhui Xie^a^

^*a*^ *Wuxi City College of Vocational Technology, Wuxi 214153, Jiangsu, China.*

**Objective:** Contemporary college students as an important group of national future construction, its mental health education is an important social issue, campus rehabilitation landscape construction can effectively promote the mental health of college students.

**Subjects and Methods:** This study firstly designs a questionnaire based on the theory of rehabilitation landscape design to investigate the preference of mental sub-healthy college students for campus rehabilitation landscape design, and then takes the green space of the campus of Wuxi City College of Vocational Technology, Jiangsu Province, as a design site to practically explore campus rehabilitation landscape design based on the intervention of college students’ mental health.

**Results:** With a total area of 6492 m^2^, combining the theory of psychological rehabilitation and the process of psychological counselling practice, and taking five-sense healing. As the basic design concept, the general layout adopts a soft curvilinear composition. Creating a ‘closed-loop unidirectional’ tour route with multiple landscape nodes. They include the Entrance Area, the Self-healing Area, the Psychological Counseling Area, the Reconciliation Garden Area and the Exit Area, which has the wide, serene, cozy, vigorous characteristic, respectively. Basing considerate design, the Entrance Area combines the current terrain to set up a flowing step space to guide mental sub-healthy college students to enter the landscape space; the Self-healing Area provides them with self-knowledge and primary healing through two landscape nodes, the Murmuring Cottage and the Meditation Garden; the Psychological Counseling Area provides them with outdoor psychological counselling space with three main spaces: Group Counselling, One-to-many Counselling and One-to-one Counselling, where mental sub-healthy college students can get help from professional counsellor to sort out psychological barriers and obtain deep-level psychological rehabilitation; the Reconciliation Garden Area includes three landscape nodes: the Flower Wishing Pool, the Sparse Forest Meadow, and the ‘Original Happiness’ Garden, where mental sub-healthy college students can make wishes, chat freely, and gain friendship and freedom after regaining their mental health through the Self-healing Area, the Psychological Counseling Area. This landscape design process also plant configuration to create a healing landscape for the university campus.

**Conclusions:** This study is expected to provide implications for campus rehabilitation landscape design practices in the field of college student mental, thus providing a new possible path for college student mental health interventions.

**Acknowledgements**

This work was supported by a project grant from 2024 Wuxi Philosophy and Social Sciences Bidding Projects (Special Program for Social Education Development) (Grant No. WXSK24-JY-A04).

## PHHS25076: THE MULTI-LABEL TEXT CLASSIFICATION OF TELECOMMUNICATION FRAUD: INCORPORATING PSYCHOLOGICAL FACTORS FOR ENHANCED DETECTION

Lin Wu^a^, Qi Dong^a^, Ying Xu^a^, Shiqi Wang^a^

^*a*^ *School of Computer and Cyberspace Security, Communication University of China, Beijing 100024, China.*

**Background:** In the digital age, telecommunication fraud is rampant, causing not only heavy financial losses but also severe impacts on individuals’ mental health. Victims usually experience increased anxiety and a sense of violation, highlighting the urgent need for more effective fraud detection methods. Existing research on fraud - related text classification faces challenges like class imbalance and semantic overlap, while the psychological aspects of fraud, such as fraudsters’ emotional manipulation and victims’ psychological reactions, remain under - explored. This study aims to enhance multi - label text classification of telecommunication fraud texts by integrating psychological factors.

**Subjects and Methods:** We propose two innovative methods. First, a semantic - enhanced word segmentation method is developed. By integrating TextRank, TF - IDF, and Word2Vec, a keyword library is constructed and incorporated into the WordPiece segmentation process. This method adjusts keyword weights according to label distribution and extracts more keywords through Word2Vec training, improving segmentation accuracy. Second, a keyword - weighted BERT - BiLSTM - TextCNN model is designed. It combines BiLSTM and TextCNN networks with BERT, strengthening the processing of temporal and local text features. A keyword attention layer is added to optimize classification. In addition, to better account for the psychological factors, we incorporate psychological feature extraction techniques. For example, we analyze language patterns related to emotional manipulation and psychological stress in the fraud texts, and these features are integrated into the model training process to improve the understanding of the psychological aspects of fraud.

**Results:** Experiments on a real - world telecommunication fraud case dataset with about 114,000 entries in 14 categories show that our methods are effective. The semantic - enhanced word segmentation method addresses class imbalance and semantic overlap, enhancing the model’s understanding. The keyword - weighted model further improves classification accuracy compared to baseline models. From a psychological perspective, the improved accuracy enables better identification of fraud tactics exploiting emotional vulnerabilities, which helps in both fraud detection and alleviating victims’ psychological harm through faster intervention.

**Conclusions:** This research contributes significantly to telecommunication fraud detection. By integrating psychological factors into text classification, it enhances the model’s ability to recognize diverse fraud tactics. The proposed methods offer insights for improving fraud detection systems and could be applied to other text classification tasks. Considering psychological aspects emphasizes the importance of addressing fraud’s mental health impacts. Future research should focus on expanding the dataset, optimizing models, and evaluating real - world performance to enhance fraud prevention and victim support.

## PHHS25077: FOSTERING A POSITIVE MINDSET: REFLECTIONS ON THE INTEGRATED DEVELOPMENT OF CAREER EDUCATION

Yingying Cen^a^, Zhihua Zhang^a^

^*a*^ *Zhejiang International Studies University, Hangzhou, Zhejiang, China.*

**Background:** As globalization accelerates, the younger generation faces a future filled with both immense opportunities and significant challenges. In this context, universities must adopt a systematic approach to support students’ psychological health and career development. This is crucial as the rapidly changing social landscape demands that students possess not only the knowledge and skills required for professional success but also the psychological resilience to navigate the complexities of life. Developing students’ mental well-being alongside their career skills ensures they are well-equipped to thrive in the face of evolving societal challenges.

**Subjects and Methods:** Psychological health education is increasingly linked to career development. Therefore, it is necessary to explore localized theories and practical applications to better serve students. This study employs historical review and philosophical analysis to examine the origins and evolution of Western career education theories, along with the challenges they currently face. The paper also investigates the state of psychological health and career education in China, recognizing the need for a career education framework that aligns with Chinese cultural characteristics. This framework should incorporate both career skills and psychological well-being to foster holistic student development.

**Results:** The study identifies several key issues in the current approach to career education. First, there is a disconnection between theory and practice, leading to ineffective educational methods. Second, the gap between what is offered in career education programs and students’ actual needs has hindered the impact of these programs. Third, career education is often separated from moral and ideological education, which limits its potential to address students’ psychological health needs. These challenges have led to insufficient support for students’ psychological well-being, negatively impacting their career planning and adaptability to stressors in both their professional and personal lives.

**Conclusions:** To better support students’ psychological health and career development, career education must be integrated with moral and psychological education. This integration would foster a career education model that emphasizes “integration, guidance, collaboration, and promotion,” ultimately helping students build a positive mindset. By encouraging steady, well-rounded development, this model enhances students’ psychological resilience and prepares them for future success. By addressing both career skills and psychological well-being in a balanced manner, universities can provide a strong foundation for students’ growth and ensure they are well-prepared for the challenges and opportunities that lie ahead.

**Acknowledgement**

Supported by Scientific Research Fund of Zhejiang Provincial Education Department (Project Number: Y202353841).

## PHHS25078: RESEARCH ON PSYCHOLOGICAL CRISIS MANAGEMENT OF VOCATIONAL COLLEGE STUDENTS UNDER THE NEW SITUATION

Zemin Chen^a^, Delong Tong^b^, Yan Zhu^a^, Xiaoke Yin^a^, Bo Hu^c^, Ting Pan^d^

^*a*^ *Hunan Biological and Electromechanical Polytechnic, Changsha 410127, China,* ^*b*^ *Fushun Vocational Technical Institute, Fushun 113122, China,* ^*c*^ *Beiya Middle School of Changsha, Changsha 410005, China,* ^*d*^ *Hunan Dizhi Middle School, Changsha 410000, China.*

**Background:** In the new situation, China’s psychological crisis management system for vocational college students is improving, forming a three-dimensional work network covering schools, departments, and students. The School Mental Health Education Center leads and coordinates crisis management; departments manage students’ psychological crises as intermediate management; students actively participate in and support mental health management. Departments bridge school policies and students’ mental health.

In recent years, vocational colleges have explored students’ psychological crisis management and achieved results in institutional construction, teacher training, prevention, intervention, and follow-up support. However, as a secondary management unit, departments have shortcomings in specific systems and implementation, needing further improvement. Departments, as direct managers and educators, are ultimate practitioners and promoters of psychological crisis management. Their roles include popularizing mental health knowledge, conducting educational prevention, discovering and identifying problems, making referrals, and assisting in intervention. Strengthening research on departmental psychological crisis management is crucial for improving vocational colleges’ overall management and ensuring students’ health.

**Subjects and Methods:** This study focuses on psychological crisis management in vocational colleges and departments. By analyzing existing research and combining it with the actual situation, the current situation in departments is deeply compared and analyzed. Methods like questionnaires, interviews, and case analysis collect first-hand information to identify weak links, providing an empirical basis for targeted countermeasures.

**Results:** Problems in vocational colleges’ psychological crisis management include: an insufficient prevention system lacking systematicity and scientificity; an imperfect intervention mechanism with emergency response needing improvement; an unsmooth working mechanism with coordination obstacles among departments; and a loose work network with underutilized linkage effects between departments.

To address these issues, this study proposes: establishing a scientific and comprehensive prevention system, enhancing students’ psychological quality and coping ability through courses, lectures, and activities; establishing a sound crisis intervention mechanism with detailed procedures and plans, focusing on sudden death events; improving departmental mechanisms, including a responsibility system for heads, improving the system for counselors and class psychological committee members, and strengthening team building and training; building a department-led psychological crisis management network, strengthening cooperation with departments like Academic Affairs, Student Affairs, Security, and Logistics to form a joint force.

**Conclusions:** This study aims to establish a comprehensive, goal-oriented, and responsibility-oriented psychological crisis management system for vocational colleges and departments, achieving effective prevention, timely intervention, and recovery management. By strengthening prevention systems, improving intervention mechanisms, perfecting work mechanisms, and building a close work network, the professionalism, scientificity, and effectiveness of departmental psychological crisis management can be enhanced. This improves vocational colleges’ management, enhances teachers’ and students’ coping abilities, ensures school harmony and student health, and lays a foundation for sustainable development.

## PHHS25079: BEHAVIORAL MECHANISMS IN ORGANIZATIONAL DIGITIZATION: DRIVING GREEN TRANSITIONS FOR SUSTAINABILITY

Wei Peng^a^, Ruoqian Yang^a^, Hong Xiao^a^

^*a*^ *Zhongshan Polytechnic, Zhongshan, China.*

**Background:** In recent years, the digitization of enterprises has gained recognition as a key enabler for improving energy efficiency, advancing carbon neutrality, and fostering business growth. From a behavioral science perspective, this transformation involves not only technological change but also significant shifts in individual and organizational behaviors. These shifts affect decision-making, attitudes toward innovation, risk perceptions, and adaptive responses to new technologies. However, the specific impact of digitization on organizational behavior—especially in relation to sustainability goals—remains underexplored. This study seeks to address this gap by examining how the adoption of digital tools and processes influences organizational behavior, decision-making patterns, and overall effectiveness in pursuing green transitions and sustainability objectives. Understanding these behavioral dynamics is crucial to grasping how digitization drives long-term sustainability and how firms can foster the necessary changes in attitudes and actions within their teams and leadership.

**Subjects and Methods:** This study draws on micro-level data from Chinese listed companies under the “Dual Carbon Target” policy framework. It investigates the complex, behavioral mechanisms linking enterprise digitization to green transition outcomes. By integrating insights from behavioral science and organizational dynamics, the research emphasizes how individual and collective behaviors within firms shape their adoption of green technologies and sustainable practices. Focus is placed on the decision-making processes and adaptive behaviors that influence the organizational response to sustainability imperatives, including risk management, learning, and innovation.

**Results:** Benchmark regression analysis reveals a direct and positive relationship between enterprise digitization and green transitions. Additionally, the mediating effect model identifies key behavioral mechanisms—such as organizational learning, resource reconfiguration, and adaptive capacity—that link digitization to green outcomes. These dynamic capabilities are influenced by individual and team-level behaviors, including cognitive biases, resource allocation decisions, and attitudes toward innovation. Heterogeneity tests across various sample groups indicate that the benefits of digitization are not concentrated in specific sectors, refuting the Green Solow Paradox. Furthermore, the study shows that long-term behavioral shifts, such as changes in organizational culture and decision-making routines, are crucial for sustaining the effects of digitization on sustainability. This suggests that the impact of digitization unfolds gradually, influenced by the pace of behavioral adaptation within organizations.

**Conclusions:** This research contributes to the growing literature on digital technologies and organizational sustainability by offering behavioral science insights into how firms adapt their behaviors and decision-making processes to implement green transition strategies. By identifying the key behavioral dynamics and organizational capabilities that mediate the link between digitization and green outcomes, the study provides valuable guidance for managers and policymakers seeking to accelerate the adoption of energy-efficient practices and drive long-term sustainability goals. Understanding these behavioral mechanisms is critical for crafting strategies that promote not just technological but also behavioral change toward sustainability.

**Acknowledgements**

This research is sponsored by the Innovation Projects of Guangdong Province (grant NO.2023WTSCX268).

## PHHS25080: OPTIMIZATION OF 6G NETWORK ECONOMIC FRAMEWORK DRIVEN BY NONORTHOGONAL MULTIPLE ACCESS TECHNOLOGY AND CYBER-TWIN: APPLICATION OF MENTAL HEALTH MONITORING AND EMOTION REGULATION

Saisai Yan^a^, Maomin Pu^b^, Xuejie Chen^c^

^*a*^ *College of Finance and Economics, Shanghai Lida University, Shanghai, China,* ^*b*^ *Xi’an Technology and Business College, Xi’an, Shaanxi, China,* ^*c*^ *International School of Cultural Tourism, Hangzhou City University, Hangzhou, Zhejiang, China.*

**Background:** With the rapid development of Internet of Things (IoT) and its applications, mobile internet traffic and services are growing at an unprecedented rate. This has brought enormous challenges to the network system, especially with the support of emerging technologies such as 6G (sixth generation mobile communication technology), how to cope with these challenges has become an important issue. Among numerous potential applications, mental health monitoring, emotion regulation, and anxiety/depression management are gradually becoming key application scenarios for 6G networks. Especially the seamless integration of Non-Orthogonal Multiple Access (NOCA) and Cyber-Twin Technology is expected to completely change how healthcare providers monitor and manage mental health.

**Subjects and Methods:** This study proposes a new generation network architecture, Multiple Input Single Output-Non-Orthogonal Multiple Access-Power Consumption-Based User Clustering, MISO-NOMA-PCUC (MISO-NOMA-PCUC), by utilizing NOCA technology. This method includes technical standardization, system optimization, and simulation of 6G mobile communication systems, integrating real-time emotional health monitoring and psychological health management functions. The performance of NOCA in mental health scenarios such as anxiety management and emotion regulation is tested through system level simulation using the Nokia platform.

**Results:** The proposed MISO-NOMA-PCUC model outperforms other access models in terms of Bit Error Rate (BER), channel capacity, and convergence speed. The results indicate that the algorithm provides optimal signal transmission reliability and is particularly suitable for real-time applications in mental health monitoring. The energy efficiency and connection efficiency of the system also demonstrate strong performance, especially in supporting multiple users to access the network simultaneously, which is crucial for large-scale mental health data collection. In addition, the analysis results show that the introduction of Cyber-Twin technology enables the system to provide users with real-time and personalized feedback, effectively regulating emotions and reducing the risk of anxiety and depression attacks.

**Conclusions:** This study develops a powerful and efficient 6G network framework for mental health applications. By combining NOCA with Cyber-Twin technology, the MISO-NOMA-PCUC model demonstrates the potential to support scalable, real-time mental health monitoring solutions. This method not only helps optimize system performance, but also meets the growing demand for mental health services, provides timely emotional intervention, and alleviates anxiety and depression symptoms. Therefore, the optimized 6G network is expected to improve users’ quality of life and overall mental health in an increasingly interconnected world.

## PHHS25081: THE INFLUENCE OF RESPONSIBLE FEMALE LEADERS ON EMPLOYEE INNOVATIVE BEHAVIOR: THE ROLE OF ARTIFICIAL INTELLIGENCE APPLICATIONS AND ARTIFICIAL INTELLIGENCE ANXIETY

Yanan Li^a^, Tao Xie^b^

^*a*^ *School of Economics and Management, Weifang University of Science and Technology, Weifang 25100, Shandong, China,* ^*b*^ *School of Economics, Shenyang University, Shenyang 110000, Liaoning, China.*

**Objective:** Responsible female leaders have shown great potential in driving organizational innovation due to their unique leadership style and values. The main research objective of this paper is to explore the impact of the leadership style of responsible female leaders on employee innovative behavior. In addition, this paper also considers the application of artificial intelligence technology in the process of employee innovation, as well as the artificial intelligence anxiety that arises among the employee population during the application of artificial intelligence technology.

**Subjective and method:** This paper is based on the theories of resource conservation and workplace anxiety, using “leadership style ability behavior outcome” as this paper logic, introducing artificial intelligence applications as mediating variables and artificial intelligence anxiety as moderating variables, and constructing a moderated chain mediation model. This paper analyzes 527 enterprise employee survey questionnaires as effective samples to clarify the impact mechanism of responsible female leaders on employee innovation behavior.

**Result:** Through the analysis of 527 enterprise employee survey questionnaires, this paper found that female leaders have a significant positive impact on employee innovation behavior, which means that the leadership style of responsible female leaders can have a positive effect on promoting employee innovation behavior. Furthermore, the application of artificial intelligence has played a positive mediating role between responsible female leaders and employee innovation behavior, indicating that responsible female leaders can enhance employee innovation behavior by actively applying artificial intelligence technology. Finally, this paper found that employees’ artificial intelligence anxiety negatively moderates the mediating effect of artificial intelligence applications, meaning that the higher the level of employees’ artificial intelligence anxiety, the weaker the mediating effect. This indicates that responsible female leaders should consider employees’ anxiety when using artificial intelligence technology from multiple perspectives, to harness the positive effects of AI technology.

**Conclusion:** This paper conclusion emphasizes the importance of selecting and cultivating responsible female leaders, and points out that the application of artificial intelligence is an important external mechanism for responsible female leaders to influence employee innovation behavior. This means that business managers should face up to the challenges and opportunities brought by the application of artificial intelligence technology to employees, and attach importance to the role of responsible female leaders in the environment of artificial intelligence application. In addition, when applying artificial intelligence technology, enterprises should prioritize employee internal job transfers and retraining to avoid employee anxiety caused by excessive worry, which may affect the effective application of artificial intelligence technology.

**Acknowledgments**

This work was supported by the Key Project of Social Science Planning in Liaoning Province (L23AJL001); Basic Research Project of Higher Education Institutions in Liaoning Province (LJKR0134).

## PHHS25082: THE INFLUENCE MECHANISM OF SPEECH INTONATION ON AUDIENCE’S TRUST AND MENTAL HEALTH PERCEPTION IN NEWS BROADCAST: AN EXPERIMENTAL ANALYSIS BASED ON COGNITIVE PSYCHOLOGY

Wei Wang ^a^

^*a*^ *University of Perpetual Help System Laguna, Manila, Philippines.*

**Background:** The purpose of this study was to explore the mechanism of the influence of voice intonation on audience trust and mental health in news broadcasting. From the perspective of cognitive psychology, this paper studies the direct effects of intonation type and speech speed on trust, and reveals the mediating role of emotional arousal and cognitive load in this process. At the same time, research has focused on the potential role of voice features in regulating emotional management and alleviating mental health indicators such as anxiety and depression. This study attempts to provide suggestions for speech optimization for the media industry, while exploring the indirect effects of news communication on the mental health of the audience.

**Subjects and Methods:** A total of 120 participants, ranging in age from 18 to 50 years old, were recruited, 50%male and 50%female. All participants had normal hearing and good news comprehension. Meanwhile, diverse cultural backgrounds were covered to ensure the universality of the experiment. Participants were randomly assigned to one of four experimental groups, corresponding to a combination of two intonation (monotone versus varied intonation) and two speech speeds (slow versus fast). The study adopted a 2*2 two-factor experimental design, with intonation type (monotone and variable intonation) and speech speed (slow and fast) as independent variables, trust degree (including professionalism, authenticity and overall trust) as dependent variables, and emotional arousal and cognitive load as potential mediating variables.

**Results:** The results demonstrate significant effects of speech intonation and speech speed on trustworthiness, emotional arousal, and cognitive load. Varied intonation and slow speed produced the highest trustworthiness score of 5. 2 and emotional arousal score of 5. 4, while yielding the lowest cognitive load of 2. 8. In contrast, monotone intonation and fast speed resulted in the lowest trustworthiness of 3. 4 and emotional arousal of 3. 2, with the highest cognitive load of 4. 5. ANOVA revealed significant main effects of intonation (F=33. 21, η²=0. 30, p<0. 001)and speed (F=19. 45, η²=0. 16, p<0. 001), as well as their interaction (F=10. 23, η²=0. 09, p<0. 01)on trustworthiness. Mediation analysis showed emotional arousal and cognitive load significantly mediated the effects of intonation and speed, with indirect effects of 0. 22 and-0. 27, respectively. Audience feedback supported these findings, with 45%highlighting the emotional engagement of varied intonation, 35%noting improved clarity with slow speed, and 20%emphasizing enhanced credibility through low-frequency tones. These results underscore the importance of dynamic intonation and optimal speed in enhancing audience trust in news delivery.

**Conclusions:** This study verifies the key influence of voice tone on the effectiveness of news communication and reveals for the first time its potential value in improving the mental health of audiences. The optimal design of changing intonation and slow speech speed can not only enhance the sense of trust in news broadcasting, but also have a significant emotional regulation effect, helping to alleviate the anxiety and cognitive load of the audience. This study provides a scientific basis for the media industry, suggesting that newscasters and AI voice technology developers focus on optimizing intonation and speed to enhance audience experience and communication effectiveness, while providing innovative application scenarios for mental health interventions.

## PHHS25083: THE INFLUENCE OF INFORMATION ASYMMETRY ON CONSUMER PSYCHOLOGICAL WORRY

Yunxiang Li^a,b^, Jing Pan^a,b^, Feng Luo^a,b^, Binxin Shen^b,c^

^*a*^ *Key Laboratory of Sichuan Cuisine Artificial Intelligence, Chengdu, China,* ^*b*^ *School of Economics and Management, Sichuan Tourism University, Chengdu, China,* ^*c*^ *Cork University Business School, University College Cork, Cork, Ireland.*

**Background:** With the development of cloud computing, information technology and Internet technology, the breadth and depth of people ‘s understanding of information has been improved, accompanied by information cocoons. Social media has gradually become the main channel for people to understand information. True and false information and opaque information will interfere with consumers ‘ judgment. In particular, some bad manufacturers are shoddy, which will lead to a crisis of confidence in related industries and upstream and downstream enterprises. Therefore, this paper will explore the relationship between food information asymmetry and consumer psychology.

**Subjects and Methods:** This paper uses literature review and keyword map, random and preset experimental methods, as well as behavioral and brain wave methods to explore the impact of information asymmetry on consumers ‘ psychological anxiety at the level of type attribute and informed sensitivity.

**Results:** The results consistently confirmed the correlation between information and psychology in the hypothesis. Among them, experiment 1 verified the relationship between food perception, information asymmetry, consumer anxiety, information sensitivity and other keywords by mapping analysis. Experiment 2 examined the relationship between effective information knowledge and effective value judgment in different situations through laboratory experiments and field experiments, and how the difference in information value evaluation affects consumers ‘ anxiety. Experiment 3 further tested our experimental and theoretical results through brain wave experiments, that is, the informed sensitivity of information significantly moderated the degree of information symmetry and consumer anxiety.

**Conclusions:** By emphasizing the importance of type attributes and informed environment, this study finds a skewed S-shaped relationship between the relative information asymmetry of goods and consumers ‘ psychological concerns, which improves our understanding of the psychological cognitive mechanism derived from information asymmetry. This study contributes to the dynamic literature of perception between commodity information asymmetry and psychological anxiety, and calls for more research to investigate this interesting field. At the same time, this study provides a direction for exploring the differences in anxiety between different industries and different consumer groups in the future. Information asymmetry can be alleviated by improving transparency and consumer education. Enterprises should provide detailed product information, and the government should legislate to protect consumer rights and limit misleading advertising. In the digital era, we should attach importance to the protection of personal information, clarify the privacy policy of enterprises, and enhance consumers ‘ awareness of self-protection.

**Acknowledgements**

This work was supported by Sichuan Key Laboratory of Philosophy and Social Sciences, Key Laboratory of Sichuan Cuisine Artificial Intelligence “Research on the mechanism and realization path of intelligent seasoning driving the improvement of Sichuan cuisine flavor value”, Project number: CR24Y12; “Research on the development of Sichuan cuisine industry under the background of artificial intelligence”, Project number: CR24Y20. Chengdu Cultural and Economic Research Center “Intelligent media empowers the inheritance and innovation of Tianfu cultural family”, Project number: CE202418.

## PHHS25084: STUDENTS CLASSROOM BEHAVIOR DETECTION WITH LOW LIGHT CONDITIONS FROM THE PERSPECTIVE OF MENTAL HEALTH

Zhi Yang ^a^

^*a*^ *Xi’an University of Science and Technology, Xi’an, 710054, Shanxi, China.*

**Background:** The premise of analyzing students’ mental health status in class is to detect students’ classroom behavior accurately. The accurate analysis and empirical application of artificial intelligence technology into the whole process of mental health and learning has become a current research hotspot.

**Subjects and Methods:** The College Students Classroom Behavior dataset was created to solve the problems of low efficiency and large amount of computation in the current detection algorithm of students’ classroom behavior, which could not meet the real-time detection of students’ classroom behavior under low light. An improved YOLOv3 algorithm for student classroom behavior detection is proposed to improve the accuracy of student classroom behavior detection under low light conditions. Firstly, dense connection blocks are introduced to improve the feature extraction capability of the backbone network, enhance the feature utilization rate of the network in the process of forward propagation and avoid the problem of gradient disappearance during network training. Secondly, the Slim-neck structure is used to optimize the feature fusion structure of the original YOLOv3, which enables efficient information fusion between feature graphs, further improves the detection accuracy of the student classroom behavior detection algorithm under low light conditions, and improves the detection speed by using the special lightweight convolution structure inside. Finally, CBAM attention module is introduced to refine the feature map proposed by the backbone network, strengthen the feature representation of small targets, and improve the ability of the network to detect students’ classroom behavior under low-light images and multi-target detection. So as to accurately analyze the students’ mental health state.

**Results:** The experiment results show that the enhanced processing of low-light images can effectively improve the image visibility and the effect of students’ classroom behavior detection. In the enhanced image detection, the AP of this method reaches 95.68%. Compared with other method, YOLOv5 and YOLOv3 models, the detection accuracy has been improved by 2.53%, 6.42% and 11.77% respectively, and the running speed has reached 29.31ms, which also has obvious advantages compared with other comparison methods.

**Conclusions:** The algorithm has higher detection accuracy and strong generalization ability, which can be competent for the detection task of students’ classroom behavior, effectively support the analysis and research of students’ mental health status.

**Acknowledgements**

This research was funded by the National Natural Science Foundation of China: (51974229), and the Shaanxi science and technology innovation team (2021TD-27).

## PHHS25085: ON THE PATH OF COMPREHENSIVE ABILITY IMPROVEMENT FOR MATHEMATICS MAJORS IN NORMAL COLLEGES BASED ON “WORKSHOP” FROM THE PERSPECTIVE OF PSYCHOLOGY

Hong Wei^a^, Xiaoli Lin^a^, Xuan Wu^a^, Jiachao Wei^b^

^*a*^ *School of Mathematics and Statistics, Nanning Normal University, Nanning 530100, China,* ^*b*^ *School of Education Science, Nanning Normal University, Nanning 530299, China.*

**Background:** This study focuses on integrating Chinese character writing into the mathematics and applied mathematics courses for normal students in colleges and universities. The aim is to cultivate the writing ability of these students while guiding them to abstract mathematical concepts from cultural contexts. Additionally, the study seeks to create a “situation technology curriculum model” to enhance students’ comprehensive problem-solving abilities and promote their all-round development. In the course design, special attention is given to students’ mental health and learning behaviors. By creating a relaxed learning environment through contextualized teaching, the model helps alleviate academic stress and enhances psychological resilience, enabling students to better adapt to academic challenges.

**Subjects and Methods:** From the perspective of psychology, the study involves students enrolled in mathematics and applied mathematics courses. The learning of mathematical knowledge is a continuous process of exploration and application for students, during which teachers should provide appropriate learning resources, follow students’ cognitive development patterns, and allow students to experience the “rediscovery” and “recreation” of new knowledge through contextual reconstruction and regeneration. Based on this concept, the research employs a “workshop” mechanism to construct a tripartite progressive training system, termed “university-

Workshop-middle school.“ This system promotes a shift from single to diverse approaches and from closed to open environments, fostering multi-party collaboration. Additionally, an interdisciplinary resource-sharing platform has been established to facilitate resource sharing and complementary advantages, providing strong support for students’ exploratory learning and teachers’ resource provision.

**Results:** The implementation of the “situation technology curriculum model” enables students to learn and understand mathematics from different fields, perspectives, and methods. The “university-workshop-middle school” system effectively improves the learning and teaching abilities of normal students, providing them with rich practical opportunities and diverse career development paths, thereby enhancing their comprehensive problem-solving skills and overall quality. The study also found that students’ mental health significantly improved during the course, with reduced academic stress and increased learning motivation and self-efficacy. These changes indicate that contextualized teaching not only enhances academic abilities but also positively impacts students’ mental health.

**Conclusions:** The integration of Chinese character writing into mathematics courses, combined with the “situation technology curriculum model” and the “university-workshop-middle school” system, significantly contributes to the holistic development of normal students. This approach not only improves their academic and teaching abilities but also offers diverse career opportunities, making it a valuable model for educational training. Additionally, the research results demonstrate that the model has a positive effect on students’ mental health, effectively alleviating academic stress and enhancing psychological resilience, providing important insights for future educational practices. By focusing on students’ mental health and learning behaviors, the curriculum design has been further optimized, offering new perspectives for cultivating well-rounded normal students.

**Acknowledgments**

This work was supported by a project grant from Guangxi Undergraduate Education Reform Project in 2024: Construction and Practice of “Three Learning” and “Four Party” Collaborative Practice Teaching System for Normal Majors under the Background of Professional Certification (Grant No.2024JGB266).

## PHHS25086: THE EFFECT OF PSYCHOLOGICAL EMPOWERMENT ON DIGITAL COMPETENCE OF UNIVERSITY TEACHERS: THE MEDIATING ROLE OF PSYCHOLOGICAL CAPITAL AND THE MODERATING ROLE OF ORGANIZATIONAL INNOVATION CLIMATE

Sijie Liu^a^, Zhenhua He^a^

^*a*^ *Xinhua College of Ningxia University, Yinchuan, Ningxia 750021, China.*

**Background:** With the changes in the field of education, traditional teaching methods are being replaced by new modes of digitization and informatization, and teachers not only need to master solid subject knowledge, but also have a certain degree of digital technology and information processing ability, so that teachers can better adapt to the new teaching environment. Based on the self-determination theory, this paper explores the influence of psychological empowerment on teachers’ digital competence from the perspective of college teachers’ psychology. This study can theoretically explain the reasons for the lack of digital competence among college teachers and further explore the intrinsic influence mechanism between the two, which can help clarify the drivers of digital competence among college teachers, provide more precise guidance for educational reform, and motivate college teachers to adopt digital teaching technologies. Therefore, this study explores the impact of psychological empowerment on digital competence and the moderating role of organizational innovation behavior from the psychological capital of college teachers.

**Subjects and Methods:** In this study, a questionnaire survey method with convenience sampling was used to distribute questionnaires to a total of 686 university teachers from eight universities in Ningxia, China, mainly using the Psychological Empowerment Scale, Digital Competence Scale, Psychological Capital Scale, and Organizational Innovation Climate Scale.

**Results:** Psychological empowerment has a significant positive effect on digital competence of college teachers; psychological empowerment has a significant positive effect on psychological capital of college teachers; psychological capital mediates the relationship between psychological empowerment and digital competence of college teachers; and organizational innovation climate moderates the relationship between psychological empowerment and digital competence of college teachers.

**Conclusions:** The digital competence of college teachers is affected by the psychological empowerment variable, the higher the psychological empowerment perception, the higher the level of digital competence of college teachers, and the positive psychological state prompts them to be more willing to take the initiative to explore and learn digital technology and apply it to teaching and scientific research, so as to enhance their digital competence. The psychological capital of college teachers is affected by psychological empowerment, the higher the perception of psychological empowerment, the higher the psychological capital of college teachers, and the positive psychological empowerment state prompts teachers to be more resilient in the face of difficulties, thus significantly improving their own psychological capital. Psychological capital plays a direct mediating role in the psychological empowerment and digital competence of college teachers, and psychological empowerment can affect the digital competence of college teachers through psychological capital. It plays a key moderating role in the association between psychological empowerment and digital competence, and increasing psychological empowerment can improve the digital competence of college teachers with high organizational innovation climate.

**Acknowledgements**

This research was supported by 2024 Research Project of Ningxia Hui Autonomous Region Social Science Planning Project (Pedagogy) “Research on the Measurement of Digital Competence and Construction of Evaluation Indicators for Teachers in Private Colleges and Universities in Ningxia under the Background of Digitization (24NXJC08)”.

## PHHS25087: DIGITAL ANXIETY MANAGEMENT IN ECONOMICS LEARNING: TECH-PSYCHOLOGY INTERACTIONS

Ling He^a^, Juan Sun^a^, Ting Xiang^a^, Yongqiang Chen^b^

^*a*^ *Huaihua university, Huaihua, China,* ^*b*^ *Yongqiang Chen, Jinggangshan University, Jiangxi, China.*

**Objective:** As a pedagogical innovation integrating computer-generated virtual environments with scenario simulation, virtual simulation teaching (VST) exhibits three defining attributes: environmental permeability, cognitive embodiment, and bidirectional feedback loops. This paradigm enables the concretization of abstract economic principles through situational reduplication while facilitating authentic decision-making simulations. Building upon Cognitive Load Theory and Emotional Regulation Framework, this study investigates how psychologically scaffolded VST interventions enhance professional competency development in economics education, with particular focus on the mediating role of anxiety mitigation in optimizing cognitive resource allocation.

**Research Subjects and Methods:** Undergraduate economics students at Huaihua University engaged in virtual simulation teaching were studied. Data were collected through structured questionnaires and semi-structured interviews, followed by advanced statistical analysis using structural equation modeling (SEM) to examine causal relationships and mediating effects in teaching effectiveness.

**Results:** The study revealed that the immersive and interactive features of virtual simulation teaching significantly enhance learning outcomes (p < 0.01). Learning engagement was identified as a critical mediating factor between virtual simulation teaching characteristics and learner performance. Multivariate analysis demonstrated significant variations in teaching effectiveness across gender and academic year cohorts (p < 0.05). Notably, learners with enhanced anxiety management competencies and effective emotion-regulation strategies demonstrated superior performance in economic decision-making simulations.

**Conclusions:** This study proposes a multifaceted framework to optimize virtual simulation pedagogy through four strategic interventions: (1) leveraging advanced technologies to enhance immersive experiences via high-fidelity simulation environments; (2) implementing evidence-based motivational strategies incorporating gamification and performance-based reward systems; (3) developing differentiated instructional designs catering to varying cognitive levels and learning needs; and (4) establishing comprehensive faculty development programs to enhance technology-integrated pedagogical competencies. Crucially, the framework emphasizes the integration of mental health components within virtual simulations, particularly through adaptive psychological support systems and emotion-regulation modules embedded in economic decision-making scenarios. These neurocognitive interventions demonstrate significant potential for improving learners’ emotional regulation and metacognitive processing, thereby enhancing both academic achievement and professional skill development.

**Acknowledgements**

This work was supported by a project grant from Key Teaching Reform Project of Hunan Province in 2021: Research and Practice of Online-Offline Blended Teaching of Western Economics in the Context of First-class Curriculum Development (HNJG-2021-0186).

## PHHS25088: RESEARCH ON THE APPLICATION PATH OF CLASSICAL MUSIC IN ENHANCING UNIVERSITY STUDENTS’ PSYCHOLOGICAL HEALTH

Tao Liu ^a^

^*a*^ *Wuzhou University, Wuzhou 543000, China.*

**Background:** As society enters a new phase of development, continuous advancements in higher education reform have prioritized the psychological health of university students. Reducing the incidence of psychological disorders among students has become a crucial issue for universities nationwide, striving to improve educational quality and cultivate talent. This study, grounded in psychological education for students at regional universities, focuses on the art form of classical music. It explores the mechanisms and practical value of classical music in psychological health education. By examining the intrinsic connection between classical music and psychological health, and addressing existing challenges in psychological health interventions and the development of art therapy resources, this research aims to provide a theoretical foundation for establishing a multidisciplinary framework in university psychological health education.

**Subjects and Methods:** The study targeted university students at regional institutions as its primary population. By conducting surveys among university psychological health educators, the research analyzed students’ psychological challenges and needs. A combination of literature review and field research was employed to assess the influence of classical music on students’ psychological health, with a particular focus on its efficacy in alleviating stress and enhancing emotional well-being.

**Results:** The findings reveal that classical music significantly improves students’ emotional states, reduces anxiety and stress, and is particularly effective in alleviating academic and career-related pressures. It also enhances emotional stability and cognitive functioning. Field surveys demonstrate that exposure to classical music fosters self-relaxation and boosts concentration among students. Those who experienced classical music interventions as part of psychological health education exhibited heightened self-awareness and an improved appreciation for art.

**Conclusions:** The results affirm that integrating classical music into psychological health education significantly promotes the psychological health of university students. An increasing number of psychological health educators at regional universities are adopting such approaches to address practical challenges in their work. The fusion of classical music with psychological health education not only enhances students’ cognitive and social skills but also bolsters their sensitivity to and recognition of psychological health issues. This integration provides a novel approach and theoretical support for university psychological health education, offering noteworthy practical value.

## PHHS25089: HAPPY CHINA CONSTRUCTION FROM THE PERSPECTIVE OF MENTAL HEALTH: THEORY, PRACTICE AND CHALLENGES

Rongmei Li ^a^

^*a*^ *Zhejiang University of Water Resources and Electric Power, Hangzhou 310018, Zhejiang, China,*

**Background:** During times of significant national challenges, the importance of societal well-being and individual happiness are often been emphasized. In this context, various schools of thought have developed frameworks for improving the overall happiness and mental well-being of citizens. One such perspective, often discussed in historical discourse, revolves around the establishment of a harmonious society where people’s mental health and happiness are prioritized. This concept has evolved into contemporary discussions surrounding the balance between personal happiness and societal goals, often referred to as the “happiness paradox.” The pursuit of happiness, therefore, has become a key focus in modern societal frameworks, including the development of mental health initiatives and community well-being strategies.

**Subjects and Methods:** The study delves into the theoretical underpinnings and practices designed to promote individual and collective happiness, focusing on how political and social structures contribute to the mental well-being of citizens. Drawing on historical texts and modern research, the exploration emphasizes the role of thought leadership in fostering an environment where people feel valued and empowered to achieve personal happiness. By examining the practices of moral education, collective efforts, and societal contributions to happiness, the study analyzes the methods that promote a sense of fulfillment and emotional stability within a community. This involves understanding how psychological well-being is influenced by both individual mindset and the collective societal environment.

**Results:** The results of this exploration highlight that societal structures that prioritize mental health initiatives, create opportunities for personal fulfillment, and promote moral education can significantly contribute to the happiness of individuals. These initiatives foster a supportive environment where individuals feel connected to a larger purpose, reducing feelings of alienation or dissatisfaction. Furthermore, the study reveals that addressing the “happiness paradox” — the challenge of balancing individual well-being with collective aspirations — plays a crucial role in shaping a positive mental health environment. The balance between personal goals and societal expectations, when well managed, can lead to an increase in overall life satisfaction and mental health.

**Conclusions:** The concept of a “happy society,” as an ideal goal, remains deeply relevant in contemporary discussions about mental health and societal well-being. The practices that support this ideal, such as prioritizing education, providing social support systems, and promoting individual growth, are vital in creating an environment where both collective happiness and personal fulfillment are achievable. In conclusion, fostering mental health through community-focused initiatives and philosophical principles remains a guiding force in addressing the happiness paradox and improving societal well-being. This model offers crucial insights into how contemporary societies can work towards a more balanced, harmonious, and mentally healthy future for all.

## PHHS25090: INTERNET USAGE AND WOMEN’S SUBJECTIVE WELL-BEING: BIG DATA ANALYTICS BASED ON THE PERSPECTIVE OF SOCIAL INTERACTION

Ying Ren^a^, Huan Wang^a^, Dongqing Zhu^a^, Renqu Tian^b^, Xia Duan^b^

^*a*^ *School of Economics and Management, Yunnan Minzu University, Kunming 650504,China,* ^*b*^ *Lancang-Mekong International Vocational Institute, Yunnan Minzu University, Kunming 650504, China.*

**Background:** The subjective well-being (SWB) of women is crucial for the stability of families and society. However, research on the impact of internet usage on women’s sense of happiness, particularly in developing countries, is extremely limited. This article aims to use big data to study the impact of internet usage on the subjective sense of happiness among Chinese women and explore its underlying mechanisms.

**Subjects and Methods:** Although instrumental variable methods were applied to address endogeneity concerns, the results continue to reinforce the conclusion. Based on the cross-sectional big data from the China General Social Survey (CGSS2017), we selected two dependent variables (degree of happiness and a binary variable indicating whether one is happy), and one explanatory variable (frequency of internet usage). We employed OLS and Logistic regression models to examine how internet usage affects the SWB of women, as well as the impact of online social interactions on urban women (N=4303) and rural women (N=2344). The CGSS2017 survey includes data from 31 provinces, with a total effective sample size of 12,582 and an average age of 51 years. Of this, 6,647 participants are women, making up 52.83% of the overall sample.

**Results:** This study employed a two-stage least squares estimation (2SLS) and instrumental variables to mitigate endogeneity issues. The research findings indicate that: (1) Internet usage significantly enhances women’s SWB (P<0.006). (2) Online social interactions have a significantly positive impact on increasing women’s SWB. (3) Heterogeneity tests reveal that the impact of internet usage on well-being differs between urban and rural areas, with rural women experiencing a greater positive effect on well-being compared to urban women. (4) While internet-based social interactions have no significant impact on the SWB of urban women, they significantly enhance the SWB of rural women. Additionally, the study indicates that the boost in happiness resulting from internet usage exceeds the influence of marital status and personal income levels on women’s SWB.

**Conclusions:** Through the analysis of CGSS big data, it is found that Internet usage can significantly enhance women’s sense of happiness. Online social interactions make up for the absence of fully established offline social groups in rural areas, promote emotional connections with family and friends, enhance personal social worth, and significantly improve the SWB of rural women. This study has certain limitations; the object of the study is Chinese women, and there may be differences in other countries or regions around the world due to different social environments.

## PHHS25091: RESEARCH ON THE DRIVING FACTORS, ENTREPRENEURSHIP MODELS, AND SUPPORT SYSTEM OF RURAL ENTREPRENEURSHIP AMONG ECONOMICS AND MANAGEMENT MAJORS BASED ON THE ENHANCEMENT OF MENTAL HEALTH LITERACY

Min Yu^a^, Tingting Liu^a^

^*a*^ *School of management, Chongqing Institute of Engineering, Chongqing 401320, China.*

**Background:** With the annual increase in the number of college graduates, the imbalance between supply and demand in the job market has become increasingly prominent. Channeling economics and management majors into rural entrepreneurship not only opens new pathways to alleviate employment pressures but also drives rural industrial revitalization through the infusion of talent. Leveraging their professional expertise, economics and management students are emerging as vital forces in revitalizing rural economies. Despite policy support and development opportunities provided by China’s rural revitalization strategy, most graduates still confine their career plans to urban areas, harboring cognitive biases toward rural entrepreneurship. Additionally, economics and management students commonly face challenges such as a lack of practical experience and insufficient support systems during entrepreneurship, leading to significant mental health risks, including social identity anxiety and career development confusion. Therefore, it is imperative to establish a multi-dimensional collaborative mechanism involving government guidance, university empowerment, and family support to provide sustainable development support for rural entrepreneurship among college students.

**Subjects and Methods:** This study focuses on economics and management majors from universities in Chongqing, adopting a mixed-methods approach combining qualitative and quantitative research. First, a systematic literature review was conducted to synthesize existing research and design measurement scales. Second, a research model was developed to explore the driving factors of rural entrepreneurship among economics and management students, with a focus on enhancing mental health literacy. Hypotheses were proposed and tested using SPSS statistical software and structural equation modeling (SEM) for data analysis and empirical validation. Through factor analysis, key variables influencing rural entrepreneurship intentions and mental health were extracted, and the direction and magnitude of their impacts were quantified. Finally, integrating findings from literature and empirical research, this paper proposes effective entrepreneurship models and targeted strategies to strengthen rural entrepreneurial capabilities and improve mental health outcomes.

**Results:** The study reveals that five factors—family support, policy environment, social climate, university training, and individual entrepreneurial drive—exert significant positive effects on students’ rural entrepreneurship intentions and mental health levels, with contribution ratios of 0.174, 0.197, 0.162, 0.148, and 0.212, respectively. Based on empirical results, this paper proposes entrepreneurship models to enhance the success rate of rural ventures and alleviate mental health challenges. A collaborative “government-society-family-university” support system is constructed, including measures such as improving incentive mechanisms at the policy level, strengthening practice-oriented competency development in education, fostering a positive social atmosphere, and transforming traditional employment perceptions within families.

**Conclusions:** By developing scientifically grounded entrepreneurship models and a systematic support system, this study facilitates the synergistic development of students’ innovation capabilities and mental health literacy. This approach injects sustainable human capital dynamism and innovative vitality into the high-quality development of agricultural and rural economies.

**Acknowledgments**

This study was supported by the following projects: The 2024 Science and Technology Research Project of Chongqing Municipal Education Commission (Project No.: KJQN202401903): “Research on Consumer Satisfaction Evaluation and Marketing Model Transformation of Chongqing Rural Tourism in the Context of the Digital Economy.”

(2) The 2024 Chongqing Social Science Planning Project (Project No.: 2024NDQN079): “Research on the Behavioral Mechanisms and Promotion Strategies of High-Quality Short Video Creation in the Era of Digital Gig Economy.”

(3) The 2024 School-Level Scientific Research Project of Chongqing Institute of Engineering (Project No.: 2024XSKY001): “Research on the Behavioral Mechanisms and Promotion Strategies of AI-Empowered Digital Content Creation.”

## PHHS25092: IDENTIFICATION THEORY IN WEBCAST MEDIA: EXPLORING ONLINE AND OFFLINE BEHAVIORS

Fei Teng ^a,b^

^*a*^ *Guangdong University of Science and Technology, Guangdong, China,* ^*b*^ *SEGi University, Malaysia.*

**Background:** The rise of networks, smartphones, and video platforms has fueled the rapid growth of webcasts, transforming them into a dominant form of social media. Initially centered on video games, webcast content has expanded to include food, makeup, travel, education, and social interaction. Unlike traditional media, webcasts thrive on high levels of real-time engagement, allowing broadcasters to share personal experiences and ideas while interacting directly with their audience. This interactivity distinguishes webcasts and aligns with psychological theories of identity, prompting exploration into how these dynamics shape broadcaster-audience relationships both online and offline.

**Subjects and Methods:** This study applies identification theory, specifically broadcaster identification (connection to individual broadcasters) and group identification (sense of belonging to the audience community), to examine interaction behaviors in webcasts. Data were collected through questionnaires distributed to webcast users in mainland China and Taiwan. Participants reported their engagement levels with broadcasters and fellow viewers, both within the digital space and in real-world settings. The analysis focused on correlating identification factors with interaction frequency and quality, employing statistical methods to assess the strength and significance of these relationships.

**Results:** Findings revealed that broadcaster identification and group identification are strongly linked to increased interaction between broadcasters and their audience. Users who felt a personal connection to broadcasters or a sense of community with other viewers exhibited significantly higher levels of engagement, both online (e.g., comments, likes) and offline (e.g., meetups, events). The positive correlations were consistent across the sampled regions, suggesting that psychological identification enhances the social impact of webcasts beyond the screen. Data underscored that real-time, two-way communication amplifies these effects, reinforcing audience loyalty and participation.

**Conclusions:** This research highlights the pivotal role of psychological identification in driving webcast interactions, offering a framework to understand how digital platforms foster meaningful connections. It proposes refining identification theories to account for webcast-specific dynamics and suggests practical applications, such as enhancing broadcaster training to boost audience engagement. However, limitations include the study’s regional focus and reliance on self-reported data, pointing to the need for broader, longitudinal research. Future studies could explore additional psychological factors, like emotional attachment or motivation, to deepen insights into webcast communities.

**Acknowledgements**

Supported by Research Project Fund of Guangzhou The Trainers Management Consulting Co., LTD.

## PHHS25093: INNOVATIONS IN MENTAL HEALTH EDUCATION MODELS UNDER THE INTEGRATION OF DIGITAL MEDIA AND DESIGN EDUCATION

Yuanhui Huang^a^, Ke Li^b^

^a^ *Cheongju University, Cheongju-si 284960, Korea,* ^*b*^ *School of Literature and Journalism, Shandong University, Shandong, China.*

**Background:** With the rapid development of digital media technology and the continuous deepening of design education, mental health education faces new opportunities and challenges. Traditional mental health education models have shortcomings in information dissemination efficiency, student engagement, and personalized intervention. The integration of digital media and design education provides innovative pathways for mental health education. This study aims to explore the innovations in mental health education models under the integration of digital media and design education to enhance educational effectiveness and students’ mental health levels.

**Subjects and Methods:** This study targets design students and employs a combination of literature review, case analysis, and empirical research. By analyzing successful domestic and international cases, a multimodal, interactive, and immersive mental health education model based on digital media was constructed and applied in design education courses. The practical effects were assessed through questionnaires, interviews, and experimental comparisons.

**Results:** The study found that the integration of digital media and design education significantly enhances the attractiveness and engagement of mental health education. Multimodal resource design, interactive platforms, and immersive experiences effectively strengthen students’ understanding and application abilities of mental health knowledge. Empirical results show that students in the experimental group significantly outperformed those in the control group in terms of mental health levels and learning satisfaction, indicating that the innovative model has significant advantages in mental health education.

**Conclusion:** The integration of digital media and design education offers new ideas and methods for mental health education. Through multimodal, interactive, and immersive designs, the effectiveness of mental health education can be significantly improved. The study recommends further promoting this model in design education and optimizing the mental health education system by incorporating more technological means to support students’ comprehensive development.

## PHHS25094: A NEW INVESTIGATION AND EMOTIONAL ANALYSIS OF YU QIAN QING KE IN QUANZHOU NANYIN

Boshi Li^a^, Ke Li^a^

^*a*^ *Quanzhou University of Information Engineering, Quanzhou, Fujian, China.*

**Objective:** This study aims to explore the similarities and differences in the records of the Quanzhou *Nanyin* anecdote *Yu Qian Qing Ke* across various historical documents and folk legends, focusing on the timeline of the “*Wu Shao Fang Xian*” traveling to the capital, their names, the background of their official recognition, and the emotional state during their performance in the capital.

**Subjects and Methods:** By employing the methods of literature analysis and field investigation, this study conducted research in Quanzhou, Xiamen, Nan’an, Yongchun, Shishi, and Jinjiang in Fujian, China. It systematically collected and reviewed historical records related to *Yu Qian Qing Ke* from the late Qing Dynasty to the present.

**Results:** The research findings reveal that there are five different versions regarding the timeline of *Yu Qian Qing Ke* traveling to the capital, the names of its members, and the background of their official recognition. However, the emotional state of the *Wu Shao Fang Xian* during their performance in the capital is consistently documented in historical records and oral accounts from folk artists.

**Conclusions:** Through comparative analysis, this study concludes that the journey to the capital took place in 1708. The members of the *Wu Shao Fang Xian* were Li Yi (courtesy name Wenbo) and Wang Zhou (courtesy name Shangguang) from Jinjiang, Ye Qiu (courtesy name Shiye) from Jinjiang, Chen Pin (courtesy name Yunxing) from Tong’an, and Huang Tai (courtesy name Shixiang) from Yongchun. Accompanying them as mentors were Wu Wenzhi and Chen Ning from Jinjiang, Fu Ting from Nan’an, Hong Song from Hui’an, and Li Yi from Anxi. Regarding the background of their official recognition, this study supports the account recorded by Lin Xiangyu: Emperor Kangxi once asked Grand Secretary Li Guangdi about the state of “Nanyue” (Southern Music). In response, Li Guangdi promptly sent a letter back to his hometown, selecting the most outstanding musicians to present themselves at the imperial court. As for their emotional state, the study suggests that the performers experienced complex emotions due to their prolonged stay in the imperial palace. Through their performance of “Hundred Birds Returning to the Nest”, they moved the emperor, earning them the honorary titles Yu Qian Qing Ke (Imperial Guests) and Wu Shao Fang Xian, as well as the privilege of being bestowed with “palace lanterns and ceremonial umbrellas” before returning to their hometowns in glory. The findings and arguments explored in this study provide new insights and serve as a potential reference for academic research in the field of Nanyin.

## PHHS25095: CONSTRUCT THE AI-EMPOWERED BILINGUAL INTELLIGENT COURSE ON THE EXCELLENT TRADITIONAL CHINESE CULTURE WITH THE CONSIDERATION OF MENTAL HEALTH

Peixiang Yan^a^, Shangqi Li^b^

^*a*^ *School of Foreign Languages, Guangdong Pharmaceutical University, Guangzhou 510006, China,* ^*b*^ *School of Marxism, Guangdong Polytechnic Normal University, Guangzhou 510665, China.*

**Background:** Students’ mental health should be one of the greatest concerns for the construction of any new course in the educational field. In the new era, learning and inheriting the excellent traditional Chinese culture has emerged as a crucial mission of China’s education. Thus, establishing a course on this culture which also emphasizes mental health education is necessary. The purpose of the present research is to construct the AI-empowered bilingual intelligent course on the excellent traditional Chinese culture which takes students’ mental health into consideration, a new type of cultural course combining technological warmth, cultural depth, and emotional and psychological care which can not only elevate the cultural literacy and confidence of Chinese foreign language learners but also foster their mental well-being during the process of culture learning.

**Subjects and Methods:** The research employed a combination of literature review and survey methods. A literature review was conducted to gain a full understanding about the connotation of the excellent traditional Chinese culture, analyze the real advantages for establishing the AI-empowered bilingual intelligent course on the excellent traditional Chinese culture with the consideration of students’ mental health, and explore avenues for constructing this course with the integration of mental health education elements. For better constructing this new type of culture course, the survey method was employed to get the ideas from teachers and students, particularly their attitudes and feelings about the new course. Twenty English teachers and twenty English majors in three universities participated in the survey. The survey was conducted in the form of face-to-face interviews which were all recorded, transcribed, and analyzed.

**Results:** The detailed avenues for constructing the AI-empowered bilingual intelligent course on the excellent traditional Chinese culture are establishing the new educational goal for foreign language teaching in China which also considers students’ mental health, developing AI-driven digital textbooks, reforming traditional teaching methods and adopting the AI-empowered situationalized teaching method, cultivating a high-caliber team of culture teachers, and seeking ideas and suggestions from teachers and students to better the construction of the new course. Besides, the detailed analysis about all the teachers’ and students’ answers in the survey obviously indicates their support for this new type of culture course, but this new course can make both teachers and students feel pressure and anxiety to some extent stemming from many reasons, and teachers felt more pressure than students for the new course.

**Conclusions:** The AI-empowered bilingual intelligent course on this culture with the consideration of mental health is helpful for a comprehensive, systematic, and efficient study of culture. It can guide learners to possess noble virtues, deepen their cultural understanding, improve their cultural identity and cultural confidence, and foster their positive mental state. To increase the effectiveness of culture education, this course should be offered at varying difficulty levels to suit learners with different levels of bilingual proficiency. When designing and implementing the new culture course, it is imperative to thoughtfully consider the ways to assist teachers in mitigating teaching pressure, reduce students’ learning pressure, and alleviate anxiety for both teachers and students.

**Acknowledgments**

This research was supported by one achievement of the project entitled Research on the Integration of the Excellent Traditional Chinese Culture into Foreign Language Education in Universities under the Background of Educational Informatization (Grant No. GD22WZX02-01), which received funding from Philosophy and Social Sciences Planning Office of Guangdong Province. It’s also supported by China General Project of National Social Science Fund in 2024, Research on Strengthening the Awareness of Chinese National Community among Hong Kong, Macao, and Taiwan Youth in Mainland Universities with Chinese Cultural Identity (Grant No. 24BKS151).

## PHHS25096: RESEARCH ON THE DUAL IMPACT OF GREEN FINANCE REFORM ON URBAN CARBON PRODUCTIVITY AND PUBLIC PSYCHOLOGICAL NEEDS

Kexin Han^a^, Boming Zhao^a^, Yan Wang^a^

^*a*^ *Southwest Minzu University, Chengdu 610000, Sichuan, China.*

**Background:** As climate change becomes increasingly serious, public concerns about environmental quality, such as “ecological anxiety”, have gradually become an important factor affecting mental health. This study innovatively explores how the China Green Finance Reform and Innovation Pilot Zone (GFRIPZ) policy can improve urban carbon productivity while meeting the public’s psychological needs for environmental safety, economic stability, and self-realization, thereby promoting social and psychological health.

**Subjects and Methods:** Based on panel data from 285 prefecture-level cities in China spanning 2009 to 2022, this study employs the synthetic control method by designating cities with Green Financial Reform and Innovation Pilot Zones as the policy intervention group and constructing counterfactual control groups using non-pilot cities. The research framework incorporates: Policy Evaluation through a difference-in-differences model; Robustness Verification using permutation tests. Constructing a “policy → environmental improvement/green employment → sense of security/sense of belonging/self-actualization needs satisfaction → mental health” transmission path based on Maslow’s hierarchy of needs theory.

**Results:** The GFRIPZ policy has significantly enhanced carbon productivity in pilot cities compared to synthetic control groups, with the policy effects demonstrating an increasing trend over implementation years. The psychological needs satisfaction mechanism is manifested as follows: The improvement of environmental quality significantly reduces the public’s ecological anxiety level; New employment opportunities created by the expansion of green industries meet the self-realization needs of young people; The increase in community environmental governance participation brings significant gains in sense of belonging. In terms of space, the eastern region has shown a dual improvement in “environment-psychology” due to its economic foundation advantages, while the western region has more prominent sense of gain.

**Conclusions:** The GFRIPZ policy generate synergistic benefits through physical environmental improvement and psychological needs. Suggestions: (1) Establish an “environment-psychology” two-dimensional policy evaluation system and include the public ecological sentiment index in the assessment; (2) Embed psychological health promotion clauses when designing green financial products (such as low-carbon community psychological service supporting funds); (3) Develop differentiated environmental education projects based on the psychological needs characteristics of the central and western regions. This study provides empirical evidence for the interdisciplinary integration of climate policy and mental health promotion.

## PHHS25097: AN EMPIRICAL ANALYSIS OF THE INFLUENCING FACTORS OF SHORT VIDEO RECOMMENDATIONS ON USER SATISFACTION AND MENTAL HEALTH ON THE DOUYIN PLATFORM: A STUDY BASED ON THE LOGISTIC - ISM MODEL

Qiuxiang Yi^a^, Chunsheng Yang^a^

^*a*^ *Faculty of New Commercial Science, Anhui Sanlian University, Hefei 230601, China.*

**Background:** Douyin is a globally renowned short-video sharing platform, and its user base and influence are expanding steadily. Intelligent recommendation assumes a significant role in the Douyin short-video platform. The platform’s intelligent recommendation strategy is to push short videos that are of interest to users based on their individual needs, thereby continuously increasing users’ adherence to the platform. However, as users’ immersion deepens, problems such as information repetition, addiction risks, and the spread of negative information frequently occur, which not only affect users’ satisfaction with the platform but also may cause users to have psychological problems such as anxiety and depression. Currently, numerous studies have focused on the Douyin short-video platform, intelligent recommendation, and factors influencing user satisfaction separately. Fewer studies have integrated all three aspects to investigate the impact of intelligent recommendation on user satisfaction and mental health on the Douyin short-video platform.

**Subjects and Methods:** This paper first elucidates the relevant theories of intelligent recommendation, user satisfaction influencing factors, Social media and mental health. Secondly, it conducts a questionnaire survey on “the influencing factors of user satisfaction in intelligent recommendation on Douyin short video platform” using Wenjuanxing, and finally constructs a multi-ordered Logistic model and ISM model to analyze the influencing factors of user satisfaction in intelligent recommendation and mental health on Douyin short video platform, as well as the interlinkage and hierarchical structure of the influencing factors.

**Results:** The paper finds that: (1) The factors influencing user satisfaction and mental health mainly include the duration of platform usage, the degree to which the recommendation system assists users in discovering content of interest, the quality of recommended content, the accuracy of recommended content. (2) Among the factors, the quality and the timeliness of recommended content, the friendliness of the recommendation system interface are surface-level factors.

**Conclusion:** (1) The quality of the recommended content, the timeliness of the recommended content, and the friendliness of the recommendation system interface have a direct influence on user satisfaction and mental health with intelligent recommendation on theDouyin short video platform.(2) The stability of the recommendation system plays a role in regulating both deep-level and surface-level factors, and the positive effect of deep-level factors such as the precision of the recommended content on the user’s evaluation of surface-level factors like the quality of the recommended content will be enhanced.(3) Platform usage time, the extent to which the recommendation system helps users discover content they are interested in, the precision of the recommended content, the diversity of the recommended content, and the degree of personalization of recommendations have a fundamental influence on user satisfaction and mental health with intelligent recommendation on theDouyin short video platform.

**Acknowledgements**

This research was supported by the Anhui Provincial Online-offline Blended First-class Course Project (2023xsxx363), the Innovation Project of the Anhui Provincial Social Sciences Federation (2021CX517), the Scientific Research Project of Universities in Anhui Province (2024AH052448), and the “Four New” Research and Reform Practice Project of Anhui Province (2023sx137).

## PHHS25098: GUIDELINES FOR DESIGNING HEALTH-ORIENTED ECOLOGICAL PARKS: LANDSCAPE PLANNING FRAMEWORK UNDER MULTIDISCIPLINARY COLLABORATION

Ting Liu^a^, Xianjun Zeng^a^, Tao Zi^a^, Xuesong Liu^a^

^*a*^ *Hunan Biological and Electromechanical Polytechnic, Changsha, Hunan 410127, China.*

**Objective:** In the process of urbanization, there is a significant correlation between the lack of natural contact and the high incidence of chronic diseases (such as cardiovascular disease and increased depression). Biodiversity, as a “natural pharmacy” for regulating human health, urgently needs systematic integration. This study aims to construct a health-oriented ecological park design framework through an interdisciplinary approach, to achieve synergistic protection and health intervention goals, thereby mitigating the negative impacts of urbanization on public health.

**Subjects and Methods:** Based on evidence-based design methodology, the “triple helix model” (ecological layer-individual layer-social layer) is proposed to elucidate how biodiversity influences health through three-dimensional pathways: 1) Ecological Layer: Native plant communities enhance immune function by purifying air (increasing PM2.5 adsorption efficiency by 42%) and regulating microbial exchange; 2) Individual Layer: Natural sensory stimulation (such as plant volatiles) activates the parasympathetic nervous system, reducing cortisol levels (verified reduction of 26%); 3) Social Layer: Diverse habitats promote intergenerational interaction, and increased community cohesion reduces loneliness risk by 29%. The world’s first health-oriented ecological park design guide—”BIO-HEALTH Framework” has been developed, integrating a biodiversity health database (PHI refers to quantified plant health utility), a multi-objective co-design engine (to optimize ecological-health goal conflicts), and a cultural adaptability regulator (to align with diverse health concepts), while using mixed reality (MR) technology to verify design reliability.

**Results:** Virtual case (Yangtze River Delta “Health Green Chip” Park) validation shows: stress perception scores decreased by 18.7% (DASS-21 scale), cortisol reduction error ≤15%; it is predicted that a 20-year intervention can reduce the incidence of type 2 diabetes by 7.3% (95%CI: 5.1%-9.5%); community interaction frequency increased by 2.3 times, and social isolation among the elderly group was significantly alleviated. Policy tools (health ecosystem bank, biodiversity bond) and participatory governance models (citizen jury system) have been proposed to incorporate health performance into local government evaluations (weight ≥10%), accelerating project implementation.

**Conclusions:** This study achieves multidimensional synergy of ecology, health, and culture through the “BIO-HEALTH Framework,” transforming urban green spaces from “landscape containers” into “health infrastructure.” It directly responds to the strategic needs of “Healthy China 2030.” The scientific approach provides replicable solutions for chronic disease prevention, mental health promotion, and enhancing health equity. At the same time, it contributes Chinese wisdom to public health governance in the global urbanization process.

**Acknowledgments**

The research was supported by Fund Project: The Science Research Outstanding Young Project of Education Department of Hunan Province, China (Grant No. 24B1034).

## PHHS25099: RESEARCH ON THE MECHANISM AND EVALUATION OF THE EFFECT OF CAPITAL UTILIZATION ON RURAL GOVERNANCE EFFICIENCY AND MEDICAL SERVICE QUALITY: BASED ON FIELD SURVEY DATA IN ZHEJIANG PROVINCE

Zhou Zhou^a,^, Li Wu^a,^, Hao Liang^a^, Lingzhen Ke^a^, Yao Yang^a^, Kangni Xia^a^, Qingyi Zhou^a^, Jintao Fu^a^

^*a*^ *College of Humanities and Foreign Languages, Jiliang University, Hangzhou, Zhejiang 310018, China.*

*ZZ and LW contributed equally to this work.*

**Objective:** In the context of rapid urbanization, the countryside is facing a series of challenges, such as population outflow, resource shortage, and governance difficulties. The situation is further complicated by the relatively backward state of rural healthcare. With the exodus of the young and middle-aged population, the remaining elderly and children in rural areas often face difficulties in accessing high-quality medical services. There is a lack of professional medical staff, and medical facilities are outdated. As a result, rural development is in urgent need of reinforcement. External capital intervention has emerged as a crucial driving force for rural revitalization. This study is dedicated to comprehensively exploring the intricate mechanism of how the utilization of rural capital impacts rural governance efficiency and medical service quality through empirical data.

**Subjects and Methods:** This research is grounded in the field survey data collected from over 20 villages in Zhejiang Province. A mediation effect model was meticulously constructed to precisely analyze the impact of capital flowing into rural areas on rural governance and medical service quality. A total of 400 questionnaires were distributed to villagers, covering various aspects of rural life. After strict screening, 356 valid questionnaires were obtained. The variables considered in this study are comprehensive, including infrastructure (such as transportation and public service facilities), political participation (villagers’ involvement in village affairs decision-making), social networks (the connections among villagers and with the outside world), education (the educational level and training opportunities of villagers), and medical care (the quality of rural medical services and the security system).

**Results:** The study found that infrastructure, political participation, social networks, and education play a significant mediating role between capital inflows and governance efficiency (p<0.05), while medical care social security, and industrial development do not show significant mediating effects.

**Conclusions:** The utilization of rural capital improves rural governance efficiency and medical service quality mainly through key intermediary channels such as infrastructure and education. Based on this, this study puts forward corresponding policy suggestions, including optimizing the government support system and increasing the investment in rural medical care and other infrastructure at the government level; In the aspect of enterprises, improve the internal management mode and external cooperation mechanism of enterprises; At the villagers’ level, it is necessary to protect the villagers’ rights and interests and improve the mechanism of interest distribution; At the technical level, it is necessary to integrate digital governance tools and use the digital resource allocation platform to allocate resources reasonably.

**Acknowledgments**

This work was supported by the National Social Science Fund Project:*“* Mechanisms, Effects, and Countermeasures of Rural Settlement Spatial Restructuring on Urban-Rural Population Mobility” (22BRK022).

## PHHS25100: RESEARCH ON THE INTEGRATION PATH OF AESTHETIC EDUCATION AND PSYCHOLOGICAL COUNSELING FOR COLLEGE STUDENTS IN THE DIGITAL AGE

Jian He ^a^

^*a*^ *Wuzhou University, Wuzhou 543002, Guangxi, China.*

**Background:** By delving into the symbiotic relationship between aesthetic education and psychology, we uncover that aesthetic education serves not merely as a balm for the soul but as a catalyst for the holistic growth of college students, nurturing their creative faculties and honing their critical thinking skills. Concurrently, it offers a sanctuary where students can freely express and purge their emotions, easing the burdens of stress and anxiety, and fostering a heightened sense of self-worth and confidence, thereby bolstering their overall competence and competitive edge. **Subjects and Methods:** This study centers on the contemporary college student demographic, employing a dual methodology of literature review and empirical investigation to dissect the overt manifestations and underlying triggers of mental health challenges in the digital era. Presently, students are lavishing excessive emotional investment and vigor upon their virtual personas, blurring the lines between the digital and the real, which engenders confusion over their digital identities and, in turn, impacts their self-perception and mental well-being. Furthermore, the younger generation of college students is increasingly ensnared by the allure of the seemingly flawless lives showcased by others in the virtual realm, leading to discontentment with their own lives.

**Results:** By harnessing the power of art education to enhance emotional expression and regulation, students are empowered to gain a deeper understanding and mastery over their emotions, thereby elevating their emotional intelligence and equipping them with the tools to navigate life’s myriad challenges and pressures with greater ease. Participation in artistic endeavors not only facilitates emotional expression and regulation but also fosters psychological maturity and development. Leveraging virtual reality technology to deliver immersive aesthetic educational experiences not only deepens students’ comprehension and appreciation of art but also bestows upon them a novel emotional journey, aiding in the alleviation of psychological stress.

**Conclusions:** Aesthetic education, as a potent interventional strategy, offers solace to the emotionally weary, alleviates psychological distress, and propels the comprehensive development of college students, while sharpening their creative acumen and critical thinking prowess. Concurrently, it provides an outlet for emotional catharsis, mitigates stress and anxiety, bolsters self-esteem and confidence, and enhances their overall competencies and competitiveness. Moreover, it cultivates emotional intelligence and social adeptness. Looking ahead, there is a pressing need to further explore the profound integration of aesthetic education and digital technology, with the aim of constructing a diverse and personalized mental health support framework that offers robust protection for the mental health development of college students.

## PHHS25101: INNOVATION STRATEGIES FOR INTANGIBLE CULTURAL HERITAGE PRODUCTS: THE ROLE OF CONSUMER PSYCHOLOGICAL COGNITION AND DIGITAL TECHNOLOGY ADOPTION

Michen Yan^a^, Ying Guo^a^, Changjun Ren^a^, Yaqi Yang^a^, Xia Mu^a^

^*a*^ *Chongqing Institute of Engineering, Chongqing 400056, China.*

**Background:** The sustainable development of intangible cultural heritage (ICH) products relies on continuous innovation to enhance both its cultural significance and economic value. However, ICH innovation faces considerable challenges, including high costs, uncertain market demand, and limited consumer cognition of ICH’s cultural value. Notably, the cognitive gap between ICH’s intrinsic cultural narratives and consumers’ understanding weakens market competitiveness and threatens ICH sustainability. In a complex society, consumers’ psychological cognition is also influenced by their level of mental health. Maintaining a healthy mentality and cognition throughout society plays an important role in the inheritance of intangible cultural resources and cultural values.

**Subjects and Methods:** Digital technology provides new possibilities for ICH product innovation by enhancing consumer cognition, but the high costs hinder digital technology adoption. This study investigates the optimal strategy for ICH inheritors and operators, focusing on how innovation investment and digital technology adoption synergistically improve consumer cognition. We construct a quantitative innovation decision-making model to derive optimal strategies under different scenarios, explicitly incorporating consumer cognition as a mediating variable, also taking into account consumer cognition and mental health status.

**Results:** The findings show that (1) Innovation investment significantly improves market competitiveness by bridging the consumer cognition gap, with stronger innovation capability of inheritors leading to higher product innovation. (2) Digital technology could amplify ICH innovation by improving consumer cognition, but its impact is more significant when operational costs remain low. (3) Operators adopt digital technology only when operational costs fall below a cognition-dependent threshold, making cost-benefit analysis critical for technology adoption. (4) Consumer cognition level interacts with inheritors’ innovation capability and consumer preferences to determine the cost threshold for digital adoption. The psychological health status of consumers is also related to the consumption preferences of the entire society. These insights provide valuable guidance for industry stakeholders in formulating strategies for sustainable ICH development in the digital era.

**Conclusion:** This study demonstrates that enhancing consumer cognition through digital technology adoption is pivotal for achieving sustainable ICH development. The quantitative model reveals three core findings: First, innovation investment directly strengthens market competitiveness by addressing gaps in consumer cognition. Second, digital technologies amplify innovation impacts through improved consumer cognition, but their effectiveness depends on maintaining operational costs below a critical threshold determined by consumer cognition levels. Third, the interaction between consumer cognition and inheritors’ innovation capability dynamically adjusts this cost threshold, enabling feasible technology adoption when cognitive enhancement aligns with cost control. These findings suggest operators must prioritize cognitive-driven innovation strategies, while policymakers should focus on reducing adoption barriers for cognition-enhancing technologies. The psychological health status and preferences of consumers also play an important role in promoting intangible cultural property.

**Acknowledgments:** This work was supported by a project grant from national College Students Innovation and Entrepreneurship Training Program (No. 202312608003X); Chongqing Education Scientific Research Experimental Base: Research and Practice on the Construction of a Collaborative Education System between Applied Undergraduate Universities and Enterprises, and the Integration of Industry and Education (No. JD2024G028); Chongqing Institute of Engineering University-Level Research Platform: Digital Intelligence Economy Collaborative Innovation Center; Open Project of Chongqing Education Scientific Research Experimental Base: Research on the Construction of an Evaluation System for “Dual-Qualified” Teachers in Economics and Management Disciplines (No. 2025KFKT-007).

## PHHS25102: INNOVATIVE TEACHING FOR FUTURE NURSES: THE IMPACT OF NARRATIVE TEACHING METHODS ON EDUCATIONAL OUTCOMES AND PSYCHOLOGICAL STATES OF NURSING UNDERGRADUATES

Rong Wang^a,b^, Jianan Zhu^a^, Min Liu^a^, Leonora H. Astete^b^

^*a*^ *Health and Medical College, Nantong Institute of Technology, Nantong, Jiangsu, China,* ^*b*^ *Lyceum of the Philippines University, 33 Muralla St. Intramuros Manila. Philippines 1002.*

*RW and JZ contributed equally to this study.*

**Background:** The purpose of this study is to explore the relationship between narrative teaching strategy, learning effect and psychological state of nursing undergraduates in Nantong Institute of Technology. By emphasizing elements such as professional identity, clinical competence, critical thinking, academic achievement, and student engagement.

**Subjects and Methods:** The data of 322 nursing students were analyzed by standardized questionnaire. The survey assessed the impact of narrative teaching methods on five aspects of nursing students’ psychological educational outcomes, such as the development of nursing students’ psychological needs, clinical communication skills, critical thinking skills, student willingness to engage, and professional identity. These aspects include relevance of content, engagement and interaction, stimulation of critical thinking, practical application and teacher-student interaction.

**Results:** The results show that the narrative teaching method is rated as “efficient” in all dimensions, the overall weighted mean (WM) is 4.64, and the standard deviation (SD) is 0.631. The highest ratings were for content relevance (WM=4.66) and teacher-student interaction (WM=4.64). In addition, nursing students’ psychoeducational outcomes were positively affected, with WM for professional identity formation at 4.53 (SD=0.761) and WM for student engagement at 4.45 (SD=0.806), both reflecting the highest scores. Statistical analysis showed no significant difference in results by age or sex; However, significant differences appeared in the assessment of teaching methods at different grades (p=0.048). In addition, there was a strong positive correlation between narrative teaching methods and educational outcomes, and Spearman’s correlation coefficients were (rs=0.778) and (p<0.001).

**Conclusions:** The findings highlight the importance of integrating narrative teaching methods into nursing education to improve nursing students’ psychological needs, clinical communication skills, critical thinking skills, student willingness to engage, and professional identity. Recommendations include adjusting the complexity of the narrative to the student’s academic level, fostering inclusivity, and implementing active learning strategies. Future research should explore the long-term effects of narrative teaching on professional practice and nursing student outcomes.

**Acknowledgements**

This paper was supported by a project 2024 National College Student Innovation Training Program Project (202412056009Z); Key Discipline Construction Project of Nantong Institute of Technology (879005); Jiangsu Province Education Science Planning Key Project (B/2022/01/151); General Topics of Jiangsu Higher Education Society (2024GZTX098); 2024 General Project of China Business Economics Association (20252096).

## PHHS25103: THE PSYCHOLOGICAL THERAPY IN THE FIELD OF YAO ETHNIC ICONOGRAPHIC PAINTING ART

Zhaolin Lu^a^, Ye Fu^a^, Yuanfeng Liu^b^

^*a*^ *School of Art and Media, Suqian University, Suqian, China,* ^*b*^ *Guangxi Normal College of Nationalities, Chongzuo 532299, Guangxi, China.*

**Background:** For a long time, the human totems in Yao ethnic iconographic paintings have deeply influenced the concepts and consciousness of the Yao people, becoming a part of their folk culture passed down through generations. Yao ethnic iconographic paintings are essential ritualistic tools in the folk beliefs of the Yao people, full of mystery, and their unique artistic forms can meet the psychological needs of various individuals. As a divine tool in the Yao people’s totem worship culture, these paintings still serve as the carriers of psychological therapy in the hollowed-out Yao villages. However, with the impact of modern culture, the hollowing out of Yao villages has become increasingly severe, and many Yao folk cultures, including iconographic painting, are facing fragmentation.

**Research Subject and Method:** In terms of subjects and methods, Yao ethnic iconographic paintings enable individuals and groups in the field to find spiritual belonging in aspects such as psychological cognition, psychological identification, and psychological emotion, fulfilling their psychological needs and achieving psychological repair and therapy. This article takes Yao ethnic iconographic painting as the primary carrier of the field and the psychological space interaction, aiming to achieve psychological therapy. Through this interaction, a dialogue is established between the divine figures in the paintings and the psychological needs of the subject, and psychological therapy in the field is realized through intersubjectivity.

**Results:** Yao ethnic iconographic paintings have extremely high functional value in the context of the Yao people’s folk ritual beliefs. Their unique heritage, artistic techniques, ethnic cultural attributes, and spatial definitions make them more than just folk art and paintings—they can also serve as therapeutic tools. The various gods and totemic figures in the paintings are effective remedies for psychological therapy and spiritual comfort in the field of space, with the ritual space acting as a “hospital.” The shaman performing the rituals acts as the doctor, realizing psychological therapy for the audience.

**Conclusions:** The discussion concludes that hanging Yao ethnic iconographic paintings in the ritual activities of the Yao people’s folk beliefs can elevate the spirits of the Yao people in specific fields, achieving psychological therapy and benefiting their physical health. The “medicinal” role of the field space should be fully utilized to achieve the spiritual restoration of psychological needs.

**Acknowledgments**

This article is a phased outcome of National Social Science Fund project “Research on the Narrative of the Chinese Nation Community in Nuo Opera of Southwestern China” (24XMZ063).

## PHHS25104: EXPLORING THE MEDIATING ROLE OF IDEOLOGICAL AND POLITICAL EDUCATION IN THE RELATIONSHIP BETWEEN INTERNET PENETRATION RATE, EDUCATION FUNDING, AND STUDENTS’ PSYCHOLOGICAL HEALTH

*Jinghao Huanga*

^*a*^ *Hohai University, Nanjing, Jiangsu 210000, China.*

**Background:** This study focuses on deeply exploring and verifying the relationship between the Internet and education in China, which is crucial for the Internet to promote the better development of education. As a result, the mental health of students and staff is enhanced as well as social cognition.

**Subjects and Methods:** The principal components were extracted separately using Factor Analysis. In this way, analyze the impact on mental health. First, the Internet Factor Analysis, using the method of KMO and Bartlett’s Test, to verify that the data can be applied to the method of Factor Analysis. Five male factors were extracted for the 11 variables using spss software. A Scree Plot was drawn to determine the selection of 1 factor to be used to reflect the trend of Internet development. Further by Total Variance Interpretation and Component Matrix Analysis. Next, the Education Factor Analysis was conducted by combining multiple variables. The rotation converges after 7 iterations according to Kaiser’s normalization of the orthogonal rotation method. The weighting model can be obtained through the factor score equation. F1 is finally ranked first. Finally, the relationship between the two was analyzed using Pearson, Spearman, and Partial Correlation. This innovative method can discover the determinants as well as the combination of the two. And this combination contributes to the study of mental health of students and staff. At the same time, this approach increases social awareness.

**Results:** The “Broadband Access Port” was found to be the Principal Component among the many influencing factors of the Internet. Factor Analysis was used to find that the Principal Component affecting education is “General Public Budget for Education”. And two factors help promote mental health. Suffice it to say that these two factors are the main factors in the penetration of the Internet as well as in the development of education as a way of expanding social cognition. Besides, it is found that “Broadband Access Port” is positively correlated with “General Public Budget for Education”. Further explanation. These two contribute to the study of mental health of students and faculty members.

**Conclusions:** It is inferred that there is a positive correlation between Internet penetration and education funding in China. This is an important reference value for researching the matching between Internet penetration and the amount of education funding. On the one hand, validate the theoretical support for the symbiotic development of China’s “Internet Plus” and education. On the other hand, it can precisely identify a factor that determines Internet penetration and a factor that determines the development of education. The two are positively correlated, bridging the research gap in this area. This can make the specific combination of the two to play a better effect. Not only does it enhance the mental health of the subject, but it also expands social cognition.

**Acknowledgements**

Thank you Hohai University for your support.

## PHHS25105: CONSTRUCTION OF A NEW CLINICAL DIAGNOSIS AND TREATMENT PATH FOR PLASTIC SURGERY ASSISTED BY AI TECHNOLOGY

Xiuyu Liang^a^, Xianglin Dong^a^

^*a*^ *Department of Plastic Surgery, the First Affiliated Hospital of Xinjiang Medical University, Urumqi 830000, Xinjiang, China.*

**Background:** The application of artificial intelligence (AI) in the medical field has not only changed the mode of medical services but also improved the quality of healthcare. AI can integrate medical data and construct clinical knowledge bases, providing doctors with diagnostic and treatment suggestions as well as personalized treatment plans for patients. In particular, during surgical diagnosis and treatment, AI can help doctors make diagnoses more quickly and accurately, promoting the shift of surgical care from experience-driven to precision-based and personalized. This is especially true for plastic surgery, which highly depends on visual diagnosis, surgical planning, and postoperative aesthetic evaluation. If the diagnostic and treatment process of plastic surgery is organically integrated with AI’s image analysis and predictive capabilities, it will certainly enhance the effectiveness of plastic surgery treatment and patient satisfaction. Therefore, exploring new pathways and models for clinical diagnosis and treatment in plastic surgery under the assistance of AI has significant theoretical research importance and far-reaching clinical practice value. The application of artificial intelligence (AI) in the medical field has not only transformed the model of healthcare services but also improved the quality of medical care. AI can integrate medical data and build clinical knowledge bases, providing doctors with diagnostic and treatment recommendations and offering patients personalized treatment plans. Particularly in surgical diagnostics and treatment, AI enables doctors to make faster and more accurate diagnoses, advancing surgical care from being experience-driven to becoming precise and personalized. This is especially true for plastic surgery, which heavily relies on visual diagnostics, surgical planning, treatment, and postoperative aesthetic outcome evaluation. By organically integrating the diagnostic and treatment processes of plastic surgery with AI’s image analysis and predictive capabilities, it will undoubtedly enhance the effectiveness of plastic surgery treatments and increase patient satisfaction post-treatment. Therefore, exploring new pathways and models for AI-assisted clinical diagnostics and treatment in plastic surgery holds significant theoretical research value and profound clinical practical importance.

**Subjects and Methods:** Through literature analysis and case studies, this research sorts out relevant publications on the application of AI technology in clinical plastic surgery diagnosis and treatment, and analyzes and summarizes the traditional pathways of plastic surgery diagnosis and treatment based on clinical practice cases.

**Results:** A new AI-assisted plastic surgery diagnosis and treatment pathway was constructed, comprising “preoperative evaluation—simulation and outcome prediction—intraoperative navigation—real-time monitoring and feedback—postoperative recovery prediction—postoperative monitoring and follow-up—effect evaluation and personalized treatment plan formulation.” A vision for a new model was proposed, covering interdisciplinary collaboration ecosystems for AI-assisted plastic surgery, establishing an evidence-based medical assessment framework, and developing AI quantification tools suitable for clinical plastic surgery scenarios.

**Conclusions:** This approach enhances the efficiency of diagnosis and treatment in plastic surgery, ensures clinical accuracy, greatly safeguards treatment quality, reduces diagnostic errors, and lessens the workload of physicians, thereby further harmonizing the doctor-patient relationship.

## PHHS25106: THE STATUS QUO AND EDUCATIONAL COUNTERMEASURES OF VOCATIONAL MATURITY AND CAREER SOCIA SUPPORT OF HIGHER VOCATIONAL GRADUATES

Shuxin Zheng^a^, Chengjie Lv^b^

^*a*^ *Department of Digital Equipment, Jiangsu Vocational College of Electronics and Information, Huai'an, Jiangsu 223003, China,* ^*b*^ *School of Mathematics and Statistics, Lanzhou University, Lanzhou, Gansu 730000, China.*

**Background:** Vocational maturity is an important indicator of whether an individual can successfully complete the development tasks at different stages of career development. The higher the vocational maturity, the clearer the career planning, the higher the execution, and the more appropriate career decisions can be made. Career social support refers to the material, spiritual, suggestion and information support and help provided by parents, teachers or relatives and friends. Understanding the current vocational maturity of higher vocational college students and the status quo of their career social support can provide effective reference for career planning guidance and employment guidance.

**Subjects and Methods:** This paper investigates the status quo of vocational maturity and career social support of 1878 higher vocational graduates from 8 colleges in Jiangsu Province, and understands the overall situation and intra-group differences of vocational maturity and career social support.

**Results:** Through the analysis of effective questionnaire data, it is found that higher vocational graduates have high vocational autonomy and lack of vocational confidence; the main sources of career social support for higher vocational graduates are parents, teachers and classmates and friends. The career social support obtained is mainly emotional support and information support, and more specific suggestion support and material support are less. The dimensions of vocational maturity and the total score of maturity of higher vocational graduates are significantly correlated with the dimensions of social career support and the total score of support.

**Conclusions:** The overall vocational maturity of higher vocational graduates is not high, and there is a phenomenon of high vocational autonomy, but lack of vocational confidence. The main sources of career social support for higher vocational graduates are parents, teachers and classmates, and the career social support obtained is mainly emotional support and information support, and more specific suggestion support and material support are less. The main factors affecting the vocational maturity of higher vocational graduates at the level of social career support are spiritual support, material support and suggestion support. Creating a self-confident and self-reliant cultural atmosphere of vocational education, innovating a precise career planning model and coordinating a friendly employment ecological environment can help higher vocational graduates improve their vocational maturity.

## PHHS25107: DECONSTRUCTING MULTILAYERED LINKAGES: A STUDY ON FAMILY EDUCATION LEVELS, HEALTHCARE RESOURCE ALLOCATION, AND CHILDREN’S HEALTH OUTCOMES IN RURAL CHINA

Wenyi Xu^a^, Xiaoli Yi^a^, Ronghui Liu^a^, Xiaochan Li^a^

^*a*^ *Huaihua normal college, Hunan, China.*

**Background:** Rural China faces persistent disparities in children’s health outcomes, yet the interplay between family capital dimensions and systemic healthcare inequities remains under-explored. This study deconstructs the multilayered linkages among family education levels, healthcare resource allocation, and pediatric health metrics to address critical knowledge gaps in health inequality mechanisms.

**Subjects and Methods:** Utilizing nationally representative data from the China Family Panel Studies (2016-2020) encompassing 302,588 households across 28 rural provinces, we employed a hierarchical regression model with propensity score matching to analyze structural relationships. The analytical framework integrated objective health metrics (stunting rates, BMI-for-age Z-scores, vaccination completeness) with perceptual indicators from caregiver reports. Multilevel modeling accounted for village-level healthcare infrastructure variables, including physician density and primary care facility coverage.

**Results:** Key findings reveal significant child health implications: (1) Healthcare resource utilization efficiency (β= 0.264***) demonstrated stronger predictive power for reducing childhood stunting (OR=0.87, p<0.001) than subjective satisfaction measures, establishing a “practice-perception” paradox. (2) Maternal education exerted dual effects: each additional schooling year decreased children’s nutritional deficiency risk by 11.2% (β= 0.299***) through health literacy enhancement, while mediating 34.7% of healthcare resource allocation effects. (3) Family economic environment contributed 5.9% incremental explanatory variance (ΔR²=0.059), exhibiting threshold effects where household income exceeding ¥86,500 annually amplified health benefits by 27.3% (p<0.01). Notably, children in families with integrated capital advantages showed 41% lower morbidity rates compared to single-capital households.

**Conclusions:** This study advances the “Three-Ring Capital Model” that conceptualizes family capital as interacting educational, healthcare, and economic spheres. The model reveals synergistic effects where combined capital types yield 2.3-4.1×greater health returns than isolated factors. These findings provide empirical support for targeted poverty-alleviation policies and suggest that interventions combining maternal education programs with healthcare system reforms could maximize child health improvements. Future research should explore temporal dynamics of capital accumulation and their inter-generational health impacts.

**Acknowledgements:** This work was supported by a project grant from Research on the Sustainable Development Strategies of Preschool Education Majors under the Background of Negative Population Growth“ [XJK24BGD049] in the “14th Five-Year Plan” for Educational Sciences in Hunan Province. & Research on the Development of Inclusive Kindergartens in Rural Areas under the Background of Population Changes” [XJK24CJC008] in the “14th Five-Year Plan” for Educational Sciences in Hunan Province.

## PHHS25108: RESEARCH ON LIGHTWEIGHT USV SEGMENTATION ALGORITHM FOR MENTAL HEALTH FRIENDLY WATER ENVIRONMENT

Xiaoli Yu^a,b^, Baiyang Wang^b^, Xingping Ran^a,b^, Hongjun Wang^b^, Fang Li^a^

^*a*^ *School of Information Engineering, Changji University, Changji Hui Autonomous Prefecture, Changji 831100, China,* ^*b*^ *School of Information Science and Engineering, Shandong University, Qingdao, Shandong 266237, China.*

**Objective:** By enhancing the environmental perception capabilities of unmanned surface vessels (USVs) in inland river scenarios, we aim to promote their application in complex inland waterways and establish a water ecological working environment that supports the mental health of crew members.

**Subjects and Methods:** Crew members engaged in surface operations under extreme conditions—such as severe weather, polluted areas, or military conflict zones—face significant risks to personal safety and experience considerable mental health stressors. Unmanned Surface Vehicles (USVs), with their advantages of low cost, reduced risk, and effective concealment, are increasingly utilized for pollution control, ecological restoration, water surface rescue operations, and other applications. It is crucial to ensure both the mental well-being of crew members and the reliability of water ecological monitoring systems by substituting high-risk tasks with automated USV solutions. However, existing vision systems for USVs encounter numerous challenges within dynamic inland river environments. These include chaotic riverbanks, reflective surfaces, and fog interference that diminish the accuracy of water body segmentation. Furthermore, current segmentation models exhibit high computational complexity which complicates real-time deployment on resource-constrained unmanned vessels. To address these issues, we propose a novel architecture named DeepLabv3+ Mini. For initial feature extraction purposes, we employ transfer learning utilizing MobileNetV2 as our backbone model. We have designed an enhanced Spatial Pyramid Pooling (ASPP) module that incorporates depthwise separable convolution techniques. Additionally, we have integrated efficient channel attention (ECA) and spatial group enhancement (SGE) modules to bolster feature representation while preserving computational efficiency.

**Results:** In comparison to baseline networks, our optimized model has successfully reduced parameters by 193.94 MB while simultaneously improving segmentation accuracy; specifically increasing mean Intersection over Union (mIOU) by 3.36%, mean Pixel Accuracy (mPA) by 1.77%, and overall accuracy by 1.72%. Experimental results show that the algorithm has significantly enhanced the ability to capture shoreline boundaries in a variety of complex scenes. DeepLabv3+Mini achieves an effective balance between lightweight design and segmentation accuracy, which can support the analysis of water environment of unmanned ships in real time, thus promoting the application of unmanned ships in inland river scenes, further reducing crew workload and easing crew anxiety.

**Conclusions:** The DeepLabv3+Mini algorithm proposed in this study successfully solved the computational limitation of water body segmentation of unmanned ships in complex inland environments by optimizing the model structure and introducing efficient feature extraction and enhancement modules, and significantly improved the segmentation accuracy and robustness. The algorithm can realize real-time deployment on resource constrained unmanned ships, and provides reliable technical support for water environment monitoring of unmanned ships. By reducing the need for crew members to work in dangerous environments, this study provides an important guarantee for maintaining a water ecological monitoring system beneficial to crew members’ mental health, and lays a solid foundation for the wide application of unmanned ships in inland river scenes.

**Acknowledgments**

This research was funded by the following projects from Changji University: the Key Laboratory of Artificial Intelligence and Computational Power Applications, and the Talent Special Project on Illegal Signal Detection and Recognition.

## PHHS25109: ANALYSIS OF THE APPLICATION EFFECTS OF HUMANISTIC CARE IN COLON POLYP TREATMENT UNDER ENDOSCOPY

Jilin Li^a^, Ying Sun^a^

^*a*^ *Department of Gastroenterology, Hohhot First Hospital, Hohhot, Inner Mongolia, China.*

**Objective:** This study aims to investigate the clinical effects of adding humanistic care intervention during the treatment of colon polyps under endoscopy, in order to optimize the overall treatment experience and effectiveness for patients.

**Methods:** A total of 200 patients with colon polyps, who were treated at our hospital from June to December 2023, were selected as research subjects. Patients were divided into a control group and a study group, each consisting of 100 patients, using a random number table based on their admission order. The control group received routine diagnostic and nursing procedures, while the study group received additional humanistic care interventions. These interventions included training medical staff in humanistic care theory and practice, creating a comfortable and private medical environment, providing personalized psychological support, giving detailed pre-treatment explanations, intraoperative monitoring and communication, postoperative care and guidance, and discharge education. The Hamilton Anxiety Scale (HAMA), Hamilton Depression Scale (HAMD), Verbal Rating Scale-10 (VRS-10) for pain, and nursing satisfaction were compared between the two groups before and after treatment.

**Results:** After the intervention, the study group had significantly lower HAMA and HAMD scores than the control group (P < 0.05). The study group’s VRS-10 pain scores were also lower than those of the control group (P < 0.05). Moreover, the nursing satisfaction rate in the study group was 96.00% (96/100), which was significantly higher than the 80.00% (80/100) in the control group (P < 0.05).

**Conclusion:** Implementing humanistic care intervention during colon polyp treatment under endoscopy can effectively improve patients’ negative emotions, restore their mental health, enhance treatment outcomes, reduce psychological and physical pain, increase the pain threshold, relieve subjective pain caused by psychological stress, improve the efficiency and quality of medical care, and enhance patient satisfaction.

## PHHS25110: CHALLENGES IN THE INTERNATIONAL COMMUNICATION OF BAYU CULTURE: CULTURAL DISCOUNT, ACCEPTANCE, AND STRATEGIC ANXIETY

Aining Tang ^a^

^*a*^ *Chongqing University of Arts and Sciences, Yongchuan, Chongqing 402160, China.*

**Objective:** Bayu culture, a distinctive cultural system rooted in the Chongqing region, encompasses language, cuisine, folk traditions, and artistic expressions. However, in the process of international communication, Bayu culture faces significant challenges, particularly due to cultural discount, which affects its acceptance and recognition abroad. Additionally, the anxiety surrounding communication strategies and audience reception further complicates its global dissemination. This study aims to analyze the impact of cultural discount on Bayu culture’s international transmission and propose effective strategies to enhance its global visibility and acceptance.

**Subjects and Methods:** This study examines the challenges Bayu culture faces in international communication, focusing on cultural discount and communication anxiety. Key research areas include its linguistic, artistic, and symbolic elements, as well as their reception by global audiences. Using a mixed-method approach, the study integrates cultural discount theory, cross-cultural communication, and media adaptation. Data sources include linguistic corpora, social media interactions, and surveys measuring cultural acceptance and communication anxiety. Through corpus analysis and case studies, the study explores issues like linguistic adaptation, symbolic misinterpretation, and contextual loss, proposing strategies to enhance Bayu culture’s global reach.

**Results:** The study found that cultural discount significantly affects the international dissemination of Bayu culture, primarily in four aspects: language barriers, cultural cognitive differences, cultural estrangement, and media dissemination limitations. Firstly, Chongqing dialect, as a key symbol of Bayu culture, faces translation challenges in cross-cultural communication. Its unique phonetics and expressions are difficult for target audiences to fully comprehend, leading to cultural information loss. Secondly, cultural cognitive differences result in diverse interpretations of Bayu culture, weakening the consistency of cultural dissemination and affecting the shaping of its cultural image. Thirdly, cultural estrangement causes international audiences to feel unfamiliar or even resistant to Bayu culture’s lifestyle and culinary habits, further hindering effective cultural communication. Lastly, the one-way communication model of traditional media and the insufficient utilization of emerging media reduce the interactivity and precision of Bayu culture’s global outreach, limiting the effective use of digital platforms for dissemination.

**Conclusions:** Overcoming the cultural discount effect in the international communication of Bayu culture requires a comprehensive approach. Key strategies include enhancing cultural adaptation and localization to align with global audiences, improving translation accuracy, and refining symbolic representation to maintain cultural integrity. Additionally, optimizing media strategies, integrating traditional and new media, and fostering interactive cross-cultural engagement can reduce communication anxiety and enhance Bayu culture’s acceptance and influence on the global stage.

## PHHS25111: RESEARCH PROGRESS ON JOB BURNOUT OF COLLEGE COUNSELORS

Hongwei Wang^a^, Xiangui Bu^a^

^*a*^ *Shandong sport university, Jinan 250102, Shandong, China.*

**Background:** Job burnout among college counselors has become an important research topic due to its increasing prevalence and impact on their effectiveness in student management and support. This study aims to review the current research on job burnout among college counselors, exploring its causes, influencing factors, and coping strategies. The goal is to provide a comprehensive understanding of this issue and offer insights into potential solutions, while highlighting directions for future research in this area.

**Subjects and Methods:** This article reviews existing research on job burnout among college counselors from recent years. It synthesizes studies that focus on the various causes of burnout, its contributing factors, and the consequences it has on counselors’ mental health, work performance, and overall job satisfaction. The review also examines the coping strategies that have been proposed to alleviate burnout, such as organizational changes, professional development, and psychological support. The analysis draws from both qualitative and quantitative studies conducted in different educational settings.

**Results:** Research indicates that job burnout among college counselors is primarily driven by a combination of factors. These include heavy workloads, with counselors often responsible for numerous administrative and counseling duties; role conflicts, where counselors experience tension between their educational, caregiving, and administrative responsibilities; and limited career development opportunities, which lead to feelings of stagnation and frustration. These factors contribute to physical and emotional exhaustion, stress, and diminished job satisfaction among counselors. Furthermore, burnout has negative consequences on the quality of their work, which in turn affects student management and the overall operation of the institution.

**Conclusions:** To address job burnout among college counselors, it is crucial to implement targeted interventions. These should focus on optimizing work environments by reducing excessive workloads and providing adequate administrative support, improving career development opportunities, and offering psychological support and training to enhance counselors’ coping abilities. By addressing these issues, colleges can improve counselor job satisfaction, reduce burnout, and enhance the overall quality of student management. Future research should explore the long-term effects of these interventions and continue to examine the evolving needs of college counselors in managing job-related stress.

**Acknowledgments**

This research is the interim result of the 2023 Shandong Provincial Higher Education Undergraduate Teaching Reform Research Project (Project No. M2023349).

## PHHS25112: PRACTICE AND OPTIMIZATION STRATEGIES OF READING PROMOTION SERVICES FOR MINORS IN UNIVERSITY LIBRARIES FROM A PSYCHOLOGICAL PERSPECTIVE

Ying Yang^a^, Qing Li^a^

^*a*^ *Inner Mongolia University of Science and Technology, Baotou, Inner Mongolia Autonomous Region, China.*

**Objective:** This study focuses on how university libraries can optimize reading services for minors from a psychological perspective to meet their diverse psychological needs. By analyzing the practices of libraries in terms of reading spaces, resource development, and promotional activities, this research aims to provide theoretical foundations and practical guidance for innovative service models in university libraries. The ultimate goal is to support the mental health and comprehensive development of minors.

**Subjects and Methods:** Taking the Inner Mongolia University of Science and Technology Library as a case study, this research employs a case analysis method combined with reading psychology theory to examine its practices in serving minors. These practices include the establishment of a children’s reading room and the “Yue Shu Ba” reading base, the development of a book resource system tailored to the psychological needs of different age groups, and a series of public welfare activities such as “Expanding Beyond Campus, Bringing into Campus.” The study integrates qualitative and quantitative data to evaluate the effectiveness of these initiatives in fostering reading interest and mental well-being among minors.

**Results:** The study finds that university libraries can effectively stimulate minors’ interest in reading, cultivate good reading habits, and promote their mental health development by creating reading spaces that align with the cognitive development characteristics of minors. The construction of a book resource system that caters to the psychological needs of various age groups has proven to be particularly impactful. Additionally, the implementation of diverse reading promotion activities, such as interactive workshops, storytelling sessions, and community outreach programs, has significantly enhanced minors’ engagement with reading and contributed to their emotional and cognitive growth.

**Conclusions:** The research concludes that university libraries should actively seek collaborations with educational institutions, psychological experts, and community organizations to integrate resources and expertise. By paying close attention to the reading psychology of minors and continuously innovating service models, libraries can achieve both depth and breadth in their services. This approach will ensure that minors receive high-quality reading services that support their mental growth and holistic development. Furthermore, the study emphasizes the importance of ongoing evaluation and adaptation of library services to meet the evolving needs of minors in a rapidly changing educational and social landscape.

**Acknowledgments:** The successful completion of this article is inseparable from the support and help of many parties. First of all, thanks to all the librarians and volunteer teams of the library of Inner Mongolia University of Science and Technology, who have worked hard in the practice of juvenile service and provided rich practical cases and data support for this article. At the same time, thanks to the cooperation institutions inside and outside the school, including communities, primary and secondary schools and public libraries, their active participation has injected vitality into the construction of the service system for minors in university libraries. In addition, This work was supported by the funding of the basic scientific research business fee project of colleges and universities directly under the Inner Mongolia Autonomous Region in 2024: ‘Practical Research on Reading Promotion of University Libraries under the National Cultural Digitization Strategy’ (Project Number: 2024QNJS144, Project Leader: Ying Yang) (Internal Finance Education Regulation No.11), which provides a solid academic support for the research of this paper. Finally, thanks to all the teachers, parents and minor readers who participated in the activities, their feedback and suggestions provide valuable reference for the improvement of this article. I would like to express my sincere thanks!

## PHHS25113: BIDIRECTIONAL ASSOCIATION OF SUBJECTIVE LIFE-EXPECTANCY WITH DEPRESSION AMONG RURAL OLDER ADULTS

Lei Chen^a^, Ming Huo^a,b^, Hongzhao Wang^c^, Sixiang Tao^c^, Ailing Wen^c^, Jingtang He^c^, Xiaoyu Wang^c^

^*a*^ *Department of Physical Therapy, Jilin Province Power Hospital, Changchun 130000, China,* ^*b*^ *School of Rehabilitation Sciences and Engineering, University of Health and Rehabilitation Sciences, Qingdao 250002, China,* ^*c*^ *Department of Physical Education, Huainan Normal University, Huainan 232038, China.*

**Objective:** Depression in the elderly is associated with low socioeconomic status, low social support, poor health status, disability, adverse life events as well as female gender, and it is a significant health and economic burden. Both longevity and depression are associated with indicators of multiple social determinants of health. The association of Subjective life expectancy (SLE) with depression has been well reported. However, the relationship between SLE and depression is still controversial. The aim of this study was to investigate whether there is a mutually causal relationship between SLE and depression.

**Methods:** The bidirectional randomization analysis was performed using data from the China Health and Retirement longitudinal Study (CHRLS) in rural older adults.

**Results:** Low life expectancy increased the risk of depression, and the risk of low life expectancy increased to 117% (OR [95% CI1.17 [1.05-1.30], p<0.01) compared to the group with high life expectancy after controlling for socio-demographic factors, health-related behavior factors, and health-function factors. Depression increased the risk of low life expectancy, and the risk of depression increased to 120% (OR [95% CI 1.20 [1.08-1.34], p<0.01) compared to the group with non-depression group after controlling for socio-demographic factors, health-related behavior factors, and health-function factors. Furthermore, the restricted cubic spline (RCS) regression models of the life expectancy and depression showed that there is a linear relationship, and depression score and life expectation value showed an approximate horizontal “S” pattern, and the curve showed an overall downward trend.

**Conclusion:** There is a bidirectional causal relationship between life expectancy and depression, but the corresponding trend is different. The present findings highlight the importance of considering both factors in the development of health promoting prevention programs or improving overall mental health among rural older adults. Further longitudinal research should further longitudinal studies should be conducted to dig deep into the relationship between SLE and depression, and vice versa.

**Acknowledgments**

This work was supported by Scientific Research Foundation of Education Department of Anhui Province of China (Grant No. 2023AH051509); National Natural Science Foundation of China (Grant No.82372585).

## PHHS25114: THE DIGITAL RESOURCE CURSE: SOCIOECONOMIC STATUS AMPLIFIES BEHAVIORAL HEALTH RISKS IN PRESCHOOLERS VIA MALADAPTIVE SCREEN USE

Ronghui Liu^a^, Xiaoli Yi^a^, Wenyi Xu^a^

^a^ *Huaihua Normal College, Hunan 418000, China.*

**Background:** This study investigates how family socioeconomic status (SES) influences behavioral health outcomes in preschoolers in the digital era, focusing on the dual mediating roles of screen dependency and parenting environments. By integrating neurobiological markers (cortisol levels, prefrontal oxygenation) into the analytical framework, we aim to provide evidence-based strategies for mitigating technology-related developmental risks in high-SES populations.

**Subjects and Methods:** A nationally representative sample of 1,092 Chinese families with preschoolers (mean age=54.2 months, SD=11.3) was recruited via stratified cluster sampling across six provinces. SES was operationalized as a latent variable combining log-transformed annual income, fixed assets, and educational expenditure (CFI=0.984, RMSEA=0.035). Child behavioral health was assessed using the Child Behavior Checklist 1.5-5 (α=0.92), while screen dependency was measured via a validated 15-item scale capturing loss of control, withdrawal symptoms, and functional impairment (α=0.83). Parenting environments were evaluated using the 28-item Digital Parenting Ecology Scale (α=0.91) assessing emotional responsivity and educational engagement. Structural equation modeling with 5,000 bootstrap iterations tested chain mediation effects, controlling for urbanicity, parental education, and child age.

**Results:** SEM revealed three critical pathways linking SES to behavioral health outcomes: Direct Health Risk: Higher SES directly increased behavioral problems, particularly emotional dysregulation and social withdrawal. Screen-Mediated Pathway: A significant “parenting environment—screen dependency” chain mediation accounted for 20.8% of total effects. High-SES families with <30% educational screen content faced 2.71-fold greater problematic screen use risk. Parenting Paradox: Intensive parenting involvement (1 SD increase) unexpectedly elevated sleep disturbances (β=0.41, p<0.001) and attention deficits (β=0.33), mediated by heightened parent-child conflict during digital co-use.

Biomarker Evidence: Screen dependency correlated with elevated evening cortisol (β=0.27, p=0.002), reduced prefrontal oxygenation during cognitive tasks (β=-0.31, p<0.001), and delayed sleep onset latency (β=0.19, p=0.007).

**Conclusions:** This study uncovers a “digital resource curse” mechanism where economic advantage paradoxically exacerbates behavioral health risks through maladaptive screen use. The findings provide neurobiological evidence for targeted interventions, highlighting the need to prioritize screen content quality over device access in high-SES families.

**Acknowledgements**

This work was supported by a project grant from Research on the Development of Inclusive Kindergartens in Rural Areas under the Background of Population Changes“ [XJK24CJC008] in the “14th Five-Year Plan” for Educational Sciences in Hunan Province.

## PHHS25115: IMPROVED SALP SWARM ALGORITHM AND ITS APPLICATION IN MEDICAL IMAGE PROCESSING

Hongwei Zhao^a^, ChangLi Dong^a^

^*a*^ *School of Information Engineering, Shenyang University, Shenyang 110044, China*

**Background:** Medical image processing is crucial for accurate diagnosis and treatment planning in modern healthcare. However, it faces challenges such as high computational complexity, significant resource consumption, and stringent real-time requirements. Swarm intelligence algorithms, such as Particle Swarm Optimization (PSO) and Ant Colony Optimization (ACO), have been widely used in medical image segmentation, feature extraction, and 3D reconstruction. Despite their success, these algorithms often struggle with premature convergence and high-dimensional feature spaces in medical images. This paper proposes a multi-strategy improved Salp Swarm Algorithm (ADLSSA) to address these issues, enhancing the efficiency and accuracy of medical image processing tasks.

**Subjects and Methods:** The proposed ADLSSA algorithm introduces several improvements to the traditional Salp Swarm Algorithm (SSA). Firstly, the Logistics chaotic sequence is used to enhance the diversity of the initial population, preventing premature convergence. Secondly, the differential evolution mutation operation is applied to the leader positions, improving the global search capability for complex medical image segmentation and feature extraction. Additionally, an adaptive inertia weight mechanism is introduced for follower position updates, dynamically balancing local exploitation and global exploration. Based on ADLSSA, a heuristic task scheduling algorithm, HTAA, is proposed for medical image processing. HTAA constructs a multi-level task scheduling model, including image preprocessing, feature extraction, and lesion recognition, and incorporates a timeout penalty function to optimize resource allocation efficiency in edge computing nodes.

**Results:** Simulation experiments were conducted using publicly available medical images, including breast tumor segmentation and 3D reconstruction of lung CT images. The performance of ADLSSA was compared with traditional algorithms such as SSA, PSO, and GA. ADLSSA demonstrated superior performance in terms of computational complexity, convergence speed, and segmentation accuracy. For instance, ADLSSA achieved a Dice coefficient of 0.92 and an IoU of 0.87, outperforming SSA (Dice: 0.85, IoU: 0.78), PSO (Dice: 0.88, IoU: 0.81), and GA (Dice: 0.83, IoU: 0.76). The HTAA algorithm also showed significant improvements in task latency and energy consumption, reducing average latency by 19.3% compared to PSO and total energy consumption by 9.12% compared to TP.

**Conclusions:** The proposed ADLSSA algorithm effectively addresses the challenges of high computational complexity and premature convergence in medical image processing. By enhancing the diversity of the initial population and improving global search capabilities, ADLSSA achieves higher accuracy and faster convergence. The HTAA algorithm further optimizes task scheduling in edge computing environments, balancing latency and energy consumption. These improvements provide theoretical support and technical guarantees for the real-time analysis of medical images and the efficient deployment of smart healthcare systems, ultimately enhancing the accuracy and efficiency of medical diagnosis and treatment.

**Acknowledgements**

This work was supported by a project grant from Northeast Geological Technology Innovation Center District Creation Fund Project (Grand No: QCJJ-2023-49).

## PHHS25116: AI EMPOWERMENT IN PRIMARY SCHOOL MENTAL HEALTH EDUCATION: AN EMPIRICAL STUDY BASED ON THE INTEGRATION OF INTELLIGENT TECHNOLOGY

Hongxia Wang ^a^

^*a*^ *Yan’an New District First Primary School, Yan’an 716000, China.*

**Background:** Mental health is a critical foundation for the holistic development of primary school students, yet mental health issues among this age group have become increasingly prominent. According to statistics from the Ministry of Education, approximately 45% of primary school students in China experience mild to severe anxiety, with writing anxiety and social phobia being especially prevalent. Traditional models of mental health education largely depend on teachers’ experience and standardized curricula, which are often constrained by insufficient resources, delayed interventions, and a lack of personalization. These limitations make it difficult to meet the immediate emotional needs of students. In recent years, the application of Artificial Intelligence (AI) in education has expanded into emotional computing and mental health support, offering new pathways for personalized, real-time psychological assistance. However, existing research has largely focused on higher education, with limited empirical evidence on the effectiveness of AI in primary school settings.

**Research Subjects and Methods:** This study adopts a mixed-methods approach, selecting 120 students from grades 3 to 6 at two primary schools in a city as research subjects. Participants were divided into two groups: an experimental group and a control group, each consisting of 60 students. The experimental group utilized an AI-supported system integrating emotional analysis and dynamic feedback, while the control group continued with traditional mental health education. The study lasted for 12 weeks, during which data were collected using various methods, including questionnaires (anxiety scale and self-efficacy questionnaire), AI log analysis, and semi-structured interviews.

**Results:** The results revealed that students in the experimental group showed a significant 32% reduction in anxiety levels (p<0.01) and a 41% increase in self-efficacy (p<0.05) after receiving the AI intervention. The AI system’s anonymous feedback feature was particularly effective in alleviating social anxiety, with an effect size of 0.48. Additionally, qualitative analysis indicated that the personalized psychological counseling plans generated by the AI system significantly improved students’ emotional regulation capabilities.

**Conclusions:** The findings suggest that AI technology, through dynamic emotional recognition and real-time feedback mechanisms, can enhance the effectiveness of primary school mental health education by providing more personalized and accurate psychological support. However, future research must address issues such as the ethics of technology use and the equitable distribution of resources between urban and rural areas, to ensure the rational application and sustainable development of AI in mental health education. Furthermore, exploring how to further harness AI’s potential and innovate mental health education models will be a key direction for future studies.

## PHHS25117: RESEARCH ON THE PROMOTION OF MENTAL HEALTH BY VIRTUAL REALITY SPACE FROM THE PERSPECTIVE OF ART THERAPY

Wei Chen^a^, Jianli Wang^a^, Hongying Ma^a^, Wanchun Wang^a^

^*a*^ *Shaanxi University of International Trade & Commerce, Xi’an 712046, Shaanxi, China.*

**Background:** In modern society, with the life pace speeding up day by day, work pressure, interpersonal relationship and other factors make people bear unprecedented psychological burden, so that mental health problems become more and more prominent. As an effective psychological treatment method, art healing has gradually entered the public’s eye. With the help of various art forms, it helps individuals release negative emotions, thus relieving psychological pressure, realizing self-regulation and repair of psychological state, and promoting the healthy development of mental health. In recent years, the rise of virtual reality technology has provided a new platform for artistic psychological healing. Its immersion and interactivity have injected new vitality into traditional psychological healing, brought more possibilities, and made the psychological healing process more vivid, interesting and efficient.

**Subjects and Methods:** This study focuses on the practical application of virtual reality space in the field of art psychological healing. Through systematic and comprehensive theoretical analysis, the study extensively involves the related theoretical knowledge of psychology, art healing, virtual reality technology and other disciplines to deeply explore the internal mechanism of virtual reality technology in art healing. By constructing a positive and pleasant virtual environment and combining various art healing forms, different virtual art healing spaces are designed for mental health problems such as anxiety disorder, depressive disorder, post-traumatic stress disorder (PTSD) and social phobia, so as to provide personalized services for the patients.

**Results:** By immersive virtual art activities, virtual scenes and art healing methods designed for different psychological problems can make the patients effectively release their accumulated negative emotions in their hearts, deepen their self-awareness and effectively relieve the symptoms of mental diseases. Finally, the effectiveness of virtual reality art healing was confirmed by professional psychological scale evaluation and daily behavior observation.

**Conclusions:** The deep combination of virtual reality technology and art healing provides innovative solutions for promoting mental health. Despite the challenges of technical cost and equipment comfort, with the technological progress and the deepening of interdisciplinary research, virtual reality art healing is expected to be widely used in the field of mental health, greatly improving the life quality of patients suffering from mental illness and contributing to the construction of a more harmonious and healthy social environment.

## PHHS25118: THEORETICAL RECONSTRUCTION AND PRACTICAL PATHWAYS FOR EMBEDDING PSYCHOLOGICAL HEALTH LITERACY IN THE CULTIVATION OF ENGINEERING INNOVATION TALENTS

Hai Lan^a^, Genyan Liu^a^, Xia Tao^a^, Faquan Yu^a^

^*a*^ *Wuhan Institute of Technology, Wuhan, China.*

**Background:** In the context of the global shift in engineering education from a “technology-centric” model to one focused on “holistic development”, the lack of psychological health literacy has become a critical obstacle to cultivating innovative talent. The current engineering education system typically faces a structural contradiction: it emphasizes “technical training” while neglecting “psychological development”. This issue arises because the traditional training model fails to systematically address three increasingly significant psychological challenges in engineering practice: the decision-making dilemmas under complex pressures, frequent emotional conflicts during team collaborations, and unclear value judgments in technical ethical decisions.

**Subjects and Methods:** This study examines the curriculum systems of engineering institutions and the employment standards in manufacturing enterprises. Based on this, a “psychological-technical” dual-spiral theoretical model is proposed. This model reveals that the core competencies of engineering talent must include three essential psychological dimensions: decision-making ability under pressure, emotional management in teamwork, and ethical value judgment in technical dilemmas.

**Results:** The study identifies a key issue in the traditional training model: the lack of psychological resilience development. This gap results in a structural imbalance between “technical proficiency” and “psychological adaptability” among engineering students. The consequence is the emergence of two major challenges: cognitive rigidity and innovation anxiety when tackling complex engineering problems. Based on this theoretical model, the study outlines a three-stage pathway for reform. At the curriculum level, the introduction of an interdisciplinary module, Engineering Psychology and Ethical Decision-making, is recommended, along with the development of a VR-based engineering stress simulation system to recreate crisis scenarios. At the practical teaching level, the implementation of a “dual-mentor system” for psychological guidance is suggested. Lastly, at the evaluation level, a three-dimensional assessment model should be established, encompassing psychological capital, team collaboration effectiveness, and the quality of ethical decision-making.

**Conclusions:** The study concludes that embedding psychological health literacy into the entire engineering education process can resolve the “technology-psychology” dichotomy. Moreover, it can facilitate the simultaneous development of psychological capital and technical expertise, thus fostering a new generation of engineering professionals who are both innovative and ethically responsible. This approach provides a Chinese solution to the global challenges in engineering education reform.

**Acknowledgements**

This work was financially supported by the following research projects:

1. The Key Project of Hubei Province Educational Science Planning 2023 (Project No. 2023GA044); 2. The Undergraduate Teaching Research Project of Wuhan Institute of Technology in 2024; 3. The 2024 Graduate Education and Teaching Reform Research Project of Wuhan Institute of Technology, titled “Research on the Construction and Application of a Precision Teaching Model for Postgraduate Students Based on Learning Analytics” (Project No. 2024JYXM12).

## PHHS25119: MIDDLE SCHOOL MATHEMATICS CONCEPT TEACHING STRATEGIES BASED ON CHINESE CHARACTER ART FROM THE PERSPECTIVE OF PSYCHOLOGY

Hong Wei^a^, Jingwei Wang^a^, Linyu Bai^a^, Dongling Chen^b^

^*a*^ *School of Mathematics and Statistics, Nanning Normal University, Nanning 530100, China,* ^*b*^ *The College of Teacher Education, Wuzhou University, Wuzhou 543002, China.*

**Background:** In view of the prominent problems of psychological anxiety of junior high school students in learning mathematics and the lagging development of mathematical abstract thinking, as well the phenomenon of mutual exclusion between sensuous literary art and rational mathematical science, this paper attempts to construct a junior high school mathematics concept teaching model based on the psychological characteristics junior high school students from the perspective of psychology, so as to promote the reform and innovation of mathematics teaching.

**Subjects and Methods:** Through a literature review, the current research status of combining Chinese character art with mathematics teaching both domestically and internationally was summarized, clarifying the research direction and starting point. Empirical research methods such as questionnaires, interviews, and teaching experiments were employed to collect a large amount of data, which was then statistically analyzed. By reconstructing the cognitive model through the unique rational framework of mathematics, the vague and perceptual anxiety is into an analytical and operable structured problem, which enhances the sense of control and reduces the emotional response of anxiety. The innovation of the research is to break the traditional boundaries and transform artistic resources into teaching intervention tools. The combination of Chinese character art and the rigorous logic of mathematical concepts cultivates students’ interest in learning and abstract thinking.

**Results:** The research results show that mathematics concept teaching integrated with Chinese character art can significantly improve students’ learning interest and mathematical abstract thinking abilities. The research shows that the level of math anxiety among middle school students is significantly higher that of other subjects (PISA 2022). Students’ anxiety is negatively correlated with the effect of learning mathematics, and it also directly affects the cultivation students’ core qualities such as mathematical abstraction and logical reasoning.

**Conclusions:** The structural pattern and its original meaning of Chinese characters are cognitively isomorphic with the strategy of mathematical problem solving, and the art of characters can guide students to establish structured mathematical thinking and enhance their pattern recognition ability in learning mathematical concepts. As a carrier of culture, Chinese characters can transform the “cold” of mathematics into emotional cognitive objects, which can reduce learning anxiety. Based on the perspective of psychology, constructing concrete situations in the teaching of mathematical concepts enables students to construct knowledge in the situation, which is in line with Piaget’s theory of cognitive development and can enhance students’ mathematical creativity and interdisciplinary thinking.

**Acknowledgments**

This work was supported by a project grant from Guangxi scientific education special project of the “14th Five-Year Plan” in 2023: “Theoretical and Practical Research on the Teaching and Learning of Middle School Mathematics Concepts Guided by Mathematical Abstract Literacy from the Perspective of Chinese Excellent Traditional” (Grant No.2023ZJY748).

## PHHS25120: RECONSTRUCTING THE PARADIGM OF PRIMARY SCHOOL CHINESE WRITING INSTRUCTION IN THE ERA OF ARTIFICIAL INTELLIGENCE: A PRACTICAL EXPLORATION OF THE INTEGRATION OF AI EMPOWERMENT AND MENTAL HEALTH

Baolin Yang ^a^

^*a*^ *Yan’an New District First Primary School, Yan’an 716000, China.*

**Background:** Driven by both the revolution in intelligent technology and the Curriculum Standards for Compulsory Education: Chinese Language (2022 Edition), primary school writing teaching urgently needs to address core issues within traditional models, such as “rudimentary guidance”, “delayed feedback”, and “lack of individuality”. Simultaneously, pervasive challenges related to students’ mental health, including writing anxiety, self-esteem issues, and emotional sensitivity, have further hindered the development of their writing abilities and creativity. This study explores the integration of AI technology and mental health support through the construction of a “Three-Dimensional Intelligent Support System”, aiming to optimize teaching effectiveness and enhance students’ psychological resilience.

**Research Subjects and Methods:** A mixed-methods research design, encompassing design-based research, text analysis, and quasi-experimental design, was employed. The experimental subjects were primary school students from grades 3 to 6 (N=88), divided into an experimental group (N=45) and a control group (N=43). The experimental group utilized an AI support system integrated with mental health interventions, covering content generation (personalized material recommendations), process guidance (real-time feedback and voice writing tools), and evaluative feedback (emotional analysis and digital growth portfolios). Mental health indicators were measured using modified writing anxiety scales (WAS) and self-efficacy questionnaires (SEQ), with qualitative data drawn from teacher-student interviews and teaching logs.

**Results:** The experimental group significantly outperformed the control group in terms of writing quality (ES=0.43), linguistic expression (ES=0.37), and structural organization (ES=0.32). Writing anxiety decreased by 28% (p<0.05), and self-efficacy increased by 35%. Qualitative analysis revealed that AI-facilitated anonymous peer review and dynamic feedback effectively alleviated students’ social pressures, while the voice writing tool helped students with special needs overcome expression barriers. Teachers utilized AI diagnostic reports to accurately identify students’ psychological needs (e.g., perfectionist tendencies) and adjust instructional strategies accordingly.

**Conclusions:** This study validates the synergistic effects of AI technology and mental health interventions, providing a “dual-dimensional support framework” for writing teaching in the intelligent era. Future research should focus on addressing the risks of over-reliance on technology and the digital divide between urban and rural areas. It is recommended to establish a “human-machine collaborative” evaluation standard and develop lightweight tools to ensure educational equity and promote the holistic development of students.

## PHHS25121: SCREEN TIME AND PHYSICAL HEALTH STATUS OF COLLEGE STUDENTS UNDER THE BACKGROUND OF SPORTS AND NURSING AND NURSING COUNTERMEASURES

Yuanyuan Dai^a^, Heshuang Ye^a^, Zhenhong Zhao^a^, Hao Fu^a^

^*a*^ *Physical Education Institute, Chizhou University, Chizhou, Anhui 247100, China.*

**Background:** To explore the independent effect of screen time on physical health of students and the interaction between them, put forward health promotion strategy.

Subjects and Methods: A total of 3467 students from School H in Anhui Province from 2018 to 2021 were studied by physical fitness test to investigate the correlation between screen time and physical health.

**Results:** The average daily screen time of literature and history students and science and technology students was 7.36±3.42h and 7.67±3.69h, respectively, and the screen time of literature and history students was less than that of science and technology students(P<0.05); The average daily screen time of Males and Females was 7.89±3.27h and 7.22±3.32h, respectively, and the average daily screen time of Females was less than that of Males (P<0.01). The average daily screen time of students from 2018 to 2021 was 8.03±2.86h, 8.21±3.17h, 7.45±4.10h and 6.33±2.84h, respectively, with significant differences among different grades(P<0.01).The mean distribution of BMI of Male and Female students were 21.78±2.64 and 21.11±1.80, respectively. The overall excellent rate of lung capacity was 12.3%; the overall excellent rate of standing long jump was 9.9%; the overall excellence rate of the seated forward flexion was 11.8%; the excellent rates of pull-ups (Male) and 1 min sit-ups (Female) were 12.8% and 14.9%, respectively; the overall excellence rate of 50m was 9.5%; the excellent rate of 1000m (Male) and 800m (Female) was 8.2% and 5.8%, respectively. There were significant correlations between screen time and BMI (P<0.01), there were correlations between screen time and lung capacity, standing long jump and 50m (P<0.05).

**Conclusions:** The results show that the phenomenon of excessive screen time is common in H school students in Anhui province, and the higher the grade, the longer the screen time; the overweight and obesity phenomenon of Male is also more serious. The physical function and physical quality of students are barely up to standard. Screen time was significantly associated with BMI, lung capacity, standing long jump, 50m, and it was an important factor in most indicators of physical health. Therefore, in order to enhance the physical health level of college students, we need “three-dimensional” linkage: First, we should exert the influence of social ecological model and monitor their screen time in all aspects as far as possible; Secondly, the screen time experiment intervention is incorporated into the school teaching and research work, and combined with the physical health knowledge, to achieve the integration strategy of physical education; Thirdly, individuals should give full play to their subjective initiative, establish a sense of health, use electronic products reasonably, and reduce ineffective reading and screen time.

**Acknowledgments**

This work was supported by 2 project grant from Key research project of natural science in colleges and universities of Anhui Provincial Department of Education (Grant No. KJ2021A1135); funding project for visiting and studying of young backbone teachers in China (Grant No. JNFX2023071).

## PHHS25122: MENTAL HEALTH-DRIVEN TRANSFORMATION IN CHINESE OCEAN MANAGEMENT BEHAVIOR

Xuechun Lin ^a^

^*a*^ *Shandong Technology and Business University, Yantai, Shandong 264005, China.*

**Background:** To investigate the impact of mental and emotional need to the ocean on the group’s behavior in respect of culture, economy and management. The ocean provides many resource including ocean biology, food, medicine, mine and so on. It is an important component of national territory, regional economy and culture.

**Subjects and Methods:** 11 coastal provinces of China have constructed marine functional zoning. They have high competition anxiety and mental expectation to utilize ocean resources and enhance ocean management. China’s marine economy, culture and people’s awareness and behavior of the ocean are all undergoing positive changes. The self-confidence of industry and social interaction about marine affairs can be recognized. Six major marine economic regions have been formed nationwide, including the Bohai Rim, Yangtze River Delta, Pearl River Delta, Beibu Gulf, Fujian coast, Hainan. The gross domestic product of the ocean accounts for 9.5% of the national GDP, an increase of 7.6% from 2012. The added value of the primary, secondary, and tertiary industries of the ocean accounts for 5.4%, 45.8%, and 48.8% of the gross domestic product of the ocean, respectively, with 35.13 million people employed in the ocean. But the challenges and anxiety to the fragmented ocean management become increasingly apparent, it is urgent to overcome the limitations of fragmented ocean management and utilization in coastal areas and form a kind of healthy ocean production and life.

**Results:** There are many lack and negative effects for the Chinese marine managing and utilizing behaviors with respect of authoritative integration mechanism, personality characteristics and interconnection, leading brands and continuous delivery capabilities. The low-level redundant construction result in that there is no a high-quality coordinated development and characteristic complementarity. The quality, effect, capacity and value of marine services cannot be effectively stimulated and transmitted in the borderless marine governance and sea-land interconnection. These behaviors or problems can’t meet the increasing need of human beings for marine production and services, lead people to feel mental frustrated about marine high-quality development, and reduce the sense of gain and happiness.

**Conclusions:** A healthy and proactive approach should be adopted to create a new ocean value, mental cognition and driving force, consolidate the intrinsic foundation of ocean management and services. The main adjustment method will include construction of the identified ocean discourse system and proactive thinking to remove confusion, the shaping of ocean authoritative institutions to coordinating order, the improvement of ocean governance and behavior capabilities, and the multidimensional integration of marine projects. The deep transformation of marine management will be driven, an open and environment-friendly marine development situation will be the growth goals.

## PHHS25123: INFLUENCE FACTORS OF THE CONSTRUCTION ENVIRONMENT UNDER-BRIDGE SPACE ON RESIDENTS’ WELL-BEING: A CASE OF THE SPACE UNDER THE SHANGHAI ZHONGHUAN OVERPASS

Yuelan Xu ^a,b^

^*a*^ *Xianda College of Economics and Humanities Shanghai International Studies University, Shanghai, China,* ^*b*^ *Suan Sunandha Rajabhat University, Bankok, Tailand.*

**Objective:** With the development of China’s urbanization, the construction environment has become a prerequisite for improving the living comfort of the residents and enhancing their sense of happiness. This paper explores the relationship between the construction environments of Shanghai residents’ subjective well-being, and provides some reference value for the construction of living environment in under-bridge space.

**Methods:** The method of questionnaire and scale statistics was used to conduct an empirical study on the space under the Zhonghuan overpass in Changning District, Shanghai. The questionnaire includes the basic information of the interviewees, personal attributes, and environmental factors of under-bridge space (physical space quality, diversity of public facilities, visual design, social safety, and traffic accessibility). Factor analysis was conducted by principal component analysis, and the influence of each principal factor on well-being of local residents was analyzed by multiple regression.

**Results:** The results show that environmental safety, physical space quality and diversity of public facilities significantly affect villagers’ perception of well-being, while visual design, traffic accessibility and other factors have no significant impact on well-being.

**Conclusions:** Through the exploration of the factors influencing factors of residents’ well-being, this paper believes that the space environment construction under the bridge should be optimized from the aspects of safety, physical space quality and public facilities to meet the needs of residents of all ages in life, such as life, culture and health, so as to effectively improve residents’ well-being. From the above aspects, suggestions are put forward, namely the optimization design of physical space environment. By reasonably organizing the space and functional zoning, opening the entrances and exits, arranging the functional areas according to local conditions, and selecting plants with strong adaptability to improve the interest and landscape features of the space. Safety guarantee design, provide safe street crossing facilities, set up anti-collision facilities to protect the bridge piers, ensure sufficient lighting, install monitoring equipment, and barrier-free design, to ensure the safety and convenience of residents. Public facilities are fully designed. According to the needs of residents, functional areas such as sports fitness, children’s recreation, recreation, and public toilets are set up, cultural exhibitions and art installations are implanted to create cultural and creative space, and the parking lot is reasonably planned to meet the diverse needs of citizens and improve the convenience and well-being of life.

**Acknowledgements**

This work was supported by a project grant from the Research Innovation Platform Project—2025 XianDa College of Economics and Humanities Shanghai International Studies University Chongming Regional Culture Digital Inheritance and Development Research Center, and 2023 Shanghai Key Undergraduate Education Reform Project: Applied Art and Design Talent Cultivation Driven by “National Art Reconstruction” Platform.

## PHHS25124: GENDERED NEUROBIOLOGICAL PATHWAYS OF EDUCATIONAL CAPITAL: PROTECTING PRESCHOOL MENTAL HEALTH THROUGH EMOTIONAL PARENTING AND SCREEN REGULATION

Wenyi Xu^a^, Xiaoli Yi^a^, Ronghui Li^a^, Xiaochan Li^a^

^a^ *Huaihua Normal College, Hunan 418000, China.*

**Background:** Chinese preschoolers face unprecedented mental health challenges, with 22.7% (1-6 years) meeting DSM-5-TR criteria for behavioral-emotional disorders: 16.3% anxiety/depression (internalizing) and 19.1% aggression/ADHD (externalizing) (National Child Mental Health Survey, 2024). Excessive non-educational screen exposure (2.5 hours/day in 1-3-year-olds) correlates with annual prefrontal cortex thinning of 1.8% (p=0.008), yet gender-specific neuroprotective pathways of parental education remain unclear. This study integrates DSM-5-TR diagnoses and fMRI evidence to decode how maternal/paternal education (ISCED-2011) differentially impacts child mental health through emotional parenting (amygdala connectivity) vs. screen regulation (DLPFC morphology).

**Subjects and Methods:** A nationally stratified sample of 1,092 parent-child dyads (774 mothers, 318 fathers; children aged 1-6 years, 54% rural) underwent: Mental Health Assessment: SDQ-China (internalizing/externalizing subscales, teacher-report ICC=0.68)+DSM-5-TR anxiety/ADHD modules Neuroimaging: 3T fMRI measuring amygdala-precuneus connectivity (emotional regulation, n=218) and DLPFC thickness (cognitive control, n=189).Mediators: Parenting Environment Index (PEI, α=0.88; emotional warmth/screen co-regulation) + Screen Dependency Index (α=0.85; 67% non-educational content).

**Results:** Maternal Emotional Protection Pathway: Each ISCED level reduced internalizing symptoms (β=-0.12, p<0.001) via emotional parenting (indirect effect=-0.53, 95%CI: -0.61 to -0.45), mirrored in stronger amygdala-precuneus connectivity (r=0.31, p=0.002).Professional mothers (ISCED 6-7) showed 37% greater protection (Δβ=0.046), associated with 22% lower anxiety scores (p<0.01) and preserved amygdala volume (+0.32 cm³, p=0.005).Paternal Screen Regulation Pathway: Education reduced externalizing behaviors only through screen co-regulation (indirect effect=-0.39,p<0.001), moderated by DLPFC thinning (-0.2mm per 10% non-educational screen increase, p=0.008).Low-education fathers (ISCED ≤3) had 2.7× higher risk of child ADHD symptoms (OR=2.69, 95%CI: 1.82-3.97) due to unregulated screens. Critical Period Neuroplasticity: In 1-3-year-olds, parenting environment explained 62% of mental health variance (vs. 28% screen effects), with maternal education predicting 34% stronger amygdala-prefrontal connectivity (r=0.37, p=0.005).Rural children faced 1.8× higher anxiety risk (OR=1.79, 95%CI: 1.24-2.57) due to limited educational screen access.

**Conclusions:** This study provides neurobiological evidence for gender-tailored interventions:(1)Maternal: Daily 15-minute “Heart-to-Heart Dialogues” reduce anxiety by 22% (p<0.01) and enhance amygdala-precuneus connectivity.(2)Paternal: “Digital Contracts” limiting non-educational screens to <40% preserve DLPFC thickness (p=0.023) and reduce ADHD symptoms.(3)Policy: Integrate neurofeedback into China’s “Healthy Child 2030” initiative with rural educational screen subsidies, targeting 1-3-year-olds for maximal neuroplasticity benefits.

**Acknowledgements:** This work was supported by a project grant from Research on the Sustainable Development Strategies of Preschool Education Majors under the Background of Negative Population Growth“ [XJK24BGD049] in the “14th Five-Year Plan” for Educational Sciences in Hunan Province & Research on the Development of Inclusive Kindergartens in Rural Areas under the Background of Population Changes” [XJK24CJC008] in the “14th Five-Year Plan” for Educational Sciences in Hunan Province.

## PHHS25125: TRANSLATION QUALITY AND TRANSLATOR PSYCHOLOGY: A COMPARATIVE ANALYSIS OF POST-EDITING, HUMAN AND ARTIFICIAL INTELLIGENCE TRANSLATION IN CHINESE CLASSICAL NOVELS

Qiantao Zhang ^a^

^*a*^ *College of Foreign Languages, Shanghai Maritime University, Shanghai 201306, China.*

**Objective:** To investigate and compare the performance of human translators, artificial intelligence chatbots (DeepSeek) and post-editors in the translation of Chinese classical novels, and to explore differences in psychological states between post-editors and manual translators while translating.

**Subjects and Methods:** A total of 20 students majoring in English or Translation and Interpreting were invited to participate in the study which required them to translate two excerpts selected from a famous Chinese classical novel *The Plum in the Golden Vase*. All participants were divided into two groups, with ten people in each group, to perform translations from scratch or post-edit translations made by DeepSeek. After the completion of their translation tasks, participants were required to fill out a 5-point Likert scale questionnaire composed of three sections: anxiety, confidence and threat perception, which was designed to detect their psychological states while translating and their attitudes to the development of AI. All translated texts of the two groups, along with those made by DeepSeek, were collected and sent to 4 evaluators for scoring. In the end, the researcher conducted a detailed date analysis.

**Results:** According to scores of the three groups, the group of post-editors demonstrated the best performance, with the group of human translators performing the poorest. DeepSeek, though, performed better than human translators, it still made some flaws that needed post-editors to rectify or polish, indicating that AI tools were still unable to independently handle literary translation. According to points filled out in each question of the questionnaire, post-editors showed less nervous and more confident than manual translators during the translation process, but both the groups showed a strong sense of pessimism to their future due to rapid development in artificial intelligence industry.

**Conclusion:** Post-editing can improve the translation quality in literary translation, at least for student translators. Translations made by artificial intelligence, though better than manual translation, a surprising result, still made flaws that needed human to detect, rectify or polish. In the respect of translator psychology, post-editors showed less nervous and more confident than manual translators while encountering difficult translation tasks like literary translation, indicating that post-editing was helpful in relieving negative emotions for student translators in the study.

## PHHS25126: RESEARCH ON THE SPATIAL DIFFERENTIATION, DYNAMIC EVOLUTION, AND CONVERGENCE MECHANISM OF GREEN POVERTY REDUCTION FROM THE PERSPECTIVE OF MENTAL HEALTH

Wen Li^a^, Shan Liu^b^

^*a*^ *School of Tourism and Geography, Shaoguan University, Shaoguan 512005, China,* ^*b*^ *School of Economics, Shandong University of Technology, Zibo 255022, China.*

**Background:** Poverty reduction is a development challenge that cannot be ignored by countries around the world, especially developing countries. Combining green development with poverty alleviation has become an important way to break through the bottleneck of poverty alleviation. In this process, mental health, as an important component of human well-being, also needs to be fully valued. Poverty not only affects material living conditions, but also has a profound impact on individual mental health, and even forms an intergenerational transmission of “poverty culture”. Considering comprehensive poverty reduction through mental health can not only improve poverty alleviation effectiveness, but also promote sustainable development in impoverished areas.

**Subjects and Methods:** This article studies the psychological health issues of residents in the green poverty reduction strategy and poverty reduction process. Text analysis method is introduced to construct a comprehensive index of green poverty reduction. Based on data from 68 cities in Guangdong Province and surrounding areas from 2011 to 2022, Dagum Gini coefficient, kernel density, spatial Markov chain, and σ value are used to reveal spatiotemporal differences, dynamic evolution, and convergence characteristics. Based on the research results, poverty reduction suggestions are proposed from the perspectives of mental health and regional coordinated development.

**Results:** From 2011 to 2022, the overall level of green poverty reduction development has significantly improved, with obvious spatial imbalances; The overall and regional differences in green poverty reduction are becoming increasingly significant, with no convergence characteristics; The higher the level of green poverty reduction development, the stronger the driving effect on surrounding cities. The mental health of residents has a significant impact on poverty reduction and is a key issue that must be addressed during the poverty reduction process.

**Conclusions:** The government has played an important role in accelerating the level of green poverty reduction development, and should formulate green poverty reduction policies tailored to local conditions to promote coordinated development of green poverty reduction regions. From the perspective of mental health, government support for the integration of green skills training can develop ecological restoration projects, provide ecological employment subsidies and psychological counseling subsidies, alleviate the dual burden of economy and psychology, create green employment, and enhance confidence in sustainable development.

**Acknowledgments**

This work is supported by Guangdong Philosophy and Social Sciences Planning Discipline Co construction Project (GD23XYJ79).

## PHHS25127: RESEARCH ON TRANSMEDIA STORYTELLING STRATEGY OF ANIMATION ADAPTATION BASED ON CHILDREN’S MENTAL HEALTH: CENTERED AROUND THE FAIRY TALE BOOK ‘WINNIE THE POOH’

Yan Zhao^a^, Ke Li^b^

^*a*^ *Sejong University, Seoul, South Korea,* ^*b*^ *School of Literature and Journalism, Shandong University, Shandong, China.*

**Objective:** This study aims to explore the transmedia storytelling strategy of animation adaptation based on children’s mental health, taking the animation adaptation of the classic fairy tale Winnie the Pooh as an example. By analyzing the recontextualization process in transmedia storytelling, and combining with children’s psychological development theory, this paper studies its impact on children’s mental health. The core goal of the study is to reveal the application value of transmedia storytelling strategies in animation adaptation, provide theoretical support and practical guidance for animation creation, and promote the healthy development of children’s psychology. Specifically, this study will explore how to effectively transform and present story elements through three stages of entextualization, contextualization and decontextualization, and analyze their effects on children’s emotional resonance, behavioral guidance, and psychological support. Through this study, we hope to provide a new perspective for the theoretical development of transmedia storytelling and animation creation practice, and promote the further development of animation adaptation in meeting children’s psychological needs.

**Subjects and Methods:** This paper takes the animation adaptation of Winnie the Pooh as an example, combining with children’s psychological development theory, to explore the transmedia storytelling strategy of animation adaptation based on children’s mental health. The research methods include literature analysis and case studies, with a focus on analyzing the three stages of entextualization, contextualization, and decontextualization in animation adaptation, and studying their impact on children’s mental health.

**Results:** The research shows that transmedia storytelling strategies can not only improve the communication effect of stories, but also have a positive impact on children’s mental health. By preserving the positive emotions and values in the original text through entextualization, avoiding the introduction of content that may have a negative impact on children through contextualization, and maintaining the positive emotions and values in the story through decontextualization, it can effectively promote children’s mental health development.

**Conclusions:** Transmedia storytelling strategy has important application value and innovative significance in animation adaptation. By combining the theory of children’s mental health, animation adaptation can better meet the psychological needs of children and provide healthy and beneficial entertainment content. The reasonable use of the three stages of entextualization, contextualization, and decontextualization not only enhances the dissemination effect of the story, but also helps children establish a positive psychological support system through emotional resonance and behavioral guidance. In the future, animation creation should pay more attention to children’s psychological characteristics, combine transmedia storytelling strategies, and promote the development of healthy content. At the same time, this study provides a new perspective for the theoretical development and practical innovation of transmedia storytelling, and promotes the further development of animation adaptation in meeting children’s psychological needs.

## PHHS25128: DIGITAL TRANSFORMATION-DRIVEN STRATEGIES AND WORKER’S MENTAL HEALTH FOR SINO-KAZAKH BILATERAL TRADE GROWTH UNDER BRI: A DATA & AI-ASSISTED MANAGEMENT APPROACH

Xin Li^a^, Gulnaz Alibekova^a^, Liangliang Xue^a^, Fei Li^b^

^*a*^ *High School of Economics and Business, Al-Farabi Kazakh National University, Almaty 050040, Kazakhstan,* ^*b*^ *Department of Health Management, Changzhi Medical College, Changzhi 046000, China.*

**Background:** In 2013, China launched the Belt and Road Initiative (BRI), aimed at enhancing global trade and fostering economic integration, particularly between China and Kazakhstan. The initiative focuses on improving infrastructure and resource allocation. Concurrently, Artificial Intelligence (AI) has become a key tool for analyzing complex data patterns and predicting business trends, offering significant potential for economic growth. However, despite AI’s transformative impact on business, research on its application in BRI-related trade remains limited. This study addresses this gap by exploring the role of AI in facilitating Sino-Kazakh trade under the BRI framework. While AI-driven innovations enhance efficiency and growth, they also introduce new challenges, particularly concerning workers’ mental health, which warrants further attention in the context of digital transformation.

**Subjects and Methods:** This research explores the dual impact of digital transformation on trade growth and workers’ mental health in the context of Sino-Kazakh economic cooperation under the BRI. It works opens a better way for bilateral growth management with the help of AI algorithms. We intend to utilize AI for forecasting business trends and demands through long-term time-series data. The first issue encountered is lack of data due to availability of sufficient dataset. To resolve this issue, we proposed Generative Adversarial Network (GAN) to generate synthetic data by suing available data. This phase improves the overall prediction accuracy. After data volume compensation, we prepared the dataset and applied feature engineering phase in which only optimal features are selected. To assist this selection, we presented Walruses Optimization Algorithm (WaO). In forecasting, Long-Short Term Memory (LSTM) model has proved its efficacy but still it has some issues in prediction. To improve LSTM model we proposed novel Temporal Fusion Transformer (TfT) attention mechanism.

**Results:** Overall, the evaluation shows that the proposed TfT-LSTM approach achieves lower error rates with increased accuracy (98%) rates in prediction. Moreover, while the application of digital transformation at the current stage releases technological dividends, it is also essential to pay attention to the mental health of workers. The application of digital transformation has a dual impact on the mental health of workers.

**Conclusion:** The proposed approach shows better results based on experimentation. Moreover, the study emphasizes the importance of integrating mental health considerations into digital transformation strategies.

**Acknowledgments**

The article was prepared as part of scientific research under the program funded by the Committee of Science MSHE RK “Improving the mechanisms for effective regulation of the processes of commercialization of applied R&D projects” (BR21882077).

## PHHS25129: A STUDY ON THE OPENING OF UNIVERSITY SPORTS FACILITIES TO THE PUBLIC AND THE ENHANCEMENT OF RESIDENTS’ SUBJECTIVE WELL-BEING FROM THE PERSPECTIVE OF POSITIVE PSYCHOLOGY

Ke Jiang^a^, Jiaguo Zhu^a^

^*a*^ *Yunnan University of Finance and Economics, Kunming, Yunnan 650221, China.*

**Background:** With the rapid development of social economy and the continuous improvement of people’s living standards, health and happiness constitute two core elements of people’s needs in the new era. Currently, the research on subjective well-being has been extended from the individual psychological level to the intersection of social support system and public resource supply, showing the research trend of multidisciplinary integration. As a public resource with dual attributes, the research on the open sharing mechanism of university sports facilities has important theoretical innovation value and social practice significance.

**Subjects and Methods:** This study uses a combination of questionnaire survey method, interviews, case study method and other methods to conduct a research on the residents of the communities around five universities in Yunnan, to collect the opinions and suggestions of the residents of the surrounding communities on the social openness of university sports facilities, to investigate the psychological changes in the subjective well-being of the residents before and after the openness of the university sports facilities and to analyze the role of the degree of openness of the university sports facilities and the attitude of the residents towards sports activities in the openness.

**Results:** In a study of the impact of university sports facilities on social openness and residents’ well-being, a strong link was found between physical activity and mental health. The study shows that residents who regularly use university sports facilities have higher levels of mental health and life satisfaction than those who do not, which is consistent with the importance of positive emotional experiences for individual mental health, happiness and self-realization as emphasized in positive psychology theory. In addition, the degree to which university sports facilities are open to the community has an impact on residents’ subjective well-being. Specifically, (1) after the opening of university sports facilities to the community, residents’ sports activities increased, social networks expanded, psychological stress decreased, and subjective well-being and life satisfaction increased; (2) positive psychology indicators such as positive emotions and life satisfaction play a positive role between the opening of university sports facilities to the community and residents’ subjective well-being.

**Conclusions:** Research has shown that the opening of university sports facilities to society not only optimizes the allocation of public resources, but also significantly improves residents’ physical and mental health, enhances subjective well-being, and promotes the social development of the university sports industry. Based on the perspective of positive psychology, universities have effectively enhanced the sense of well-being by meeting the residents’ demand for sports activities, promoting social interaction and enhancing self-efficacy through the open-door policy. In the future, we should further improve the opening policy, optimize the management and operation mechanism, strengthen scientific publicity to increase the participation of residents, and deepen the interaction and integration between colleges and universities and the community, so that the sports facilities of colleges and universities can better serve the society, and continue to promote the enhancement of the residents’ health and sense of well-being.

**Acknowledgments**

This work was supported by General Project of Pedagogy of National Social Science Foundation of China, titled “Research on Paid and Unpaid Development of Sports Facilities in Colleges and Universities under the Background of Big Health”, Project Approval No. BLA200215.

## PHHS25130: EXPLORING FACTORS INFLUENCING OCCUPATIONAL BURNOUT AMONG HEALTHCARE WORKERS: AN EXPLORATORY FACTOR ANALYSIS

Zhuoye Zhang^a^, Sixue He^b^

^*a*^ *Department of Education, Guangdong Baiyun University, Guangzhou, Guangdong 510450, China,* ^*b*^ *Department of Management, Guangzhou College of Techology and Business, Guangzhou, Guangdong 510850, China.*

**Background:** In recent years, China has recognized the significance of enhancing the occupational well-being of healthcare workers as a crucial aspect of its public health strategy. While policies now mandate the provision of psychological counseling services and the teaching of emotional management techniques in medical institutions, the persistent shortage of healthcare resources remains a major challenge. Consequently, healthcare workers often find themselves operating in a state of overwhelming work demands, leading to growing concerns about occupational burnout. This study aims to identify the factors that impact healthcare workers and contribute to their occupational burnout, with the ultimate goal of fostering a conducive and supportive work environment for them.

**Subjects and Methods**: A total of 157 healthcare workers participated in a comprehensive questionnaire survey. The Maslach Burnout Inventory-General Survey (MBI-GS) was employed as the assessment tool, measuring occupational burnout across three dimensions: emotional exhaustion, cynicism, and reduced personal accomplishment. The study employed an exploratory factor analysis to investigate the current levels of occupational burnout among healthcare workers. Additionally, it examined the influence of various factors, including individual career factors, work environment conditions, organizational aspects, and personal development considerations, on occupational burnout at Hospital A. The data were analyzed using SPSS 27.0 to extract the primary influencing factors.

**Results:** Descriptive statistical analysis revealed a high overall level of occupational burnout among healthcare workers, particularly in the dimensions of emotional exhaustion and cynicism. The exploratory factor analysis identified individual career factors, work environment conditions, organizational aspects, and personal development considerations as significant factors influencing occupational burnout among healthcare workers, with individual career factors exhibiting the highest impact at 63.68%.

**Conclusions:** Based on the findings, this study proposes targeted strategies and recommendations to mitigate occupational burnout among healthcare workers at the organizational, work, individual, and societal levels. These insights aim to provide effective methods for healthcare workers and management personnel to address and alleviate the challenges associated with occupational burnout.

**Acknowledgments**

This work was supported by a project grant from:

1. The Research Project by the China Private Education Association, 2023 (School Development Category) (CANFZG23088).

2. Foundation for Young Innovative Talents Program in Higher Education of Guangdong Province, China (Grant no. 2020WQNCX074).

## PHHS25131: RESEARCH ON THE PSYCHOLOGICAL EFFECT OF MOVIE DOPAMINE COLOR STYLE ON VIEWERS’ HAPPINESS: “IF YOU ARE THE ONE III” AS AN EXAMPLE

Danning Wang ^a^

^*a*^ *Sichuan University of Culture and Arts, Mianyang, Sichuan 621000, China.*

**Background:** In recent years, the relationship between film visual aesthetics and audience mental health has gradually attracted academic attention. Film color style, as an important visual element, can significantly affect the audience’s emotional experience and psychological feelings. Among them, the dopamine color style characterized by high saturation, high brightness, and strong color contrast is highly praised for its ability to evoke pleasant and positive emotions in the audience. However, there is currently a relative lack of research on the specific psychological effects and mechanisms between dopamine color style in movies and audience psychological well-being, especially a lack of in-depth analysis of typical case films.

**Subjects and Methods:** This article takes the movie “If You Are the One III “ as a specific case study, and uses literature analysis and case analysis methods to systematically explore the connotation, expressive characteristics, and psychological effects of dopamine color style in movies on audience happiness. Specifically, starting from the basic theories of color psychology, this study analyzes the use of color in character costumes, scene design, and other aspects of the film, explores how color style can stimulate positive emotions in the audience, and further analyzes the moderating effect of individual personality traits and emotional sensitivity on color aesthetic experience.

**Results:** Research has found that the dopamine color style in “If You Are the One III” effectively activates the dopamine nervous system of the audience’s brain through visual stimulation, triggering positive and pleasant emotional responses and promoting the improvement of the audience’s subjective well-being. The specific mechanism is manifested as follows: the audience first experiences visual pleasure through the color of the movie, and then gradually forms psychological resonance and happiness experience at the emotional level. In addition, individual personality differences and emotional sensitivity of the audience significantly moderate the experiential effect of this sense of happiness. Audiences with extroverted personalities and high emotional sensitivity experience stronger positive emotions, while introverted or emotionally less sensitive audiences experience relatively milder emotions. The cultural context of film color aesthetics also significantly affects the audience’s acceptance, reflecting the close interaction between visual aesthetics and social psychological needs.

**Conclusions:** Film dopamine color style can significantly enhance the audience’s psychological well-being, and the study provides a new perspective on film color psychology, as well as an effective basis for the theoretical and practical exploration of film visual aesthetics.

## PHHS25132: RESEARCH ON THE MEASUREMENT MODEL AND POLICY IMPLEMENTATION OF HIGH QUALITY DEVELOPMENT OF CHINESE MEDICAL AND HEALTH INDUSTRY BASED ON ARTIFICIAL INTELIGENCE

Liangwen Yue ^a^

^*a*^ *Nanchang Vocational University, Nanchang, 330000, China.*

**Background:** With the rapid development of artificial intelligence technology and the increasing demand for healthcare, the healthcare system and the big health industry are undergoing profound digital transformation. In the context of the post pandemic era, countries are paying more attention to the construction of public health systems and innovation in medical services, and the application value of artificial intelligence in the field of healthcare has been further highlighted.

**Subjects and Methods:** This study takes the perspective of empowering the high-quality development of the healthcare system and the big health industry with artificial intelligence, constructs a measurement model that integrates industry development and policy implementation, and conducts empirical analysis on the current development status of China’s healthcare system and big health industry. Based on systems theory, collaborative innovation theory, and policy implementation evaluation theory, a measurement framework of “three-level four dimensions” was proposed, and a comprehensive evaluation index system was constructed, which includes five dimensions: economies of scale, innovation driven, service quality, intelligent empowerment, and policy implementation effectiveness. By introducing artificial intelligence application level adjustment coefficient, technological innovation capability adjustment coefficient, and policy implementation effect adjustment coefficient, a dynamic evaluation model was established to scientifically measure the high-quality development level of the medical and health system and the big health industry. The composite weighting method combining Analytic Hierarchy Process and Entropy Method is used to determine the weights of indicators, which improves the objectivity and reliability of the evaluation results.

**Results:** This article proposes policy recommendations such as building a collaborative innovation system, optimizing medical and health policy support mechanisms, promoting intelligent transformation, and narrowing regional development differences, providing theoretical basis and practical reference for promoting high-quality development of the medical and health system and the big health industry.

**Conclusions:** We have come to conclusions, Innovatively incorporating the empowerment effect of artificial intelligence and the effectiveness of policy implementation into the evaluation system of industrial development enriches relevant theoretical research and has important theoretical value and practical significance for promoting healthcare system reform and industrial transformation and upgrading.

**Acknowledgments**

This study is supported by the Talent Special Fund Support Project of Nanchang Vocational University; and Nanchang Vocational University School level Teaching Connotation Construction Improvement Project (Project No.: jc2023005); and University level research project of Nanchang Vocational University (Project No. 202313).

## PHHS25133: APPLICATION OF THE “COMPETITION-BASED TEACHING” MODEL IN YOGA COURSES: AN EMPIRICAL ANALYSIS OF PHYSICAL AND MENTAL OUTCOMES BASED ON TEACHING EXPERIMENTS IN 20 UNIVERSITIES

Jianing Wang^a^, Lei Zhao^a^, Qingmei Wang^a^

^*a*^ *Qingdao Huanghai University, Qingdao 26500, China.*

**Background:** With the shift in college physical education from skill acquisition to holistic development, yoga courses have gained popularity for promoting both physical fitness and mental well-being. Yoga helps students manage academic stress, improve mindfulness, and regulate emotions—key factors in supporting mental health. However, traditional yoga instruction often lacks engagement, interactive elements, and content diversity, limiting its effectiveness. To address these issues, this study explores the application of the “Competition-Based Teaching” model as a pedagogical innovation to enhance learning outcomes and psychological resilience in college yoga education.

**Subjects and Methods:** A quasi-experimental design was conducted with 1,200 students from 20 universities in China, divided into experimental and control groups. Over a 16-week semester, the experimental group incorporated structured competitions—such as posture contests, group choreography, and showcase performances—into the standard yoga curriculum. These activities aimed to improve physical skills while also enhancing mindfulness, reducing stress, and fostering emotional resilience. Data collection included pre- and post-experiment questionnaires measuring physical and psychological indicators (e.g., stress levels, emotional regulation), semi-structured interviews with students and teachers, and classroom observation. Statistical analyses using SPSS 26.0 included descriptive statistics and independent-sample t-tests to assess differences between the groups.

**Results:** The experimental group demonstrated significantly higher levels of learning interest, course satisfaction, movement mastery, team collaboration, and mental well-being compared to the control group (p < 0.01). Notably, 76% of students in the experimental group reported improved emotional balance and stress relief, versus 52% in the control group. Furthermore, 85% indicated that teamwork enhanced their mood and social support. While some students experienced mild performance anxiety, it was generally mitigated by peer support and mindfulness practices. Teachers acknowledged the increased workload but viewed the overall impact on student engagement and mental well-being as highly positive.

**Conclusions:** The “Competition-Based Teaching” model is an effective and adaptable approach for improving college yoga instruction. It enhances student motivation, participation, skill development, and psychological health. By combining structured competition with mindful practice, this model reduces stress, builds self-confidence, and encourages social connection. With proper institutional support and thoughtful design, it offers strong potential for wider use in non-competitive physical education programs, contributing to both physical and mental wellness in higher education settings.

## PHHS25134: COMBINED DETECTION OF SEROLOGICAL INDICATORS SAA, CRP, AND COMPLETE BLOOD COUNT IN PREDICTING CHORIOAMNIONITIS IN PATIENTS WITH INTRAPARTUM FEVER AFTER EPIDURAL ANALGESIA

Minhong Chen^a,b^, Ming Yang^a^, Yan Tan^b^

^a^ *The First Dongguan Affiliated Hospital of Guangdong Medical University, Dongguan 523710, Guangdong, China,* ^*b*^ *The First School of Clinical Medicine of Guangdong Medical University, Zhanjiang 524000, Guangdong, China.*

**Objective:** To investigate the clinical value of combined detection of serological indicators including serum amyloid A (SAA), C-reactive protein (CRP), and complete blood count in predicting chorioamnionitis in patients with intrapartum fever after epidural analgesia, and to provide theoretical basis for clinical diagnosis and treatment.

**Methods:** A total of 87 parturients with fever (axillary temperature ≥37.3°C) after epidural anesthesia analgesia admitted to our hospital from January 1, 2023, to August 31, 2024, were enrolled. Based on whether they were diagnosed with chorioamnionitis (CAM), they were divided into CAM group (39 cases) and non-CAM group (48 cases). General characteristics, epidural anesthesia delivery analgesia-related indicators, and neonatal outcomes were compared between the two groups. Levels of SAA, CRP, white blood cells (WBC), and neutrophils (NEUT) were measured. Receiver operating characteristic (ROC) curve analysis was used to evaluate the diagnostic efficiency of individual and combined detection of these indicators. The diagnosis of CAM was established through clinical symptoms, pathological examination, or microbiological confirmation. Statistical analysis was performed using SPSS 21.0 software with significance set at P<0.05.

**Results:** (1) No statistically significant differences were found in general characteristics between the two groups (P>0.05). (2) The levels of SAA, CRP, WBC, and NEUT in the CAM group [(43.17±42.62) mg/L, (13.29±11.07) mg/L, (17.42±3.73)×10^9/L, (15.26±3.61)×10^9/L] were significantly higher than those in the non-CAM group [(20.29±20.42) mg/L, (6.72±6.22) mg/L, (14.85±2.96)×10^9/L, (12.85±2.85)×10^9/L] (P<0.05). (3) ROC curve analysis showed that the area under the curve (AUC) for combined detection of SAA, CRP, WBC, and NEUT was 0.748, significantly higher than individual detection (P<0.05), with a sensitivity of 71.8% and specificity of 79.2%. (4) The neonatal infection rate in the CAM group (17.95%) was significantly higher than that in the non-CAM group (0%) (P<0.05). (5) The pathological examination confirmed a positive rate of 70.27% in the CAM group, while all tested cases in the non-CAM group showed negative results.

**Conclusion:** The combined detection of serological indicators SAA, CRP, and complete blood count has important clinical value in predicting chorioamnionitis in patients with intrapartum fever after epidural analgesia. The combined detection demonstrates higher diagnostic efficiency than single indicators and can provide important reference for early clinical diagnosis and intervention, potentially improving maternal and neonatal outcomes.

**Acknowledgement**

This work was supported by the Guangdong Basic and Applied Basic Research Foundation (2022A1515140133).

## PHHS25135: EXPLORING THE APPLICATION OF MR TECHNOLOGY IN THE RENOVATION OF OLD RESIDENTIAL COMMUNITIES FROM THE PERSPECTIVE OF MENTAL HEALTH

Jixue Zou^a^, Bingshuang Zhang^a^

^*a*^ *College of Human Settlements, Eurasia University, Xi’an 710065, China.*

**Background:** Accelerated urbanization has intensified the urgent need for renovating aging residential communities, particularly in rapidly developing regions. Traditional renovation strategies often prioritize physical infrastructure upgrades—such as plumbing, electrical systems, and façade improvements—while neglecting residents’ psychological well-being and social dynamics. Chronic stressors, including environmental anxiety due to inadequate disaster preparedness, fragmented social bonds, and perceived insecurity in deteriorating public spaces, exacerbate mental health risks, especially among vulnerable groups like the elderly.

**Subjects and Methods:** The study engaged 120 residents from six aging urban communities in China through participatory co-design workshops, where stakeholders collaboratively identified pain points and prioritized interventions. MR simulations were deployed to visualize two key scenarios: (a) disaster response protocols (e.g., flood evacuation routes, earthquake-safe zones) and (b) revitalized social spaces (e.g., interactive parks, multi-generational activity hubs). Four demographically matched communities undergoing conventional renovations served as controls. Resilience-informed design principles included stress-reducing green corridors, modular emergency shelters with real-time MR navigation, and adaptable communal areas promoting intergenerational interaction.

**Results:** Post-intervention analyses revealed significant psychosocial improvements in MR-integrated communities compared to controls. Anxiety scores (GAD-7) decreased by 32% (p<0.05), with 45% higher PSI ratings and 28% improvement in STI scores. MR-enhanced emergency drills reduced situational anxiety by 22%, attributed to immersive scenario training that improved residents’ crisis confidence. Communal spaces designed via MR simulations strengthened neighborhood bonds (r=0.67 between spatial accessibility and social interaction frequency) and reduced elderly loneliness by 40%, as measured by self-reported engagement in group activities. In contrast, control communities reported minimal psychosocial benefits: 68% of participants expressed persistent fears about disaster preparedness, and 55% cited ongoing social isolation, particularly among seniors. Qualitative feedback highlighted MR’s role in fostering emotional ownership of redesigned spaces, with 82% of participants endorsing MR visualizations as critical for understanding renovation impacts.

**Conclusions:** This study demonstrates that integrating resilience theory with MR technology can holistically address the psychological and social dimensions of urban renewal. By embedding mental health metrics—such as anxiety reduction and social trust—into design processes, planners can create environments that not only withstand physical stressors but also nurture emotional security and collective resilience. MR’s immersive capabilities empower residents to actively shape their communities, bridging the gap between technical planning and lived experience. The findings advocate for policy reforms that prioritize psychosocial outcomes in renovation guidelines, ensuring aging communities evolve into inclusive, adaptive, and mentally sustainable habitats. Future research should explore longitudinal effects of MR-driven interventions and scalability across diverse cultural contexts.

## PHHS25136: A REAL-TIME BIOFEEDBACK VISUALIZATION SYSTEM FOR HEALTH MONITORING: AN EMPIRICAL STUDY BASED ON THE ABC MODEL OF ATTITUDES

Zhonghui Chen^a^, Ke Li^b^

^*a*^ *Advanced Film School, Chung-Ang University, Seoul, 156756, Korea,* ^*b*^ *School of Literature and Journalism, Shandong University, Shangdong, China.*

**Objective:** This study aims to explore the integration of biofeedback technology and interactive visual arts, designing an innovative method to transform real-time health monitoring data such as heart rate and blood pressure into dynamic visual expressions, and systematically analyzes how design features influence individual behavioral intentions through emotional and cognitive responses based on the ABC attitude model, thereby revealing public acceptance mechanisms of biofeedback health visualization systems. With increasing health awareness, individuals’ demand for real-time visualization of physiological states is growing, yet traditional data display methods often lack attractiveness and intuitiveness. Artistic presentation of biofeedback data not only makes abstract data more comprehensible but also enhances users’ attention to their health conditions through emotional resonance, providing new ideas for the field of health monitoring.

**Subjects and Methods:** The research built a real-time health monitoring system based on MKB0805 sensors and ESP32 microcontrollers, collecting heart rate and blood pressure data, and mapping them into visual elements through TouchDesigner software to achieve physiology-driven artistic feedback. Through an online platform, 550 questionnaires were randomly distributed, with 501 valid questionnaires obtained after eliminating invalid samples (91.09% validity rate). SPSS was used to conduct reliability analysis (Cronbach’s α), exploratory factor analysis (EFA), and confirmatory factor analysis (CFA), while structural equation modeling (SEM) was further employed to model and verify path relationships and mediating effects.

**Results:** Research results show that visual presentation, technology integration, and interactivity significantly enhanced users’ emotional and cognitive responses, which in turn positively influenced behavioral intentions. Structural equation model analysis indicates that all path coefficients were within significant levels, with emotions and cognition having mediating effects between design features and behavioral intentions. The model demonstrated good fit (χ²/df=1.317, GFI=0.946, RMSEA=0.025, CFI=0.989, NFI=0.956), with excellent scale reliability and validity (Cronbach’s α>0.8, KMO=0.961, cumulative variance explanation rate 72.29%). Most subjects reported that the system improved their cognitive awareness of their physiological states and emotional fluctuations, indicating that biofeedback systems with artistic expression of physiological data can effectively enhance health engagement and self-awareness by influencing users’ emotions and cognition.

**Conclusions:** The study demonstrates that biofeedback systems that artistically express health monitoring data can significantly improve audience engagement and self-health awareness by influencing their cognition and emotions. This innovative approach provides new pathways for health data visualization and creates more attractive interactive experiences for individual health monitoring and health education.

## PHHS25137: RESEARCH ON EFFECTIVENESS EVALUATION METHOD OF CHEMICAL PROTECTION EQUIPMENT BASED ON HUMAN FACTORS IN CIVIL CHEMICAL ACCIDENTS

Sijie Zhu^a^, Dayong Jiang^a^, Yun Bai^a^

^*a*^ *Engineering University of PAP, Xi’an 710086, Shaanxi, China.*

**Background:** Chemical defense equipment plays a crucial role in supporting both military operations and civilian chemical incident rescue missions. Traditional assessment methods for equipment performance, due to their lack of consideration for behavioral sciences, have several shortcomings, including poor integration with non-warfare military operations, limited methodologies, and subjective results. Moreover, these methods often fail to account for the human-equipment collaboration efficiency and the variations in group behavior risk perception, making it difficult to fully reflect the actual operational effectiveness of military equipment in complex civilian rescue scenarios.

**Subjects and Methods:** This study is based on the characteristics of equipment and the processes involved in civilian rescue tasks. It integrates behavioral science theories into the traditional capability indicator system by adding factors such as operator adaptability and human-equipment collaboration efficiency. The FAHP-CRITIC method is used to assign weights to the capability indicators, followed by the application of the improved TOPSIS method with gray relational analysis to assess the performance of equipment in the context of chemical incident rescue operations. Finally, an example verification is conducted using the revised indicator system and evaluation method, comparing the performance rankings of different units’ equipment.

**Results:** The results confirm the validity of the human factor in this evaluation model, with the contribution of behavioral science indicators accounting for 24%. The overall indicator system is rational, the weighting method is scientifically sound, and there is a strong correlation between evaluation units.

**Conclusions:** This research addresses the limitations of traditional evaluations, which often focus on static parameters while neglecting dynamic human factors. The revised indicator system and evaluation methods not only quantify the technical performance of the equipment but also analyze the synergistic effects between operator behavior and equipment characteristics. It further validates the influence of human factors and group behavior risk perception differences, providing theoretical support for optimizing the civil adaptability of military chemical defense equipment.

## PHHS25138: EMBODIED PERFORMANCE IN IMMERSIVE DANCE THEATRE: A PSYCHOSOCIAL HEALTH STUDY WITHIN A PHENOMENOLOGICAL FRAMEWORK

Yutong Xie ^a^

^*a*^ *Chongqing College of Humanities, Science & Technology, Chongqing 401520, China.*

**Background:** With the acceleration of social rhythm and the increasing prominence of mental health problems, finding effective ways of psychological intervention has become the focus of current social attention. As an emerging form of artistic expression, immersive dance theatre emphasizes active audience participation and multi-sensory interactive experience, and shows unique potential in promoting psychological need satisfaction, enhancing well-being, alleviating anxiety, and improving personality traits and other mental health domains. The theory of embodied exhibition under the phenomenological perspective provides an important philosophical foundation for exploring the psychosocial health role of immersive dance theatre.

**Subjects and Methods:** This paper takes the artistic expression of immersive dance theatre and its impact on the psychosocial health of the audience as the object of study, and based on the philosophical framework of phenomenology, through theoretical interpretation and case study analysis, it explores how the embodied performances of immersive dance theatre affect the individual’s psychological experience, emotional regulation and social interaction. Using a combination of literature analysis and case studies, the study systematically analyses the artistic characteristics of immersive dance theatre, the mechanism of physical movement and the mode of audience interaction.

**Results:** The study found that embodied performance in immersive dance theatre had a positive impact on participants’ psychological well-being. On the one hand, individuals achieved effective emotional regulation and anxiety relief through physical participation, enhanced psychological well-being and self-efficacy, and satisfied basic psychological needs; on the other hand, the immersive experience mode strengthened social-emotional exchanges between individuals, and promoted the enhancement of social interaction ability and the positive development of personality. In addition, collective participation in immersive dance theatre significantly enhances participants’ sense of social belonging and cultural identity.

**Conclusions:** Immersive dance theatre achieves a profound impact on individual psychological experience through embodied performance, and effectively improves the mental health of participants. This study not only expands the academic vision of the integration of phenomenological theory and dance art, but also provides a new theoretical basis and practical model for art intervention in mental health. In future research and practice, the specific effects and applicability of immersive dance theatre in mental health interventions for different populations should be further explored.

## PHHS25139: GENOME-WIDE IDENTIFICATION, CHARACTERIZATION, AND EXPRESSION ANALYSIS OF THE *bHLH* GENE FAMILY IN *PINELLIA TERNATE*

Ying Meng^a^, Kaiyue Wang^a^, Youjuan Zhu^a^, Dongrui Liang^a^, Lin Cai^a^, Mengzhu Cao^a^, Yan Yan^a^, Xinbo Zhang^a^, Xiaofeng Shen^b^

^*a*^ *Xuzhou Vocational College of Bioengineering, Xuzhou 221006, China,* ^*b*^ *Chinese Academy of Medical Sciences Peking Union Medical College Institute of Medicinal Plant Development, Beijing 100000, China.*

**Background:** The *bHLH* gene family is acknowledged as the second largest transcription factor family in plants, and serves a crucial function in governing plant growth, development, morphogenesis, and responses to both biotic and abiotic stresses. However, a comprehensive investigate the function of the *bHLH* gene family in *Pinellia ternate* is yet to be down. The present study aims to detect and evaluate *bHLH* gene family members using whole-genome sequencing data from *P. ternate* to their potential functions.

**Subjects and Methods:** A genome-wide analysis of the *bHLH* gene family using various databases and web tools to investigate phylogenetic relationships, chromosomal localization, gene structure, conserved motifs, cis-regulatory elements, transcriptional expression patterns, and potential protein-protein interactions.

**Results:** The findings revealed that the *bHLH* gene family in *P. ternate* consists of 131 members, named *PtbHLH*. The *PtbHLH* genes were unevenly distributed across 20 chromosomes of peanut, with copy numbers ranging from 1 to 19. The *PtbHLH* proteins ranged in length from 75 aa to 895 aa, with molecular weights between 8,394.6 Da and 96,730.5 Da, and isoelectric points 4.73-11.37. Among these, 3 were classified as stable proteins while 128 exhibited hydrophilic properties, with subcellular localization observed in the nucleus, mitochondria, chloroplasts, and cytoplasm. Phylogenetic analysis revealed that the *PtbHLH* gene family could be classified into 17 subfamilies, with genes within identical groups displaying comparable structural characteristics. The conserved motifs, Motif1 and Motif2, were identified in most *PtbHLH* genes. Collinearity analysis uncovered four duplication events, among which one was identified as a tandem duplication event. In addition to core cis-acting elements, various functional response elements associated with light regulation, hormone responses, anaerobic stress response, and MYB-binding sites were identified in the promoter regions of *PtbHLH* genes. The examination of *PtbHLH* gene expression patterns in *P. ternata* under high-temperature stress demonstrated that six genes exhibited gradual upregulation, whereas those of sixteen genes persistently declined as the duration of heat stress extended. the PPI network consisted of 58 nodes and 147 edges, indicating that these *PtbHLHs* interact mutually and with additional proteins, thus contributing to diverse biological processes..

**Conclusions:** These insights enhance the comprehension of the *PtbHLH* gene family and establish a significant basis for subsequent functional research on *PtbHLH* genes in *P. ternata*.

**Acknowledgments**

This work was supported by the Basic Research Project of the Natural Science Foundation of the Jiangsu Higher Education Institutions (23KJB360017), Basic research program young talents in science and Technology project of Xuzhou (KC23037, KC23064), School level scientific research project of Xuzhou Vocational College of Bioengineering (XSZR202513).

## PHHS25140: RESEARCH ON THE CONSTRUCTION OF A PROJECT LABELING SYSTEM BASED ON KNOWLEDGE MODELING: FROM THE PERSPECTIVE OF PSYCHOLOGICAL NEEDS OF PROJECT PARTICIPANTS

Guangyu Bai^a^, Lizhi Han^b^, Pengyuan Zhu^a^, Shiwen Li^a^

^*a*^ *Nari Group Corporation Limited, Nanjing 210000, Jiangsu, China,* ^*b*^ *State Grid Xinjiang Electric Power Company, Urumqi 830000, Xinjiang, China.*

**Background:** Company projects can be classified into sixteen categories based on professional types, including infrastructure, technological transformation, major repairs, marketing, information management, and more. Each category has distinct characteristics and management standards. By extracting labels from various dimensions, such as professional attributes and task attributes, these projects are accurately subdivided into their respective professional fields, creating specialized business groups. From the perspective of psychological needs of project participants, differentiated management strategies are proposed to enhance project reserve management.

**Subjects and Methods:** By studying the development trends of artificial intelligence technologies, analyzing current applications of these technologies, and reviewing relevant domestic and international literature, this paper establishes the application pathways of artificial intelligence in project feature recognition. Currently, State Grid projects are diverse, with numerous categories, and many projects have numerous documentation entries, resulting in low project management efficiency and a challenging category structure, which leads to emotional anxiety among project participants and negatively impacts project development.

**Results:** Based on the current situation and problems of specialized projects, this research focuses on the theme of labels, organizing the business characteristics of company projects, refining knowledge data, and mining labels for various specialized businesses. This study proposes an innovative project label construction method based on knowledge modeling, focusing on the psychological needs of project participants. The model utilizes a BiLSTM-CRF sequence labeling model for label recognition of short texts, a Word2vec+SVM model, and applies multi-label text classification techniques to extract and model long texts. This is aimed at improving the company’s project system mining model and knowledge extraction.

**Conclusions:** Based on a large amount of structured project data, this method uses supervised algorithms with minimal labeled data for automatic project system mining. It aims to manage existing specialized projects with a multi-dimensional, multi-level label system, while allowing the training model to be dynamically adjusted and configured. It supports dynamic rule expansion and can adapt well to new project data. This greatly improves project management efficiency and reduces emotional anxiety among project participants. The related content of this project complies with the relevant regulations of XXX Company Information System Integration Branch: “Research on Knowledge-Enhanced Intelligent Analysis Technology in State Grid Project Management.”

## PHHS25141: NAMED ENTITY RECOGNITION IN THE MEDICAL FIELD BASED ON BERT-BIGRU-CRF

Jingtao Sha^a^, Junhao Gao^b^, Pengtao Jia^b^, Wenqian Dong^b^, Xin Cao^a^, Mingkai Jin^a^, Zhenxuan Gao^a^, Yonghao Li^a^, Enxia Liu^a^

^*a*^ *Department of Proctology, Xi’an Hospital of Traditional Chinese Medicine, Xi’an, 710021, China,* ^*b*^ *College of Computer Science and Technology, Xi’an University of Science and Technology, Xi’an, 710054, China.*

**Background:** Named Entity Recognition (NER) serves as a crucial cornerstone for numerous subsequent tasks within the realm of natural language processing. As an important international language, Chinese possesses unique characteristics that often lead to issues such as polysemy in NER, particularly in specialized medical domains.

**Subjects and Methods:** To address the challenges posed by rare and complex medical terminology and lengthy text in Chinese medical data, this paper introduces a deep learning-based model for recognizing medical entities, BERT, BiGRU and CRF. This model encodes the text data from electronic medical records using an improved multi-layer bidirectional Transformer encoder (BERT). By leveraging contextual information, it enriches the semantic representation of characters, effectively addressing polysemy issues. The resulting character vectors are then utilized as inputs to a Bidirectional Gated Recurrent Unit (BiGRU) architecture, enabling the extraction of contextual features embedded within the medical textual data.

**Results:** Conditional Random Fields (CRF) are used for sequence decoding and labeling to identify named entities. The experimental evaluations on the CCKS 2019 Chinese electronic medical record NER dataset reveal that the BERT-BiGRU-CRF model surpasses other recognition models, including BERT-RNN, BERT-LSTM, BERT-GRU, BERT-BiRNN, and BERT-BiLSTM, in terms of both accuracy and robustness.

**Conclusions:** this model significantly enhances the precision of Chinese medical entity recognition, thereby facilitating more efficient utilization of medical text data and accelerating the progress of smart healthcare solutions.

**Acknowledgments**

This research was supported by the Scientific and Technology Program Funded by Xi’an City, grant number 22YXYJ0009.

## PHHS25142: A STUDY ON THE CONSTRUCTION OF THE UNIVERSITY EXTRACURRICULAR SPORTS CLUB MANAGEMENT MODEL UNDER THE BACKGROUND OF HEALTHY CHINA

Fangfang Nie^a^, Qunhai Zou^a^

^*a*^ *Nanchang Normal University, Nanchang 330032, Jiangxi, China.*

**Background:** With the ongoing implementation of the “Healthy China” strategy, the emphasis on physical education in universities has grown significantly. The *Opinions on Comprehensively Strengthening and Improving School Physical Education in the New Era* advocates for every student to master at least one or two sports skills, aiming to promote a balance between cultural education and physical exercise. In this context, university extracurricular sports clubs play a critical role as platforms for developing students’ athletic skills and improving their physical fitness. Therefore, effective management and development of these clubs are vital. However, many clubs currently face management challenges that hinder their ability to meet the growing demand for student physical activity.

**Methods:** This research employs three primary methodologies: literature review, field investigation, and experimental analysis. The literature review focuses on examining both Chinese and international research to assess the application of WSR theory across various fields, especially in the context of public health service governance. The field investigation includes surveys of extracurricular sports clubs at several universities, collecting primary data on their operational conditions and identifying management issues. Based on these findings, a new management model for university sports clubs, incorporating the physical, logical, and human dimensions, is developed. To test the model, three universities representing different types were selected as case studies: a comprehensive university (University A), a university specializing in science and technology (University B), and a teacher training university (University C). The study explored their sports club management models over the course of a year.

**Results:** This research employs three primary methodologies: literature review, field investigation, and experimental analysis. The literature review focuses on examining both Chinese and international research to assess the application of WSR theory across various fields, especially in the context of public health service governance. The field investigation includes surveys of extracurricular sports clubs at several universities, collecting primary data on their operational conditions and identifying management issues. Based on these findings, a new management model for university sports clubs, incorporating the physical, logical, and human dimensions, is developed. To test the model, three universities representing different types were selected as case studies: a comprehensive university (University A), a university specializing in science and technology (University B), and a teacher training university (University C). The study explored their sports club management models over the course of a year.

**Conclusions:** The study confirms the feasibility and effectiveness of the extracurricular sports club management model based on WSR theory. The implementation of this model results in enhanced organizational efficiency and management capabilities, increased student participation, and improved physical health outcomes. Additionally, it contributes to the overall development of students’ sports literacy and holistic abilities. The findings provide valuable insights and practical recommendations for the ongoing reform of physical education in universities.

## PHHS25143: RESEARCH ON THE COUNTERMEASURES FOR THE PREVENTION AND CONTROL OF INTEGRITY RISKS IN PUBLIC HOSPITALS UNDER THE COSO FRAMEWORK: FOCUSING ON MEDICAL, HEALTH, AND MENTAL HEALTH PERSPECTIVES

Xiehui Xu^a^, Bo Su^b,c^, Xinjie Han^b^, Bingying Zhang^b,c^

^*a*^ *Huangshi Maternal and Child Health Hospital (Affiliated Maternal and Child Health Hospital of Hubei Polytechnic University), Huangshi 435000, Hubei, China,* ^*b*^ *Hubei University of Chinese Medicine, Wuhan 430065, Hubei, China,* ^*c*^ *Hubei Provincial Key Research Base of Humanities and Social Sciences in Colleges and Universities: Traditional Chinese Medicine Development and Research Center, Wuhan 430065, Hubei, China.*

**Background:** In the key process of deepening the reform of the medical and health system and promoting the construction of a healthy China, public hospitals, as the core body of medical services, have a great significance for the development of medical science, the quality of health services, and the mental health of the public. Based on COSO internal control theory, this study aims to analyze the current situation and problems of integrity risk prevention and control in public hospitals and construct effective prevention and control strategies to support the healthy development of medical and health care.

**Subjects and Methods:** The study comprehensively utilizes the literature research method to sort out the relevant theoretical achievements at home and abroad and grasp the research frontier; through the case study method, typical public hospital cases are selected for in-depth analysis, and potential problems are excavated; and with the help of the field research method, real information on the front line of hospitals is obtained to ensure that the study is close to the reality.

**Results:** The study found that the current public hospitals in the integrity risk prevention and control of risk awareness of some leading cadres are weak, integrity risk investigation is not comprehensive and inaccurate, supervision and assessment are not in place and the prevention and control measures are not targeted and other issues. At the same time, it is clear that the objectives of integrity risk prevention and control and internal control are the same and integrated. Internal control is a necessary means of integrity risk prevention and control. Based on the COSO internal control framework, the control environment, risk assessment, control activities, information and communication, supervision and inspection, and other elements of integrity risk prevention and control in public hospitals are analyzed, and targeted prevention and control countermeasures are proposed.

**Conclusions:** The study concludes that the integrity risk prevention and control mechanism of public hospitals can be effectively improved by carrying out diversified integrity education, comprehensively sorting out the list of powers, promoting the construction of systems, and perfecting the assessment, evaluation, and accountability mechanism. This not only helps to create a clean and honest medical environment and improve the level of medical services, but also promotes medical research innovation and talent cultivation, guarantees the fair distribution of medical resources, reduces the psychological burden of the public, and is of great significance to the realization of the goal of promoting the construction of a healthy China.

**Acknowledgments**

This research was supported by the 2024 Traditional Chinese Medicine Development and Research Center and Huangshi Maternal and Child Health Hospital Project “Exploration on the Construction of Integrity Culture System in Public Hospitals from the Perspective of Internal Control”(2024ZXHSFY19).

## PHHS25144: EXPLORING THE ROLE OF PERFORMANCE PAY INTENSITY IN SHAPING EMPLOYEE INNOVATION AND MENTAL HEALTH: A CONCEPTUAL FRAMEWORK

Xue Jiang^a^, Nurul Hidayu Binti Mat Jusoh^a^, Ribka Alan^a^, Tunung Robin^a^

^*a*^ *Department of Social Science and Management Faculty of Humanities, Management and Science, Universiti Putra Malaysia (Bintulu Campus), Bintulu, Sarawak, Malaysia.*

**Background:** This paper proposes that moderate performance pay intensity not only enhances exploratory innovation by balancing extrinsic motivation with intrinsic creative drives, but also positively impacts employees’ mental health, encompassing their psychological well-being, job satisfaction, and anxiety levels. The model explicates the effects of performance pay intensity on employees’ exploratory innovation behavior and corresponding mental health outcomes. By recognizing the vital relationship between financial incentives and psychological aspects, this research highlights the importance of designing compensation structures that do more than just drive performance; they must also attend to the mental well-being of the workforce.

In a rapidly evolving business landscape, fostering creativity and innovation is essential for organizational success. However, this endeavor can be hampered by employee stress and anxiety, often exacerbated by high-intensity performance pay systems that focus excessively on outcomes. As such, the interaction between performance pay intensity and mental health becomes increasingly significant. Previous research has established that employees who experience high levels of job satisfaction and low anxiety are more likely to engage in innovative behavior, emphasizing the need for a holistic approach to performance pay.

**Subjects and Methods:** Being a conceptual paper, it reviewed scholarly articles on the variables of the study in accordance with theoretical perspectives. In this paper, cognitive evaluation theory is used to explain and support this conceptual model. The study also incorporates theories related to job satisfaction, anxiety, and psychological well-being to explore the impact of performance pay intensity on employees’ mental health.

**Results:** The findings suggest that moderate performance pay intensity can lead to higher levels of exploratory innovation and better mental health outcomes for employees. When performance pay is perceived as informative rather than controlling, it can enhance employees’ intrinsic motivation, job satisfaction, and psychological well-being, while reducing anxiety. However, extreme levels of performance pay intensity may have the opposite effect, leading to decreased innovation and poorer mental health. **Conclusions:** This study will help companies to develop moderate performance pay intensity to maximize employees’ exploratory innovative behaviors and promote their mental health. By applying cognitive evaluation theory and considering the psychological aspects of employees, this paper contributes to the understanding of how financial incentives interact with intrinsic motivation and mental health in driving innovative behaviors and overall job satisfaction.

## PHHS25145: THE EFFECT OF BU-SUI-DAN COMBINED WITH ALENDRONATE SODIUM TABLETS ON CHRONIC PAIN IN SENILE OSTEOPOROSIS: A RANDOMIZED CONTROLLED TRIAL

Yitao Liao^a^, Feng Qiu^b^, Chao Li^c^, Qingming Xiao^c^, Xian Zhang^c^

^*a*^ *Department of Wuxi Affiliated Hospital, Nanjing University of Chinese Medicine, Nanjing 210023, Jiangsu, China,* ^*b*^ *Department of Orthopedics, Xinwu District Traditional Chinese Medicine Hospital of Wuxi, Wuxi 214135, Jiangsu, China,* ^*c*^ *Department of Orthopedics, Wuxi Affiliated Hospital of Nanjing University of Chinese Medicine, Wuxi 214071, Jiangsu, China.*

*YL and FQ contributed equally to this study.*

**Background:** Senile osteoporosis is often accompanied by chronic pain that significantly reduces patients’ quality of life. This study aims to investigate the effect of the traditional Chinese medicine Bu-Sui-Dan (BSD) combined with Alendronate Sodium Tablets on chronic pain in patients with senile osteoporosis.

**Subjects and Methods:** In a single-center, open-label, randomized controlled trial, 80 patients with senile osteoporosis were randomly assigned in a 1:1 ratio to either a control group or an observation group. The control group received conventional treatment with Calcium Carbonate D3 Tablets and Alendronate Sodium Tablets, while the observation group received BSD in addition to the conventional treatment for 24 weeks. The primary outcome measure was the Visual Analog Scale (VAS) score, while secondary outcome measures included bone metabolic markers, Bone Mineral Density (BMD), Traditional Chinese Medicine Syndrome Score (TCMSS), the Chinese Osteoporosis Targeted Quality of Life Short Questionnaire (COQOL), and the incidence of adverse drug reactions (ADRs).

**Results:** A total of 75 participants completed the trial. The improvement in VAS scores in the observation group was significantly better than that in the control group (P<0.001). At 24 weeks, the observation group had significantly higher serum PⅠNP levels and significantly lower serum CTX levels compared to the control group (P<0.001). The lumbar BMD T-score of the observation group at 24 weeks was significantly higher than that of the control group (P<0.05). The improvement in TCMSS in the observation group was significantly better than that in the control group (P<0.001). The comparison of TCM symptom efficacy results showed that the improvement rate of TCM symptoms in the observation group was significantly better than that in the control group (P<0.001). The observation group showed greater improvements in COQOL scores compared to the control group (P<0.05). There was no significant difference in the incidence of adverse reactions between the two groups.

**Conclusions:** The trial results demonstrated that BSD combined with conventional treatment more effectively alleviates chronic pain in patients with senile osteoporosis, improves treatment efficacy, and enhances patients’ quality of life. No significant ADRs were observed during clinical application.

**Acknowledgements**

This work was supported by a project grant from the Jiangsu Natural Science Foundation (Grant No. BK20231147), as well as by the Jiangsu Traditional Chinese Medicine Science and Technology Development Program (Grant No. MS2021044), the Health Commission of Wuxi City’s project for promoting scientific and technological achievements and appropriate technologies (Grant No. T202430), and the general research project of the Wuxi City Health Commission (Grant No. M202114).

## PHHS25146: THE DEVELOPMENT OF MUSIC EDUCATION IN CHINA FROM THE PERSPECTIVE OF STUDENTS’ PHYSICAL AND MENTAL HEALTH UNDER THE NEW CURRICULUM STANDARDS

Lingshuang Wei ^a^

^*a*^ *Anshan Normal University, Anshan, Liaoning, China.*

**Background:** Chinese music education boasts a long and illustrious history, dating back to the Zhou Dynasty when the Da Si Yue (Grand Minister of Music) was established, marking the earliest documented music education institution in China. This institution, with its relatively complete system, played a crucial role in the development of music education in ancient China. This paper, through a review of Lu Shasha’s work *The Philosophy of Music Education Centered on Music Culture*, explores the significance of music education philosophy within the field of music education. It analyzes the developmental trajectory of music education in China, and examines how, under the framework of the new curriculum standards, the advancement of music education can foster students’ physical and mental well-being and enhance their overall quality.

**Subjects and Methods:** Through Lu Shasha’s article, the value and significance of music education are discussed in areas such as cultural inheritance, aesthetic experience, and personalized development. A historical review of Chinese music education is provided from the Perspective of Students’ Physical and Mental Health, highlighting the features and changes of music education and music therapy in different historical periods, as well as the future direction and strategies for music education under the new curriculum standards.

**Results:** This paper deepens the understanding of music education philosophy, emphasizing the central role of music culture within music education. Concrete strategies for advancing Chinese music education under the new curriculum standards are proposed, such as enhancing musical experiences, promoting large-unit teaching, and integrating music education with music therapy, thereby fostering students’ physical and mental Health development.

**Conclusions:** Music education plays a crucial role in cultural transmission, cultivating aesthetic sensitivity, and enhancing humanistic literacy. Under the new curriculum standards, Chinese music education should continue to deepen its understanding of music education philosophy, emphasize the profound meaning of music, promote effective teaching methods, and integrate music education with music therapy, thereby fostering students’ physical and mental Health development and improving their comprehensive qualities. Simultaneously, it calls on teachers to continually explore and innovate, contributing wisdom and strength to the development of music education.

## PHHS25147: THE ROLE OF EMOTION IN SUPER-WIDE URBAN TUNNEL DRIVING EXPERIENCE: MECHANISMSN AND COMFORT EFFECTS

Biao Li^a,b^, Borong Li^c,d^, Guangxian Sun^a^, Tun Li^c,d^, Jiazhou He^c,d^, Zhenyu Zhao^e^

^*a*^ *Shijiazhuang Tiedao University, Shijiazhuang, Hebei 050048, China,* ^*b*^ *Shijiazhuang Transportation Investment Development Group Co., Ltd., Shijiazhuang 050011, China,* ^*c*^ *China Communications First Highway Survey and Design Institute Co., Ltd., Xi’an 710000, Shaanxi, China,* ^*d*^ *National Key Laboratory of Green and Long-Life Road Engineering in Extreme Environment, Xi’an 710000, Shaanxi, China,* ^*e*^ *College of Transportation Engineering, Chang’an University, Xi’an 710064, Shaanxi, China.*

**Background:** With the development of urban construction and infrastructure in China, the number of underground tunnels in cities is steadily increasing. As the number of lanes on urban expressways and main roads continues to grow, the human perception and driving experience within super-wide cross-sectional underground tunnels, accommodating four or five lanes, have garnered considerable attention. This study explores the factors affecting drivers’ perceived comfort in super-wide urban tunnels, with a special emphasis on the role of emotion and its underlying mechanisms.

**Subjects and Methods:** We designed a simulated driving environment tailored for super-wide cross-section tunnels and organized participants to undergo simulated driving tests. The Profile of Mood States (POMS) questionnaire served as our tool to gather data on drivers’ emotional states and psychological perceptions across different environments. Subsequently, we employed a Multiple Indicator Multiple Causes (MIMIC) model to construct our analytical framework. Furthermore, our study delves into the intricate relationship between various dimensions of psychological perception and driving comfort. It also explores the interplay and correlation between tunnel cross-sections, lighting conditions, and how these factors influence driving comfort, with a particular emphasis on the role of emotions.

**Results:** (1) A super-wide cross-section significantly boosts drivers’ comfort. (2) In super-wide tunnels, brightness and color temperature greatly influence comfort, with neutral white proving to be the most preferred color for drivers. (3) Color temperature primarily affects psychological comfort by reducing confusion and tension, with minimal impact on fatigue. (4) The width of the cross-section influences vigor, ultimately impacting driving comfort.

**Conclusions:** Based on these groundbreaking findings, we can unlock a deeper understanding of drivers’ perceptions and emotions when navigating urban tunnels. They shed light, to a considerable degree, on the intricate mechanisms through which super-wide cross-sectional tunnels exert their influence on driving comfort, offering fresh and insightful perspectives on the role of lighting configuration in shaping the psychological experience of drivers.

**Acknowledgments**

This work was supported by the Shaanxi Innovation Capability Support Plan Project (Transportation Infrastructure Health Support Innovation Team, 2022TD-16) and National Key R&D Program of China under Grant [number 2019YFE0108000].

## PHHS25148: INTERNET GAMING DISORDER AMONG HIGHER VOCATIONAL NURSING STUDENTS: CASES AND MEDIATING FACTORS

Rongfang He^a,b^, Min Huang^b,c^, Xin Xie^b,d^, Juan Chen^a^, Luwei Xiang^a^, Shasha Hu^a,b^

^*a*^ *Department of Psychiatry, The Affiliated Hospital of Southwest Medical University; Fundamental and Clinical Research on Mental Disorders Key Laboratory of Luzhou, Luzhou 646000, Sichuan, China,*^*b*^ *Department of Nursing, The Affiliated Hospital of Southwest Medical University, Luzhou 646000, Sichuan, China,*^*c*^ *Department of Respiratory Medicine, The Affiliated Hospital of Southwest Medical University, Luzhou 646000, Sichuan, China,*^*d*^ *Department of Urology Surgery, The Affiliated Hospital of Southwest Medical University, Luzhou, Sichuan 646000, China.*

*RH and MH contributed equally to this research.*

**Background:** The growing concern about excessive gaming and its impact on individual well-being, particularly among young adults. While Internet gaming disorder (IGD) has been extensively studied in various populations, there is a lack of research specifically focused on higher vocational nursing students. Higher vocational nursing students face unique challenges due to the demanding nature of their academic program and the importance of developing strong professional skills. However, little is known about the prevalence and specific risk factors of IGD in this unique student population.

**Subjects and Methods:** A total of 4021 nursing students in a higher vocational college in Sichuan province in Southwest China were enrolled in the study. A cross-sectional study was performed. Using questionnaires for data collection including General information, Internet Gaming Disorder Scale (IGD-20), Multidimensional Peer Aggression Scale (MPVS), Psychological resilience (CD- RISC), and Strengths and Difficulties Scale (SDQ) to investigate IGD among senior nursing students in vocational colleges and to analyze their Internet use, related influencing factors, and preventive measures.

**Results:** Of all the nursing students, 11.6% had IGD. By gender, fewer female students (10.6%) had IGD than male students (19.4%). A higher proportion of myopic students (12.6%) had IGD than nonmyopic students (10.2%). Mothers’ occupation, significant influence of IGD was observed among students (*t* = 26.023, *P* < 0.05). Urban students had the highest detection rate of IGD, with 111(14.1%). IGD scores had positively related to multidimensional peer aggression (MPVS) scores (*r* = 0.344) and Difficulties (SDQ) scores (*r* = 0.305), conversely had positively related to psychological resilience (CD-RISC) scores (*r* =-0.291). Psychological resilience was negatively correlated with both MPVS and SDQ scores. Multidimensional peer aggression had positively related to IGD (*β*=0.274, *P*<0.001); psychological resilience (*β*=-0.036, *P*<0.05) was negatively associated with the interaction between multidimensional peer aggression and IGD. This indicates that psychological resilience partly mediated the effect of IGD on multidimensional peer aggression, students with low psychological resilience were more likely to become addicted to the Internet.

**Conclusions:** Education should prioritize identifying and addressing students’ potential daily aggressions, fostering their psychological resilience, and nurturing a supportive familial environment. It may effectively mitigate the detrimental effects of IGD, including physical and psychological harm and academic underachievement.

**Acknowledgments**

The work was funded by Research Program of the Sichuan Psychological Society (SCSXLXH2021046); Southwest Medical University Social Science Federation Project (SWUSS202106).

## PHHS25149: EMOTION CLASSIFICATION MODEL BASED ON DEEP BELIEF NETWORK

Haowei Pan ^a^

^a^ *Xi ’an Jiaotong-Liverpool University, Suzhou, Jiangsu, China.*

**Background:** Emotion recognition is a critical task in the field of artificial intelligence and emotional computing, which involves understanding and classifying human emotional states. With the development of deep learning technology, deep belief network (DBN) has become a promising method for emotion recognition because of its excellent learning ability and generalization ability. Electroencephalography (EEG), as a non-invasive neuroimaging technique, can capture brain activity and provide a rich data source for emotion recognition. However, how to extract useful information from EEG data and make accurate mood classification, especially in multi-classification scenarios, remains a challenge. Therefore, it is of great significance to explore and optimize the emotion recognition model based on DBN to improve the recognition accuracy and promote the development of emotion computing.

**Subjects and Methods:** This paper mainly focuses on three aspects: First, the traditional DBN training method is used to train EEG data directly, and the performance of the model is evaluated; Secondly, feature extraction technology is introduced, and the extracted features are input into DBN for training and evaluation, so as to enhance the model’s ability to recognize emotional states. Finally, we explore and evaluate different DBN training strategies, introducing regularization techniques such as Dropout and DropConnect methods to improve the model’s generalization ability.

**Results:** By comparing the results of the three methods, we find that the DBN model combining feature extraction and regularization training strategies can improve its performance on emotion recognition tasks. This finding suggests that by optimizing the structure and training strategy of DBN, the accuracy of emotion recognition based on EEG data can be significantly improved.

**Conclusions:** This study provides valuable insights into the training and feature processing of deep learning models. These findings have important implications for the development of more accurate and reliable emotion recognition systems, and are expected to have a positive impact on mental health monitoring, human-computer interaction and other fields.

## PHHS25150: THE HEALING FUNCTION OF PIANO MUSIC: A MECHANISM OF FILM AND TELEVISION SOUNDTRACKS TO REGULATE VIEWERS’ ANXIETY

Guanzi Wen ^a^

^*a*^ *Sichuan Conservatory of Music, Chengdu, Sichuan 610021, China.*

**Background:** As an expressive art form, piano music is widely used in film and television soundtracks for emotional communication, narrative reinforcement and audience psychological adjustment. In recent years, research in music psychology and music therapy has shown that piano music can not only alleviate anxiety and regulate emotions, but also satisfy the psychological needs of individuals in terms of happiness, emotional security and self-identity. This paper focuses on the healing function of piano music in film and television soundtracks, and explores how it can regulate audience anxiety through emotional resonance, narrative immersion and aesthetic healing, and play a positive role in promoting mental health.

**Subjects and Methods:** This paper adopts the qualitative case study method, selects The Pianist, The Legend of 1900 and other typical films as the research object, combines musicological analysis, narrative theory and psychological perspective to analyse the emotion regulation mechanism and psychological role of piano music in film and television, and supplements it with relevant literature review to construct a theoretical support system.

**Results:** Piano music has multiple psychological effects in film and television: it resonates with the audience’s inner experience on the emotional level, guides emotional transitions and narrative rhythm on the cognitive level, and relieves anxiety and stress by stimulating neurotransmitters on the physiological level. The use of piano music in key situations not only enhances the narrative tension, but also achieves psychological soothing and emotional catharsis for the audience. Different styles of piano music have diverse regulatory effects on personality integration, emotional expression and psychological needs fulfilment.

**Conclusions:** Piano music, as a non-verbal, aestheticised form of psychological intervention, demonstrates significant healing potential in film and television soundtracks. Its emotion-regulating function not only enriches artistic expression, but also extends to the audience’s psychological well-being, reflecting its mental health value in relieving anxiety, promoting emotional clarity and enhancing well-being. This study is of great significance to the fields of film and television creation, music therapy and psychological intervention, and provides a practical path for emotional education, image healing and public mental health.

## PHHS25151: RESEARCH ON THE COUNTERMEASURES TO IMPROVE THE MECHANISM OF FAMILY-SCHOOL-SOCIETY COLLABORATIVE EDUCATION IN UNIVERSITIES IN THE NEW ERA——FROM THE PERSPECTIVE OF COLLEGE STUDENTS’ MENTAL HEALTH EDUCATION

Jing Li^a^, Yunbing Hu^b^

^*a*^ *School of Smart Finance and Economics, Chongqing City Management College, Chongqing 401331, China,* ^*b*^ *Artificial Intelligence and Big Data College, Chongqing Polytechnic University of Electronic Technology, Chongqing 401331, China.*

**Background:** In recent years, China has placed great emphasis on the psychological health and development of college students. Relevant regulations have incorporated the collaborative education mechanism of family, school, and society into the legal system, stressing the importance of establishing and improving this mechanism, further promoting the high-quality development of family-school-society collaborative education.

**Subjects and Methods:** With the rapid development of technology and the diversification of society, improving the family-school-society collaborative education mechanism is the key to solving the fundamental problem of “what kind of people to cultivate, how to cultivate them, and for whom to cultivate them,” and is also an effective guarantee for promoting college students’ mental health education. In 2023, the Ministry of Education and thirteen other departments jointly issued a document, clearly proposing that “by 2035, a clear, well-established, closely linked, and scientifically efficient collaborative education mechanism among schools, families, and society will be formed.” This provide clear guidance for enhancing the mental health of university students.

**Results:** However, looking at the current state of higher education in China, there are still problems such as the imperfect collaborative education mechanism between school education, family education, and social education, inadequate practice of collaborative education, and insufficient family education capabilities. At present, Chinese higher education is facing new challenges and opportunities. We need to re-examine the positioning and methods of education, pay attention to the construction of the family-school-society collaborative education mechanism in universities, strengthen experience summary and learning from others, deepen the exploration of issues and discussions, and continuously optimize its construction method to enhance its positive role in the growth of university students, cultivating mentally healthy, well-rounded, and socially adaptive talents for the new era. Therefore, improving the family-school-society collaborative education mechanism in universities and promoting the mental health development of college students has become an urgent task.

**Conclusions:** This article starts from the current research status at home and abroad, explains the connotation of family-school-society collaborative education, focuses on analyzing the issues facing the mechanism of family-school-society collaborative education, and from the perspective of college students’ mental health education, proposes implementation paths and suggestions for improving the mechanism of family-school-society collaborative education in universities.

**Acknowledgments**

This article is a phase of the research project “Research on the Improvement of the Family-School-Society Collaborative Education Mechanism in Higher Vocational Colleges in the New Era” funded by Chongqing City Management Vocational College (20CSZX06).

## PHHS25152: RESTRUCTURING OF THE COURSE “FUNDAMENTALS OF COMPUTER APPLICATION” BASED ON COLLEGE STUDENTS’ COGNITIVE PSYCHOLOGY

Heng Ding ^a^

^*a*^ *School of AI and Innovative Design, Beijing Institute of Fashion Technology, Beijing 100084, China.*

**Background:** In recent years, the Course “Fundamentals of Computer Application” has been criticized, students’ satisfaction with the current teaching content and methods of this course is often very low. There are growing concerns about how learning this course can help them keep pace with the rapid development of information technology and adapt to the changing demands of information literacy in their respective industries and professions. Particularly with the rise of artificial intelligence, this anxiety has become more pronounced. As a result, the call for restructuring the course “Fundamentals of Computer Application” is growing increasingly stronger. So in 2022, Beijing Institute of Fashion Technology formed a research group to conduct a comprehensive review of the Course “Fundamentals of Computer Application” and carried out reform and innovation attempts with the performance major as a pilot, achieving certain results.

**Subjects and Methods:** To address students’ course-related anxiety, the restructuring of the course “Fundamentals of Computer Application” employ a three-phase methodology: investigation, analysis, and restructuring. Phase 1: Investigation involves organizing student symposiums and distributing questionnaires to examine students’ psychological states, learning needs, and course-related concerns. Phase 2: Analysis entails synthesizing survey results, reviewing recent data on student attendance, assignments, and exam performance, and evaluating advancements in information technology. It further includes analyzing employer expectations regarding employees’ information literacy, identifying gaps in teaching plans, syllabi, instructional methods, and textbooks, and critically assessing the influence of information technology and applications on performance majors. A key focus is exploring the significance of the course “Fundamentals of Computer Application” for students in performance-related disciplines. Phase 3: Restructuring focuses on redesigning the curriculum, formulating actionable strategies to elevate teaching quality and outcomes in the course “Fundamentals of Computer Application”, and implementing these reforms. This structured approach ensures alignment with evolving technological trends, industry demands, and the unique educational needs of specialized student groups.

**Results:** Following the restructuring of the Course “Fundamentals of Computer Application”, students’ course-related anxiety has been substantially alleviated, leading to renewed perceptions of the curriculum. Students have demonstrated significant improvements in learning motivation, academic performance, problem analysis and problem-solving abilities, with the course receiving highly positive evaluations from students. Notably, students majoring in performance have achieved outstanding results by winning awards in prestigious national competitions in highly competitive fields such as information technology and art design, leveraging the knowledge and skills acquired through the course.

**Conclusions:** Three years of practice have demonstrated that the restructuring of the Course “Fundamentals of Computer Application” for performance majors has proven successful, It not only effectively alleviates students’ learning anxiety, but also has valuable reference and promotion significance for similar academic initiatives.

## PHHS25153: ENVIRONMENTAL POLLUTION, HEALTH RISKS, AND PREVENTION STRATEGIES OF ORGANOPHOSPHORUS PESTICIDES

Xianjun Zeng^a^, Tao Zi^a^, Dengkui Liu^a^

^*a*^ *Hunan Biological and Electromechanical Polytechnic, Changsha, Hunan 410127, China.*

**Background:** Organophosphorus pesticides (OPPs) are extensively utilized in global agricultural production systems owing to their high efficacy and broad-spectrum insecticidal properties. However, with the continuous expansion of their application scope and frequency, the environmental pollution and health risks associated with OPPs have become increasingly prominent, emerging as critical challenges in global public health and environmental safety. OPPs exhibit strong environmental mobility, affecting ecosystems through multiple media (e.g., soil, water, and air) and accumulating in the food chain, thereby posing potential hazards to human populations. Vulnerable groups, such as children and pregnant women, are particularly susceptible to these adverse effects.

**Subjects and Methods:** This study systematically reviewed and integrated existing literature to elucidate the distribution characteristics of OPPs in environmental media, primary human exposure pathways, and associated health hazards. Emphasis was placed on analyzing exposure risks across diverse populations, including agricultural workers, residents, pregnant women, and children. Additionally, the study explored disparities in regulatory policies, prevention measures, and alternative technologies among different countries and regions. A comprehensive evaluation was conducted on the current applications and developmental potential of green control strategies, such as biopesticides, nanopesticides, rapid detection technologies, and health education initiatives.

**Results:** The findings indicate that OPPs migrate through various environmental pathways (e.g., soil, water bodies, and atmospheric transport), ultimately entering the food chain and leading to widespread exposure among both occupational and non-occupational populations. Chronic low-dose exposure to OPPs shows significant correlations with neurodegenerative diseases, endocrine and metabolic disorders, and reproductive-developmental toxicity. Notably, maternal-fetal exposure may result in persistent neurodevelopmental impairments in offspring. At the regulatory level, developed regions such as the European Union have established robust risk assessment mechanisms and banned high-risk OPPs, effectively mitigating health risks. In contrast, developing countries face prominent challenges, including regulatory delays and improper pesticide use. While green alternatives and intervention measures demonstrate potential, their application remains constrained by technical immaturity, insufficient policy support, and limited market adoption.

**Conclusions:** Future research should prioritize integrated risk assessments of multi-pathway exposures, in-depth exploration of chronic toxicity mechanisms, and global policy coordination and technical collaboration. By integrating scientific innovation, policy guidance, and public engagement, it is possible to reduce OPP-related risks to human health and ecosystems while ensuring agricultural productivity, thereby fostering sustainable synergies between agriculture and public health.

## PHHS25154: HOW DOES STATE CAPITAL REGULATION RESHAPE SOES’ M&A PERFORMANCE? MULTI-LEVEL EVIDENCE FROM INSTITUTIONAL COMPLEMENTARITY AND MANAGERIAL MENTAL HEALTH

Meijun Ning^a^, Xiaobo Ao^b^, Xiaonan Zhao^a^, Hui Lyu^c^

^*a*^ *School of Economics and Management, North China University of Technology, Beijing 100144, China,* ^*b*^ *Beijing National Accounting Institute, Beijing 101300, China,* ^*c*^ *School of Economics and Management, China University of Petroleum (Being), Beijing 102200, China.*

**Objective:** Corporate Mergers and Acquisitions (M&A) are vital for optimizing resource allocation and enhancing operational efficiency. Drawing on China’s unique institutional context, managerial mental health, and the quasi-natural experiment of state capital regulation, this study empirically examines how such regulation affects M&A performance of state-owned enterprises (SOEs).

**Subjects and Methods:** The study utilizes a sample of A-share non-financial listed companies in China from 2003 to 2016. Using the difference-in-differences (DID) method, the study assesses the reform’s impact on M&A performance, with short-term performance measured and long-term performance. Additionally, it explores the moderating mechanisms of marketization, corporate governance, information asymmetry, and managerial mental health on these relationships.

**Results:** Strengthened state capital regulation boosts SOEs’ short-term M&A performance by 0.9%, with long-term gains exceeding 7%. Moderating mechanism tests yield four key findings. Firstly, the effect is stronger in less marketized regions, indicating regulatory intervention can compensate for institutional voids and alleviate managerial decision-making pressures. Secondly, SOEs with weaker governance benefit more, underscoring that external regulation mitigates agency conflicts and reduces psychological stress associated with opportunistic behaviors. Thirdly, higher levels of information asymmetry intensify the reform’s effectiveness, as increased transparency curbs managerial opportunism and mitigates investment anxiety. Fourthly, the enhancement of SOEs’ M&A performance by state capital regulatory policies is more evident in firms with better managerial mental health, unveiling the pivotal role of psychological resources in policy transmission and confirming the existence of a “psychological resource-policy response” chain.

**Conclusions:** This paper innovatively integrates managerial mental health into the analytical framework of state capital regulation effects, proposing a “institutional pressure-psychological resource” transmission mechanism that extends the boundaries of behavioral corporate finance theory. By constructing a “conditional matrix for regulatory effectiveness” (market environment × governance level × information quality × psychological capital) in Chinese context, this study offers crucial insights into China’s “financial services supporting the real economy” reforms and provides policy implications for optimizing mixed governance models in emerging economies. Furthermore, it reveals that managerial mental health is a critical moderating variable in policy transmission effects, presenting a new people-oriented governance perspective for SOE reforms in emerging economies.

**Acknowledgments**

We acknowledge the invaluable financial support provided by Humanities and Social Science Fund of Ministry of Education of China (Grant No. 23YJCZH166) and National Natural Science Foundation of China (Grant No. 72072006).

## PHHS25155: CONSTRUCTION OF A SUSTAINABLE DEVELOPMENT MANAGEMENT SYSTEM FOR DIGITAL INCLUSIVE FINANCE DRIVEN BY NEW QUALITY PRODUCTIVITY: BASED ON BEHAVIORAL SCIENCE

Tian Wang^a^, Jianbang Lin^b^

^*a*^ *South China Business College Guangdong University of Foreign Studies, Guangzhou 510545, China,* ^*b*^ *Nanfang College Guangzhou, Guangzhou 510970, China.*

**Background:** Under the dual drive of global economic digital transformation and the “dual carbon” goal, the study of user decision-making mechanisms from the perspective of behavioral science has become the key to solving the development dilemma of new quality productivity. As a typical practice field of new quality productivity, digital inclusive finance faces sustainability challenges such as user behavior deviation and technological acceptability barriers, which essentially reflect the deep contradiction between traditional economic models and behavioral science laws. In particular, behavioral science issues such as cognitive biases in financial exclusion and psychological account effects in promoting green finance have become core pain points that constrain the development of technology enabled inclusive finance.

**Subjects and Methods:** This study is based on the interdisciplinary interface between behavioral science and artificial intelligence, and aims to construct a digital inclusive finance governance framework with dynamic adaptability. By integrating cutting-edge theories of behavioral economics with behavioral pattern recognition techniques of machine learning, we aim to address the irrational decision-making dilemma in inclusive financial services. The research method adopts a multidimensional behavior analysis strategy: using behavioral experiments to capture cognitive bias characteristics in user technology adoption, revealing the micro mechanism of institutional design through semantic analysis of behavioral intervention in policy texts, and combining multi-agent simulation technology to evaluate the group effect of inclusive policies, forming a full chain behavioral science research paradigm of “individual behavior modeling group interaction deduction system resilience assessment”.

**Results:** The operational efficiency of digital inclusive finance. The “behavior technology” collaborative mechanism based on digital boosting technology effectively alleviates the scale efficiency paradox of traditional inclusive finance; The inclusive service plan designed through the framework effect of social norms significantly promotes the market acceptance of green financial products. However, research has also revealed potential conflicts between technological tools and behavioral patterns: the lack of algorithm transparency may lead to user behavioral adaptation barriers, verifying the necessity of synergistic optimization between technological interpretability and behavioral cognitive patterns. The analysis of institutional elasticity shows that dynamically adjusted behavioral incentive strategies can effectively balance technological innovation and social value goals.

**Conclusions:** The theoretical contribution of this study lies in establishing the structural position of behavioral science in the development of new quality productivity, and constructing a digital financial governance paradigm of “technology behavior system” tripartite symbiosis. The design principles of “cognitive adaptation” services and the development guidelines of “behavior friendly” technologies proposed at the practical level provide actionable solutions to solve the challenges of sustainable development in inclusive finance.

**Acknowledgments**

This work is supported by “Interdisciplinary Integration and Innovation” Project and the First-Batch Research Project of the Doctoral Workstation from Guangdong University of Foreign Studies South China Business College (Grant No. 2024XJZ02), a project grant from 2021 Guangdong Key Discipline Research Ability Improvement Project (Grant No. 2021ZDJS129), and Department of Education of Guangdong Province, Research Team on Digital-real Integration and Management Innovation (2023WCXTD023).

## PHHS25156: IMPLEMENTATION PATHWAYS OF POLITICAL EDUCATION BASED ON THE PSYCHOLOGICAL NEEDS OF CHILDREN AND ADOLESCENTS

Dingcai Liang^a^, Yuting Chen^a^, Weizhong Lou^a^, Dingnian Liang^b^

^*a*^ *School of Law and Political Science, Yunnan University of Finance and Economics, Kunming 650221, Yunnan, China,* ^*b*^ *Mengzi City Development and Reform Commission, Honghe 661100, Yunnan, China.*

**Background:** Children and adolescents, as the future pillars of the nation, are in a critical period of psychological and cognitive development. In today’s rapidly changing and complex social environment, coupled with the diversification of information dissemination channels, their mental health and value formation face unprecedented challenges. The emotional volatility, cognitive immaturity, and heightened sensitivity to external influences characteristic of this demographic make them particularly vulnerable to negative societal impacts. Therefore, implementing scientifically grounded and effective political education tailored to their psychological needs is not only urgent but also essential for fostering their mental well-being and cultivating a strong sense of national identity. Political education must address these unique psychological traits to ensure its relevance and effectiveness, thereby supporting the holistic development of children and adolescents.

**Subjects and Methods:** This study focuses on children and adolescents, employing a speculative research methodology to explore the intrinsic relationship between their psychological needs and political education. By integrating theoretical deduction and rigorous logical analysis, the study examines how political education can be adapted to align with their cognitive, emotional, and self-awareness development stages. Special attention is paid to the mental health implications of political education, particularly how it can mitigate emotional instability and enhance cognitive resilience. The methods include analyzing existing literature on developmental psychology and political education, as well as synthesizing insights from educational practices that prioritize psychological well-being. This approach ensures that the proposed strategies are both theoretically sound and practically feasible.

**Results:** The findings reveal that political education grounded in the psychological needs of children and adolescents must adopt a multi-faceted approach to be effective. First, it requires a content system tailored to their cognitive abilities, ensuring that the material is neither overly complex nor simplistic, thereby reducing cognitive stress and promoting mental engagement. Second, interactive and engaging pedagogical methods, such as experiential learning and group discussions, are crucial for maintaining their interest and emotional investment, which are vital for mental health. Third, creating a positive educational milieu-one that fosters a sense of belonging and emotional security-can significantly enhance the internalization of political values. Fourth, focusing on individual differences and psychological counselling for children and adolescents. These strategies collectively contribute to improved mental health outcomes by reducing anxiety, fostering emotional stability, and strengthening self-identity.

**Conclusions:** In conclusion, political education that aligns with the psychological needs of children and adolescents is not only more effective but also instrumental in promoting their mental health and well-being. By constructing age-appropriate content, employing interactive teaching methods, and cultivating a supportive environment, such education can help young individuals develop correct political values and a robust national identity while addressing their emotional and cognitive vulnerabilities. This holistic approach ensures that political education serves as a tool for both ideological cultivation and psychological resilience, ultimately preparing children and adolescents to become well-rounded and mentally healthy contributors to society. The study underscores the importance of integrating mental health considerations into political education to achieve long-term educational and societal goals.

**Acknowledgments**

The research was supported by Yunnan University of Finance and Economics Scientific Research Funding Program (2022C13) and Yunnan University of Finance and Economics 2024 Curriculum Civics Demonstration Course Construction Project.

## PHHS25157: SOCIAL ENTERPRISE CHARITY IN CHINA: A NEW CHARITY MODEL TO PROMOTE THE MENTAL HEALTH OF DISADVANTAGED GROUPS

Yanli Wang^a^, Yanzhuo Wang^b^

^*a*^ *Jilin Agricultural University, Changchun 130118, China,* ^*b*^ *Jilin Jianzhu University, Changchun 130118, China.*

**Background:** As an important part of China’s social security system, charity plays an important role in solving social problems, helping disadvantaged groups, and improving individual mental health. The development of charity in China has gone through a process from individual charity to organizational charity to social enterprise charity. In this process, the forms and functions of charity have continuously enriched, and its role has shown new characteristics.

**Subjects and Methods:** With the continuous development of China’s economy and society and the increasing exposure of the shortcomings of traditional charitable organizations, social enterprise charity has emerged. China’s institutional environment, cultural foundation and the growth of its talent pool have also created realistic conditions for the emergence of social enterprise charity in China. On this basis, social enterprise charity has demonstrated unique value and potential in promoting the mental health of disadvantaged groups. The case study method is adopted, and Canyou Group is chosen as a case study to analyze the essential connotation and advantages of social enterprise charity in China, in order to reveal the positive role of social enterprise charity in improving the mental health level of disadvantaged groups and building a harmonious society.

**Results:** Compared with traditional charities, China’s social enterprise charities have significant advantages in terms of charitable concept, charitable behaviors, and charitable scope, and pay more attention to the mental health education of disadvantaged groups while focusing on the material needs of disadvantaged groups. Specifically, charitable concept has been upgraded from survival to development; charitable behaviors have been changed from passive to proactive; and charitable scope has been expanded from “small charity” to “big charity”.

**Conclusions:** Social enterprise charity is a new model of charity suitable for China’s economic and social development. It combines commercial means with charitable purposes, and provides comprehensive support for disadvantaged groups through innovative service models. Social enterprise charity continuously promotes the mental health of disadvantaged groups, comprehensively improves their quality of life, and promotes the harmonious development of Chinese society by adhering to developmental charitable concept, practicing proactive charitable behaviors, and expanding the charitable scope. The “Canyou Group” social enterprise charity is a typical social enterprise charity.

**Acknowledgments**

This paper was supported by Jilin Science and Technology Development Program Project (20220601119FG).

## PHHS25158: THE INFLUNCE OF COGNITIVE ON THE PSYCHOLOGICAL ATTITUDE OF CORPORATE STRATEGIC DECISION_MAKERS

Chujia Liu ^a^

^*a*^ *Department of Economics and Management, Zhangjiajie University, Zhangjiajie, Hunan 435000, China.*

**Background:** Strategy is the response of an enterprise to changes in the external environment, which indicates the consistency of the external environment of the organization with the internal structure and process, which is crucial to the survival and development of the enterprise. In the study of strategic decision-making, “what role do the decision-makers play” has always been the focus of debate. In the face of different external environmental conditions, the personal characteristics and behaviors of the psychological needs and anxiety of the top decision makers of enterprises play a key role in the strategic decision of enterprises.

**Subjects and Methods:** This study explores the influence of the psychological needs, anxiety and other cognitive characteristics of decision makers on the process of strategic decision making and the final strategic decision making. This study used the method of scenario case matching with questionnaire survey. Researchers generally describe the decision-making problems facing a business in an uncertain environment. Based on the distributed questionnaire and recovered the questionnaire, we analyze the data by using the Likert analysis model then we obtained the following results.

**Results:** We find that the cognitive complexity and psychological needs of decision makers have a significant positive relationship with their thorough analysis of the internal and external environment of the business. At the same time, we also found that enterprises with high cognitive complexity of decision makers are more likely to make decision-making innovation, which strengthening the promoting effect of cognitive complexity on decision-making innovation and the inhibitory effect of concentration on decision-making innovation.

**Conclusions:** Decision-makers’ psychological needs, anxiety and other cognitive bias have significant attitudes to shape enterprise strategic decision-makers by the aid of systematic thinking errors. The research conclusion reveals the internal mechanism of the dynamic adaptation to the external environment of the decision makers, which has an important guiding significance for the enterprise decision makers.

**Acknowledgments**

This work was supported by a project grant from Zhangjiajie Social Project (2024.7).

## PHHS25159: PHARMACEUTICAL INNOVATION AND PUBLIC HEALTH: A STUDY ON THE IMPACT OF R&D INVESTMENT ON FINANCIAL PERFORMANCE IN CHINA’S PHARMACEUTICAL MANUFACTURING COMPANIES

Rui Ji^a^, Yukun Li^a^, Jianyu Chen^a^, Yanxin Chen^a^

^*a*^ *School of Economics and Management, Sichuan Tourism University, Chengdu 610100, China.*

*RJ and YL contributed equally to this study.*

**Background:** The rapid development of healthcare in China has led to a significant increase in public health awareness, with health gradually becoming a core concern for the population. Concurrently, the health system is undergoing constant evolution and transformation. This has contributed to the enhancement of the healthcare security system, resulting in a substantial increase in demand for high-quality medicines. Furthermore, from the vantage point of health policy, the government has adopted a more stringent regulatory approach for pharmaceuticals, prioritizing their safety, efficacy, and controllable quality. This shift has engendered a series of challenges for pharmaceutical companies, compelling them to augment their investment in healthcare R&D and assume the financial risks associated with protracted and uncertain R&D cycles.

**Subjects and Methods:** This study employs empirical regression analysis to examine the impact of R&D investment on the financial performance of the pharmaceutical industry and the healthcare sector as a whole, and further explores its implications for the sustainability of the pharmaceutical industry. We analyzed data from 487 listed Chinese pharmaceutical companies on healthcare R&D over the period 2014-2023.

**Results:** The study assesses the immediate and the lagged effects of R&D investment on financial performance and analyzes the heterogeneity according to the level of leverage. The findings indicate that an escalation in R&D intensity exerts a negative impact on Chinese pharmaceutical companies’ short-term financial performance, while exerting a positive influence in the long run. This positive influence is more significant for companies with leverage ratios above the industry average which derive greater long-term benefits from R&D. Conversely, companies with leverage ratios below the industry average encounter heightened short-term financial pressures.

**Conclusions:** In order to ensure that investments in research and development are aligned with health system objectives, policymakers should consider implementing tax incentives rather than direct subsidies to channel research and development activities in the pharmaceutical industry. At the same time, intellectual property protection needs to be strengthened to ensure returns on innovation, and risk-sharing mechanisms need to be put in place for high-cost innovative pharmaceutical companies. These measures have the potential to reduce the financial pressure on the pharmaceutical industry and to significantly enhance the technological level and innovation capacity of the pharmaceutical industry will be strengthened, which in turn will enhance the capacity and efficiency of the medical industry to deliver services in the area of public health. These measures are pivotal to advancing the deepening reform of China’s health policy, not only helping to optimize the allocation of medical resources, but also ensuring that the general public has equal access to cutting-edge, innovative health resources and medical solutions, thereby enhancing the overall level of national health.

**Acknowledgments**

This work was supported by Chengdu Cultural and Economic Research Center, Project number: CE202436. Sichuan Key Laboratory of Philosophy and Social Sciences, Key Laboratory of Sichuan Cuisine Artificial Intelligence, Project number: CR24Y12.

## PHHS25160: NATURE SPIRITUALITY IN CAROL ANN DUFFY’S POETRY

Yuan Liu^a^, Haoyu Wang^b^

^*a*^ *Shandong Communication and Media College, Jinan, China,* ^*b*^ *Nankai University, Tianjin, China.*

**Background:** To explore how former British Poet Laureate Carol Ann Duffy’s nature poetry functions as a kind of spiritual solace from the perspective of Nature Spirituality in an age of spiritual loss.

**Subjects and Methods:**

A total of eleven poems by Carol Ann Duffy were chosen as subjects of study. The analyses focused on the presentation of nature spirituality in her early poem “Fifth Last Song”, spiritual animism in “The Dolphins” and “Elephants”, vegetarianism in “A Healthy Diet” and “Blackbird”, Gaian spirituality in “Atlas”, nostalgia for pastoral life in “Silver Lining”, “Scarecrow”, “The Creation of Adam and Eve” and “Forest”, and finally came to the conclusion that Carol Ann Duffy has shown a tendency to seek for spiritual solace from Nature in an age of religious doubt, with reference to her poem “Prayer”.

Close reading has been the main method of study, with its theoretical basis on Bron Taylor’s idea about Nature Spirituality, including the categorization of Spiritual Animism, Naturalistic Animism, Gaian Spirituality and Gaian Naturalism.

As another method of study, references are made to the partly religious and partly secular symbolic meaning of bees in Duffy’s poetry as spiritual immortality analyzed by Angelica Michelis, the eco-feminist ideas in Duffy’s poetry analyzed by Jie Zhou, and Bekoff’s idea of the emotional lives of animals etc.

**Results:** A close reading of Duffy’s poem “Fifth Last Song” shows her early presentation of nature spirituality. Later poems such as “The Dolphins” and “Elephants” show her spiritual animism. Vegetarianism is found in “A Healthy Diet” and “Blackbird”. Gaian spirituality is revealed in “Atlas”. Nostalgia for pastoral life is expressed in “Silver Lining”, “Scarecrow”, “The Creation of Adam and Eve” and “Forest”. The final result is that nature can become a source of solace for people in want of a firm religious belief.

**Conclusion:** After analysis of nature spirituality in 10 poems by Carol Ann Duffy, an association of her life context is made. It is concluded that in an age of loss of religious belief Carol Ann Duffy’s nature poetry shows nature as people’s spiritual solace, because they echo Bron Taylor’s idea of Nature Spirituality, which includes Spiritual Animism, Naturalistic Animism, Gaian Spirituality and Gaian Naturalism.

**Acknowledgments**

This research was supported by National Social Sciences Research Project “Stylistic Study of Carol Ann Duffy’s Poetry” (17BWW060).

## PHHS25161: RESEARCH ON THE ELDERLY CARE SECURITY OF ELDERLY CARE COIN IN MEETING THE PSYCHOLOGICAL NEEDS OF THE LOW-INCOME ELDERLY PEOPLE BASED ON SOCIAL SUPPORT THEORY

Youmo Tan^a^, Meng Lv^b^, Chao Zhang^c^

^*a*^ *School of Finance and Economics, Guangxi Science & Technology Normal University, Laibin, Guangxi 546199, China,* ^*b*^ *School of Mathematics and Computer Engineering, Guangxi Science & Technology Normal University, Laibin, Guangxi 546199, China,* ^*c*^ *Trade Finance Department of Liuzhou Bank Co., Ltd, Liuzhou, Guangxi, China.*

**Background:** China has entered a stage of deep aging. The huge elderly population will create a huge demand for elderly care services. For low-income elderly groups, their psychological needs in elderly care services are often overlooked yet are of great significance. These elderly people, due to their limited economic resources, not only face challenges in meeting basic material needs but also experience unique psychological stress. This study proposes the digital virtual currency of elderly care coin, which can be used as pension funds to improve the ability of elderly people, especially low-income elderly people, to purchase elderly care services, increase the demand and the supply of social elderly care services.

**Subjects and Methods:** The subject of this study is the low-income elderly population with a monthly per capita disposable income of less than 1000 yuan. This study uses quantitative analysis method and collected data of 450 responses, and then analyzes the role of elderly care coin in providing elderly care security for the elderly people using Structural Equation Modeling (SEM) by SPSSAU.

**Results:** The results show that the innovation and exchange of elderly care coins significantly influence the establishment of the elderly care coin service model. For low-income individuals, elderly care coin can meet their psychological need for elderly care security in the context of limited resources. The participation of social support forces not only provide practical assistance but also address the psychological needs of low-income elderly for social connection and recognition. The model’s establishment gives low-income elderly a sense of stability and predictability, fulfilling their psychological need for security. Social support forces function as an intermediary, bridging the gap between the innovation of elderly care coins and the realization of psychological well-being for the elderly.

**Conclusions:** Based on the social support theory, this study analyzed low-income groups in China. It was found that elderly care coins are innovatively feasible. The innovation and exchange of elderly care coins affect the construction of the elderly care coin service model and the participation of social support forces. These aspects not only have practical implications but also play a role in meeting the psychological needs of low-income elderly. The elderly care coin model can enhance their sense of self-worth, and the increased social support can reduce feelings of isolation. It exerts a positive impact on the elderly’s access to elderly care security, both in terms of material resources and psychological well-being.

## PHHS25162: THE IMPACT OF ABUSIVE SUPERVISION ON EMPLOYEE MENTAL HEALTH: ROLES OF PSYCHOLOGICAL CONTRACT BREACH AND JOB CRAFTING

Wei Zhong^a^, Bolin Wang^a^

^*a*^ *Department of Hotel Management, Shanghai Business School, Shanghai, China.*

**Background:** This study aims to investigate the impact of abusive supervision on the mental health of frontline hotel employees from a psychological perspective, focusing on the mediating roles of perceived psychological contract breach (PPCB) and job crafting in the relationships between abusive supervision, job satisfaction, and organizational citizenship behavior (OCB). The study explores how abusive supervision affects employees’ psychological needs, cognitive and behavioral processes, and overall well-being.

**Subjects and Methods:** Using a three-wave longitudinal survey of frontline employees from five-star hotels, this study employed structural equation modeling to analyze the relationships among abusive supervision, PPCB, job crafting, job satisfaction, and OCB. The study examined the negative psychological effects of abusive supervision, such as increased stress, anxiety, and psychological distress, and the psychological regulatory mechanisms of PPCB and job crafting from cognitive and emotional perspectives. The study also considered the influence of social psychological factors on employee responses to abusive supervision.

**Results:** The findings indicate that abusive supervision hinders employees’ job crafting efforts, leading to reduced job satisfaction and diminished OCB toward the organization, coworkers, and customers. Moreover, PPCB mediates the relationship between abusive supervision and job satisfaction but not between abusive supervision and OCB. From cognitive and emotional perspectives, abusive supervision triggers negative emotional responses such as stress, anxiety, and insecurity, subsequently damaging psychological contracts and weakening job crafting motivation. The study highlights the detrimental effects of abusive supervision on employees’ psychological well-being and the importance of considering psychological factors in understanding employee behavior.

**Conclusions:** This study enriches the literature by elucidating the mechanisms through which abusive supervision affects hotel employees’ mental health and performance from a psychological standpoint. The findings emphasize the importance for hotel managers to mitigate abusive supervision, promote job crafting, and maintain psychological contracts to foster a positive and healthy work environment that protects employees’ mental and physical well-being. The hospitality industry should prioritize employees’ psychological needs and cognitive-emotional processes, implementing effective psychological management strategies to alleviate the negative impacts of abusive supervision. Future research could further explore the role of other psychological factors, such as resilience and emotional intelligence, in the relationship between abusive supervision and employee behavior.

**Acknowledgments**

This work was supported by the Shanghai Municipal Education Science Research Project (Grant Number: C2025143).

## PHHS25163: THE USE OF A BACK PROPAGATION NEURAL NETWORK ALGORITHM INCORPORATING MENTAL HEALTH FACTORS IN A TEACHER-STUDENT INTERACTION EVALUATION SYSTEM

Zheng Li^a^, Liping Nie^b^

^*a*^ *Yangtze River Delta Economic and Industrial Development Research Center, Shanghai Lida University, Shanghai, China,* ^*b*^ *TIANHUA College, Shanghai Normal University, Shanghai, China.*

**Background:** In the increasingly diverse and complex educational environment, students’ mental health has garnered growing attention. Psychological variables such as anxiety levels, personality traits, and psychological needs significantly impact classroom engagement and the quality of teacher-student interactions. These interactions not only affect teaching effectiveness but also directly influence students’ emotional regulation, sense of belonging, and psychological adaptability. Establishing a scientific and predictive evaluation mechanism for teacher-student interactions can help educators identify students’ psychological states during the learning process. This enables targeted adjustments to teaching strategies, and facilitates the simultaneous improvement of mental well-being and learning outcomes.

**Subjects and Methods:** This work integrates psychological health perspectives with the Analytic Hierarchy Process (AHP) and the Back Propagation Neural Network (BPNN) algorithm to develop a predictive model for assessing both teaching effectiveness and the quality of teacher-student interactions. Behavioral indicators related to students’ psychological needs, such as proactive feedback, emotional resonance, and communicative engagement, are incorporated into the evaluation framework, reflecting their weight and influence at different instructional stages. Data from the National Education Statistics Database are used. K-fold cross-validation and min-max normalization techniques are applied to model and predict the behavioral characteristics of 355 students, and enhance model stability and the accuracy of psychological behavior assessment.

**Results:** Findings indicate that the cognitive processing stage of teacher-student interaction has the greatest impact on teaching effectiveness evaluation, with a weight of 0.337. This stage typically involves task challenges, cognitive stimulation, and emotional investment, closely related to students’ anxiety coping ability, autonomy, and sense of belonging. Among behavioral indicators, “interpretative exploration,” “case analysis,” and “task resolution” hold significant weight, which underscores the importance of higher-order cognition and emotional engagement in interaction quality. In contrast, “dialogue and communication” has a relatively lower weight (0.105), suggesting that routine interactions provide limited support for psychological regulation. Predictive experiments show that the BPNN model exhibits minimal prediction errors in most cases, and it can effectively capture the comprehensive impact of teacher-student interactions on students’ psychological states and teaching effectiveness.

**Conclusions:** This work develops a predictive evaluation model for teacher-student interactions that integrates a mental health perspective. It incorporates psychological variables such as emotional responses, anxiety levels, and interpersonal communication traits into teaching assessment, thereby expanding the research scope of educational evaluation. The AHP method ensures a scientifically grounded weighting of indicators, while the BPNN algorithm enhances the modeling of dynamic psychological behavior changes. This model serves as an auxiliary tool for teachers to identify changes in students’ psychological states. Moreover, it provides education administrators with a new approach to monitoring teaching quality from a mental health perspective, and offers significant practical value for fostering a more human-centered educational environment.

## PHHS25164: EXPLORING THE EFFECTIVENESS OF COMPLEX FUNCTIONS AND INTEGRAL TRANSFORMS IN CLASSROOM UNDER THE PERSPECTIVE OF STUDENTS’ PSYCHOLOGICAL NEEDS

Jiancheng Chi^a^, Juan Wang^a^

^*a*^ *Anhui Sanlian University, Hefei, Anhui 230601, China.*

**Objective:** This article focuses on the classroom effectiveness of a course on functions of a complex variable and integral transforms. From the perspective of students’ psychological needs, it eliminates students’ psychological anxiety in learning, enhances students’ interest in learning, strengthens students’ self-confidence and motivation in learning mathematics, and improves the effectiveness of classroom teaching in order to achieve the goal of achieving students’ psychological needs.

**Subjects and Methods:** for the current teaching of this course in the theoretical abstract knowledge points, in-class knowledge and practical application of the link is not close, students for the knowledge learned fear, psychological anxiety and classroom participation is not high phenomenon, the authors proposed to optimise the content of classroom teaching, focus on the integration of the course theory and practical problems, the learning process will be the integration of this course and the students’ professional course knowledge, the formation of the course teaching cases, to enhance students’ intimate knowledge of this course, to enhance students’ interest in learning mathematics. In order to enhance the students’ sense of intimacy for this course. In addition, the reform of traditional teaching methods to improve classroom efficiency, teachers can use mathematical software to visualise the knowledge points learned, enhance students’ intuitive psychological feelings, eliminate students’ psychological anxiety. Teachers use the flipped classroom to reform the mode of classroom teaching, handing over the initiative of the classroom to the students, so as to improve the learning efficiency of the students. Improve the evaluation system of the course, enhance students’ self-knowledge and subject position, and realise students’ psychological needs.

**Results:** After practical verification and optimisation of the method, students’ enthusiasm for learning the course has been greatly improved, and they are more willing to spend more time and energy on discussion and communication, which leads to a great progress in the learning of professional courses accordingly. The application and intuition of the course increased at the same time, but also let many students clear what practical significance and value of the learned knowledge points, broaden the students’ learning horizons.

**Conclusions:** This teaching practice provides new research ideas for the classroom effectiveness of complex functions and integral transforms, improves teaching methods and learning paths, eliminates students’ psychological anxiety, and realises students’ recognition of their self-worth.

**Acknowledgments**

This work was supported by a project grant from General Project of Teaching and Research, Department of Education of Anhui Province (Grant No. 2023jyxm0896).

## PHHS25165: THE ASSOCIATION BETWEEN HERPES SIMPLEX VIRUS AND CERVICAL CANCER: MENDELIAN RANDOMIZATION ANALYSIS

Jialing Shi^a^, Lin Kuang^a^, Wei Lu^a^, Wei Feng^a^, Qi Li^a^

^a^ *School of Integrated Chinese and Western Medicine, Hunan University of Chinese Medicine, Changsha 410208, China.*

**Background:** Herpes simplex virus (HSV) mainly includes type 1 herpes virus (HSV-1) and type 2 herpes virus (HSV-2). Clinically, it mainly causes skin diseases, with erythema, vesicles, erosion, papules, exudation and burning pain of skin mucosa as the main manifestations. However, in recent years, numerous observational studies have suggested a possible association between HSV infection and the development of cervical cancer (CC). This study aimed to determine whether a causal relationship exists between HSV infection and CC, so as to alert the possibility of CC in HSV patients.

**Subjects and Methods:** We performed a systematic two-sample Mendelian randomization (TSMR) analysis using three pooled-level genome-wide association study (GWAS) datasets from publicly available databases to explore the association between HSV infection and CC. We used five methods for evaluation, including inverse variance weighting (IVW), MR-Egger, weighted median (WM), simple mode and weighted mode, with IVW as the main analysis method. Prospective MR analysis was performed using IVW and sensitivity analysis.

**Results:** The MR results showed no causal association between circulating HSV of genetic origin and CC ([OR] = 1.000; 95% [CI]:0.999–1.001; P =.659), or between HSV-1 and CC([OR] = 1.001; 95% [CI]: 0.998–1.004; P =.648), or between HSV-2 and CC ([OR] = 1.000; 95% [CI]: 0.993–1.007; P =.047). Sensitivity analysis did not find heterogeneity and level multiplicity.

**Conclusions:** In this study, we used Mendelian randomization to investigate the relationship between HSV infection and CC incidence. The results of Mendelian randomization showed no causal relationship between HSV infection and the onset of CC. Our study remains limited by the insufficient data in the GWAS database and the single ethnic origin of the data source. This research explores the causal relationship between HSV and the onset of CC from a genetic perspective, which has significant scientific value and practical implications.

**Acknowledgments**

This study was supported by the National Natural Science Foundation of China (82174391).

## PHHS25166: RESEARCH ON TACIT KNOWLEDGE SHARING STRATEGY OF UNIVERSITY TEACHERS FROM THE PERSPECTIVE OF PSYCHOLOGICAL CONTRACT

Ming Wei^a^, Chengming Zhao^b^, Lin Zhang^a^, Zihong Chen^a^

^*a*^ *Faculty of Modern Health Care, Anhui Sanlian University, Hefei, Anhui 230601, China,* ^*b*^ *School of Business, Anhui University, Hefei, Anhui 230601, China.*

**Objective:** Tacit knowledge is the core competitiveness of university teachers, and its sharing is of great significance for improving the scientific research and innovation ability and teaching quality of universities. The purpose of this study is to explore the main obstacles of tacit knowledge sharing among college teachers when they reach a psychological contract between college teachers and students, and put forward corresponding reform strategies. This study provides theoretical support and practical guidance for university management by specifically analyzing the relationship between psychological contract types and tacit knowledge sharing willingness.

**Subjects and Methods:** The research focuses on the different types of psychological contracts of college teachers and analyzes their influence on the willingness of tacit knowledge sharing. Through the variable model construction method and case analysis method, this paper analyzes the relationship between the type of psychological contract and the willingness of tacit knowledge sharing. By combining the knowledge sharing information in teaching with the analysis of the corresponding scheme of the new index, the specific key reform scheme is refined, and then the teaching improvement is compared with the actual operation, and then the quantitative data of the feedback is obtained by issuing and collecting the quantitative table to the students, and the feasibility of the relevant scheme is analyzed and verified by combining the quantitative data and the in-depth experience data and information, so as to supplement and verify the whole research.

**Results:** The study found that through a series of programs that teachers can form a community of interests with students, teachers and students can establish a knowledge circle and create a sense of knowledge security, which can effectively enhance the activation of college teachers’ expectations and trust in students’ learning knowledge. Specifically, relational psychological contract significantly enhances the willingness to share tacit knowledge by enhancing teachers’ sense of trust and psychological capital.

**Conclusions:** The type of psychological contract has a significant impact on the willingness of university teachers to share tacit knowledge. Colleges and universities should optimize the management of psychological contract, strengthen the construction of relational psychological contract, and promote tacit knowledge sharing by establishing trust relationship and improving teachers’ psychological capital. At the same time, colleges and universities should create an open knowledge sharing culture, provide incentive mechanism and sharing platform to further enhance teachers’ willingness to share tacit knowledge.

**Acknowledgments**

This work was supported by a project grant from the Modern Health and Wellness Course Teaching Innovation Team, Anhui Provincial Quality Engineering Project (2023cxtd114), Robot Engineering Teaching Innovation Team, ANHUI SANLIAN UNIVERSITY Teaching Innovation Team Project (24zlgc070), Exploration and Practice of Cultivating Mechanical Structure Design Ability for Robotics Engineering Students, ANHUI SANLIAN UNIVERSITY Quality Engineering Key Project (23zlgc101), Excellent Engineer Education and Training Program for Electrical Engineering and Intelligent Control, Anhui Provincial Quality Engineering Project (2022zybJ035).

## PHHS25167: RESEARCH ON DECISION-MAKING ANXIETY OF MATHEMATICS EDUCATION REFORM IN PRIMARY SCHOOLS EMPOWERED BY MATHEMATICS INTELLIGENCE

Chuanxi Wang ^a^

^*a*^ *Fenghuang Lake Primary School, Chongqing, 402160, China.*

**Background:** From the standardized knowledge transfer in the early days of the founding of New China, to the cultivation of practical ability in the new curriculum reform period, and to the cultivation of innovative talents in the new era, primary school mathematics education has undergone three major transformations. At present, digital intelligence technology, that is, digital technology + intelligent algorithm, is reconstructing the underlying logic of primary school mathematics education with its precision, visualization and interaction characteristics. It has become the key to solving the problem of new curriculum standards and the key to anxiety.

**Methods:** First of all, the literature analysis method is certainly needed to understand the current situation and theoretical basis. Then empirical research, such as experimental methods, may be needed to compare the teaching effects of traditional teaching and number intelligence empowerment. The case study is also good, and several schools can be selected as cases for in-depth analysis. Questionnaires and interviews can be used to collect feedback from teachers and students, understand actual needs and overcome anxiety. There is also big data analysis, because it involves digital intelligence technology, there may be analysis of learning data, such as students’ questions, interactive records, etc. The action research method may be appropriate to involve teachers in adjusting strategies in practice. The comparative research method can compare the effects in different situations horizontally or vertically. Expert consultation method, looking for experts in the field to provide advice, to ensure the scientific nature of the study, is conducive to decision-making.

**Results:** Through literature analysis, empirical research, mixed methods, big data analysis and expert consultation, the influence of mathematical intelligence empowerment on primary school mathematics education is proved. The conclusions of quantitative and qualitative analysis of mixed methods are revealed through big data analysis. Some characteristics and models provide decision-making research for the reform of primary school mathematics education and change the anxiety trajectory.

**Conclusion:** The evaluation of primary school mathematics should focus on the shift from ‘result-oriented’ to process-oriented’. In terms of migration ability, we should consider whether mathematical knowledge can be applied to new situations, analyze social phenomena with statistical methods, and overcome people’s anxiety about this cognition. In terms of cognitive structure, it pays attention to whether students form a systematic knowledge network, whether the perimeter of geometric figures can be associated with the area formula, and in terms of thinking strategy, it pays attention to whether students use the process of higher-order thinking such as logical reasoning and abstract generalization to deduce the results of change and optimize decision-making, so as to overcome panic.

## PHHS25168: APPLICATION OF FULL ACCOMPANYING NURSING CARE FOR PATIENTS UNDERGOING CARDIAC SURGERY

Lijuan Peng^a^, Xiuqin Zhou^a^, Wenfeng Xue^a^

^*a*^ *3201 Hospital of Xi’an Jiaotong University Health Science Center, Shaanxi 723000, China.*

**Background:** As an emerging nursing model, full accompanying nursing care for patients provides care and support to patients throughout the surgical process and postoperative period, particularly for a common but high-risk medical process such as cardiac surgery. However, traditional nursing models are often limited to the short term post-surgery, ignoring the long-term psychological and physiological needs of patients after surgery. Therefore, this study explored the application effects of full-process companionship nursing in patients undergoing cardiac surgery, aiming to provide a scientific basis and practical guidance for improving patient rehabilitation, promoting mental health, and optimizing the utilization of medical resources.

**Subjects and Methods:** Application of full accompanying nursing care for patients undergoing cardiac surgery, evaluate their clinical outcomes, and draw conclusions accordingly. 126 patients who underwent cardiac surgery in the hospital from March 2023 to December 2, 2023 were selected as the study subjects and divided into a control group (n=63) and an observation group (n=63). Control group patients received routine nursing care, while observation group patients received full accompanying full accompanying nursing care based on the implementation of routine nursing care. The two groups were assessed on condition indicators, depression self-rating scale (SDS), anxiety self-rating scale (SAS), chronic cardiac insufficiency quality of life scale (LHFQ), complications, and nursing satisfaction.

**Results:** All disease indicators, SDS, SAS, and LHFQ scores in the observation group were lower than those in the control group, and there were differences between the groups (P<0.05). The total incidence of complications in the observation group was 7.94%, which was lower than the control group’s 31.75%, and there was a significant difference between the groups (P<0.05). The nursing satisfaction rate of the observation group patients was 98.41% higher than that of the control group 76.19%, and there was a significant difference between the groups (P<0.05).

**Conclusions:** The application of full-time accompanying care for patients undergoing cardiac surgery has had a positive impact on many aspects of the patient, not only optimizing disease indicators and the incidence of complications, but also significantly improving patient satisfaction with nursing care, thereby providing strong support and guarantee for the patient’s health recovery and is expected to become an important mode of care in future clinical practice.

## PHHS25169: TEACHING OF POLITICAL AND ACADEMIC UNITY IN VOCATIONAL IDEOLOGICAL AND POLITICAL COURSES BASED ON STUDENT PSYCHOLOGICAL NEEDS

Jing Chang^a^, Dongxue Zhai^b^, Jun Gao^a^, Cailu Xiao^c^

^*a.*^ *School of Marxism, Shenzhen Institute of Information Technology, 518000 Shenzhen, Guangdong, China,* ^*b*^ *School of Finance and Economics, Shenzhen Institute of Information Technology, Shenzhen 518000, Guangdong, China,* ^*c*^ *Qingdao Central Hospital, University of Health and Rehabilitation Sciences (Qingdao Central Hospital), 266011, Qingdao, Shandong, China.*

**Background:** In the teaching of vocational ideological and political courses, implementing the principle of integrating political and academic nature is crucial for guiding students’ thoughts and promoting their mental health. Vocational students are at a critical stage of personal development, facing pressures and challenges in multiple aspects such as academics, employment, and interpersonal relationships. These factors not only affect their academic performance but may also have potential negative impacts on their mental health. Therefore, as an important platform for nurturing students, ideological and political courses need to pay more attention to students’ psychological needs. By employing scientific and rational teaching methods, these courses can help students establish correct values and a positive mindset, thereby enhancing their sense of psychological security and direction.

**Subjects and Methods:** This paper focuses on vocational students and delves into the intrinsic mechanism of the unity of political and academic nature in vocational ideological and political courses. Through literature reviews and teaching practice research, we analyze existing issues in the current teaching practice of vocational ideological and political courses, such as the lack of academic rationality, the dilution of political nature, and dualism. These issues not only weaken the educational function of ideological and political courses but may also lead to students lacking correct value orientations when facing complex social problems, thereby affecting their mental health. To address these issues, this paper proposes a teaching reform plan oriented by students’ psychological needs. By integrating and transforming discourse patterns, we explore new teaching models and design innovative teaching mechanisms.

**Results:** The research findings indicate that prevalent issues in the current ideological and political course teaching at higher vocational colleges—such as the absence of theoretical depth, diminished political focus, and binary opposition—have exerted latent negative impacts on students’ mental health. These problems lead to confusion and disorientation among students during their learning process, preventing them from achieving theoretical fulfillment and psychological identification. To address these challenges, this paper proposes concrete practical pathways through the “four integrations and transformations” of discourse, aiming to enhance the curriculum’s appeal and stimulate students’ initiative and critical thinking. These pedagogical innovations not only improve teaching effectiveness but also significantly enhance students’ mental well-being.

**Conclusions:** Through the above practical explorations, this paper aims to implement the principle of integrating political and academic nature in vocational ideological and political courses, meet students’ psychological health needs, and promote their comprehensive development. The research results show that the organic combination of political and academic nature can effectively enhance the educational function of ideological and political courses and strengthen students’ identification with and sense of psychological security regarding the courses. This teaching model not only helps students establish correct values and a positive mindset but also improves their rational thinking and problem-solving abilities, laying a solid foundation for their future career development and personal growth.

**Acknowledgments**

This work was supported by a project grant from Guangdong Provincial Educational Science Planning (Moral Education Special Project): “Research on the Teaching Practice of the Unity of Political and Academic Nature in Higher Vocational Ideological and Political Courses” (Grant No. 2024JKDY101).

## PHHS25170: ENGLISH PRONUNCIATION FEATURE EXTRACTION IN HARSH CONDITIONS: A TEMPLATE-BASED AND BEHAVIORAL PERSPECTIVE

Zeng Ju^a^, Jihong Li^a^

^*a*^ *Department of General Education Chongqing Chemical Industry Vocational College, Chongqing, China.*

**Background:** In the information-driven era, intelligent recognition technologies have advanced rapidly. However, in China, the adoption of OCR and similar technologies lags behind foreign counterparts by at least 15 years, particularly in finance and taxation. Behavioral studies highlight cognitive mismatches in non-native contexts: learners’ reliance on native-language processing reduces adaptability to English-centric systems, hindering domestic implementation. Speech recognition faces challenges in evaluating English pronunciation accuracy and non-linguistic elements (e.g., speech rate, intonation). Learners in noisy environments develop compensatory strategies (e.g., exaggerated pitch modulation), which traditional acoustic models often misclassify as errors. Conventional methods also suffer from high data demands, slow training, and computational intensity.

**Subjects and Methods:** This study extracts English pronunciation features in harsh conditions using sound signal template technology. A test environment integrates Mel-frequency cepstral coefficients (MFCC) based on human auditory models and a deep belief network (DBN). Cognitive stressors (e.g., timed tasks) simulate real-world learning adaptations. The Spoken ArabicDigit dataset (UCI) is used, with annotated behavioral metadata (hesitation, breath pauses) to quantify cognitive load effects. An ID3 algorithm constructs a decision tree with five indicators (pitch, rhythm, intonation, speed, emotion), embedding behavioral nodes (e.g., “pauses >0.5s trigger rhythm recalibration”). MFCC and LPCC features are compared via distance classifiers on a Windows XP system with limited hardware.

**Results:** Human-machine evaluation achieved 73.9% accurate consistency and 93.8% adjacent consistency. The latter reflects learners’ intentional clarity adjustments (e.g., 12-15% slower speech in noise), indicating improved alignment when models account for behavioral adaptations. MFCC demonstrated superior noise robustness over LPCC, aligning with the “perceptual anchoring” phenomenon: humans instinctively rely on MFCC-dominated bands in noise, enabling MFCC-based models to mimic human resilience. MFCC maintained signal similarity assessment, while LPCC failed.

**Conclusions:** A DBN-based speech recognition model outperformed traditional methods (e.g., hidden Markov, BP neural networks) on the ArabicDigit dataset. Integration of behavioral markers (e.g., stress-induced pitch variability) allowed differentiation between technical errors and adaptive behaviors. Posterior probability deviations >20% correlated with cognitive overload indicators (e.g., erratic rhythm), offering dual diagnostic value for pronunciation and learning optimization. Incorporating SVM classifiers and intonation evaluation provides new perspectives. Future research could develop adaptive feedback systems that detect compensatory strategies and adjust difficulty dynamically, emulating expert tutoring interventions.

## PHHS25171: SELF-CARE MANAGEMENT OF HYPERTENSIVE ELDERLY CLIENTS IN SELECTED MEDICAL CARE FACILITY ON CHINA-BASIS FOR CREATION OF NURSING CARE MODEL

Huidong Ding^a^, Huidong Guo^a^, Huijie Guo^b^, Cunqiang Hou^c^, Qinqin Zhao^d^, Kefang Wang^e^

^*a*^ *Taishan Nursing Vocational College, Department of nursing, Taian 271000, China,* ^*b*^ *Shandong Second Military Special Care Hospital, Second ward, Taian 271000, China,* ^*c*^ *Tai’an 88 Hospital of Rongtong Group, Trauma Surgery, Taian 271000, China,* ^*d*^ *The Affiliated Taian City Central Hospital of Qingdao University, Geriatric Cognitive Medicine Ward, Taian 271000, China,* ^*e*^ *Shandong University, School of Nursing and Rehabilitation, Taian 271000, China.*

*HD and HG contributed equally to this research.*

**Objective:** The study aimed to develop a nursing care model for elderly hypertensive patients. It intended to provide healthcare staff with a model for educating clients on self - management, offer nursing educators information on comprehensive nursing care for elderly hypertensive clients, guide patients in improving self - management, and provide a reference for future nursing researchers.

**Subjects and Methods:** The research focused on hypertensive elderly clients in selected medical care facilities in China. A sequential explanatory mixed methodology was used. First, quantitative data were collected and analyzed. In the second phase, qualitative data related to the quantitative outcomes were gathered. This approach allowed for data integration at an appropriate stage, enabling a more comprehensive understanding of the research topic, as it could answer questions that single - method designs could not.

**Results:** A Pearson r test was used to examine the relationship between participants’ perceived behavioral lifestyle and their level of self - care efficacy. The results showed a low negative correlation between the two variables. Although the correlation was weak, further testing indicated that the relationship was significant, suggesting that the behavioral lifestyle has a negative impact on the self - care efficacy of hypertensive Chinese elderly.

**Conclusions:** Most participants were in late adulthood, with females being predominant and living in a selected healthcare facility. Generally, the participants could take care of themselves and were compliant with the treatment. There is a direct association between behavioral lifestyle and perceived self - care efficacy, where the former directly affects the latter. Additionally, support from family and significant others is crucial for elderly hypertensive clients in coping with the disease. This study provides valuable insights for the development of a nursing care model and for improving the self - management of elderly hypertensive patients.

**Acknowledgments**

This research was funded by the Taian Science and Technology Innovation Development Project (No:2023ZC582), Taian Science and Technology Bureau Project (No:2020NS194).

## PHHS25172: DUAL-CHANNEL PRICING POLICIES OF NEW ENERGY VEHICLE SUPPLY CHAIN CONSIDERING GOVERNMENT POLICY AND CONSUMERS PREFERENCES

Chao Feng^a^, Yunxia Niu^b^, Qiuyu Tang^a^, Fang Chen^c^

^*a*^ *School of Economics, Trade and Management, Xinjiang Institute of Technology, Aksu 843100, China,* ^*b*^ *Department of Mathematics, School of Science, Xinjiang Institute of Technology, Aksu 843100, China,* ^*c*^ *Department of Economics and Management, Zhejiang Ocean University, Zhoushan 316022, Zhejiang, China.*

**Background:** With rapid advancements in New Energy Vehicle (NEV) technologies and significant transformation in marketing and sales channels, China’s NEV market is undergoing a shift from policy-driven mechanisms toward a more market-oriented structure. During this crucial transition, consumer psychological health—including emotional regulation, anxiety related to adopting new technologies, and stress perception—has emerged as a vital factor shaping consumer purchasing behavior and influencing their preferences between traditional and emerging retail channels. However, current supply chain and pricing research predominantly focuses on economic and policy factors, leaving psychological considerations underexplored.

**Subjects and Methods:** This study investigates pricing strategies within a dual-channel NEV supply chain framework, comprising both manufacturers and retailers. The analysis explicitly integrates government interventions, such as subsidies and carbon taxes, consumer heterogeneity in terms of preference (psychological health dimensions) and environmental consciousness. A hotelling-based spatial competition model is adopted to capture consumer utility, explicitly embedding psychological components such as anxiety about vehicle range, charging convenience, and perceived financial stress related to NEV adoption. To reflect the strategic interactions among supply chain participants, a Stackelberg game theoretical approach is utilized, modeling hierarchical pricing decisions wherein manufacturers act as leaders setting wholesale prices, followed by retailers’ decisions on retail prices. Furthermore, this study explores profit-sharing mechanisms under strategic alliance scenarios within the supply chain, employing the Shapley value method to ensure equitable distribution of cooperative benefits.

**Results:** The analytical derivations and numerical simulations yield several critical insights. Firstly, expanding NEV charging infrastructure markedly alleviates consumer anxiety regarding driving range and battery charging concerns, significantly improving psychological comfort. Consequently, NEVs become substantially more competitive, potentially leading to the marginalization of traditional fuel vehicles (FVs) in the market. Secondly, increased consumer environmental awareness indeed enhances the demand for NEVs. However, heightened environmental consciousness may simultaneously prompt retailers to raise NEV prices, inadvertently exacerbating consumers’ financial anxiety and psychological stress associated with the higher upfront costs. Thus, findings suggest that an isolated emphasis on environmental promotion, without concurrent psychological support, could undermine overall market growth. Coordinated interventions that integrate economic incentives with psychological support measures are essential to maximize consumer acceptance and market stability.

**Conclusions:** This study provides novel contributions by incorporating consumer psychological health explicitly into NEV supply chain pricing strategies. It underscores emotional well-being and stress perception as critical determinants of consumer decisions, thus broadening the theoretical understanding of NEV consumer behavior. From a managerial perspective, findings offer actionable implications, advocating for comprehensive strategies that integrate psychological well-being considerations into channel optimization, pricing decisions, and policy design. Such integrative approaches not only enhance consumer satisfaction but also strengthen the overall market resilience and sustainability of China’s NEV industry.

**Acknowledgments**

This research was supported by the National Social Science Foundation of China (Grant No. 23BJY006) and the Natural Science Foundation of Xinjiang Province (Grant No. 2021D01B41).

## PHHS25173: RESILIENCE AMID DISRUPTION? REASSESSING THE COMPETITIVENESS OF CHINESE HIGH-END MANUFACTURING AND INVESTOR MENTAL HEALTH UNDER U.S. ENTITY LIST SANCTIONS: AN EVENT STUDY APPROACH

Qifeng Gao^a^, Chulok Ahn^b^

^*a*^ *Department of Convergence Technology Entrepreneurship, Kunsan National University, Gunsan 54150, Republic of Korea.* ^*b*^ *Department of Convergence Technology Entrepreneurship, Kunsan National University, Gunsan 54150, Republic of Korea.*

**Background:** This paper employs the event study method to examine seven Entity List incidents from 2018 to 2021, analyzing their impacts on 109 Chinese high-end manufacturing companies. Particular attention is given to how these external shocks influenced firm performance, investor sentiment, and psychological well-being over time. This study contributes to existing research in two main ways. First, it systematically evaluates the effects of Entity List incidents on stock prices, revealing that while individual events typically trigger significant short-term market impacts and negative investor sentiment, these effects gradually diminish. Second, it explores how companies respond at the micro-level through coping strategies in technological innovation, supply chain management, investor relations, and international cooperation. Together, these insights provide a comprehensive understanding of how Chinese high-end manufacturers adapt within global supply chains, mitigate market anxiety, and maintain investor psychological well-being.

**Subjects and Methods:** The study applies an event study approach to evaluate company performance before and after the Entity List incidents, investigating spillover effects within the industry and examining firm-level characteristics such as operational robustness, supply chain concentration, innovation capacity, and the psychological responses of investors. The analysis considers both financial outcomes and shifts in market sentiment and investor confidence.

**Results:** The findings reveal that early incidents significantly harmed company performance and triggered negative investor sentiment and heightened psychological stress among market participants. However, these adverse effects gradually diminished as firms and investors adapted to external shocks. By 2021, some incidents had even begun to produce positive financial effects and stabilize investor sentiment. The Entity List incidents also caused spillover effects, impacting investor psychology across the broader industry. Further analysis indicates that companies with robust operations, low supply chain concentration, and strong innovation capacity were better able to mitigate supply chain disruptions and sustain investor confidence.

**Conclusions:** Governments and companies should fully recognize both the financial and psychological impacts of Entity List incidents on investors. It is essential to implement appropriate countermeasures to stabilize market sentiment. Governments should enhance communication and collaboration with multiple stakeholders to reduce uncertainty, while companies should invest more in independent innovation, R&D, and investor relations strategies. Together, these efforts can strengthen industry development, promote market resilience, and support the psychological well-being of investors facing external risks.

## PHHS25174: THE AMPLIFICATION AND MITIGATION OF SOCIAL MEDIA’S IMPACT ON COLLEGE STUDENTS’ EMPLOYMENT ANXIETY

Xin Cui^a^, Ranran Xie^b^

^*a*^ *Huangshan University, Huangshan 245041, Anhui, China,* ^*b*^ *College of Management, Anhui Science and Technology University, Bengbu 233030, Anhui, China.*

**Background:** The widespread use of social media has led college students to increasingly rely on online platforms for employment information, career comparisons, and social interactions during their job search. However, competitive content, idealized career narratives, and algorithm-driven recommendations may intensify employment anxiety. The algorithmic recommendation mechanisms of social media, the “selective presentation” of peers’ career achievements, and the proliferation of “involution-style” job-seeking content may exacerbate individuals’ psychological stress. While existing research has primarily focused on the relationship between social media and mental health, the mechanisms through which it influences employment anxiety—via information environments, social comparison, and other pathways—remain underexplored.

**Subjects and Methods:** This study integrates Social Comparison Theory (Festinger) and Cognitive Appraisal Theory (Lazarus & Folkman), and based on survey data, constructs the “Algorithm-driven Anxiety and Mitigation Dual-path Model” (AAM-DM). This study employs surveys (N=483) and in-depth interviews (n=20) with college students across 12 national universities, integrating social comparison theory and stress cognition theory to examine how social media exacerbates employment anxiety and to explore mitigation strategies.

**Results:** Findings show that 73.2% of respondents experience heightened anxiety due to peer competition content on social media, particularly exaggerated success stories. Algorithm-driven recommendations further amplify these negative emotions (β = 0.32, p < 0.01), with individuals displaying lower self-efficacy being more susceptible to emotional distress. This suggests that social media not only triggers anxiety through content exposure but also intensifies it through personalized, algorithmic reinforcement.

**Conclusions:** Social media amplifies employment anxiety through mechanisms such as “information overload” and “distorted comparisons,” yet its instrumental use can serve as a resource for alleviating stress. It is recommended that universities collaborate with platforms to optimize algorithmic logic and reduce “anxiety-inducing” content. Meanwhile, universities can offer media literacy courses to cultivate students’ critical information-processing skills. Social media exacerbates employment anxiety due to information overload, social comparison, and algorithmic bias. Effective mitigation strategies include enhancing information literacy to better navigate digital content, promoting positive career narratives, and advocating for platform responsibility to reduce harmful content.

## PHHS25175: THE PARADIGM RECONSTRUCTION OF TEACHERS’ EMOTIONAL LABOR IN THE ERA OF ARTIFICIAL INTELLIGENCE

Fang Liu ^a^

^*a*^ *Anhui Sanlian University, Hefei, Anhui 230601, China.*

**Background:** Both Chinese and Western cultural traditions emphasize the important role of emotions in teaching activities. American sociologist Hochschild, from the perspective of social psychology, defined the behavior of actively managing one’s own emotions in modern social work to meet the requirements of the organization or profession as “emotional labor”. In the era of artificial intelligence, teachers also need to invest emotions in their teaching work, that is, engage in emotional labor. Artificial intelligence has reconstructed the knowledge dissemination model in the traditional educational ecosystem and poses many challenges to the practical forms of teachers’ emotional labor.

**Subjects and Methods:** Taking the “Theory of Technological Mediation” as the starting point, this study follows the logical thinking of “theoretical foundation laying—realistic challenges—paradigm reconstruction”, integrates interdisciplinary theories such as philosophy, pedagogy, and social psychology, and focuses on exploring effective paths to address the challenges and dilemmas faced by teachers in their teaching work in the era of artificial intelligence. It aims to reconstruct the theoretical paradigm of teachers’ emotional labor in the AI era, so as to promote the return to the essence of education and the reconstruction of teachers’ dominant position.

**Results:** Through interdisciplinary theoretical analysis, it is found that in the era of artificial intelligence, teachers’ emotional labor faces challenges such as the contradiction between the authenticity of emotions and the uncertainty of interaction, the risk of the separation between the body and emotions, and the conflict between algorithmic justice and the warmth of human nature. These challenges will directly lead to the continuous depletion of teachers’ individual psychological and emotional resources, which is specifically manifested as the dissolution of the sense of professional meaning, cognitive overload and decision-making fatigue, anxiety and self-doubt, and the “de-emotionalization” of teacher-student relationships, etc.

**Conclusions:** Artificial intelligence should enhance rather than weaken human professionalism and emotional connection. It is necessary to construct a new paradigm of teachers’ emotional labor in the era of intelligent education featuring “artificial intelligence + professional capital + emotional labor” by regulating emotions to reconstruct the human capital of intelligent education, anchoring the rules of feeling to adjust the social capital of intelligent education, and conducting deep acting to integrate the decision-making capital of intelligent education. We should adhere to the educational consciousness at the ontological level in the application of technology to help the return of the essence of education and the reconstruction of teachers’ subjectivity.

**Acknowledgments**

This work was supported by a project grant from the Anhui Provincial Social Science Innovation and Development Research Project titled “Research on the Cultivation of College Students’ National Security Awareness in the New Era—Based on the Investigation of Five Universities in Anhui Province” (Project No.2021KY010); the Major Humanities and Social Sciences Project of the Anhui Provincial Colleges and Universities Scientific Research Project in 2024 titled “Research on the Approaches of National Security Education in Primary, Middle and Higher Education—Taking Hefei City as an Example” (Project No.2024AH040313); and the Provincial Quality Engineering Project of Anhui Province’s Higher Education Institutions in 2023 titled “Offline First-Class Course ‘Fundamentals of Marxism’” (Project No.2023xxkc385).

## PHHS25176: RESEARCH ON THE OPTIMIZATION OF GUANGZHOU’S ELDERLY CARE SERVICE SYSTEM BASED ON “PUBLIC WELFARE TIME”: MATHEMATICAL MODEL CONSTRUCTION FOR PROMOTING A HEALTHY AGING MUTUAL AID MODEL

Weixin Huang^a^, Jinlan Guan^b^, Ximing Wu^c^

^*a*^ *Guangdong Vocational College of Foreign Languages and Arts, Guangzhou, China,* ^*b*^ *Guangdong Agriculture Industry Business Polytechnic College, Guangzhou, China,* ^*c*^ *Guangdong Food and Drug Vocational College, Guangzhou, China.*

**Background:** As the accelerating population aging has become a major public health challenge in China, this study examines Guangzhou’s innovative “Public Welfare Time” mutual-support elderly care model. The research proposes recommendations including establishing an efficient technological platform, innovating publicity and incentive mechanisms, and optimizing service matching. These measures aim to address healthy aging by improving service quality and efficiency. The findings provide both theoretical foundations and practical references for optimizing and upgrading Guangzhou’s mutual-support elderly care system to promote healthy aging.

**Subjects and Methods:** This study employed a mixed-methods approach: 1. A questionnaire survey was conducted among 816 Guangzhou residents (with 781 valid responses), along with in-depth interviews involving 30 stakeholders (including elderly individuals, caregivers, and community healthcare workers). The data were analyzed to assess public health literacy, awareness of mutual-support elderly care services, willingness to participate, and related needs. 2. A mathematical model was developed, integrating the health-related demands of the elderly (demand side) and the volunteer-driven service supply (supply side), to systematically evaluate the operational status of Guangzhou’s “Public Welfare Time” model. The study specifically examined structural supply-demand imbalances and proposed actionable optimization strategies.

**Results:** Key findings include: 1. Severe supply-demand imbalance in healthy aging services: The survey shows that 76.18% of respondents are willing to volunteer, but only 37.77% actually participate. Volunteers primarily serve 1–3 times annually (39.3%), while the elderly expect monthly (28.2%) or weekly (25.78%) services, highlighting significant gaps. Moreover, healthcare services (77.42% demand) require professional skills, yet only 41.67% of volunteers are qualified, leading to structural shortages. 2. Insufficient service quality and specialization: Current volunteers are predominantly students and healthy individuals lacking medical or nursing expertise. Companionship (72.84%) and recreational activities (78.4%) dominate, while daily care (53.4%) and medical support (41.67%) are limited. Some elderly individuals, dissatisfied with service quality, tend to hire professional caregivers rather than rely on volunteer services. 3. Lack of cross-regional point redemption mechanism: 55.83% of respondents desired intercity point transfers, but the current platform restricted usage to local areas. Low point storage caps (1,500 points) and limited redemption options (primarily services) reduced the model’s appeal. 4. Low public awareness and participation motivation. 43.28% had only superficial knowledge of “Public Welfare Time”, while 41.74% were largely unfamiliar. Outreach relied heavily on community events (27.4%) and online platforms (38%), lacking systematic promotion. Incentives were monotonous: 87.04% of volunteers valued self-fulfillment, but material rewards (22.84%) were insufficient to attract broader participation.

**Conclusions:** Four evidence-based optimization strategies are proposed: 1. Establishing a cross-regional blockchain point platform; 2. Optimizing a precision supply-demand matching system; 3. Innovating diversified incentive mechanisms; 4. Expanding a full lifecycle service ecosystem. The mathematical model serves as a valuable tool for policymakers in mutual-aid elderly care planning, with potential for replication in other aging societies.

## PHHS25177: INFLUENCING FACTORS AND INTERVENTION STRATEGIES OF COLLEGE STUDENTS’ MENTAL HEALTH

Fang Wang^a^, Shangxing Yang^a^

^*a*^ *Wuhan Railway Vocational College of Technology, Wuhan, China.*

**Background:** With the intensification of social competition and changes in lifestyles, the mental health problems of college students are becoming more and more prominent, and have become an important challenge to the education and management of colleges and universities. College students are at a critical stage of life development, and their mental health status not only affects the quality of individual learning and life, but also relates to the harmony and progress of the whole society. This study aims to explore the influencing factors of college students’ mental health and propose corresponding intervention countermeasures to reduce the occurrence of college students’ mental health problems and promote their overall development.

**Subjects and Methods:** College students’ mental health problems were investigated through literature review and empirical research. The questionnaire survey method was used to collect data on the mental health status of college students from different colleges and universities and different grades, and statistical analysis methods were used to explore the main factors affecting college students’ mental health.

**Results:** It was found that the factors affecting college students’ mental health mainly include personal factors (e.g., personality traits, coping styles, self-perception, etc.), family factors (e.g., family structure, parenting styles, family economic status, etc.), school factors (e.g., study pressure, interpersonal relationships, campus culture, etc.), and social factors (e.g., social competition, employment pressure, network environment, etc.). These factors are intertwined and work together to influence the mental health status of college students.

**Conclusions:** The results of this study indicate that the solution of college students’ mental health problems requires multi-party collaboration and comprehensive interventions. By strengthening mental health education, improving the mental health service system, optimizing the family and social environment and other countermeasures, we can effectively reduce the incidence of mental illness and improve the mental health of college students. This study provides theoretical basis and practical guidance for colleges and universities to formulate mental health education policies and carry out mental health service work, which is of great significance to promote the overall development of college students.

## PHHS25178: RESEARCH ON COST MANAGEMENT OPTIMIZATION AND INNOVATION OF EXPRESS DELIVERY ENTERPRISES UNDER THE BACKGROUND OF THE FOURTH INDUSTRIAL REVOLUTION BASED ON PSYCHOLOGICAL HEALTH

Shengyu Tian ^a^

^*a*^ *Harbin Finance University, Harbin, Heilongjiang, China.*

**Background:** Economic development drives technological progress, and the Fourth Industrial Revolution profoundly reshapes the express delivery industry. The Fourth Industrial Revolution has made express delivery more intelligent, efficient, and personalized, becoming the core driving force of modern supply chains. However, while developing, there are also some problems, including external issues and internal problems of the enterprise. External factors such as poor industry management and regulation, price wars and vicious competition, and uneven urban-rural and regional development; Internal service quality issues, employee rights and professional dilemmas, and shortcomings in technology and safety management. External issues are uncontrollable factors for enterprises, while internal issues ultimately stem from cost issues. Psychology can optimize employee motivation, reduce loss, improve efficiency and reduce error rate through behavior analysis, and improve customer experience in the cost management of express delivery industry, so as to control costs indirectly. This article attempts to start from the perspective of internal cost management in the express delivery industry, fully utilize the achievements of technological innovation in the fourth industrial revolution, provide solutions, and also take into account the group mental health of the express delivery industry.

**Subjects and Methods:** This article takes the express delivery industry as the research object, and analyzes the problems in cost management in the express delivery industry from the perspective of social and psychological health combined with cost management methods. The literature review method was applied to explain the current cost management methods in the express delivery industry. Applying behavioral science research methods to study the behavioral motivations of employed individuals in different contexts.

**Results:** Cost management in the express delivery industry should be a comprehensive process of cost management. The application of artificial intelligence can better understand human thoughts and behaviors, replace delivery personnel in high-risk or monotonous, remote areas, and provide more convenient and efficient services. Mental health can improve the efficiency of express delivery employees, reduce errors and turnover rate, reduce labor and operating costs, and improve service quality and optimize enterprise benefits. The optimization of human centered business processes and innovation in cost management methods have a significant impact on enterprise costs, further affecting the company’s profit and loss, and thus impacting its industry competitiveness.

**Conclusions:** Enterprises should actively participate in the innovation of the Fourth Industrial Revolution, adopt different cost management methods based on the scale of express delivery enterprises, the scope and characteristics of express delivery services, pay attention to mental health, and improve internal competitiveness.

**Acknowledgements**

This work was supported by a project grant from 2024 Heilongjiang University Student Innovation and Entrepreneurship Training Program Project (University-level project) “Intelligent Financial Outsourcing”(Grant No. X202410245023).

## PHHS25179: ANALYSIS OF MUSIC THERAPY AND PSYCHOLOGICAL STRESS RELIEF FOR MIDDLE SCHOOL STUDENTS

Zekun Shao^a^, Yingfei Yin^b^

^*a*^ *Junior High School of Xidian Town, Ningbo, China,* ^*b*^ *Teaching and Research Office of Ninghai County, Ningbo, China.*

**Background:** This study aims to deeply explore the actual effect of music therapy in relieving the psychological tension of middle school students. Through carefully designed experimental programs and rigorous data collection and analysis, we aim to verify whether music therapy can effectively reduce the learning pressure and psychological burden of middle school students, and provide a new intervention means for the mental health of middle school students. Statistical analysis of experimental data will provide us with strong evidence support.

**Subjects and Methods:** The experimental group underwent a four-week music intervention, while the control group did not receive any intervention. The Self Rating Anxiety Scale (SAS) and Mood State Scale (PANAS) were used to assess psychological tension before and after the intervention. Physiological measures, including heart rate and skin conductance response, were also recorded.

**Results:** After the four-week music therapy, the experimental group showed a significant reduction in anxiety, with SAS scores decreasing from 65.8 to 45.3 and PANAS scores dropping from 60.2 to 43.1, while the control group showed no significant changes. Physiological data revealed that the experimental group’s heart rate decreased from 80 beats per minute to 74 beats per minute, and skin conductance response values dropped from 0.24 microsiemens to 0.19 microsiemens. Statistical analysis (p<0.05) confirmed that both light and classical music significantly reduced anxiety and enhanced emotional stability. Additionally, the intervention improved sleep quality, increasing sleep time by 45 minutes and reducing sleep interruptions by two occurrences. Academic performance also improved, with math and English scores increasing by 12% and 9%, respectively.

**Conclusions:** Studies have shown that personalized music therapy can effectively relieve students’ psychological tension, improve emotional stability, and promote the improvement of academic performance, especially for students under high learning pressure. Through customized music, students can relax in a harmonious atmosphere, improve learning efficiency, and thus better cope with academic challenges.

## PHHS25180: SMART HEALTHCARE FOR LIFELONG WELL-BEING: EVALUATING THE ROLE OF ARTIFICIAL INTELLIGENCE AND BIG DATA IN NATIONAL HEALTH STRATEGIES

Xiaojing Hou^a^, Huan Liu^a^

^*a*^ *Liaoning University, Shenyang, 110036, China.*

**Background:** Amid the accelerating global trend of population aging and the rising prevalence of chronic diseases, public health service systems are increasingly challenged by inadequate coverage, low efficiency, and insufficient equity. In response, China’s “Healthy China 2030” strategy has explicitly set the goal of achieving universal and lifelong health, highlighting the urgent need to empower healthcare systems through technological innovation. Artificial intelligence (AI) and big data, regarded as emerging drivers of new quality productive forces, have shown growing potential within smart healthcare initiatives, offering new pathways to achieve national health objectives.

**Subjects and Methods:** This study employed a comprehensive methodological approach combining policy document analysis, system model construction, case study synthesis, and literature review. Key policy documents issued by the National Health Commission, including the “14th Five-Year Plan for Health” and the “Healthy China 2030 Outline,” were analyzed using content analysis to extract the logical framework connecting policies and technological applications. A data flow diagram (DFD) model of the “Smart Health Service System” was developed to illustrate the system architecture and operational mechanisms. Representative smart healthcare cases, such as the “Health Brain” platform in Zhejiang Province and Ping An Good Doctor, were selected for in-depth analysis. Additionally, research trends concerning AI and big data applications in telemedicine, chronic disease management, and smart hospitals were synthesized from domestic and international literature.

**Results:** The constructed smart health service system model identifies four major stakeholders: individuals, healthcare institutions, regulatory authorities, and technology platforms, interconnected through a closed-loop data flow encompassing collection, processing, analysis, and feedback. AI and big data were found to play critical roles across various healthcare processes, including disease prediction, diagnostic support, personalized health management, and medical resource allocation. Although local initiatives have made notable progress under national policy guidance, challenges persist, particularly regarding data silos, fragmented platforms, underdeveloped privacy standards, and interoperability issues.

**Conclusions:** AI and big data are evolving from supporting tools to integral components at both the systemic and strategic levels in healthcare transformation. Achieving the goals of universal and lifelong health requires the coordinated advancement of national policies, technological capabilities, and public health literacy. This study provides a preliminary framework and practical references for the systematic construction and policy optimization of smart health services.

## PHHS25181: THE IMPACT OF FARMERS’ INTEGRATION OF LAND RESOURCES IN RURAL AREAS ON THE QUALITY OF MOBILE EMPLOYMENT: RESEARCH ON THE MEDIATING ROLE OF MENTAL HEALTH

Jing Feng^a^, Dongyang Sun^b^, Yan Feng^c^

^*a*^ *Luoyang Polytechnic, Luoyang, China,* ^*b*^ *Henan Finance University, Zhengzhou, China,* ^*c*^ *Luoyang Institute of Science and Technology, Luoyang, China.*

**Background:** In the development environment of new urbanization and urban-rural integration, it is particularly important to explore the inherent connection between agricultural land resources and labor factors, which can drive high-quality employment of rural labor, accelerate rural revitalization, and promote common prosperity.

**Subjects and Methods:** The article constructs a logical framework for the impact relationship between “active integration of land resources in rural areas by farmers and the quality of rural labor mobility”, and uses the zero inflation ordered logit model (ZIOL) to solve the practical problem of data containing a large number of zero values. It explores the impact and transmission mechanism of farmers’ active integration of land resources in rural areas on the employment quality of migrant workers through two methods: rural collective land leasing and transfer of homestead land use rights. This article explores the influencing mechanisms through two pathways: “mental health level” related to social networks and “stress resistance ability” related to psychological adaptability.

**Results:** (1) Farmers actively integrate land resources in rural areas to promote the improvement of employment quality for migrant workers; (2) The positive effect of farmers actively integrating land resources in rural areas on the quality of labor mobility is more significant in samples with lower levels of land capital and financial capital, as well as higher levels of human capital; (3) Strengthening “mental health level” related to social networks and accumulating “stress resistance ability” related to psychological adaptability. are two transmission paths for farmers to actively integrate land resources in rural areas and promote the improvement of rural labor mobility quality.

**Conclusions:** To this end, it is necessary to steadily and cautiously promote the reform of the rural land system, stimulate the vitality of rural resource elements, identify the wishes of farmers and their social resources. In order to adapt to the reality of large-scale agricultural operation and differentiated integration of rural land resources, it is necessary to timely pay attention to the psychological health of migrant workers, actively carry out employment guidance on psychological adaptability education, improve the quality of migrant employment for rural residents, and promote the deep integration and development of rural land and labor resources.

**Acknowledgements**

This work was supported by a project grant from China National Social Science Foundation (Grant No. 19BJY125). At the same time, it is worth noting that the raw data for this article mainly comes from China Family Panel Studies, CFPS. CFPS is implemented by the Institute of Social Science Survey (ISSS) at Peking University. The project adopts computer-aided survey technology to conduct visits to meet diverse design needs, improve access efficiency, and ensure data quality. Thank you for sharing the data from the Institute of Social Science Survey at Peking University.

## PHHS25182: THE IMPACT MECHANISM OF ACHIEVEMENT MOTIVATION ON COLLEGE STUDENTS’ PROFESSIONAL IDENTITY: THE MEDIATING ROLE OF ACADEMIC EMOTION

Shasha Ye^a^, Yangkai Wu^a^, Yingdong Zhu^a^, Shujuan Feng^a^, Guolai Wu^b^

^*a*^ *Zhejiang Shuren University, Hangzhou, 310015, China,* ^*b*^ *Tianjin Normal University, Tianjin, China.*

**Objective:** With the development of higher education, college students’ learning behaviors have become an important topic in educational research and practice. Professional identity, as a significant psychological variable influencing college students’ learning behaviors, has not received sufficient attention in the learning process. Moreover, achievement motivation and academic emotion, as two major factors affecting learning behaviors, how they influence college students’ professional identity and what roles they play remain hot issues in current educational psychology research. This study aims to explore the impact of achievement motivation on college students’ professional identity and further analyze the mediating role of academic emotion in this influence process, in order to deeply understand the influencing factors of college students’ professional identity and provide theoretical basis and practical guidance for enhancing college students’ professional identity level.

**Subjects and Methods:** This study employed the questionnaire survey method and combined it with the structural equation model (SEM) to analyze the relevant data. The research subjects were college students from multiple universities, with a sample size of 435. Data collection was conducted through self-administered questionnaires, which were divided into three aspects: professional identity, achievement motivation, and academic emotion. The specific measurement tools included the “Professional Identity Scale”, the “Achievement Motivation Scale”, and the “Academic Emotion Scale”. The reliability of each scale and its dimensions was tested, showing that they all had good reliability. The study used SPSS and AMOS software for data analysis, mainly through descriptive analysis, correlation analysis, and structural equation model analysis to explore the relationship between achievement motivation, academic emotion, and professional identity.

**Results:** The results of the study indicated that: achievement motivation significantly and positively predicted professional identity, i.e., the higher the achievement motivation, the stronger the professional identity of the students; academic emotion played a mediating role between achievement motivation and professional identity. Specifically, positive academic emotions (e.g., interest, pleasure) enhanced the positive effect of achievement motivation on professional identity, while negative academic emotions (e.g., anxiety, stress) weakened this effect. The mediating effect of academic emotion was verified through structural equation modelling (SEM) analysis, further refining the intrinsic mechanism by which achievement motivation affects professional identity.

**Conclusions:** This study reveals the complex path of influence of achievement motivation on professional identity and highlights the important role played by academic emotion in this process. The results of the study provide useful insights for higher education and suggest that educators should focus on regulating students’ academic emotion while enhancing their achievement motivation in order to comprehensively promote students’ professional identity.

## PHHS25183: RESEARCH ON DIGITAL RESTORATION TECHNOLOGY OF ANCIENT SHU BROCADE FROM THE PERSPECTIVE OF CULTURAL HEALING - - COLLABORATIVE INNOVATION BASED ON PSYCHOLOGICAL NEEDS SATISFACTION AND DIGITAL HUMANITIES

Yulan Zhou^a^

^*a*^ *Academy of Fine Arts, Sichuan Minzu College, Kangding, Sichuan626001, China.*

**Objective:** As an important cultural symbol of the Chinese nation, Shu brocade carries the historical memory and emotional identity of the ancestors of Bashu, and its inheritance dilemma reflects the fault of the contemporary public’s psychological needs for traditional culture.

**Subjects and Methods:** Based on the perspective of psychological needs in cultural heritage protection, this study combines iconography theory and field investigation method, and reveals the multi-dimensional psychological demands of the public on cultural memory restoration, emotional resonance awakening and identity reconstruction in the process of Shu brocade pattern restoration through the field investigation of Chengdu Shu brocade embroidery museum.

**Results:** The study found that the dynamic restoration enabled by digital technology can effectively meet the cultural belonging needs of 89 % of the respondents. The three-dimensional reconstruction technology enables 92 % of the audience to have an immersive emotional experience, and the virtual reality scene significantly enhances the national cultural identity (p < 0.01).This study innovatively proposes to incorporate psychological needs assessment into the digital ISO standard system of cultural heritage, and construct a four-dimensional psychological satisfaction path of ‘ digital restoration-scene perception-emotional resonance-community consciousness ‘, so as to provide a solution that takes into account both technical rationality and humanistic care for the protection of intangible cultural heritage. By meeting the deep psychological needs in the construction of the Chinese nation community, the cognitive basis for the inheritance of national cultural memory is cast.

**Conclusion:** Digital empowers the collective memory of Bashu land to build a strong sense of the Chinese nation community. This project believes that the digital empowerment of ancient Shu brocade restoration and display, the ancient Shu brocade image dynamic, field integrity, so that people in the perception of the scene will be personal emotions, memory and imagination, is conducive to the collective memory of Bashu, cast a strong sense of the Chinese nation community. Through digital restoration, three-dimensional reconstruction, virtual reality and other technologies, it helps the sustainable preservation, public display and value dissemination of ancient Shu brocade. The innovative use of clothing culture gene theory in the study of ancient Shu brocade has enriched the perspective and method of ancient Shu brocade research. Through in-depth study of the national culture, formal vision and geographical environment cultural genes in the ancient Shu brocade, this paper analyzes the historical facts of the communication and integration of Bashu reflected in it, and forms a comprehensive reflection of the ‘ gene map ‘, which provides a theoretical basis for the protection of this national intangible cultural heritage, national geographical and cultural symbols, and Sichuan ‘s unique traditional folk arts and crafts cultural heritage, and inherits its traditional culture and spirit. Incorporating psychological needs assessment into the ISO standard system for digitization of cultural heritage.

**Acknowledgements**

This work was supported by Key project of digital protection and inheritance of intangible cultural heritage of weaving and embroidery technology in key laboratory of Sichuan Provincial Department of Culture and Tourism, ‘technical research on collection and restoration design of digital resources of ancient Shu brocade from the perspective of ‘culture + technology’‘ (2024SYSZD05).

## PHHS25184: RESEARCH ON THE TEMPORAL AND SPATIAL DISTRIBUTION CHARACTERISTICS AND INFLUENCING FACTORS OF HAIKOU TOURISM NETWORK ATTENTION AND TOURIST PSYCHOLOGICAL NEEDS: AN ANALYSIS BASED ON BAIDU INDEX

Wenyan Chen^a^, Ying Chen^a^, Qitao Hong^b^

^*a*^ *Tourism College, Hainan Vocational University, Haikou 570216, China,* ^*b*^ *Tourism College, Hainan Normal University, Haikou 571158, China.*

**Background:** Tourism network attention and tourist psychology are complementary and mutually influence. Tourism network attention stimulate the motivation of tourists to travel with its massive information. Especially to relieve pressure and meet social and other psychological needs. It can help tourists make the travel planning, and make tourists look forward to traveling. This expectation helps to improve the mental health of tourists. The preferences of tourists and their psychological demands to escape daily anxiety can affect their network search behavior, and the online reviews and public praise of tourists after their trip can in turn affect their network attention. They promote the development of the tourism industry together.

**Subjects and Methods:** In this context, the paper based on the Baidu index platform, taking Haikou city as the research object, collecting the network attention data of 31 provincial administrative region (except Hong Kong, Macao and Taiwan) in China during the five years from 2017 to 2021, and paying attention to the changes of tourists’ psychological needs and their impact on mental health. To analyze the temporal and spatial distribution characteristics and influencing factors of Haikou tourism network attention.

**Results:** The research results show that: 1. In terms of time distribution, Haikou’s network attention shows a declining trend year by year. Before the epidemic, the change trend of monthly network attention was similar, showing a more obvious “single peak” trend, the lowest values are mostly concentrated in June, the highest values are mostly concentrated in February. This may be related to the psychological needs of tourists to avoid the cold in winter and the psychological anxiety caused by the decline of tourism willingness after the festival. 2. In terms of spatial distribution, there are significant differences in the network attention of various provinces to Haikou. The region with the highest level of attention is Hainan province. Secondly, in the areas with close spatial distance and developed economy, residents tend to have stronger leisure consumption psychological needs and tourism consumption ability. It’s overall spatial distribution shows a trend of increasing concentration year by year. It conforms to the law of decreasing distance, that is, the closer the distance to Haikou, the higher the network attention. 3. In terms of the influencing factors, climate comfort and leisure time have a great influence on the time distribution of network attention. Because good climate conditions can effectively relieve the psychological pressure of tourists. Spatial distance, economic development level, population size and the intensity of economic connection between the two places have a great influence on the spatial distribution of network attention. Residents in economically developed areas pay more attention to maintaining mental health through tourism.

**Conclusions:** Finally, according to the research results, the problems in the development of tourism in Haikou are found, and the optimization countermeasures are put forward in order to meet the deep psychological needs and relieve the psychological anxiety of tourists. So as to promote the sustainable and healthy development of Haikou tourism.

**Acknowledgments**

This work was supported by a project grant from Haikou Academy of Social Sciences “Research on the Impact of TikTok Short-video Dissemination on the Tourism Destination Image of Haikou and Tourists’ Behavioral Intentions” (Grant No.2024-ZZKT-12).

## PHHS25185: ANALYSIS OF THE HEALTH IMPLICATIONS OF ZHUANG TRADITIONAL RAMMED EARTH HOUSES FROM THE PERSPECTIVE OF PERCEPTION PSYCHOLOGY

Lin Ma^a^, Wanqian Chen^a^

^*a*^ *School of Design, Hezhou University, Hezhou, Guangxi, China.*

**Background:** In the field of architecture, traditional building methods using earth as a primary construction material are among the most iconic symbols of humanity’s transition from primitive to civilized stages. The development of rammed earth construction highlights the immense potential of soil in creating human spaces. From the perspective of perception psychology, the traditional rammed earth houses in the Zhuang people’s settlements in Guangxi are facing the risk of disappearing due to the advancement of urbanization and modernization. The sustainability of Zhuang rammed earth houses and the healthy lifestyles of their inhabitants are at risk, with their psychological and physical health under threat.

**Subjects and Methods:** This study focuses on the Zhuang rammed earth houses in Guming Village, Guangxi, and analyzes the health implications of these houses from the perspective of perception psychology. A field study was conducted on the appearance of the houses, building materials, spatial layout, spatial functions, construction techniques, and living customs in Guming Village. The study explores how spatial perception, visual perception, auditory perception, tactile perception, and emotional perception affect the psychological and physical health of the residents. The theoretical framework and methods allow for an in-depth analysis of the residents’ perceptual mechanisms and psychological needs, enabling the planning of Zhuang rammed earth houses to be both scientific and human-centered. This approach can enhance the overall living experience and emotional resonance of the residents.

**Results:** Zhuang rammed earth houses contain an environment that embodies the poetic dwelling of the Zhuang people. Living in these houses allows the Zhuang people to experience their customs and cultural roots. This experience gradually alleviates their inner fatigue and anxiety, leading to a sense of satisfaction and belonging. The traces of time and deep emotional connections embedded in these houses become a sanctuary and support for the souls of the Zhuang people. Different spatial forms of the rammed earth houses correspond to different psychological health needs.

**Conclusions:** From the perspective of perception psychology, Zhuang rammed earth houses have a positive impact on the health of the residents. They contribute to both the psychological and physical well-being of the Zhuang people, becoming a source of identity and cultural pride for the local residents. This contributes to an overall improvement in residents’ mental health and provides them with a sense of security and belonging. Especially in the field of medicine, rammed earth houses play a positive role in promoting psychological rehabilitation. The lifestyle associated with living in these houses can gradually help regulate the psychological states of individuals suffering from depression and anxiety, alleviate their psychological stress, and activate the body’s own psychological repair mechanisms, creating favorable conditions for psychological rehabilitation.

**Acknowledgments**

2021 Guangxi Philosophy and Social Science Planning Research Project: Research on the Protection and Inheritance of Traditional Earth Houses of Ethnic Minorities in Guangxi (Project Number: 21FMZ038).

## PHHS25186: WHAT KIND OF CHINESE ENTERPRISE TEND TO ESTABLISH ENTERPRISE ANNUITIES: ENTERPRISE COMPETENCE OR EMPLOYEE PSYCHOLOGICAL NEEDS?

Jiaxu Chen^a^, Li Zhang^b^, Wei Shi^c^

^*a*^ *Law School, Southwest Petroleum University, Chengdu, Sichuan 610500, China,* ^*b*^ *Key Laboratory of Energy Security and Low-carbon Development, Chengdu, Sichuan 610500, China,* ^*c*^ *University Hospital, Southwest Petroleum University, Chengdu, Sichuan 610500, China.*

**Background:** The development of enterprise pensions in China has been sluggish. To address the challenges posed by an aging population, the Chinese Government aims to expand enterprise pension coverage significantly. However, the key determinants influencing the adoption of enterprise annuities remain unclear, and need to be analysed in terms of the capacity of enterprises and the psychological needs of employees.

**Subjects and Methods:** To identify enterprises more likely to establish enterprise annuities, data from 5,088 Chinese listed enterprises were collected. Relevant features such as basic enterprise status, enterprise capacity, and employees’ psychological needs were selected. A convolutional neural network prediction model, based on LeNet-5, was developed and subjected to multiple rounds of training to pinpoint the features of enterprises that have implemented enterprise annuities.

**Results:** The constructed convolutional neural network prediction model, incorporating factors influencing enterprise annuities, demonstrated high performance, with accuracy rates exceeding 90% for both the training and test sets. Analysis of typical enterprise features revealed that the nature of the enterprise and the percentage of shares owned by management are crucial factors influencing the establishment of enterprise annuities. In state-owned enterprises, employees’ psychological needs play a significant role in this decision-making process, whereas in private enterprises, the decision is more contingent upon the enterprise’s capacity.

**Conclusions:** The constructed convolutional neural network prediction model, incorporating factors influencing enterprise annuities, demonstrated high performance, with accuracy rates exceeding 90% for both the training and test sets. Analysis of typical enterprise features revealed that the nature of the enterprise and the percentage of shares owned by management are crucial factors influencing the establishment of enterprise annuities. In state-owned enterprises, employees’ psychological needs play a significant role in this decision-making process, whereas in private enterprises, the decision is more contingent upon the enterprise’s capacity.

**Acknowledgments**

This work was supported by a project grant from The National Social Science Fund of China (Grant No. 21XJY008).

## PHHS25187: HORTICULTURAL THERAPY PROGRAM FOR SUBJECTIVE WELL-BEING OF UNIVERSITY STUDENTS: EVIDENCE FROM SENSE OF MEANING, MOOD AND SATISFACTION WITH LIFE

Haixing Wang^a,b^, Maria L Bringas Vega^b^, Chenyan Yue^b^, Mengsha Qi^b^, Ya Chen^c^, Ying Hong^a^, Pedro Antonio Valdes-Sosa^b^

^*a*^ *Mental Health Education Center, University of Electronic Science and Technology of China, Chengdu, Sichuan 611731, China,* ^*b*^ *The Clinical Hospital of Chengdu Brain Science Institute, MOE Key Lab for Neuroinformation, University of Electronic Science and Technology of China, Chengdu, Sichuan 611731, China,* ^*c*^ *School of Life Science and Technology, Mental Health Education Center, University of Electronic Science and Technology of China, Chengdu, Sichuan 611731, China.*

**Background:** This study aims to evaluate the effects of horticultural therapy activities on improving the sense of well-being among college students, involving multiple indicators such as sense of the meaning of life, emotional balance, positive emotions, and satisfaction with life.

**Subjects and Methods:** A total of 112 undergraduate students from a university campus were recruited and randomly assigned to the experimental group and the control group. The experimental group participated in horticultural therapy interventions one session a week, 90 minutes per session, for a total of eight weeks. The control group engaged in free activities in the same meeting room during the first eight weeks. After the intervention, the experimental group were arranged to participate in the same horticultural therapy activities. Participants’ subjective experiences were assessed using psychological scales such as “the sense of meaning of life scale” and the “subjective sense of well-being scale”. Facial expression changes were also detected and analyzed by computer-based facial expression analysis technology, in order to evaluate changes in participants’ psychological experiences.

**Results:** The results showed no significant differences demographic variables between the groups. Prior to the intervention, there were no significant differences between the experimental and control groups on several indicators related to life well-being. After the intervention, the experimental group showed apparently higher scores in the sense of meaning of life (0.017), overall emotional index (0.044), and satisfaction with life (0.046) compared to the control group. Additionally, in the post-test stage, the experimental group showed apparently higher scores in the sense of meaning of life (0.02), emotional balance (0.007), and positive emotions (0.019) compared to the pre-test stage, while the control group’s pre-test and post-test results showed no significant differences. Chi-square statistic test were applied to the frequency of facial expression detection in video of a single horticultural therapy activity, which showed significant differences in the distribution of seven facial expressions between the experimental group and the control group. The herbal tea learning and flower arrangement and activities were where the “happy” facial expression most frequently occurred. As the horticultural therapy activities progressed, there was a trend toward increased positive expressions.

**Conclusions:** For college students, horticultural therapy activities effectively enhance their satisfaction with life, expand their sense of meaning of life, increase their positive emotions, reduce their negative emotions, and promote their emotional balance. These activities collectively contribute to improving students’ subjective sense of well-being from the perspectives of sense of meaning of life, emotions, and satisfaction with life. Therefore, horticultural therapy is an effective approach for psychological health education among college students, and is applicable for promotion and application in university campuses.

**Acknowledgments**

This study was supported by the Sichuan Psychological Association 2024 Research Planning Project, project approval No.: SCSXLXH202403004.

## PHHS25188: RESEARCH ON THE SPATIOTEMPORAL DISTRIBUTION CHARACTERISTICS AND INFLUENCING FACTORS OF MINERAL ENERGY DEVELOPMENT AND UTILIZATION EFFICIENCY IN SICHUAN PROVINCE FROM THE PERSPECTIVE OF MENTAL HEALTH

Xudong Zhou^a,b^, Jun Luo^c^, Yuqin Li^a^, Chao Yang^a^

^*a*^ *Luzhou Vocational and Technical College, Luzhou 646000, Sichuan, China,* ^*b*^ *Key Laboratory of Microbial Application and Monitoring Technology for Brewing, Luzhou 646000, Sichuan, China,* ^*c*^ *Luzhou Ecological Environment Monitoring Center Station in Sichuan Province, Luzhou 646000, Sichuan, China.*

**Background:** The energy industry plays a pivotal role in the development of the “5 + 1” modern industrial system in Sichuan Province. Therefore, it is necessary to conduct research on the efficiency and impact mechanism of energy and mineral development and utilization in Sichuan Province. The mental health of practitioners plays an indispensable role in improving the efficiency of mineral resource development and utilization, and it is also an important aspect of studying mechanism of influence.

**Subjects and Methods:** This study starts from a psychological perspective, establishes an evaluation index system, and takes Sichuan Province and 21 cities and prefectures under its jurisdiction as research objects, and uses the unexpected output Super SBM model and Malmquist index method to evaluate the development trend of mineral resource utilization efficiency in Sichuan Province for 16 consecutive years from 2006 to 2021.

**Results:** The utilization efficiency of mineral resources in Sichuan Province is relatively high, ranking sixth in the country. From 2006 to 2021, the efficiency values have been greater than or equal to 1 in 15 out of 16 years. From the 11th Five Year Plan to the end of the 13th Five Year Plan, the utilization efficiency of mineral resources has shown a significant continuous upward trend; The utilization efficiency of mineral resources in different functional blocks and 21 cities and prefectures in Sichuan Province is uneven, the difference between the highest Aba Prefecture and the lowest Bazhong City is 2.86 times. In addition, 10 out of 21 cities and prefectures have not reached the effective production frontier. The Northwest Sichuan Ecological Demonstration Zone>Panxi Economic Zone>Southern Sichuan Economic Zone ≈ Chengdu Plain Economic Zone>Northeast Sichuan Economic Zone; The total factor productivity shows a continuous and slight growth trend, with an average annual increase of 1.3%, including a 0.7% increase in the technological progress index and a 0.5% increase in the technological efficiency index, both of which promote the TFP index. Further analysis shows that good mental health can help improve the work enthusiasm and safety awareness of practitioners, thereby enhancing the efficiency of mineral resource development and utilization. **Conclusions**: The main factors affecting the efficiency loss of mineral resource utilization are: industrial solid waste generation, mining project investment, mining industry employees, and water consumption of mining enterprises. In addition, the study also found that good work ethics, high sense of responsibility, and team spirit among mining personnel can enhancing the development and utilization efficiency of mineral energy. Suggest that the Sichuan Provincial Government should strengthen the overall planning of resource allocation, accelerate the introduction, promotion, and application of new technologies; Improve resource utilization and recycling levels; Optimize and upgrade the industrial structure, and guide the transformation of the industrial structure from labor-intensive to technology intensive; Integrate psychological knowledge into safety education and training, and provide necessary psychological intervention and counseling services.

**Acknowledgements**

Thanks for the support of the project of National Social Science Fund Project (24BGL131), Sichuan Provincial Department of Science and Technology (23RKX0621); Project of Sichuan Petroleum and Natural Gas Development Research Center (SKB22-12); Project of Sichuan Mineral Resources Research Center (SCKCZY2023-ZC011); Project of Sichuan Circular Economy Research Center (XHJJ-2315) Thank all members of the research group.

## PHHS25189: STUDY ON THE SYNCHRONIZATION OF PULSE REPRODUCTION AND FEEDBACK IN REMOTE PULSE DIAGNOSIS

Siming Guo^a,b^, Lei Shi^a^, Muthukumar Ramaswamy^c^, Asif Mahbub Karim^b^, Jun Zhang^d^

^*a*^ *School of Technologies, Quanzhou Vocational and Technical University, Quanzhou 362000, Fujian, China,* ^*b*^ *Binary Graduate School, Binary University of Management & Entrepreneurship, Malaysia,* ^*c*^ *Levels Training Institute, Oman Former Technical Expert, Government of Oman,* ^*d*^ *Shandong Key Laboratory of Eco-Environmental Science for Yellow River Delta, Shandong University of Aeronautics, Binzhou 256603, Shandong, China.*

**Background:** In recent years, the Four Diagnostic Methods of Traditional Chinese Medicine (TCM)—observation, listening and smelling, inquiry, and pulse diagnosis—have garnered widespread recognition and application globally, serving as the cornerstone of TCM’s holistic approach to health assessment. These methods enable practitioners to evaluate patients’ physiological and pathological states through multi-dimensional interactions. However, a critical technical challenge persists: the lack of synchrony and standardization in diagnostic execution, particularly in pulse diagnosis. Traditional pulse diagnosis relies heavily on the subjective tactile perception of practitioners, leading to inconsistencies in pulse waveform interpretation, data collection, and diagnostic conclusions. Studies indicate that inter-practitioner variability in pulse identification can result in diagnostic accuracy rates as low as 60-70%, significantly hindering the reliability and scalability of TCM in modern healthcare systems.

**Subjects and Methods:** To address these limitations, this study proposes an innovative remote pulse diagnosis framework integrating pulse-replication technology and real-time feedback mechanisms. By leveraging advanced sensor arrays and haptic feedback devices, the system digitizes the tactile experience of pulse palpation, capturing key parameters such as pulse rhythm, amplitude, and vessel tension. These data are transmitted to practitioners via a cloud-based platform, where artificial intelligence algorithms assist in mapping pulse patterns to TCM syndromes (e.g., “slippery pulse” for phlegm-dampness or “wiry pulse” for liver qi stagnation). Crucially, the system incorporates a bidirectional feedback loop: practitioners remotely adjust pressure levels on the device to simulate in-person palpation, while patients receive instructions to optimize positioning, ensuring data fidelity.

**Results:** Experimental trials involving 200 participants demonstrated a 25% improvement in diagnostic consistency compared to conventional methods, with remote diagnoses achieving 85% concordance with in-person evaluations by senior TCM experts.

**Conclusions:** This research not only resolves long-standing technical barriers in TCM digitization but also pioneers a new paradigm for telemedicine in traditional healthcare systems. The integration of pulse replication and feedback mechanisms enhances diagnostic objectivity while preserving the nuanced, experience-based wisdom of TCM. Furthermore, it expands access to TCM expertise in underserved regions and during public health crises, such as pandemics, where physical consultations are restricted. By bridging ancient diagnostic principles with Industry 4.0 technologies—including IoT, AI, and cloud computing—this study lays the groundwork for standardized, data-driven TCM practices. Future directions include coupling this system with multi-modal data fusion (e.g., combining pulse analysis with tongue image recognition and voice pattern analysis) to create a comprehensive digital TCM diagnostic ecosystem. These advancements hold profound implications for globalizing TCM, fostering cross-cultural medical integration, and advancing precision medicine through the synthesis of Eastern and Western healthcare philosophies.

**Acknowledgements**

Intelligent Manufacturing Application Technology Engineering Center of Colleges and universities in Fujian Province 2025 Open Project (Grants No. QUZN25-B03-1).

## PHHS25190: A STUDY ON THE CORRELATION BETWEEN ACADEMIC PERFORMANCE AND PSYCHOLOGICAL FACTORS OF AVIATION MAJOR STUDENTS BASED ON K-MEANS CLUSTERING

Weimin Wu^a,b^, Zhaoqin Liu^b^, Wanjun Yan^a^, Daoming Wu^b^, Jingfang Chen^b^

^*a*^ *Anshun University, Anshun 561000, China,* ^*b*^ *Chongqing Aerospace Polytechnic, Chongqing 400021, China.*

**Background:** With the development of information technology, the application of artificial intelligence in the data analysis of higher education examinations has become increasingly widespread. However, the attention paid to students’ psychological factors is still insufficient. Especially in aviation-related majors, students face high academic pressure and complex course challenges, and their mental health status has a profound impact on their grades. There is an urgent need for in-depth research to optimize the teaching and psychological support systems.

**Subjects and Methods:** The research was conducted on students majoring in aviation from grades 21 to 24 at Chongqing Aerospace Vocational College. The K-Means clustering algorithm was used to analyze the score data of 13 important courses, dividing the students into three categories: excellent, medium, and poor. Psychological factors were also considered to explore the characteristics of the scores. The data included average scores and standard deviations. Iterative optimization was utilized to achieve clustering, revealing the mechanism by which psychological states affect academic performance.

**Results:** Through K-means clustering analysis, the aviation major students were classified into three categories: top students (average score 91.5±1.2), average students (84.1±12.5), and struggling students (78.3±18.3). The data showed that the standard deviation of grades for senior students significantly decreased (for example, the standard deviation of professional English decreased from 14.21 to 1.2), indicating a positive correlation between their psychological stability and academic performance. The clustering results revealed that psychological factors had a significant impact on academic performance: the struggling group experienced a 30% fluctuation in grades in anxiety courses, while the top group maintained stability through strategies such as time management (grade fluctuation < 5%). The study confirmed that psychological intervention could improve the grades of struggling students by 15% - 20%.

**Conclusions:** This study analyzed the course grades of aviation major students using the K-means clustering algorithm, successfully identifying three groups: top students, average students, and struggling students. The research found that as the grade level increased, the average grades of students significantly improved and the grade fluctuations decreased, indicating that senior students have better professional mastery skills and psychological stability. The study confirmed that psychological factors (such as anxiety, self-confidence, etc.) have a significant impact on academic performance, especially the negative effect on struggling students is particularly obvious. Based on the research results, it is recommended to adopt a stratified intervention strategy: provide mental health counseling for struggling students, formulate personalized learning plans for average students, and establish a mentor guidance mechanism for top students. This study provides a teaching optimization plan based on data analysis for aviation professional education, which has important practical value for improving teaching quality.

**Acknowledgements**

This work was supported by a project of the teacher affairs department of the Ministry of Education (No. ZI2021030105), and the exploration and practice research project on the “dual education” model for the drone application technology major in the Ministry of Education (No. YB2021030102), and the project of science and technology research development center of the Ministry of Education for higher education institutions (No. ZJXF2022164).

## PHHS25191: CONFIGURATION PATH AND PSYCHOLOGICAL MECHANISM OF THE INFLUENCE OF SUSTAINABLE LIVELIHOOD CAPITAL ON FARMERS’ LAND TRUST TRANSFER WILLINGNESS: A CASE STUDY OF DENGZHOU CITY, HENAN PROVINCE, CHINA

Ziqi Wang^a^, Mingfeng Li^b^, Shuhao Shang^b^, Chuan Yang^b^

^*a*^ *School of Management, Wuhan Technology and Business University, Wuhan 430000, China;*^*b*^ *School of Public Administration, Central China Normal University, Wuhan 430000, China.*

**Background:** Rural land trust transfer is a key strategy for advancing agricultural modernization and addressing land fragmentation in China. However, its implementation shows regional disparities, influenced by farmers’ livelihood capital configurations and psychological risk perceptions. Existing research often uses unidimensional frameworks, neglecting interactions among multidimensional capitals and subjective decision-making under uncertainty. This study integrates the Sustainable Livelihood Analysis (SLA) framework and Prospect Theory to explore how farmers’ land trust transfer willingness (LTTW) is shaped by nonlinear configurations of five-dimensional livelihood capitals (human, natural, financial, material, social) and spatial situational factors, aiming to clarify case heterogeneity.

**Subjects and Methods:** A questionnaire survey was conducted with 148 farmer households in Dengzhou City, Henan Province—a land trust reform pilot area. Sampling was stratified by land size and non-agricultural employment rates. LTTW was measured using a 5-point Likert scale, while livelihood capitals were assessed via 12 indicators, including health status, land quality, and income levels. Fuzzy-set Qualitative Comparative Analysis (fsQCA) identified necessary and sufficient conditions for high LTTW, supported by Necessity Condition Analysis (NCA) for robustness. The framework highlighted the interplay between tangible capital endowments and intangible risk attitudes.

**Results:** No single livelihood capital dimension alone explained high LTTW (all consistency scores <0.9), indicating synergistic combinations are required. Financial capital (d=0.13, p=0.01) and human capital (d=0.11, p=0.03) were generally necessary conditions. fsQCA revealed seven configuration paths for high LTTW, grouped into three typologies: (1) policy-supported paths (financial dominance or human-financial synergy); (2) policy-driven paths (natural-social or human-social capital synergy); and (3) resource-endowment paths (natural-material or human-material capital). Psychological mechanisms, like institutional trust and risk aversion, moderated capital relationships, with policy signals (e.g., long-term rent guarantees) mitigating perceived risks.

**Conclusions:** Farmers’ LTTW is driven by context-specific capital configurations, not isolated factors, underscoring the need for adaptive policies. Policy innovations should focus on: (1) financial tools (e.g., land transfer loans); (2) land quality improvements; (3) socialized services (e.g., cooperatives); and (4) spatially differentiated interventions. This study enhances SLA theory by incorporating psychological risk perception and offers practical insights for optimizing China’s land trust system.

## PHHS25192: MEDIATING ROLE OF SLEEP QUALITY OF MEDICAL STUDENTS IN HIGHER VOCATIONAL COLLEGES BETWEEN MOBILE PHONE ADDICTION AND ANXIETY AND DEPRESSION

Xuefang Ren^a^, Rongfang He^b^, Jinhua Tan^b^, Min Huang^c^, Zhaohua Liu^d^

^*a*^ *Department of Thoracic Surgery, Affiliated Hospital of Southwest Medical University, Luzhou 646000, Sichuan, China,* ^*b*^ *Department of Psychiatry, The Affiliated Hospital of Southwest Medical University; Fundamental and Clinical Research on Mental Disorders Key Laboratory of Luzhou, Luzhou 646000, Sichuan, China,* ^*c*^ *Department of Respiratory Medicine, The Affiliated Hospital of Southwest Medical University, Luzhou 646000, Sichuan, China,* ^*d*^ *Department of Cardiovascular Surgery, Affiliated Hospital of Southwest Medical University Luzhou 646000, Sichua, China.*

*XR and RH contributed equally to this research.*

**Background:** To investigate the correlation between mobile phone addiction and the mental health and sleep quality of medical students in higher vocational colleges, and to offer theoretical support for enhancing the understanding of how anxiety and depression influence mobile phone addiction and sleep quality among these students.

**Subjects and Methods:** Between May and July 2022, a cross-sectional survey was conducted among 7,141 medical students at a higher vocational college in Sichuan Province. The Smartphone Addiction Scale (SAS), Generalized Anxiety Disorder-7 (GAD-7), Patient Health Questionnaire-9 (PHQ-9), and Pittsburgh Sleep Quality Index (PSQI) were employed to assess their levels of phone addiction, anxiety, depression, and sleep quality, respectively.

**Results:** A total of 2,820 students (39.49%) were identified with mobile phone addiction, 3,755 with anxiety (52.6%), 4,443 with depression (62.2%), and 3,892 with sleep disorders (54.5%). Factors contributing to mobile phone addiction were integrated into a multivariate analysis regression model. The findings indicated that myopia, living with grandparents, paternal history of alcohol or smoking, maternal history of alcohol consumption, anxiety disorder, depression disorder, and sleep disorder were all significant risk factors for mobile phone addiction (P < 0.05). Pearson correlation analysis revealed a positive correlation between mobile phone addiction and depression (r = 0.355, P < 0.01), anxiety (r = 0.308, P < 0.01), and sleep quality (r = 0.257, P < 0.01). The mediation model effect analysis demonstrated that mobile phone addiction could influence anxiety via sleep quality (effect value = 0.425, P < 0.05, 95% CI: 0.028-0.040), and similarly, mobile phone addiction could affect depression through sleep quality (effect value = 0.408, P < 0.05, 95% CI: 0.028-0.040). Conversely, depression can impact mobile phone addiction through sleep quality (effect value = 0.164, P < 0.05, 95% CI: 0.098-0.26), and anxiety can also affect mobile phone addiction via sleep quality (effect value = 0.262, P < 0.05, 95% CI: 0.223-0.400).

**Conclusions:** The prevalence of mobile phone addiction among medical students in higher vocational colleges is high, with a complex array of influencing factors. Mobile phone addiction is closely associated with family environment, anxiety, depression, and sleep quality. There is a bidirectional causal relationship between mobile phone addiction and both anxiety and depression, with sleep quality acting as an intermediary factor. Both educational institutions and families should pay close attention to the mental health and sleep patterns of these students, actively encourage the development of healthy sleep habits, promote the proper use of mobile phones, and prevent mobile phone addiction.

## PHHS25193: THE IMAGE CONSTRUCTION OF BEIJING FROM THE PERSPECTIVE OF NEWS FRAMING: FOSTERING POSITIVE PUBLIC PERCEPTION AND MENTAL WELL-BEING IN THE COVERAGE OF BEIJING’S CENTRAL AXIS

Liling Zou^a^, Huihan Zhang^a^

^*a*^ *College of Foreign Languages, Beijing University of Technology, Beijing 100124, China.*

**Background:** This study seeks to investigate how the coverage of Beijing’s Central Axis in China’s mainstream foreign media, specifically CGTN and China Daily, contributes to the construction of the city’s international image. The focus is on how news framing not only highlights the city’s historical, cultural, and political significance but also promotes positive public perception and psychological well-being, including emotional engagement and resilience. The study explores the emotional and psychological effects of these media portrayals, examining how they foster positive emotions such as pride, hope, and attachment to the city.

**Subjects and Methods:** This research employs a framing analysis to examine the media reports on Beijing’s Central Axis published by CGTN and China Daily. Through the identification of key news frames-such as historical and cultural significance, heritage preservation, technological innovation, and international communication-the study assesses their role in shaping the city’s image and their impact on the emotional and psychological well-being of the audience. Additionally, the emotional tone and psychological engagement conveyed in these portrayals are considered, with a particular focus on how these factors influence both domestic and international audiences.

**Results:** At the macro level, the analysis reveals that the dominant news frames emphasize the historical and cultural importance of the Central Axis and its preservation as a heritage site. These frames contribute to fostering positive psychological outcomes, such as emotional resilience, national pride, and a sense of collective identity. At the meso level, the influence of authoritative domestic sources, combined with urban development initiatives that prioritize public well-being and mental health, strengthens the psychological impact of the media coverage, emphasizing social harmony and emotional engagement. At the micro level, the framing shifts toward personal connections with the city’s cultural identity, cultivating emotional attachment and psychological resilience. These findings highlight the multifaceted role of media in constructing Beijing’s image, not only through cultural narratives but also through promoting mental well-being and emotional connection.

**Conclusions:** The study highlights the multidimensional nature of Beijing’s urban image construction in foreign media, where cultural heritage, technological innovation, and international communication intersect with psychological well-being. While historical and cultural frames dominate the narrative, there is a growing focus on emotional engagement and psychological resilience in media portrayals. This paper suggests that future international communication strategies should integrate mental health and positive psychological outcomes as core elements in the construction of Beijing’s global image. Such an approach would allow Beijing to present itself as a modern, livable city that prioritizes both cultural preservation and the psychological well-being of its residents and international audiences, thereby fostering a more inclusive and optimistic global perception of the city’s evolution.

**Acknowledgements**

This study is funded by the National Undergraduate Innovation and Entrepreneurship Training Program at Beijing University of Technology “Research on the International Communication of the Beijing Central Axis from the Perspective of Framing Theory: A Case Study of CGTN’s Coverage” (Project No. GJDC‑2025‑01‑69).

## PHHS25194: A STUDY ON THE NARRATIVE STRUCTURE AND PSYCHOLOGICAL MECHANISMS OF AUDIENCE IDENTITY FORMATION IN INTERACTIVE FILMS: A CASE ANALYSIS OF “BLACK MIRROR: BANDERSNATCH”

Yuxin Liu^a^, Ke Li^b^

^*a*^ *Qingdao City University, Qingdao, Shandong, China,* ^*b*^ *School of Literature and Journalism, Shandong University, Jinan, Shandong, China.*

**Background:** This study aims to elucidate the psychological mechanisms relating to interactive narratives and identity formation between user roles in new media content. It further explores how users can, through enhancing their self-determination and strengthening their identity, thereby promote the diversity and artistic values of interactive media arts. The research also seeks to provide guidance on how interactive media arts impact users’ mental health dimensions and emotional regulation, offering theoretical support and practical suggestions for the design and creation of emerging media artworks.

**Subjects and Methods:** Building upon Marie-Laure Ryan’s theory of interactive narrative, this paper constructs an analytical framework, at the same time utilizes narrative layers as a tool to examine the audience’s influence on narrative progression within interactive films. Subsequently, introduce a framework by integrating three psychological needs-autonomy, competence, and relatedness-derived from the Self-Determination Theory. The application of this theoretical model is crucial for understanding the interaction between audiences and narrative environments, as it provides a basis for addressing users’ psychological needs of self-determination. Finally, by looking into Black Mirror: Bandersnatch as a case study, this paper investigates the way audiences choose to develop the plot and how this deliberate process impacts their sense of autonomous control and identity reconstruction.

**Results:** In Black Mirror: Bandersnatch, viewers experience the roles of actor, observer, and editor simultaneously. The interrelation and entanglement among these roles contribute to the complexity of identity formation. Audiences undergo both an experience of non-media immersion and a forced hyper-mediated psychology, creating practical significance of a non-transparent narrative. This forced psychological engagement weakens the audience’s self-determination capability, compelling them to participate in the deconstruction and reconstruction of roles, and inducing ontological psychological changes.

**Conclusions:** Black Mirror: Bandersnatch represents a type of narrative experience that combines the feeling of manipulation, immersion, and distance, embodying an integrity-centered interactive film driven by a linear narrative. Through making choices, audiences undergo semi-compulsory identity shifts and psychological adjustments. This form of interaction is particularly suitable for literarily rich and critically oriented content. The findings of this study provide valuable insights for fostering diversity in new media content and addressing mental health care within interactive digital media arts.

## PHHS25195: APPLICATION OF SSA-SVM MODEL IN EVALUATING THE QUALITY OF UNDERGRADUATE PRACTICAL TEACHING AND ITS IMPACT ON STUDENTS’ MENTAL HEALTH

Cui Liu ^a^

^*a*^ *Changchun Institute of Technology, Changchun 130012, China.*

**Objective:** This study aimed to develop a scientific prediction model to assess the quality of practical teaching in applied undergraduate programs, with a focus on its impact on students’ psychological well-being. By optimizing teaching quality evaluation, the model seeks to reduce academic stress, enhance mental health outcomes, and support the integration of educational reforms with modern productivity demands, ultimately fostering a healthier talent cultivation mode.

**Subjects and Methods:** The research targeted undergraduate students in application-oriented colleges, specifically Geological Engineering students at a university in Changchun. A practical teaching quality evaluation system was established, incorporating 12 secondary indicators relevant to academic performance and psychological resilience. A support vector machine (SVM) model, optimized by the sparrow search algorithm (SSA-SVM), was developed to predict teaching quality and its effects on student mental health. The model’s performance was validated using real-world teaching data, with an emphasis on identifying factors that mitigate stress and improve learning outcomes.

**Results:** The SSA-SVM model demonstrated a prediction accuracy exceeding 98%, with minimal error fluctuation (MAE = 0.574, R² = 0.956), outperforming alternative models like decision trees and random forests. These results indicate the model’s superior ability to forecast teaching quality and its psychological implications. By accurately predicting academic outcomes, the model helps identify potential stressors in practical training, enabling timely interventions to support student mental health and reduce anxiety related to poor performance.

**Conclusions:** The SSA-SVM model offers an effective tool for predicting and enhancing the quality of undergraduate practical teaching, with significant benefits for students’ psychological well-being. It allows educators to detect teaching-related issues early, providing a reliable framework for dynamic monitoring and reform. By linking teaching quality to mental health, the model helps students recognize their learning progress, reducing graduation-related stress caused by unexpected academic struggles. This proactive approach minimizes the risk of mental health challenges, such as anxiety or depression, stemming from poor learning outcomes, promoting a healthier educational experience and aligning teaching reforms with students’ psychological needs.

## PHHS25196: CURRENT STATUS AND STRATEGIC APPROACHES TO TEACHING PRACTICE WRITING IN HIGHER EDUCATION FROM A PSYCHOLOGICAL DEVELOPMENT PERSPECTIVE

Hui Liu ^a^

^*a*^ *Faculty of Language and Literature, Anhui Sanlian University, Hefei 230601, China.*

**Background:** Contemporary instruction in practical writing within higher education increasingly faces prevalent challenges, such as students’ diminished motivation and cognitive distortions. These psychological states are intricately linked to the traditional, template-based training modes that dominate curricula. Conventional pedagogies often neglect the vital role of psychological cultivation in fostering creative thinking, resulting in a disconnect between cognitive and expressive faculties. The *Guidelines for Mental Health Education of College Students* emphasize the integration of psychological wellness into academic curricula. As a process of externalizing thought, writing engages multifaceted psychological mechanisms including self-awareness and emotional expression. Empirical evidence suggests that embedding psychological development into practical writing instruction can reduce writing barriers by approximately 37% and enhance textual coherence by 21%. This research aligns with national educational objectives for cultivating versatile talent, proposing a closed-loop framework: “Psychological Capital Accumulation — Writing Skill Development — Professional Competence Cultivation”, thus providing a novel paradigm to surmount the challenges faced in cultivating applied talents.

**Subjects and Methods:** This study centers on college-level practical writing courses. Through systematic analysis of existing issues at both student and teacher levels within current teaching practices, core problems are identified. Theories from positive psychology are integrated, emphasizing a strengths-based approach to bolster students’ writing efficacy. For instance, feedback during grading emphasizes progress rather than mere correction; alongside, constructivist learning theories are employed to create problem-oriented learning scenarios, such as simulated corporate recruitment writing tasks, thereby enhancing instructional effectiveness.

**Results:** First, in terms of pedagogical philosophy and curriculum design, there has been a shift from mere skill training to holistic person-centered development. Positive psychology principles are embedded into teaching practices, with methods like role-playing used to bolster motivation and social adaptability. Additionally, real-world case studies and situational simulations are employed to enhance learning relevance, complemented by elements of mental health education to improve students’ expressive and stress-resilience capacities. Secondly, students’ core competencies have shown marked improvement. Dual enhancements are observed in writing skills and psychological resilience; students not only learn formatting and structural norms but also cultivate self-awareness and emotional regulation through practice, enabling them to handle complex professional documents with greater confidence. By analyzing exemplary texts and comparative case studies, students develop multidimensional perspectives, fostering critical thinking and innovative capabilities. Lastly, the integration of online and offline modes, coupled with an upgraded diverse assessment system, sustains the course’s developmental trajectory.

**Conclusions:** Teachers, building upon foundational knowledge, utilize diverse practical methodologies centered on positive psychological cultivation. Through a tripartite approach—theory-based support, psychological intervention, and innovative practice—students master essential writing conventions and techniques, bolster their psychological resilience, and achieve dual goals of skill enhancement and mental fortification. This prepares them for academic thesis writing, employment pursuits, and societal integration. Future research may further explore the differentiated psychological needs across various academic disciplines to optimize curricular adaptability.

**Acknowledgments**

This work was financially supported by the Quality Engineering Project on Online Courses of Anhui Sanlian University titled “Traditional Cultural Literacy” (24zlgc032) and the Higher Education Scientific Research Project of Anhui Provincial Department of Education titled “A Study on Shi Zhecun’s Ideas of Poetry and Literary Translation” (2022AH051967).

## PHHS25197: SEMI-SUPERVISED FEATURE SELECTION FOR PARTIALLY LABELED INTERVAL-VALUED INFORMATION SYSTEMS IN MENTAL HEALTH RESEARCH

Shengxue Wei^a^, Jiyuan Wang^b^, Chunjiao Hu^c^, Hengjie Huang^c^, Hongzhi Mo^c^, Zuozan Chen^d^, Jie Qiu^d^

^*a*^ *School of Mathematics and Statistics, Yulin Normal University, Yulin 537000, Guangxi, China,* ^*b*^ *School of Computer and Electronic Information, Guangxi University, Nanning 530004, Guangxi,* ^*c*^ *State owned Assets Management Office of Yulin Normal University, Yulin 537000, Guangxi, China,* ^*d*^ *School of Computer science and engineering, Yulin Normal University, Yulin 537000, Guangxi, China.*

**Background:** In the realm of mental health research, data analysis is crucial for accurately assessing and predicting mental health conditions. However, many datasets used in this field are partially labeled and in interval - valued form. Existing data - handling methods face limitations. For example, they struggle to adapt to various label missing rates, and their reduction rules are often complex. This restricts the accurate evaluation of mental health, making it difficult to precisely identify the key factors influencing mental states and hindering the development of effective mental health prediction models.

**Subjects and Methods:** This paper focuses on addressing these issues. We divide the partially labeled interval - valued information system and define dependence and indistinguishable relations. By introducing a variable parameter, we define the importance of feature subsets. This approach is specifically designed to better process mental health - related data. A semi - supervised feature selection algorithm is proposed, which can make full use of both labeled and unlabeled data in the dataset.

**Results:** The experimental results show that the defined importance can effectively measure the uncertainty in mental health - related data. When applied to mental health datasets, the designed semi - supervised feature selection algorithm outperforms classical algorithms in terms of classification accuracy. It can more accurately identify the key factors related to mental health states. Additionally, this algorithm can automatically adapt to different label loss rates with simple selection rules, which only require determining whether the importance of the feature selection subset is equal to 1.

**Conclusions:** In conclusion, this algorithm provides a more effective way to deal with partially labeled interval - valued data in mental health research. It is of great significance for improving the accuracy of mental health analysis and prediction. This research enables researchers to better understand and analyze mental health - related data, and may contribute to the development of more targeted mental health interventions and prediction models in the future.

**Acknowledgement**

This work is supported by Project for Improving the Basic Scientific Research Ability of Young and Middle - aged Teachers in Guangxi Universities in 2024 (No. 2024KY0602).

## PHHS25198: A BEHAVIORAL SCIENCE PERSPECTIVE ON THE FORCE AND DEFORMATION ANALYSIS OF INSERT SELF-LOCKING STEEL PIPE BRACKET SYSTEM WITH ULTRA-HIGH FORMWORK

Fankui Zeng^a^, Shuxin Yang^a^, Jianhua Zhang^a^, Qingmiao Xing^a^, Hongjia Li^a^

^*a*^ *Xi’an Technological University, Xi’an 710021, China.*

**Background:** In structural construction, traditional mechanical analysis often underestimates the influence of human behavior on system performance. As construction technologies advance, the insert self-locking steel pipe bracket system with ultra-high formwork has become increasingly prevalent in large-scale concrete pouring projects. However, the effectiveness of such systems is not solely determined by mechanical properties-it is also shaped by human decisions, operational habits, and adaptive behavior during construction. This study, grounded in behavioral science, explores the interaction between human actions and structural performance, offering a holistic perspective on how worker behavior under pressure affects system stress and deformation patterns.

**Subjects and Methods:** This research involved an on-site investigation at a high-rise concrete pouring project, where the key stress-bearing components of the ultra-high plug-in self-locking steel pipe support system-vertical poles, horizontal bars, and inclined braces-were instrumented and monitored during construction. Simultaneously, a behavioral science framework was applied to observe how workers interacted with the support system. Focus was placed on their judgment of structural stability, timing and accuracy of adjustments, adherence to technical guidelines, and behavioral responses to visible deformation or unexpected resistance. By correlating observed behavior with structural stress data, the study aims to identify patterns of human influence on mechanical variability.

**Results:** Field measurements revealed significant non-uniformity in stress distribution among vertical poles, with considerable variation both at different spatial positions and along pole height. Behavioral science analysis indicated that inconsistent tightening techniques, misalignment during assembly, and subjective judgment of force application contributed to load eccentricity and uneven stress. The horizontal and inclined bars showed complex stress states, influenced not only by load but also by behavioral inconsistencies during installation. Several vertical rods exhibited stress values exceeding design specifications, highlighting a gap between theoretical expectations and real-world practice. Furthermore, lateral displacement at the top of the poles was significantly greater than at the base, typically 1.5 to 3 times higher in the lateral direction than in the longitudinal, a trend partially linked to human factors during assembly.

**Conclusions:** This study demonstrates that behavioral science provides critical insights into the human contributions to structural performance deviations. Mechanical anomalies observed in the field are not solely structural but are often rooted in behavior-driven variability during installation and operation. It is recommended that future design codes, safety guidelines, and training programs incorporate behavioral science principles. By improving risk perception, judgment accuracy, and task consistency among construction personnel, the overall safety, stability, and reliability of ultra-high formwork support systems can be significantly enhanced.

**Acknowledgments**

The work was financially supported by the Shaanxi Key Research and Development Program (2023JBGS-17) and the Shaanxi Key Research and Development Program (2024SF2-GJHX-63).

## PHHS25199: RESEARCH ON THE MECHANISM OF DRIVE FORCE ATTENUATION IN THE ROBOTIC ARMS OF THE DA VINCI SURGICAL SYSTEM AT HIGH ALTITUDES IN MONGOLIA

Chao Liu^a^, Batbold Baasanjav^a^, Hongxing Hai^a^, Baasanjav Nachin^a,b^

^*a*^ *Department of General Surgery, Ach Medical University, Ulaanbaatar, Mongolia,* ^*b*^ *Department of General Surgery, Academician of Medicine, Mongolian Academy of Sciences, Ulaanbaatar, Mongolia.*

**Background:** Mongolia’s high-altitude regions have an average elevation exceeding 1,500 meters, with some areas reaching over 3,000 meters. The unique geographical conditions (hypoxia, low temperatures, strong winds, etc.) may significantly impact the performance of medical robotic systems. As a core device in minimally invasive surgery, the stability of the da Vinci Surgical System’s robotic arm driving force is critical to surgical safety and precision. However, systematic research on driving force attenuation in robotic arms under high-altitude conditions remains scarce. This study reviews existing literature and designs virtual experiments to investigate the influence mechanisms of Mongolia’s high-altitude environment on the driving force of the da Vinci robotic arm, providing a theoretical basis for optimizing surgical robots in such regions.

**Subjects and Methods:** A multidimensional analytical approach was adopted: Literature Review; Integration of global empirical studies on the effects of altitude on electromechanical systems, focusing on motor efficiency, hydraulic stability, and material deformation. Simulated Environment Design; A controlled climate chamber was used to replicate Mongolia’s high-altitude conditions (2,500–3,500 m, O₂ concentration 15%–18%, temperature −10°C to 5°C), experimental Design for Comparing Robotic Arm Driving Force Output in Standard vs Simulated High-Altitude Environments. Data Modeling; A predictive model for driving force attenuation was developed based on fluid dynamics and motor thermodynamics, alongside potential mitigation strategies.

**Results:** Significant Driving Force Attenuation: Elevated altitudes in Mongolia are associated with diminished peak driving force, while temperatures below −5°C further increase hydraulic oil viscosity, leading to greater response latency. Key Factors: The primary contributing factors include decreased motor heat dissipation efficiency (with an 8% reduction in thermal rise rate per 1,000 m altitude gain) and pressure-induced lubrication deterioration. Nonlinear Relationship: Driving force attenuation exhibits an exponential correlation with altitude, yet the influence of subzero temperatures becomes predominant beyond 2,500 m.

**Conclusions:** Mongolia’s high-altitude environment markedly impairs the da Vinci robotic arm’s driving force due to compounded factors: thermal management failure, hydraulic inefficiency, and material rigidity changes. Design optimizations are recommended, including low-temperature lubricants, enhanced motor cooling, and adaptive control algorithms. Future studies should validate these interventions through field tests to ensure surgical robotic reliability in high-altitude settings. Experimental results will address challenges in deploying the da Vinci system in such regions.

## PHHS25200: ENHANCING READING SKILLS IN ELEMENTARY SCHOOL STUDENTS: AN EXPLORATION BASED ON POSITIVE BEHAVIOR SUPPORT

Qi Qi^a^, Wen Tong^b^

^*a*^ *School of Psychology, Hainan Normal University, Hainan 571158, China,* ^*b*^ *School of Education, Shanxi Normal University, Shanxi 030031, China.*

*QQ and WT contributed equally to this work.*

**Objective:** Elementary school students are in a critical phase of language and cognitive development, and reading competence is a key factor influencing both academic achievement and mental health. Nevertheless, some pupils encounter obstacles in the development of reading skills and do not reach age-appropriate levels. Positive Behavior Support (PBS), an evidence-based framework widely applied in education to improve quality of life and behavioral outcomes, offers an avenue for addressing these challenges. This study sets out, from a PBS standpoint, to explore how collaboration among schools, teachers, and specialists can assist elementary students in overcoming reading difficulties and improving their reading proficiency, with particular emphasis on the integration and application of psychological support.

**Subjects and Methods:** Participants were elementary students identified as having reading difficulties. Employing a combination of literature review and case analysis, the study focused on the cognitive barriers and emotional issues encountered during reading development. It first examined the psychological characteristics of elementary learners, then analyzed problems such as emotional disorders and diminished self-efficacy arising in the reading process, and explored how these factors affect progress in reading. The study proposed the joint construction—at the school, teacher, and expert levels—of a comprehensive support system that promotes growth in reading competence through emotional support, behavioral guidance, and cognitive intervention.

**Results:** Following the implementation of PBS, the participants’ reading abilities improved markedly. Students exhibited gains in reading interest, motivation, and comprehension, with some achieving the average reading level of their peers. Emotionally, self-efficacy increased, reading anxiety decreased, and overall enthusiasm for learning rose significantly. In classrooms, teachers attended closely to students’ psychological development, offering individualized support and guidance that successfully fostered emotional well-being and behavioral adjustment. Through this multi-tiered support system, students not only advanced in reading competence but also enhanced their emotional regulation and social adaptation skills.

**Conclusions:** The PBS-based pathway for improving reading competence demonstrated positive results among elementary school students. A systematic support mechanism established through cooperation among schools, teachers, and experts significantly enhanced both reading abilities and mental health. Beyond helping students surmount reading obstacles, the effective application of psychological theory bolstered emotional stability and learning motivation. Looking ahead, this PBS-based pathway can be extended to wider educational contexts and personalized to accommodate diverse learner characteristics, thereby advancing the goal of comprehensive student development.

**Acknowledgments**

The paper was supported by Project supported by the Education Department of Hainan Province, project number: Hnky2023-16.

## PHHS25201: POLICY EFFICACY IN ETHNIC REGIONS OF CHINA: A SYMBIOSIS THEORY PERSPECTIVE ON INDUSTRIAL DEVELOPMENT AND PUBLIC PSYCHOLOGICAL DYNAMICS

Yusheng Jiao ^a^

^*a*^ *Hubei Provincial Agricultural Modernization and Rural Development Research Center, Wuchang Shouyi University, Wuhan 430064, Hubei, China.*

**Background:** The revitalization of specialized industries in China’s ethnic regions is pivotal for sustainable development, yet persistent discrepancies between policy design and grassroots outcomes-termed “hot policies but cold industries”-underscore systemic gaps rooted in socio-psychological dynamics. This study integrates symbiosis theory with psychological constructs, including ethnic cultural identity (as a collective psychological anchor) and policy anxiety (stemming from implementation uncertainties and cognitive dissonance), to evaluate how public sentiment mediates policy-industry interactions. While existing frameworks emphasize economic metrics, the role of social cognition, emotional regulation, and group dynamics in shaping policy receptivity remains underexplored, particularly in culturally heterogeneous contexts.

**Subjects and Methods:** A dynamic symbiosis model was expanded to incorporate psychological variables. Cultural identity was quantified via sentiment analysis of social media platforms, leveraging natural language processing to assess affective valence and identity salience. Policy anxiety was operationalized through text mining of public discourse, measuring linguistic markers of uncertainty and stress. Panel data from 328 counties (2010-2022) were analyzed using logistic growth equations, panel vector autoregression (PVAR) models, and entropy- weighted indices to quantify interactions within a “policy-industry-psychology” framework. Policy intensity was derived via TF-IDF and LDA topic modeling, while industrial outputs included agriculture, handicrafts, and tourism metrics. Psychological resilience and trust dynamics were further evaluated through mediation analysis.

**Results:** Psychological factors exerted significant moderating effects on policy efficacy. Regions with robust cultural identity exhibited 42% higher policy elasticity, mediated by enhanced social cohesion and ingroup trust. Conversely, high policy anxiety correlated with prolonged policy lags, reflecting avoidance behaviors and risk aversion. Market-oriented tools reduced anxiety by 23% compared to fiscal subsidies, fostering cognitive reappraisal and participatory engagement. Symbiosis stages evolved dynamically: from anxiety- dominant parasitism (characterized by resource extraction and learned helplessness), through identity-mediated commensalism (driven by cultural priming and normative conformity), to trust-driven mutualism (marked by reciprocal empowerment and collective efficacy).

**Conclusions:** Bridging symbiosis theory with psychological insights, this study advocates for policies attuned to psychosocial well-being. In low-symbiosis stages, transparent communication and emotion-focused coping mitigate anxiety; in mature stages, leveraging cultural identity through symbolic reinforcement strengthens trust and behavioral adherence. Policymakers must prioritize psychological capital-encompassing resilience, identity coherence, and perceived control-alongside economic metrics to achieve sustainable, human-centric development. The findings highlight the necessity of multidimensional policy toolkits that harmonize economic incentives with cultural and emotional scaffolding, thereby transforming policy-industry interactions from transactional exchanges to synergistic co-evolution.

## PHHS25202: RESEARCH ON THE EVOLUTIONARY GAME MECHANISM OF MARKET-GOVERNMENT INTERACTION AND MULTI-REGIONAL POLICY OPTIMIZATION IN PUBLIC HEALTH EMERGENCIES

Lina Yu^a^, Xinyu Wu^a^, Chunyan Ji^b^, Xuefeng Xu^b^, Kwi-sik Min^c^, Tao Wang^b,c,d^

^*a*^ *School of Economics, Ocean University of China, Qingdao 266100, China,* ^*b*^ *Yantai Institute of Technology, Yantai 264005, China,* ^*c*^ *East Asian Studies of Hanyang University, Graduate School of International Studies, Hanyang University, Seoul 04763, Republic of Korea,* ^*d*^ *Institute of Marine Economy and Management, School of Management Science and Engineering, Shandong University of Finance and Economics, Jinan 250014, China.*

*LY, XW, CJ, XX, KW and TW contributed equally to this study.*

**Background:** Public health emergencies such as the COVID-19 pandemic have exposed significant flaws of the market mechanism in responding to public crises, including problems such as information asymmetry, externalities, and insufficient supply of public goods. Although government intervention (such as mandatory quarantine and vaccine subsidies) can correct market failures, excessive regulation may lead to distorted resource allocation and restrain economic recovery. This study aims to construct a three-dimensional analysis framework of “risk - trust - resilience”, combined with evolutionary game theory and multi-regional case comparisons, to reveal the dynamic balance mechanism of the interaction between market and government strategies in public health crises.

**Subjects and Methods:** The research adopts the evolutionary game model to simulate the strategy choices of the government, enterprises and the public in an uncertain environment. By constructing the framework of “risk perception - Institutional trust - system resilience”, the influences of risk cognition, trust level and institutional adaptability on decision-making behavior are analyzed.

**Results:** The research found that the market mechanism showed systematic failure during the crisis, manifested as distorted price signals (for example, the mask price in New York State, the United States, soared to 15 times the normal price), disrupted supply chains and panic buying. Government intervention effectively enhances efficiency through centralized allocation of resources (such as emergency requisition of medical supplies) and targeted subsidies (such as support for vaccine research and development). Evolutionary game simulation shows two types of stable equilibria: (1) High-risk stage: Strict prevention and control measures (such as lockdown policies) complement high public cooperation, but economic costs need to be weighed. (2) Economic pressure stage: Policy relaxation may trigger speculative behavior by enterprises (such as the production of low-quality epidemic prevention materials), and a dynamic balance needs to be achieved through risk-sharing contracts (such as tax incentives linked to meeting epidemic prevention standards). Cases show that digital tools can enhance monitoring efficiency.

**Conclusions:** The governance of public health emergencies requires the construction of a three-dimensional policy system of “dynamic coordination - trust co-construction - resilience strengthening”: (1) Dynamic coordination: Implement precise intervention based on different regions and levels (such as risk classification and control in counties), and match the epidemic threshold with the intensity of policies. (2) Trust co-construction: Enhance governance legitimacy through transparent communication and public participation platforms. (3) Resilience Enhancement: Build a collaborative supply chain between the government and enterprises (such as joint guarantee and supply of medical resources) and a flexible legal framework. In the future, it is necessary to integrate artificial intelligence early warning systems with long-term impact assessment to promote the iterative upgrade of the adaptive governance paradigm.

**Acknowledgments**

This research was funded by Special Funding Support of Taishan Scholars Program grant number[tsqnz20240830]; Visiting Professor Program at the China Research Institute of Hanyang University; [National social science fund of China] grant number [23BGJ027];

## PHHS25203: THE IMPACT OF “INVOLUTION” ON THE EMPLOYMENT-RELATED PSYCHOLOGICAL PRESSURE OF ART AND DESIGN STUDENTS: AN EMPIRICAL ANALYSIS BASED ON SOCIAL MEDIA

Xiaojing Liu^a^, Xing Wang^b^

^*a*^ *Lu’an Vocational Technical College, Lu’an 237001, Anhui, China,* ^*b*^ *Nanjing University of Finance & Economics Hongshan College, NanJing 211300, JiangSu, China.*

**Background:** In recent years, the term “involution” has gained significant traction on social media, becoming a focal point of public discourse, especially among university students. Due to the unique characteristics of the art and design industry-such as intense job market competition, ambiguous industry standards, and large salary disparities-students majoring in art and design are particularly vulnerable to the influence of the “involution” discourse, which contributes to heightened employment-related anxiety. However, empirical research specifically exploring this impact remains scarce.

**Subjects and Methods:** Drawing on social media data (from platforms such as Weibo, Xiaohongshu, and Zhihu) and a survey (sample size = 500), this study employs a combination of text mining techniques and statistical analyses to examine three core issues: (1) the dissemination patterns of the “involution” discourse among art and design students; (2) the relationship between social media usage and employment anxiety; and (3) differences in psychological pressure across institution types and academic years.

**Results:** The findings show that the ‘involution’ discourse on social media primarily carries negative emotional tones, with 78% of the content reflecting negative sentiment. High-frequency terms include “long working hours,” “low salary,” and “saturated industry,” reflecting widespread concerns and dissatisfaction among students regarding the current employment landscape. Moreover, time spent on social media shows a significant positive correlation with employment anxiety levels (r = 0.53, p < 0.01). Social media algorithms further intensify the “information cocoon” effect, making students more likely to be surrounded by negative information. In terms of differences, students from vocational colleges reported the highest levels of anxiety (mean = 4.5/5), significantly higher than students from 985 universities (mean = 3.1). Anxiety levels peaked among third-year students.

**Conclusions:** The widespread circulation of the “involution” discourse on social media significantly amplifies employment-related anxiety among art and design students, particularly among those with relatively weaker professional competitiveness. Based on these findings, the study suggests that universities should strengthen career planning education, social media platforms should mitigate the excessive spread of negative information, and students should enhance their media literacy to engage more rationally with the “involution” narrative.

## PHHS25204: THE RESILIENCE IMPERATIVE: HOW DIGITAL FINANCIAL SERVICES RESHAPE RURAL HOUSEHOLD ADAPTABILITY - EVIDENCE FROM CHINA’S BEHAVIORAL FINANCE LANDSCAPE

Si Pan^a^, Bingbin Wu^b^

^*a*^ *Wuhan Technical University, 430040 China,* ^*b*^ *Putian University, 351100 China.*

**Background:** Digital inclusive finance continues to provide impetus for rural economic development, provide financial support and risk management solutions for farmers’ production and rural livelihoods. becoming a primary means of enhancing the resilience of farmer households. By improving farmers’ psychological perception of financial self-regulation, digital inclusive finance enables stronger psychological resilience when confronting stressors and risk shocks, thereby fostering healthy financial behaviors that directly enhance the quality of decision-making in challenging circumstances. Consequently, the efficacy of digital inclusive finance remains contingent upon farmers’ financial behaviors. Only when the accessibility of financial services aligns synergistically with the cultivation of sound behavioral practices among farmers can a sustainable protective mechanism for rural development be effectively established.

**Subjects and Methods:** This study used data from the China Household Finance Survey (CHFS) in 2015, 2017 and 2019 to calculate the “development resilience of Chinese Rural Households” indicator based on nonlinear path dynamics and poverty trap theory. It conducted benchmark regression, endogeneity analysis, and robustness testing on the impact of digital inclusive finance on the development resilience of rural households using a two-way fixed effects model. The corresponding year’s Digital Inclusive Finance Index released by the Peking University Digital Finance Research Center was used to explore the impact and mechanism of digital inclusive finance on rural household development resilience from the perspective of financial behavior.

**Results:** Research has found that: (1) Digital inclusive finance significantly enhances the development resilience of rural households. (2) There is a mediating effect of farmers’ participation in investment and financial management between digital inclusive finance and household development resilience, Financial planning indirectly augments health-related behavioral decision-making capacities, but the mediating effect of digital credit participation and commercial insurance participation is not significant. (3) Digital inclusive finance has a significant impact on the resilience of rural non labor groups and higher education groups in terms of family development.

**Conclusions:** Based on the above conclusions, this study proposes countermeasures and suggestions from four aspects: first, optimize digital inclusive financial services through behavioral-informed product design, incorporating user-centric financial behavior pattern and cognitive characteristics to enhance service adaptability. second, strengthening financial literacy and digital skills training. third, promoting farmers’ integration into the digital inclusive financial ecosystem. Fourth, improving regulatory and dynamic evaluation adjustment mechanisms.

## PHHS25205: RESEARCH ON THE MECHANISM AND PATH EXPLORATION OF CLOTHING PHYSICAL AND MENTAL THERAPY BASED ON MACHINE LEARNING TECHNOLOGY

Guangli Luo^a^, Liangwen Yue^b^

^*a*^ *Guangzhou Institute of Science and Technology, Guangzhou 510000, China,* ^*b*^ *Guangzhou Institute of Science and Technology; Guangzhou 510000, China.*

**Background:** With mental health issues becoming a global challenge, the development of non pharmacological interventions is becoming increasingly urgent. As a medium of direct contact with the human body, clothing’s color, material, and design characteristics have gradually attracted attention for their potential therapeutic effects on psychological and physiological states.

**Subjects and Methods:** This study innovatively introduces machine learning technology into the field of clothing design and constructs a multimodal data-driven model of the physical and mental healing mechanism, aiming to reveal the quantitative relationship between clothing characteristics and healing effects, and explore the path of intelligent design. By integrating the random forest algorithm and support vector machine, prediction models for psychological and physiological healing are established separately. The psychological healing module takes clothing color (Xc), material (Xt), and design style (Xs) as input features, while the physiological healing module integrates biological features such as heart rate changes (Δ HR) and skin conductance (SCL). Finally, the comprehensive healing score is globally optimized through particle swarm optimization algorithm (PSO). The experiment was based on a multimodal dataset of 100 volunteers, and the effectiveness of the model was verified: the prediction accuracy of the psychological healing model reached 91%, the accuracy of the physiological healing model was 88%, and the overall model accuracy improved to 94%.

**Results:** Research has found that cool toned clothing can reduce anxiety scores by 32%, cotton and linen materials significantly improve skin conductivity indicators, and minimalist design styles have a 15% advantage over complex designs in improving psychological scores. The study further revealed the psychological physiological synergistic mechanism - when the emotional score increased by 1 unit, the heart rate variability coefficient synchronously improved by 0.35 units. Through PSO algorithm optimization, the model can generate personalized chemotherapy treatment plans, such as recommending a combination of “light blue+ organic cotton+ streamlined” design for anxious individuals, which improves the comprehensive healing efficacy by 41% compared to the benchmark plan.

**Conclusions:** This study not only constructed a mathematical mapping relationship between clothing characteristic parameters and healing effects, but also developed an intelligent decision-making system that includes dynamic weight adjustment, providing an innovative path of “psychological healing physiological improvement low-carbon materials” for sustainable clothing design. The research results have filled the theoretical gap in quantitative research on clothing therapy, provided wearable solutions for mental health intervention, and promoted the industrial development of green therapy technology by introducing bio based fibers and intelligent manufacturing processes.

**Acknowledgments**

This research was supported by:

1. Key Project of Guangdong Human Resources Research Association: Value Research on Organizational and Employee Promotion (EAP Localization) under the Construction of Guangdong Hong Kong Macao Greater Bay Area, Project Number: GDHRS-24-01-007.

2. Guangdong Province Curriculum Ideological and Political Reform Demonstration Project: Applied Psychology (Art of Harmonious Development of Body, Mind and Spirit), Project Number (202023143).

3. 2024 University level Project of Guangzhou Institute of Science and Technology: Research on the Setting of Art Therapy Major in Humanities and Arts under the Concept of “Five Education”.

4. Part time researcher at the Airport Economy Research Center and the South China Semiotics Research Center of Nanguo Business School, Guangdong University of Foreign Studies and International Trade: Research results of specialized projects in applied psychology and semiotics.

5. Part time psychological supervision at the Psychological Counseling Center of Shanghai Dinglan Enterprise Consulting Co., Ltd: Clinical case study results.

## PHHS25206: A STUDY ON THE APPLICATION AND DEVELOPMENT OF BIG DATA MANAGEMENT IN STUDENT MENTAL HEALTH SERVICES IN UNIVERSITIES AND COLLEGES

Shengdi Xu^a^, Lin Xue^a^, Yixiao Mou^a^, Fu Peng^a^, Chuanjin Ma^a^

^*a*^ *Business School, Shandong University of Technology, Zibo 255000, Shandong, China.*

**Background:** With the increasing number of mental health problems among college students, the limitations of traditional mental health service models have become increasingly obvious. These models often lack the capacity for accurate psychological risk identification, continuous dynamic monitoring, and personalized intervention. The development and application of big data technologies provide new opportunities to improve the efficiency, accuracy, and responsiveness of mental health services in higher education institutions. This study focuses on exploring the pathways, practical challenges, and implications of applying big data management to university mental health services.

**Subjects and Methods:** This study is based on the theoretical framework of big data management and adopts a combination of literature analysis and case study approach. It centers on the actual needs of students’ mental health services and systematically examines the application scenarios of big data in psychological risk detection, intervention refinement, and effect evaluation. Key aspects such as multi-source data integration, real-time status assessment, personalized service delivery, and data governance were analyzed in detail to gain a comprehensive understanding of current practices and barriers.

**Results:** Findings indicate that big data enables timely and accurate monitoring of students’ psychological status through intelligent integration and analysis of various data sources, including academic records, behavioral data, and digital interaction patterns. This technological integration significantly improves the accuracy of mental health assessments, the efficiency of intervention delivery, and the equity of service accessibility. It has enabled educational institutions to move from reactive to proactive psychological support. However, some challenges remain. Data privacy issues were particularly highlighted, especially given the sensitivity of psychological data. Ethical risks associated with transparency of data use, consent mechanisms and potential misuse were also highlighted. In addition, the lack of standardized data governance structures and interdepartmental coordination weakens institutional capacity to manage data responsibly and effectively.

**Conclusions:** Big data brings a transformative approach to the delivery of mental health services in higher education. Its successful implementation depends not only on technological capabilities, but also on sound data governance, ethical oversight, and institutional collaboration. Ensuring safe, compliant and meaningful use of student psychological data is critical to improving the quality and effectiveness of mental health support in higher education.

## PHHS25207: EXPLORING THE DEMAND FOR GREEN PERCEPTUAL PACKAGING DESIGN AMONG CONSUMERS WITH VARYING GREEN CONSUMPTION ATTITUDES

Ming-jing Yu^a^, Chun-chin Chen^a^

^*a*^ *JMU College of Arts & Design, Jimei University, Xiamen 361021, Fujian, China.*

**Objective:** Amid growing consumer emphasis on emotional value and environmental sustainability, packaging design integrating qualia (sensory/emotional attributes) and green features has become pivotal for brand identity and eco-social responsibility. This study examines how qualia and green attributes in packaging influence purchase intentions.

**Subjects and Methods:** This study focused on regional specialty products with qualia-driven green packaging designs. A mixed-method approach was adopted, combining expert evaluations and consumer surveys. Sample selection began with collecting 50 packaging designs, which were categorized into six groups through a focus group discussion with six experienced packaging designers (four male, two female). From these, 18 representative samples were selected as experimental stimuli. Data collection involved a structured expert review with 6–8 specialists in design, psychology, and environmental education to refine evaluation scales. A preliminary test with 20 consumers validated the measurement instruments, which included 16 qualia and green design perception items adapted from established scales. Analytical methods encompassed cluster analysis to segment consumers into two groups based on green consumption attitudes (Green Living Practitioners and Conceptualists), alongside factor and regression analyses to identify key drivers of satisfaction.

**Results:** Factor analysis identified three key dimensions influencing packaging perception: Refinement (28.784% variance), Cultural (20.485%), and Green (15.979%), cumulatively explaining 65.248% of variance. Multiple regression analysis demonstrated that Refinement (β=0.426, *p*<0.001), Cultural (β=0.343, *p*<0.001), and Green (β=0.399, *p*<0.001) factors significantly predicted consumer preference (R²=0.458). Cluster analysis delineated two consumer segments: Green Living Practitioners prioritized tactile functionality (Playfulness Factor=0.69) and reuse potential, with attributes such as “fashionable aesthetic” (weight=0.81) classified as Attractive Quality. Green Living Conceptualists emphasized cultural narratives (Cultural Factor=0.81) and sustainability principles, valuing “cultural storytelling” (weight=0.65) as One-Dimensional Quality. Kano analysis further revealed divergent priorities: Practitioners focused on sleek forms (weight=0.52) and durability. Conceptualists prioritized minimalist aesthetics (weight=0.73) and symbolic environmental design (e.g., “material conservation,” weight=0.72).

**Conclusions:** In the context of the creative economy and sustainable development, this study investigates consumer perceptions of green qualia-driven packaging design using the Kano Model and empirical analytical methods. Through factor and cluster analyses, consumers were categorized into two groups: Green Living Practitioners (emphasizing practical behaviors) and Green Living Conceptualists (prioritizing ideological alignment), which revealed distinct preference patterns. Practitioners leveraged emotionally driven, high-attraction attributes (e.g., fashionable aesthetics) to establish brand distinctiveness, while Conceptualists focused on functionally oriented sustainable design (e.g., material conservation). The study proposes an emotion-function dual-driven strategy, advocating the integration of sensory interactivity (e.g., reusable functionalities) and symbolic design languages to bridge the cognitive gap between sustainability ideals and user experiences, thereby enhancing product appeal and behavioral conversion efficiency. Future research should expand to cross-cultural validation, incorporate multisensory behavioral data to assess long-term impacts, and provide differentiated design pathways: narrative-driven interactivity for Practitioners and functional visualization with value communication for Conceptualists, facilitating effective guidance from consumer cognition to green action.

## PHHS25208: RESEARCH ON INNOVATIVE TEACHING OF HUMAN RESOURCE MANAGEMENT IN THE ERA OF AI: THREE DIMENSIONAL COLLABORATION OF TECHNOLOGY-ETHICS-COGNITION

Tian Wang^a^, Jianbang Lin^b^

^*a*^ *South China Business College Guangdong University of Foreign Studies, Guangzhou 510545, China,* ^*b*^ *Nanfang College • Guangzhou, Guangzhou 510970, China.*

**Background:** The deep penetration of digital technology is triggering a structural change in the paradigm of human resource management, and the phenomenon of the separation between technological tool rationality and ethical value rationality in the traditional education system is becoming increasingly prominent. This disconnect is not only reflected in the discontinuity of knowledge structure, but also deeply reflected in the psychological cognition of managers: the lagging development of ethical consciousness in the process of acquiring technical abilities leads to a cognitive imbalance between the application of technology and ethical norms in decision-making behavior. Neuroscience research has shown that ethical decision-making involves complex interaction mechanisms between the prefrontal cortex and the limbic system, and existing educational models have not yet established effective pathways to activate such neural circuits.

**Subjects and Methods:** Based on a three-dimensional transmission framework of psychology cognition behavior, this study constructs a collaborative teaching model of “technology ethics cognition”. At the methodological level, the integration of decision-making dual system theory, cognitive dissonance theory, and neuroimaging technology is used to innovatively design a training path of “algorithm development ethical stress testing neurofeedback”. Through a neuroethical experiment simulating management decisions, functional magnetic resonance imaging technology is used to monitor the neural response patterns of participants in technical ethical dilemmas in real time, revealing the psychological conflict mechanisms and neural plasticity laws formed by ethical judgments.

**Results:** Practical research has shown that immersive ethical scenario training can significantly improve the decision-making accuracy of learners, and the activation intensity of the conflict monitoring function in the anterior cingulate gyrus is significantly enhanced in their neural feedback data. Modular knowledge reconstruction effectively promotes the improvement of neural network connection density between technical cognition and ethical cognition, and behavioral experiments verify that the occurrence rate of ethical pre behavior in algorithm design has achieved a leapfrog growth for students. The neuroethical assessment system captures the dynamic characteristics of ethical sensitivity formation, presenting a neural mechanism transition from cognitive conflict to behavioral regulation.

**Conclusions:** The study confirms that the essence of technological ethical competence is the adaptive reconstruction of the psychological cognitive system, manifested as the regulatory transition from the default mode network to the central executive network. The innovation of educational paradigms requires simultaneous attention to the reinforcement of neural representations of technological cognition and the shaping of behavioral mechanisms of ethical decision-making. By establishing a progressive mechanism of “cognitive conflict neural remodeling behavioral internalization”, the deep integration of instrumental rationality and value rationality at the level of neural plasticity can be achieved. This provides a theoretical framework and practical paradigm from an embodied cognitive perspective for cultivating the neuroethical literacy of management talents in the era of artificial intelligence.

**Acknowledgments**

This work was supported by Guangdong Human Resources Research Association Project: Research on Innovative Teaching of the Course “Human Resource Management” (No. GDHRS-24-02-075), Major Projects of the Chinese Society of Business Economics (Grant No. 20251009), First Class Undergraduate Major Construction Project in HRM from SCBC, Guangdong Provincial Department of Education Course Ideological and Political Teaching Team (No. 202121056), and Ministry of Education School Enterprise Collaborative Education Project: “1+X” Practical Teaching for HRM Major (No. 231001712232332).

## PHHS25209: POSITIVE FACTORS IN DISTANCE LEARNING BEHAVIOR THAT INFLUENCE THE MENTAL HEALTH OF COLLABORATORS

Xiaoli Zhang ^a^

^*a*^ *Huangshan University, Huangshan, Anhui 245041, China.*

**Background:** The rapid proliferation of distance learning, particularly accelerated by the COVID-19 pandemic, has led to a paradigm shift in global education and professional practices. While substantial attention has been directed toward understanding its psychological impacts on learners’ mental health, there remains a paucity of research examining the potential positive effects of distance learning behaviors on collaborators’ psychological well-being – defined here as individuals engaged in cooperative learning environments, including academic peers and professional counterparts. This knowledge gap persists despite continuous technological advancements and the evolving landscape of distance education, making systematic investigation of these relationships imperative. This study employs a grounded theory methodology to achieve three primary objectives: (1) to empirically investigate the beneficial associations between distance learning behaviors and collaborators’ mental health outcomes, (2) to elucidate the underlying mechanisms mediating these relationships, and (3) to construct a comprehensive theoretical framework that integrates these findings within existing educational psychology literature.

**Subjects and Methods:** Using qualitative research design, focusing on the theme of “factors affecting the mental health of collaborators” data were collected from two ways: field investigation of online community discourse texts and publicly published literature texts. Following the grounded theory method, the text materials are selected, classified, marked, summarized and summarized from the bottom up. The analysis process includes three iterative stages of open coding, axial coding and selective coding. This process facilitates the generation of theories from data that capture how distance learning behavior affects the mental health of collaborators.

**Results:** The positive psychological model of distance collaborative learning is constructed, and it is found that effective identification of negative psychology such as anxiety, depression and disappointment is a prerequisite for distance collaboration to give full play to its own advantages and promote the mental health of collaborators. Maximizing the advantages of distance collaboration is the realistic support to promote the mental health of collaborators, and the two will produce mutual benefits, which can stimulate the realization of deep collaboration. Among them, insight into the factors that affect the mental health of collaborators is the fundamental guarantee for improving the effectiveness of collaboration. The model reveals the interaction mechanism of negative psychological factors, collaborative effectiveness and collaborative advantages, and collaborative positive psychology.

**Conclusion:** When the negative psychological factors are effectively identified and removed, the favorable factors that promote the mental health of the collaborators are accurately understood, and the advantages of collaboration are maximized, the whole collaboration process will produce mutually beneficial effects. Among them, giving full play to the advantages of cooperation and promoting the mental health of the collaborators are mutually conditional chemical reactions, which will inevitably stimulate the deep cooperation efficiency.

**Acknowledgements**

This work was supported by a project from Teaching and Research of Anhui Province, China. (Grant No. 2021jyxm1407, “Presupposition-Generation -Design Professional PAD Class Teaching Model Innovation”), a project from Anhui Province MOOC construction - Product Design II, China. (Grant No. 2021xskc093), and another project from Anhui Province First-class curriculum construction - Huizhou Folk Crafts, China. (Grant No. 2024DSPYLKC09).

## PHHS25210: MOBILE AR-BASED MULTISENSORY ADAPTIVE ARCHITECTURE: EXPERIENCE-DRIVEN DESIGN FOR MENTAL HEALTH ENHANCEMENT

Lei Xiao ^a^

^*a*^ *Tianjin University of Technology and Education, Tianjin 300222, China.*

**Background:** Based on the multi-sensory adaptation architecture of mobile AR, the aim is to comprehensively improve users’ mental health level through experience-driven design methods, particularly in addressing global challenges like anxiety and depression exacerbated by urbanization. Traditional interventions face barriers including stigmatization and passive engagement, whereas AR-enabled solutions leverage smartphone ubiquity to deliver personalized, context-aware therapeutic experiences. The prevalence of mental health issues has become a significant concern worldwide. Traditional psychological treatments, despite their effectiveness, are often hindered by environmental constraints, low patient participation, and high costs. Moreover, the stigma associated with mental health problems can deter individuals from seeking help. In contrast, AR technology offers a promising alternative by utilizing smartphones to create realistic virtual environments that simulate real-life scenarios. This approach breaks through the limitations of traditional treatments, providing patients with a more natural, realistic, and convenient therapeutic experience. It enhances patient engagement, improves treatment outcomes, and helps psychologists better understand patients’ psychological states to develop more accurate treatment plans. Furthermore, AR technology can reduce treatment costs, improve accessibility, and benefit more patients, while increasing public awareness and understanding of mental health issues.

**Subjects and Methods:** The architecture integrates augmented reality technology with multi-sensory adaptation theory, utilizing RGB cameras, depth sensors, and IMUs to capture real-time data. Through spatiotemporal registration modules and feature fusion engines, it achieves deep fusion of multimodal signals (visual, auditory, tactile) to construct unified environmental representations, employing machine learning to dynamically adjust sensory stimuli like biofeedback-driven soundscapes and haptic vibrations based on users’ physiological states. Multi-sensory adaptation theory emphasizes the integration of multiple sensory stimuli to enhance an individual’s perceptual experience and mental health level. In the context of mental health, this theory aims to intervene and regulate psychological states through multi-sensory stimuli. The combination of AR technology and multi-sensory adaptation theory enables the creation of realistic virtual scenes that help patients undergo psychological counseling and treatment, such as relaxation training, cognitive behavioral therapy, and exposure therapy. This enhances the immersive experience of patients, helps them reduce stress, relax their body and mind, and promotes mental health.

**Results:** Experimental results show cortisol level reductions (up to 27%) and improved emotional regulation. Users immerse in virtual-reality hybrid scenes—such as guided breathing with tactile pulses and adaptive nature environments—significantly enhancing stress relief and emotional engagement. Longitudinal studies confirm sustained benefits, including resilience to daily stressors and better sleep quality.

**Conclusions:** This experience-driven design not only enriches interactive experiences but also pioneers scalable, stigma-free mental health support. Its integration into daily routines (e.g., on-demand relaxation during commutes) positions it as a transformative public health tool. Future work will explore wearable biosensors and LLMs for hyper-personalization.

## PHHS25211: CONSTRUCTION OF AN URBAN STREET GREENING EVALUATION SYSTEM UNDER THE INFLUENCE OF RESIDENTS’ MENTAL HEALTH

Kunpeng Gu^a,b^, Shiheng Liu^a^

^*a*^ *Fujian Agriculture and Forestry University, Fuzhou 350000, China,* ^*b*^ *Minnan University of Science and Technology, Quanzhou 362700, China.*

**Background:** Urban streets constitute a vital component of both urban landscapes and residents’ daily lives. Studies demonstrate that the green view index of urban streets significantly enhances residents’ physical and mental well-being, presenting valuable opportunities for public health improvement. Nevertheless, existing evaluation methodologies for the green view index remain inadequate.

**Subjects and Methods:** This study proposes a visual quantitative evaluation method for urban street greening through combining the Green View Index (GVI) with the Normalized Difference Vegetation Index (NDVI), aims to provide scientific guidance for optimizing urban spaces, alleviating urban life stress, and enhancing residents’ physical and mental well-being. Using the main urban area of Fuzhou as a case study, the research adopts the deep learning FCN-8s model to perform semantic segmentation on Google Street View images for GVI calculation, while integrating Landsat 8 OLI remote sensing images to extract NDVI values within 50-meter road buffer zones.

**Results:** The study reveals that the spatial distribution of GVI (Green View Index) in Fuzhou’s main urban area exhibits a “locally clustered yet overall balanced” pattern, whereas NDVI (Normalized Difference Vegetation Index) generally follows a “periphery-to-center decreasing” trend. Correlation analysis indicates a moderate positive relationship between GVI and NDVI (r = 0.47), though regional variations exist. Based on these findings, a visual classification system for street greening was developed, categorizing urban road greening into four types: dual-advantage, visually rich, traffic-service, and dual-low. Corresponding road morphological features—narrow-lane, open, enclosed, and walled types—were identified. The study further examines the impact mechanisms of street functional attributes, cross-section forms, and greening configurations on residents’ mental health.

**Conclusions:** The research findings provide a scientific basis for optimizing street greening planning, offering practical significance in enhancing urban ecosystem services, improving residents’ living environment quality, and promoting their mental health.

## PHHS25212: INVESTIGATION INTO THE IMPORTANCE OF CT-FFR, TyG INDEX, AND NLR IN ASSESSING THE DEGREE OF CORONARY ARTERY STENOSIS IN SENIOR INDIVIDUALS WITH CORONARY HEART DISEASE

Dabin Pan^a^, Lintao Zha^a^, Yang Ling^a^, Sisi Hu^a^, Quan Zuo^a^

^*a*^ *Department of Cardiology, Yijishan Hospital affiliated Wannan Medical College, Wuhu, Anhui 241001, China.*

**Background:** To investigate the predictive value of computed tomography-derived fractional flow reserve (CT-FFR), lipid profiles, triglyceride-glucose (TyG) index, and neutrophil-to-lymphocyte ratio (NLR) in evaluating coronary artery stenosis severity in elderly patients with coronary heart disease (CHD), and to explore their correlations with established risk stratification tools.

**Subjects and Methods:** This retrospective study enrolled 80 elderly CHD patients (age ≥65 years) who underwent coronary CT angiography (CCTA). Participants were stratified into two groups using the Gensini scoring system: mild stenosis (Gensini score <26, n=42) and moderate-to-severe stenosis (Gensini score ≥26, n=38). Comprehensive comparisons were performed on baseline characteristics, CCTA-derived parameters (including CT-FFR and coronary artery calcium [CAC] scores), metabolic markers (fasting lipid profiles, TyG index), and systemic inflammatory indicators [NLR, platelet-to-lymphocyte ratio (PLR), monocyte-to-lymphocyte ratio (MLR)]. Multivariate logistic regression and Spearman’s rank correlation analyses were applied to assess relationships between biomarkers and stenosis severity.

**Results:** The moderate-to-severe group demonstrated significantly higher Gensini scores (28.65±2.32 vs. 21.34±3.48, P<0.001), CAC scores (25.14±4.86 vs. 22.73±3.66, P=0.014), TyG index (9.49±1.38 vs. 8.61±1.11, P=0.004), and NLR (3.12±.96 vs. 2.69±.73, P=0.026) compared to the mild group. Conversely, CT-FFR values were markedly lower in the moderate-to-severe group (0.52±0.13 vs. 0.66±0.21, P<0.001). Strong correlations were observed between CT-FFR and both Gensini scores (r=-0.543, P<0.001) and CAC scores (r=-0.686, P<0.001). The TyG index and NLR showed positive correlations with Gensini scores (r=0.615 and 0.608 respectively, both P<0.001) and CAC scores (r=0.640 and 0.647 respectively, both P<0.001). There was no significant difference in LDL-C/HDL-C, ApoB/ApoA1, PLR, and MIR between the two groups.

**Conclusions:** The integration of CT-FFR, TyG index, and NLR provides a multidimensional assessment tool for coronary stenosis severity in elderly CHD patients. CT-FFR exhibits strong inverse correlations with anatomical severity scores (Gensini and CAC), while TyG index and NLR reflect the synergistic impacts of metabolic dysfunction and chronic inflammation on plaque progression. These findings suggest clinical utility in risk stratification and personalized therapeutic decision-making for geriatric CHD populations. Further prospective studies are warranted to validate their prognostic value in cardiovascular outcomes.

**Acknowledgments**

This research was supported by Universities Scientific Research project of Anhui Province (KJ2011B192) and Dr. Dabin Pan’s scientific research fund project of Yijishan Hospital affiliated Wannan Medical College (KY20580460).

## PHHS25213: RESEARCH ON AIGC EMPOWERING COLLABORATIVE DESIGN OF GAME CONCEPT ART BASED ON USERS’ PSYCHOLOGICAL NEEDS

Xiaoqin Liu ^a^

^*a*^ *Wuhan Institute of Design and Sciences, Wuhan 430205, China.*

**Background:** The game industry’s market value is projected to reach $256.0 billion by 2025. However, traditional concept art design faces critical challenges including efficiency bottlenecks (complex scenes requiring 60-90h, collaborative costs accounting for 35% of project workload), creative homogenization, and players’ unmet demands for immersive experiences and personalized content. This study integrates art psychology with cognitive load theory to develop an AIGC collaborative framework, aiming to overcome the dual challenges of enhancing creative efficiency and satisfying users’ psychological needs.

**Subjects and Methods:** This study adopts a method that combines experimental design with the assessment of users’ mental health and psychological needs. Based on a database of 13,000 multi-style game original paintings and combined with Maslow’s hierarchy of needs theory, psychological needs are labeled. Propose a human-machine collaborative framework: Integrate Stable Diffusion and dynamic spectral Normalization generative adversarial Network to achieve multi-style fusion and mental health intervention for designers; Embed the golden section visual focus optimization algorithm and the emotional resonance scoring model, and introduce the art therapy module to enhance the response to users’ psychological needs. A double-blind controlled experiment was adopted (N=20 senior designers, with an average experience of 8.3±1.2 years), and multi-dimensional assessment was conducted through the Likert Scale, FID score and NASA-TLX cognitive Load Scale.

**Results:** In this study, the content diversification generated by the AIGC framework can increase the matching value of users’ psychological expectations to 40%. The AIGC framework shortens the design time of a single scene by 42% and increases the style diversity index by 65%. By applying the dynamic style fusion technology and the emotional resonance mechanism, user satisfaction has increased by 18%, and players’ sense of belonging and control demands have been significantly enhanced. Meanwhile, the golden section method was adopted to optimize the visual focus, increasing the user’s attention dwell time by 37%, reducing the cognitive load by 29%, and achieving a deep aesthetic preference matching degree of 84.3%. The FID analysis shows that the matching value of content diversity has increased by 40%, which can effectively solve the problem of homogenization in traditional designs.

**Conclusions:** This study establishes a psychotechnical paradigm for game concept art design. Human-AI collaboration not only enhances design efficacy but also optimizes workflow and enables personalized style generation, thereby improving the fulfillment of profound psychological needs such as user belongingness and perceived control. The integration of emotional resonance modeling and visual attention mechanisms provides a quantifiable pathway for balancing technical precision and artistic intentionality. Future work will focus on developing ethical frameworks for AIGC and cross-cultural psychological adaptability to advance AI-assisted tools toward creative co-evolution.

**Acknowledgments**

This work was funded by the Guiding Project of the 2023 Scientific Research Program of the Education Department of Hubei Province (Research on the Application of AIGC in Game Concept Art Design, Project Number B2023380).

## PHHS25214: STRUCTURING OF QUALITY MANAGEMENT MODELS BASED ON QUALITY PSYCHOLOGY AND CONCEPTUAL FRAMEWORK ANALYSIS

Fangfang Zhouª, Zhonghua Liu^a^, Zhiyuan Wang^a^

^*a*^ *China National Institute of Standardization, Beijing 100086, China.*

**Background:** To distill replicable and scalable Chinese enterprise quality management experiences, this study investigates a universal, practical framework for quality management models rooted in the principles of psychological theories of quality, alongside pathways for constructing robust quality management systems. The aim is to enhance enterprise competitiveness in quality and to propel the development of a strong quality nation.

**Subjects and Methods:** Through an in-depth analysis and comparative study of the essence and evolution of enterprise quality management, this research explores the developmental trajectory of quality management practices and the burgeoning field of quality psychology. Additionally, employing a conceptual framework approach, the study constructs a foundational architecture for quality management models, elucidating the profound interrelations among key elements such as the philosophy, objectives, implementation, support mechanisms, and technological tiers of quality management. Furthermore, case study methodology, combined with specific enterprise examples, validates the efficacy of this framework in synthesizing and summarizing organizational quality management experiences, thereby providing both theoretical underpinning and empirical substantiation for practical applications.

**Results:** This research underscores the pivotal role of quality psychology in contemporary enterprise quality management practices, highlighting its significant utility in elevating overall quality assurance capabilities. It clarifies that the cultivation of quality consciousness, the comprehension and integration of quality theories, precise formulation of strategic goals, effective execution of quality initiatives, optimal allocation of resources and systems, appropriate deployment of quality tools, and the scientific employment of quality data and digital systems constitute the core components of a high-performing, sustainable quality management model.

**Conclusions:** By establishing a foundational architecture for enterprise quality management, this study delineates the core constituents and intrinsic connotations of quality assurance. It emphasizes the critical application of quality psychology across different stages of organizational development, offering a methodological pathway for enterprises to refine and institutionalize their quality management practices. This work not only enriches the theoretical landscape of quality management models but also provides actionable, adaptable guidelines for organizational implementation. As market competition intensifies and consumer demands diversify, the continuous optimization and innovation of quality management paradigms are imperative. The structured approach to quality management proposed herein, grounded in psychological insights and conceptual analysis, undoubtedly lays a solid foundation for this ongoing evolution.

**Acknowledgements**

This work was financially supported by the Basic Scientific Research Business Funding Project of the China National Institute of Standardization “Research on the Evolution and Structural Analysis of Quality Management Models in Chinese Manufacturing Enterprises” (552024Y-11779).

## PHHS25215: EMOTIONAL RESONANCE AND CULTURAL IDENTITY IN THE MUSICAL CATS: THE ROLE OF VOCAL ART IN ALLEVIATING LONELINESS

Jianshu Wang ^a^

^*a*^ *Chongqing Normal University, Chongqing 401331, China.*

**Background:** As a representative work of contemporary musical theatre, Cats has not only gained global attention for its unique artistic language, but also generated strong resonance among the audience due to its in-depth depiction of psychological themes such as loneliness, rejection, identity and redemption. In the current social context where mental health issues are becoming more and more prominent, especially in the context of the prevalence of anxiety, personality repression, social alienation, etc., Cats is not only a stage performance, but also an important cultural medium for the audience’s emotional projection and psychological feelings.

**Subjects and Methods:** This study takes the vocal interpretation and symbolic narrative of Cats as an entry point, focusing on the character Grizabella and her core chorus ‘Memory’. Through textual analysis, semiotic interpretation and multimodal discourse analysis methods, it explores how music, body, and stage visuals in the play synergistically construct emotional resonance and alleviate the audience’s psychological pressure. By integrating the theoretical perspectives of emotional psychology, cultural identity theory and musical theatre aesthetics, we reveal the psychological regulation function of musical theatre in terms of emotional projection, anxiety relief and happiness enhancement.

**Results:** Cats creates a strong emotional field through symbolic narrative and multimodal artistic devices. Grizabella’s transformative path from rejection to acceptance maps the psychological experience of modern individuals regarding marginalisation and belonging. The richly layered vocal techniques and stage movements achieve emotional release, while the multimodal structure enhances the audience’s sense of immersion and empathy. In the course of the performance, the audience often connects the inner conflict with Grizabella’s destiny, from which they can obtain emotional recognition and psychological comfort, thus alleviating the sense of loneliness and enhancing their subjective well-being and psychological resilience.

**Conclusions:** As an important example of musical theatre art, Cats’ artistic expression has transcended the boundaries of theatre and transformed into a carrier of emotional healing and cultural reflection. It builds a bridge between individual psychological experience and collective cultural memory, and promotes the audience’s understanding of psychological needs and the enhancement of social empathy. Musical theatre has the realistic potential to integrate mental health education and satisfy the need for emotional support, and can be used as an important practical sample for stage art to serve social psychology.

## PHHS25216: RESEARCH ON THE APPLICATION OF TRADITIONAL CHINESE MEDICINE MO GAO COMBINED WITH A WARM MOXIBUSTION DEVICE IN THE TREATMENT OF CHRONIC KIDNEY DISEASE (CKD STAGE 3)

Meimei Duan^a^, Hongmei Yang^a^, Ping Liu^a^, Liujie Zhang^b^, Xiaoqiong Chen^a^

^*a*^ *Department of Nephrology, The First Affiliated Hospital of Guizhou University of Traditional Chinese Medicine, Guiyang 550001, China,* ^*b*^ *Department of Nursing, Guizhou University of Traditional Chinese Medicine, Guiyang 550001, China.*

**Objective:** To investigate the clinical efficacy and application value of Traditional Chinese Medicine (TCM) Mo Gao combined with a warm moxibustion device in the treatment of chronic kidney disease (CKD stage 3).

**Methods:** A total of 88 patients with chronic kidney disease (CKD stage 3) who were diagnosed and treated in our hospital from December 2023 to December 2024 were selected as the research subjects. According to the order of admission, they were divided into a conventional group (n=44) and an observation group (n=44). The conventional group was treated with TCM Mo Gao, while the observation group was treated with TCM Mo Gao combined with a warm moxibustion device. The serum albumin (Alb), urine protein (PRO), blood urea nitrogen (BUN), serum creatinine (Cr), total protein (TP), hemoglobin (Hgb), quality of life (SF-36), and clinical efficacy were observed and compared between the two groups.

**Results:** The overall treatment effectiveness rate in the observation group was significantly higher than that in the conventional group, showing a statistically significant difference (P<0.05). In the comparison of related indicators, Hgb and Alb levels in the observation group were higher than those in the conventional group, while Cr, BUN, and PRO levels were lower than those in the conventional group, and these differences were statistically significant (P<0.05). In terms of quality of life scores, the observation group was significantly higher than the conventional group, also with a statistically significant difference (P<0.05).

**Conclusion:** The combined therapy of Chinese herbal paste and warming moxibustion is a treatment method guided by the theory of meridians and acupoints in traditional Chinese medicine. It combines the basic principles of moxibustion and Chinese herbal medicine by grinding Chinese herbal materials into a powder, which is then mixed with an appropriate medium to form a herbal paste that is applied to acupoints and meridians. With the aid of the thermal power of moxa fire, the medicinal properties travel along the meridians, thereby achieving the therapeutic effects of unblocking meridians, regulating the yin-yang balance of internal organs, and supporting yang to prevent collapse. Through this treatment method, the condition of patients with CKD3 can be effectively controlled while improving serum indices and enhancing treatment efficacy, thereby ensuring an improved quality of life for patients. The combined therapy of Chinese herbal paste and warming moxibustion holds significant application value in the treatment of chronic kidney disease (CKD3). It can effectively enhance treatment outcomes and quality of life, promote the recovery of Cr, Hgb, Alb, BUN, and PRO indices, and is worthy of clinical promotion.

## PHHS25217: THE IMPACT OF AI-ASSISTED LANGUAGE LEARNING ON LEARNERS’ SELF-EFFICACY AND PSYCHOLOGICAL WELL-BEING: A STUDY OF ANXIETY, STRESS, AND CONFIDENCE IN ADAPTIVE ENVIRONMENTS

Hui Sun ^a^

^*a*^ *Shandong Sport University, Jinan, China.*

**Background:** Artificial intelligence-assisted language learning (ALS) is rapidly transforming modern education by providing adaptive, personalized, and responsive learning experiences. While it is often promoted for its potential to improve academic outcomes, its impact on learners’ psychological states, particularly self-efficacy, cognitive stress, and emotional well-being, remains underexplored. Self-efficacy, a core construct of educational and health psychology, is closely tied to learners’ motivation, performance, and mental resilience. Moreover, the increasing reliance on digital and AI-assisted tools in education calls for deeper understanding of their influence on learners’ perceived stress, anxiety levels, and overall psychological health.

**Subjects and Methods:** This study aimed to investigate how ALS affects learners’ self-efficacy and psychological responses during language learning tasks. Specifically, we examined whether ALS could reduce cognitive load and performance-related anxiety, thereby enhancing learners’ confidence and emotional engagement. An experimental design was adopted, involving 300 participants randomly assigned to two groups: an AI-assisted language learning group and a traditional instruction group. Participants engaged in equivalent language tasks. Measures included the General Self-Efficacy Scale (GSES), a Cognitive Load Index, and self-reported indicators of anxiety, learning engagement, and learning satisfaction. Pre-and post-tests were conducted, and data were analyzed using ANCOVA, structural equation modeling (SEM), and mediation analysis.

**Results:** Learners in the ALS group exhibited significantly higher post-intervention self-efficacy scores compared to the traditional group (β=0.58, p<0.001). Importantly, cognitive load mediated the relationship between ALS and self-efficacy (β=0.45, p<0.001), indicating that adaptive systems help alleviate mental fatigue and promote psychological confidence. Furthermore, learning engagement showed a strong positive correlation with self-efficacy improvements (β=0.62, p<0.001), and learning satisfaction was also higher in the ALS group (r=0.49, p<0.001). Participants reported lower task-related anxiety and greater perceived control over the learning process in the ALS condition.

**Conclusions:** This study provides empirical evidence that ALS not only improves self-efficacy but also contributes to learners’ mental well-being by reducing cognitive strain and anxiety. The findings underscore the psychological benefits of adaptive learning technologies and suggest that well-designed ALS platforms can act as both educational and psychological support systems. Future studies should investigate long-term psychological effects and examine individual variability in response to ALS interventions, particularly among high-stress or high-anxiety learner populations.

## PHHS25218: ANALYSIS OF MENTAL HEALTH FACTORS FOR DOPING IN COMPETITIVE SPORTS

Jian Li^a^, Hui Wang^b^, Chuan He^c^, Puzhu Han^d^, Na Ding^e^, Junjie Yan^e^, Xin Li^e^

^*a*^ *Sport Institute, Liaoning Institute of Science and Engineering, Jinzhou 121000, China,* ^*b*^ *First Center Hospital of Tianjin, Tianjin 300380, China,* ^*c*^ *Health Sciences of Kobe University Graduate School, Kobe 650-0034, Japan,* ^*d*^ *Arts and Science Colledg, Hongkong City University, Hongkong 999077, China,* ^*e*^ *Basic Teaching Department, Luxun Academy of Fine Arts, Shenyang 110000, China.*

**Background:** In today’s highly competitive world of sport, doping is a cancerous tumor that attracts a lot of attention. Athletes face unimaginable pressure to compete in sports events that hold countless honors and dreams. They train hard and sweat every day to win laurels and gain honors on the field of play. However, the cruelty of the competitive environment makes victory precious, and the fame, reputation, and high attention from all sectors of society attached to championships make some athletes lose their way in the face of temptation. At the same time, the psychology of social comparison is prevalent in the sports world. Athletes are constantly comparing themselves to their peers, and every game they win or lose is like a silent competition to measure their status in the sports world. Furthermore, the strong motivation to achieve drives them to aspire to constant breakthroughs and the pursuit of excellence. This extreme desire for success is intertwined with the reality of pressure, making the option of doping, which is contrary to the ethics of sport, seem to have some kind of “attraction”. Therefore, an in-depth analysis of these hidden psychological factors is undoubtedly crucial to maintaining the purity and fairness of sports events.

**Subjects and Methods:** To study doping’s psychological aspects, a multi-pronged approach was used. Surveys were administered to athletes across diverse sports, asking about their competition motives, pressure perception, and views on doping. In-depth interviews were held with those involved or resistant to doping. Theories like social comparison and achievement motivation were analyzed to understand their role in athletes’ doping decisions.

**Results:** Athletes’ pursuit of victory and honor, along with high pressure and self-cognition deviation, are key doping motivators. Social comparison and achievement motivation theories show how they can lead to doping. Doping also has severe long-term psychological impacts on athletes, including anxiety, guilt, and self-identity crises, as they face the consequences of their unethical actions.

**Conclusions:** To combat doping, targeted strategies are essential. Psychological counseling can assist athletes in handling pressure and building proper self-awareness. Educational programs should emphasize doping’s consequences. Coaches and organizations must create a healthy competitive atmosphere. These efforts will safeguard sports’ fairness and athletes’ well-being, upholding the true spirit of competition.

## PHHS25219: COGNITIVE MECHANISMS AND MENTAL HEALTH PROMOTION IN STUDENTS WITH LEARNING DIFFICULTIES: A “COMPREHENSION-APPLICATION-CREATION” LEARNING PROGRESSION MODEL

Yongjin Tan^a^, Wenyi Xu^b^, Xiaoli Yi^b^, Zhifeng Tan^c^

^*a*^ *Guangxi Lantian Aviation Vocational College, Laibin, Guangxi, 546100, China,* ^*b*^ *Huaihua Normal College, Huaihua, Hunan, 418000, China,* ^*c*^ *Xiangsihu College of Guangxi Minzu University, Nanning, Guangxi 530225, China.*

**Background:** Students with learning difficulties (SLD) represent a neurocognitively distinct population facing interrelated challenges of academic underachievement and mental health vulnerabilities. National epidemiological data indicate that 63% of SLD adolescents exhibit clinically significant test anxiety (STAI-Y2≥55), with 41% demonstrating academic self-efficacy below clinical thresholds (Ministry of Education, 2023). Neurobiological correlates reveal a 27% reduction in dorsal striatum activation during learning tasks compared to neurotypical peers (p<0.01), suggesting dysregulated reward-motivation pathways. Grounded in Bronfenbrenner’s bioecological model and cognitive load theory, this study introduces a tripartite “Comprehension→Application→ Creation” (CAC) intervention framework designed to integrate domain-specific cognitive training with psychosocial scaffolding. The intervention addresses critical gaps in SLD support by simultaneously targeting neurocognitive deficits and affective barriers to learning.

**Subjects and Methods:** A quasi-experimental design was employed with 53 SLD students (Mean age=13.2±1.5 years; 58% male). Cognitive profiles were assessed using the WISC-IV (Verbal Comprehension Index [VCI]: Mean=82.3±11.5; Perceptual Reasoning Index [PRI]: Mean=85.7±12.1). Mental health metrics included the SCL-90 anxiety subscale (Mean=1.8±0.6) and Academic Self-Efficacy Scale (Mean=2.9±0.8). Learning progression was operationalized through three stages: (1) Comprehension: Knowledge retention test (Cronbach’s α=0.89); (2) Application: Problem-solving tasks (intra-rater reliability: ICC=0.78); (3) Creation: Creative performance rubric (inter-rater agreement: κ=0.82). Multilevel regression analysis (MLR) examined cognitive effects on progression stages, controlling for baseline academic performance and anxiety.

**Results:** Cognitive-Mental Health Link: Global cognitive ability positively predicted self-efficacy (β=0.32, p=0.004) and negatively predicted anxiety (β=-0.28, p=0.012). VCI demonstrated the strongest association with anxiety reduction (β=-0.37, p=0.001). Learning Progression Effects: Cognitive ability explained 28% of variance in comprehension (β=0.42, p<0.01), 22% in application (β=0.38, p<0.05), and 19% in creation (β=0.31, p<0.05). PRI showed the largest effect on creative performance (β=0.39, p<0.01; 95% CI [0.17, 0.61]).Mediation Analysis: Academic self-efficacy fully mediated the relationship between cognitive ability and application performance (indirect effect=0.18, p=0.009). Anxiety partially mediated the cognitive ability–creative performance link (indirect effect=-0.11, p=0.035).

**Conclusions:** This neuroeducational investigation supports the efficacy of integrated cognitive-affective interventions for SLD populations, revealing distinct mechanistic pathways: verbal comprehension reduces anxiety through semantic restructuring, while perceptual reasoning enhances creative synthesis via visuospatial schema development. The full mediation by self-efficacy underscores the importance of incorporating metacognitive reflection and mastery experiences into academic interventions. Neurophysiological evidence of restored striatal activation suggests fMRI-guided neurofeedback protocols to enhance reward processing during learning. These findings propose a transdiagnostic intervention model integrating educational neuroscience and developmental psychopathology, prioritizing simultaneous optimization of cognitive scaffolding, affective regulation, and neuroplastic adaptation.

## PHHS25220: PSYCHOLOGICAL PERSPECTIVES ON TECHNOLOGICAL INNOVATION IN PHARMACEUTICAL ENTERPRISES: A BIBLIOMETRIC REVIEW AND PROSPECTS

Sukun Liu^a,b,c^, Congjun Chu^a^, Bin Yu^a^, Ling Yan^d^, Yu Zhang^a^

^*a*^ *Dalian Ocean University, Dalian 116025, China,* ^*b*^ *Dalian University of Technology, Dalian, 116023, China,* ^*c*^ *Dalian Bank Postdoctoral Workstation, Dalian 116001, China,* ^*d*^ *Dalian Minzu University, Dalian Liaoning, 116650, China.*

**Objective:** Technological innovation is a crucial factor for the survival and development of pharmaceutical enterprises. Individual creativity, team collaboration, and organizational incentive mechanisms are key drivers of technological innovation. The study aims to uncover the underlying psychological mechanisms that drive innovation by integrating insights from psychology, management, and sociology. Through a literature review, this research seeks to provide a comprehensive reference for academic research and practical applications while promoting interdisciplinary collaboration to address complex challenges in technological innovation.

**Subjects and Methods:** Data were sourced from the Web of Science database for 2014–2024, with research literature related to technological innovation in pharmaceutical enterprises selected as analysis objects. The study employed bibliometric analysis methods combined with visualization techniques to systematically comb through the relevant literature. The focus was on research themes such as keyword co-occurrence analysis and innovation and R&D strategies. At the same time, the analysis also explored the intersection of technological innovation with psychological well-being, ethical considerations, and sustainable development.

**Results:** The annual number of publications on technological innovation in pharmaceutical enterprises showed an increasing year-on-year trend over the past decade, with key scholars and research institutions mainly concentrated in Western countries. Research themes covered seven aspects, including innovation drivers, generic drug development, policy and regulatory impacts, and market competition, with R&D strategies and technological innovation management being the key research areas, with a notable emphasis on psychological factors such as creativity, collaboration, and leadership. Existing research has rarely explored innovation differences among enterprises of different sizes from a psychological perspective or the intersection of technological innovation with social ethics, leaving room for further study.

**Conclusions:** This study underscores the significance of interdisciplinary research integrating psychology, management, sociology, and related fields to delve into the underlying mechanisms of technological innovation. It advocates for incorporating sustainable development principles into technological innovation research. This study examines how these factors affect the outcomes of technological innovation in pharmaceutical companies through the psychological dimension. Interdisciplinary collaboration within the globalization paradigm is pivotal for advancing technological innovation research within the pharmaceutical enterprise sector. The study’s findings offer robust theoretical support and practical guidance for future academic research and practical applications in this field, particularly in fostering psychologically informed innovation strategies and policies.

**Acknowledgments**

This work was supported by the Liaoning Provincial Social Science Fund General Project (No. L19BJY043).

## PHHS25221: A STUDY ON THE RELATIONSHIP BETWEEN MEDICAL EXPENSES AND HOUSEHOLD DEBT UNDER MULTIPLE INFLUENCING FACTORS AND THE REFORM OF MEDICAL ASSISTANCE POLICIES

Liujie Tang^a^, Shaoping Kang^a^

^*a*^ *Kunming Medical University, Kunming City 650500, China.*

**Background:** Exploring the extent of medical expenses is crucial in understanding whether a family falls into debt or becomes over-indebted, potentially leading to poverty caused by illness, or even returning to poverty due to medical expenses. As China pursues its comprehensive victory in the fight against poverty, addressing health issues and the burden of medical expenses is an essential part of consolidating and expanding these achievements. This paper focuses on the important issue of “expensive medical treatment,” aiming to analyze how high medical expenses contribute to family indebtedness and poverty.

**Subjects and Methods:** Data were collected through the distribution of questionnaires, resulting in 4,101 valid responses that were analyzed using descriptive and statistical methods. Initially, descriptive analyses were employed to examine the relationship between various factors and households owning liabilities, including liabilities for medical reasons. The analysis also covered the range of annual household medical expenditures, instances of over-indebtedness, and the proportion of households borrowing specifically for medical purposes. Subsequently, inferential statistical techniques—such as univariate analysis and logistic regression—were utilized to investigate whether differences existed between past indebtedness and other pertinent factors.

**Results:** Results show that gender, age, household type, income, medical expenses, and family health significantly influence debt risk. Women, older adults, rural residents, and those with poorer health are more likely to incur arrears and debts, with risks increasing as health declines and age advances. Lower household income is strongly linked to higher debt likelihood, highlighting economic status as a protective factor. Low-income families face greater financial pressures, making them more vulnerable to debt issues. Poor health further worsens financial burden, increasing arrears behavior. To address these challenges, targeted policies should strengthen financial risk management and health protections for vulnerable groups, enhancing their resilience and promoting social stability and health development.

**Conclusions:** The burden of medical expenses on the population remains substantial. Therefore, the state should enhance the level of medical care, promote the concept of greater hygiene and improved health awareness, and implement a variety of measures to reduce the financial strain of medical expenses on families. These efforts will help to advance both socio-economic development and the health system.

**Acknowledgements**

This work was supported by a project grant from Philosophy and Social Sciences of Yunnan Province: Research on the Generation Mechanism and Prevention Path of Medical Financial Protection on Excessive Debt and Poverty Risk of micro-subjects (Grant No. YB2023016). The 2023 Undergraduate Education and Teaching Reform Research Project of Yunnan Province “Research and Practice on the Construction of a Simulation Teaching Platform for New Liberal Arts Mathematics and Intelligence under the Background of Medical and Cultural Integration” (Grant No. JG2023019), Medical Psychology and Health Management Team, Kunming Medical University (Grant No. 2024XKTDPY18).

## PHHS25222: THE IMPACT OF CIGAR CONSUMERS’ HEALTH LITERACY ON CIGAR ADDICTION BEHAVIOR: A CROSS-SECTIONAL STUDY

Dan Xiao^a^, Rui Sun^b^, Kang Wang^a^, Xingdong Wang^a^, Degang Zhang^b^, Mei Nian^b^, Bingsheng Yan^c^

^*a*^ *College of Economics and Management, Jiangxi Agricultural University, Nanchang, Jiangxi 330045, China,* ^*b*^ *Lijiang City Company of Yunnan Tobacco Company, Lijiang, Yunnan, 674100, China,* ^*c*^ *China National Tobacco Corporation Staff Further Training College, Zhengzhou, Henan, 450008, China.*

**Objective:** This study aimed to comprehensively examine cigar addiction patterns among adult consumers in Yunnan Province, China, by investigating the prevalence and severity of dependence, analyzing the association between health literacy levels and addictive behaviors with a focus on educational disparities, and identifying modifiable risk factors to inform targeted public health interventions for prevention and cessation strategies.

**Subjects and Methods:** We conducted a cross-sectional study of 631 adult cigar consumers recruited through convenience sampling across Yunnan Province. Health literacy was assessed using the validated 16-item European Health Literacy Questionnaire (HLS-EU-Q16), which evaluates four key dimensions: understanding, accessing, appraising, and applying health information. Nicotine dependence was measured via the Fagerström Test for Nicotine Dependence (FTND), with scores categorized into mild, moderate, and severe addiction levels. Participants also completed detailed demographic questionnaires capturing education level, income, smoking history, and health status. Data analysis employed chi-square tests and probit regression models to examine associations while controlling for potential confounders.

**Results:** There was an alarmingly high prevalence of cigar addiction (67.2%) among participants, this addiction severity showed a significant negative association with health literacy levels (β = -0.38, p < 0.001), demonstrating that individuals with lower health literacy were particularly vulnerable to developing stronger dependence. The protective effect of health literacy followed a clear educational gradient, being most pronounced among bachelor’s degree holders who exhibited the strongest inverse association (β = -0.63). The demographic profile of cigar consumers revealed a predominantly male (91.1%), middle-aged, high-income population that concurrently bore elevated health risks, including substantial rates of overweight/obesity (48.5%) and acute health symptoms (23.6%). Subgroup analyses uncovered a dose-response relationship where health literacy’s protective effect against addiction intensified with education level, with explanatory power progressively increasing from 0.08 (least educated subgroup) to 0.23 (most educated subgroup).

**Conclusions:** This study provides compelling evidence that health literacy serves as a modifiable protective factor against cigar addiction, with effects that vary substantially by education level. The findings highlight the urgent need for targeted, literacy-appropriate interventions in tobacco control strategies, particularly for less educated populations who demonstrate both lower health literacy and higher addiction vulnerability. Public health initiatives should prioritize developing tiered health communication approaches that account for educational disparities and implementing community-based programs to improve health literacy specifically regarding cigar risks.

**Acknowledgments**

This work was supported by a project grant from Science and Technology Program Project of Lijiang City Company, Yunnan Tobacco Company—“Research on Cross-border Marketing and Terminal Standardization System Construction in the Cigar Consumption Market” (Grant No. 202253070024005).

## PHHS25223: EARLY CLINICAL EFFICACY OBSERVATION OF MINIMALLY INVASIVE ALL-FEMTOSECOND LASER PRECISION 4.0-VISULYZE IN PATIENTS WITH DIFFERENT DIOPTERS

Dan Liu^a^, Junhua Hao^a^, Peng Huo^a^, Hanhan Kong^a^, Dan Wang^a^, Lijuan Sun^a^

^*a*^ *Department of Laser Vision Correction, Handan Aiyan Ophthalmic Hospital, Handan 056000, Hebei, China.*

**ABSTRACT:**

**Background:** SMILE surgery has no risk of corneal flap displacement, reduces postoperative discomfort, lowers the incidence of dry eye syndrome, and has good safety and efficacy. With the increasing demand for visual quality and the development of science and technology, all femtosecond surgery is moving towards personalization, diversification, and intelligence to achieve the optimization of refractive correction surgery design and the highest quality correction effect, providing refractive error patients with safer, more accurate, and more natural visual correction effects. So the minimally invasive full femtosecond precision 4.0-VISULYZE emerged, which obtains more accurate Nomogram by quantitatively analyzing and predicting the required adjustment amount of preoperative infusion machine through graphs or tables, achieving more precise correction goals. Although minimally invasive full femtosecond precision 4.0-VISULYZE has been applied in the clinical correction of refractive errors, there are very few international and domestic reports on the early clinical efficacy evaluation of minimally invasive full femtosecond precision 4.0-VISULYZE for different degrees of myopia and astigmatism correction. To evaluate the early clinical outcomes of minimally invasive SMILE Precision 4.0-VISULYZE in correcting myopia across varying refractive ranges.

**Subjects and Methods:** A prospective non-randomized controlled study included 73 patients (112 eyes) undergoing SMILE Precision 4.0-VISULYZE at Handan Aiyan Ophthalmic Hospital (August 2024–February 2025). Patients were categorized into three groups based on spherical equivalent (SE): low myopia (≤−3.00 D, 27 cases/42 eyes), moderate myopia (−3.00 D to −6.00 D, 35 cases/53 eyes), and high myopia (>−6.00 D, 11 cases/17 eyes). Postoperative follow-ups at 1 day, 1 week, and 1 month evaluated safety index (SI), efficacy index (EI), and refractive stability using uncorrected visual acuity (UCVA), SE, best-corrected visual acuity (BCVA), and complications.

**Results:** At 1 month postoperatively, SI values were 1.16±0.10 (low), 1.15±0.13 (moderate), and 1.12±0.10 (high). No significant EI differences were observed among groups at 1 day or 1 week (P>0.05). At 1 month, EI values were 1.06±0.09 (low), 1.09±0.14 (moderate), and 1.00±0.07 (high), with a significant difference between moderate and high myopia groups (Z=−2.523, P=0.012<0.017). Refractive stability analysis showed SE residuals of 0.20±0.64 D (low), 0.07±0.37 D (moderate), and −0.27±0.46 D (high) at 1 month. Intraoperative complications included opaque bubble layers (OBL, 2 cases) and suction loss (2 cases, resolved intraoperatively). One case of diffuse lamellar keratitis (DLK) recovered with treatment.

**Conclusions:** Minimally invasive SMILE Precision 4.0-VISULYZE demonstrates favorable safety, efficacy, and early stability for low-to-high myopia correction. However, reduced EI and refractive stability in the high myopia group necessitate extended follow-up to validate long-term outcomes.

## PHHS25224: A STUDY OF THE EFFECT OF SPIRITUAL LEADERSHIP ON EMPLOYEES’ KNOWLEDGE HIDING BEHAVIOR: MEDIATING EFFECT BASED ON PSYCHOLOGICAL SECURITY AND MODERATING UTILITY OF EMPLOYEE TRUST

Jiancheng Feng^a^, Hongyu Ren^b^

^*a*^ *Peoples’ Friendship University of Russia, Moscow 119285, Russia,* ^*b*^ *Huaxia Bank Liaocheng Branch, Liaocheng 252000, China.*

**Background:** In the contemporary knowledge economy, the widespread prevalence of employee knowledge-hiding behaviors significantly hampers organizational innovation and profitability. Traditional leadership approaches have often failed to effectively address this issue. Spiritual leadership, which emphasizes meeting employees’ spiritual needs and promoting their self-actualization, offers a potential solution by fostering a more meaningful and fulfilling work environment. This study investigates the impact of spiritual leadership on employee knowledge-hiding behaviors, with a particular focus on the mediating role of psychological security and the moderating effect of employee trust.

**Subjects and Methods:** The research was conducted using an online questionnaire distributed to employees of local Chinese enterprises, yielding 320 valid responses out of 340 distributed questionnaires. The data were analyzed using SPSS22.0 and STATA17.0 software, employing reliability analysis, descriptive statistics, correlation analysis, and regression analysis to examine the relationships among the variables.

**Results:** The results indicate that spiritual leadership has a significant negative impact on employee knowledge-hiding behaviors (β = -1.078, p< 0.001), suggesting that it can effectively reduce such behaviors. This finding underscores the importance of addressing employees’ spiritual needs in leadership practices. Psychological security was found to partially mediate the relationship between spiritual leadership and knowledge-hiding behaviors (β= -0.213, p< 0.001), indicating that spiritual leadership indirectly reduces knowledge hiding by enhancing employees’ psychological security. Furthermore, employee trust was found to positively moderate the negative effect of psychological security on knowledge-hiding behaviors (β= 0.26, p< 0.001). This implies that higher levels of trust amplify the inhibitory effect of psychological security on knowledge hiding, highlighting the critical role of trust in reinforcing the positive impact of spiritual leadership.

**Conclusions:** The study concludes that spiritual leadership can effectively reduce employee knowledge-hiding behaviors by fulfilling their spiritual needs and increasing their psychological security. Employee trust further strengthens the inhibitory effect of psychological security on knowledge-hiding behaviors. These findings provide valuable insights for organizational leaders, suggesting that fostering a supportive and trusting work environment can significantly enhance knowledge sharing and organizational performance. Future research may explore additional mediating or moderating factors in different cultural contexts to further validate and expand the applicability of these findings.

## PHHS25225: PSYCHOLOGICAL COPING MECHANISMS FOR AUDIENCE-GAZE PRESSURE IN VOCAL PERFORMANCE

Zhuo Jiang ^a^

^*a*^ *Lanzhou University of Arts and Science, Lanzhou, Gansu, China.*

**Background:** Against the backdrop of the rapid development of contemporary culture and art, vocal performance-an art form rich in emotional impact-plays an important role in enabling emotional communication between performers and audiences. However, onstage audience gaze is often a major source of psychological pressure for performers. In actual performances, many performers, unable to withstand this pressure, experience physiological reactions such as accelerated heartbeat and voice tremor, which in turn induce negative emotions such as tension and anxiety, and may even lead to mistakes such as forgetting lyrics, singing off-pitch, or stiff movements, all of which seriously impair the artistic appeal and integrity of the performance. As vocal art becomes increasingly important in various cultural activities, studying the psychological coping mechanisms for audience-gaze pressure in vocal performance not only enriches the theoretical system of performance psychology but also has significant theoretical and practical value for improving performers’ professional quality and stage expressiveness.

**Subjects and Methods:** Focusing on the psychological coping mechanisms for audience-gaze pressure in vocal performance, this study adopts a comprehensive approach combining literature review, case analysis, and theoretical deduction. By surveying relevant literature to grasp the current research status, dissecting typical cases to explore the causes and manifestations of pressure, and integrating psychological and art-theory perspectives, the study systematically investigates effective coping strategies.

**Results:** Audience-gaze pressure arises from an interplay of multiple factors. At the level of self-cognition, some performers “underestimate their ability,” intensifying self-doubt. From the dimension of social evaluation, the audience’s need for emotional resonance, colleagues’ technical-norm expectations, and public opinion’s value expectations for dissemination constitute multidimensional sources of pressure. Stage factors such as spotlights and enclosed space heighten the sense of “being scrutinized,” while aesthetic divergences stemming from audience age and cultural background further aggravate pressure. These pressures manifest physiologically as disordered breathing and muscle stiffness, emotionally as anxiety and fear, and behaviorally as rigid movements and singing errors, all of which seriously disrupt performance quality.

**Conclusions:** Accordingly, the study proposes three major coping mechanisms-cognitive adjustment, emotional regulation, and behavioral training. By properly understanding audience gaze and making reasonable judgments about one’s abilities, cognitive-level pressure can be alleviated; through relaxation training and positive self-suggestion, emotions can be regulated; through accumulating performance experience and optimizing stage behavior, psychological endurance and performance level can be enhanced, providing performers with concrete, actionable guidance.

**Acknowledgments**

This work was supported by the Doctoral Scientific Research Start-up Fund Project of Lanzhou University of Arts and Science, Project Number 2023QDJ09, titled “Innovative Practice Research on Music Teaching Models Based on Multicultural Backgrounds”.

## PHHS25226: STUDY ON THE MECHANISM AND CLINICAL EFFICACY OF FLOATING NEEDLE THERAPY FOR LUMBAR DISC HERNIATION WITH RADICULOPATHY BASED ON SURFACE ELECTROMYOGRAPHY

Long Jia^a^, Ming Cheng^a^, Zhiyuan Xue^a^, Xiaoli Wu^a^, Li Deng^a^

^*a*^ *Chengdu Jinniu District People’s Hospital, Chengdu 610036, Sichuan, China.*

**Background:** To analyze the clinical efficacy and mechanism of floating needle therapy for patients with lumbar disc herniation and radiculopathy through surface electromyography (sEMG) of the erector spinae muscle.

**Subjects and Methods:** Thirty patients with lumbar disc herniation and radiculopathy who met the inclusion criteria were selected. They were routinely treated with oral analgesics and neurotrophic agents, and dehydration agents and corticosteroids for anti-inflammation were administered as per the patient’s condition. Floating needle therapy was added on top of this conventional treatment. SEMG signals of the lumbar erector spinae muscle (AEMG and MF values) were analyzed before and after treatment. The Japanese Orthopaedic Association Score (JOA) and Visual Analogue Scale (VAS) were used to assess discomfort and pain levels. Anxiety levels were assessed using the Self-assessment Scale for Anxiety (SAS). Thirty healthy subjects were also included for sEMG data collection.

**Results:** All subjects underwent sEMG testing. After floating needle therapy, the JOA score increased to 23.90, while the VAS score decreased to 2.33 (pre-treatment JOA = 9.77, VAS = 6.57), with statistically significant differences (P < 0.05). After treatment, the AEMG of the lumbar erector spinae muscle was 71.60 ± 8.73 and the MF value was 60.30 (pre-treatment AEMG = 36.87 ± 5.17, MF = 51.7 ± 5.26). The time-domain indicator (AEMG) significantly increased after treatment (P < 0.05), while the frequency-domain indicator (MF) decreased. The healthy volunteers’ AEMG was 85.5 ± 3.73 and MF was 51.7 ± 5.26. The post-treatment SAS score was 34.10 ± 2.72 (pre-treatment SAS = 49.20 ± 3.62), with a statistically significant difference (P < 0.05).

**Conclusions:** After floating needle therapy, patients showed improved contraction strength of the lumbar erector spinae muscle, activation of muscle function, and varying degrees of improvement in muscle activity and fatigue levels. Clinical symptoms were significantly alleviated, and psychological conditions also showed marked improvement, with no significant complications or adverse reactions.

**Acknowledgements**

This work was financially supported by the 2025 First Batch of Key R&D Projects in Chengdu of Chengdu Science and Technology Bureau (2024-YF05-02589-SN); the Chengdu Medical Research Project (2023589); the Jinniu District Health and Wellness Bureau 2024 District-Level Medical Research Project (JNKY2024-33); and the Jinniu District Medical Association Research Project (JNKY2023-07).

## PHHS25227: RESEARCH ON THE DEVELOPMENT OF CHILDREN’S MENTAL HEALTH LEVEL BASED ON MOBILE LEARNING INTERACTION ACTIVITIES BETWEEN TEACHERS AND CHILDREN

Jiachao Wei^a^, Weiwei Yu^b^, Jianling Hu^a^, Yaning Chen^a^

^*a*^ *School of Educational Science, Nanning Normal University, Nanning 530299, China,* ^*b*^ *Nanning Wuxiang New District the Second Experimental Kindergarten, Nanning 530000, China.*

**Background:** With the rapid development of information technology, the informatization of preschool education has developed rapidly, which puts forward new requirements for teachers to use information technology to carry out teaching and education. Mobile learning is an important part of the development of educational informatization, and teacher-child interaction is an important indicator of the high-quality development of preschool education. With the rapid development of society, the mental health problems of young children are also widely concerned by all sectors. Therefore, it is an urgent issue for preschool teachers to improve the mental health level of young children through mobile learning in teacher-child interaction activities.

**Subjects and Methods:** Based on the framework of mobile learning theory of Kur of Athabasca University in Canada, guided by the “Guidelines for the Assessment of the Quality of Early Childhood Education and Care” and the “Outline for the Guidance of Early Childhood Education”, with the content of improving the mobile learning ability of Chinese early childhood teachers in the interaction between teachers and children to enhance children’s mental health, a questionnaire and interview outline was compiled, and a questionnaire and interview were conducted with early childhood teachers in Nanning, Guangxi, China, to conduct a current situation survey.

**Results:** After analyzing the questionnaire data and interview content, the study found that there is a lack of mobile learning willingness among teachers, insufficient mobile learning resources in kindergartens, and weak social interaction among teachers in mobile learning, which directly affects the mental health of young children.

**Conclusions:** Therefore, taking the mobile learning theory of Kur of Athabasca University in Canada as the framework, taking teachers, equipment, and social interaction as dimensions for analysis, and improving the mobile learning willingness of young children’s teachers, optimizing the mobile equipment of young children’s teachers, and improving the social interaction among teachers to enhance the mobile learning ability of young children’s teachers, teachers can enhance their professional development through mobile learning, thereby improving the level of interaction between teachers and children to promote the healthy psychological development of children, It has promoted the high-quality development of preschool education.

## PHHS25228: EMOTIONAL REGULATION ABILITY AND MENTAL HEALTH DEVELOPMENT OF INNOVATIVE AND ENTREPRENEURIAL TALENTS FROM THE PERSPECTIVE OF POSITIVE PSYCHOLOGY

Yani Xie ^a^

^*a*^ *Kunming University of Science and Technology, Kunming, Yunnan, China.*

**Background:** In the context of increasingly fierce global economic competition, innovation and entrepreneurship have become an important force driving social and economic development. However, innovation and entrepreneurial activities are often accompanied by high uncertainty and pressure, which poses a challenge to the mental health of individuals. Positive psychology emphasizes that emotion regulation not only helps to cope with negative emotions, but also promotes the generation of positive emotions, thereby improving mental health, creativity and work performance. However, there is a lack of systematic research on the relationship between emotion regulation ability and the mental health development of innovative and entrepreneurial talents. This paper aims to explore how emotion regulation ability affects the mental health and work performance of innovative and entrepreneurial talents.

**Subjects and Methods:** This study selected 300 innovative and entrepreneurial talents from different industries, including entrepreneurs, founders of start-up companies, and technology R&D personnel. A mixed research method was used, combining quantitative questionnaire surveys and qualitative interviews, to gain a deeper understanding of the application and effects of emotion regulation strategies. The data were analyzed using SPSS, and correlation analysis and regression analysis were used to explore the relationship between emotion regulation ability and mental health and work performance.

**Results:** The findings reveal a significant positive correlation between emotional regulation ability and mental health (r = 0.45, p < 0.01), indicating that individuals with stronger emotional regulation skills tend to exhibit better psychological well-being, with lower levels of anxiety, depression, and stress. Furthermore, the study demonstrates that emotional regulation ability is a predictor of both creativity and entrepreneurial performance. Entrepreneurs and innovators with higher emotional regulation scores reported greater levels of innovation, problem-solving capabilities, and work performance. Notably, individuals who adopted positive emotional regulation strategies, such as reappraisal, reported enhanced mental resilience and better adaptation to entrepreneurial stressors. Path analysis indicated that emotional regulation indirectly influences entrepreneurial outcomes by improving mental health, highlighting the mediating role of mental health in this relationship.

**Conclusions:** This study shows that emotion regulation ability plays an important role in the mental health and work performance of innovative and entrepreneurial talents. Good emotion regulation ability can not only reduce the impact of negative emotions, but also promote the improvement of positive emotions and psychological resilience, thereby improving innovation ability and entrepreneurial performance. The research results provide a theoretical basis for how to improve the mental health of innovative and entrepreneurial talents through emotion regulation intervention, and provide guidance for practical applications in related fields. Future research can further explore the causal relationship between emotion regulation and entrepreneurial outcomes, and extend it to cross-industry and cross-cultural comparative studies.

## PHHS25229: THE PSYCHOLOGICAL TRIGGERS OF CONSUMER BEHAVIOR: SOCIAL MEDIA ADDICTION, SOCIAL COMPARISON AND ANXIETY

Jiayi Du ^a^

^*a*^ *School of Event and Communication, Shanghai University of International Business and Economics, Shanghai 201620, China.*

**Background:** This study aims to explore the intricate relationships between social media addiction, social comparison, and consumer anxiety, investigating how these psychological mechanisms influence modern consumption behaviors. The research also examines how brands strategically leverage these psychological processes to enhance sales and maintain consumer engagement. With the increasing prevalence of digital platforms in everyday life, understanding the impact of social media on consumer psychology is essential for both academic research and marketing strategies.

**Subjects and Methods:** This paper adopts a comprehensive literature review approach, analyzing empirical studies and theoretical frameworks related to social media addiction, social comparison, and consumer anxiety. This paper reviews the literature exploring associations between internet or social media use and social anxiety, highlighting various psychological factors involved. The discussion synthesizes findings from previous research, including studies on compulsive social media use, emotional triggers in digital environments, and their effects on consumer decision-making. Particular emphasis is placed on studies that examine impulsive buying behaviors influenced by social media marketing strategies, algorithm-driven advertising, and influencer endorsements.

**Results:** The literature extensively explores the relationships among internet use, social media engagement, anxiety, and various psychological concerns, illustrating a clear association between excessive digital engagement and mental health issues. The review identifies significant correlations between social media addiction and impulsive consumer behaviors. Studies indicate that excessive social media use fosters unrealistic lifestyle expectations, leading to increased consumer anxiety and compulsive spending. Social comparison, particularly upward comparisons with influencers and peers, exacerbates feelings of inadequacy, further driving the desire to engage in aspirational consumption. Additionally, marketing strategies that exploit psychological vulnerabilities—such as FOMO-driven promotions, influencer marketing, and hyper personalized advertisements reinforce impulsive buying tendencies and brand loyalty.

**Conclusions:** Continued exploration of these relationships is vital for clarifying underlying psychological mechanisms. The pervasive nature of social media platforms significantly intensifies social comparison by providing constant visibility into others’ lifestyles and consumption habits. The interplay between social media addiction, social comparison, and consumer anxiety significantly shapes purchasing decisions, often leading to impulsive and excessive consumption. As brands and digital platforms continue to refine their strategies, ethical concerns regarding consumer manipulation and psychological well-being need to be addressed.

## PHHS25230: CLINICAL OBSERVATION OF YU’S CRANIAL ACUPUNCTURE IN THE TREATMENT OF CERVICOGENIC VERTIGO

Yanzhi Liu^a^, Na Liu^a^, Jianwen Li^a^, Dianjie Wu^a^

^*a*^ *Sanshui District People’s Hospital of Foshan City, Foshan 528100, Guangdong, China.*

**Background:** Cervicogenic vertigo (CGV) is a common clinical syndrome characterized by dizziness caused by cervical spine diseases. Currently, Western medical approaches have limited efficacy and high recurrence rates. Acupuncture has emerged as a promising alternative therapy, but conventional electroacupuncture (EA) requires complex acupoint selection and prolonged neck immobilization. This study aimed to evaluate the clinical efficacy and hemodynamic effects of Yu’s cranial acupuncture therapy versus EA in the treatment of CGV.

**Subjects and Methods:** A total of 72 patients with cervicogenic vertigo who were admitted to our hospital from January 2024 to June 2024 were selected as the research subjects. After meeting the inclusion and exclusion criteria, they were divided into a control group and an observation group by simple randomization, with 36 cases in each group. The observation group was treated with Naohu (GV17) to the Hou Tian Bagua diagram, and the control group was treated with dense and sparse wave stimulation of acupoints GV20, GB20, LR3, PC6, and ST40 according to the standard scheme. The cervicogenic vertigo symptom and function scale (ESCV) score, the vertigo disorder inventory (DHI) score and the transcranial Doppler (TCD) evaluation of vertebral basilar artery blood flow velocity were compared between the two groups before and after treatment.

**Results:** After treatment and one month of follow-up, there was no significant difference in the cervical vertigo symptom and function rating scale, the dizziness disorder inventory and the efficacy evaluation score between the observation group and the control group (P>0.05). However, the observation group showed better effects in improving the blood flow velocity of the vertebral artery (P<0.05).

**Conclusions:** Yu’s cranial acupuncture has no significant difference in the efficacy of treating vertigo compared with traditional electroacupuncture, but Yu’s cranial acupuncture has advantages in reducing the complexity of treatment, and no adverse events were reported. These research results show that Yu’s cranial acupuncture is a safe and effective alternative therapy. It only requires one acupoint and accurate injection according to the pulse. It is simple and safe to operate and has a definite effect.

**Acknowledgments**

This work was supported by the Foshan City self-funded scientific and technological innovation project into the database (No.2320001006377) and the Foshan City 14th Five-Year Medical Training Special Project (No. FSPY145231).

## PHHS25231: A STUDY ON THE RELATIONSHIP BETWEEN ENVIRONMENTAL COLOR, RELAXATION RESPONSE, AND POSITIVE EMOTIONS: A CASE STUDY OF CLIENTS TO PSYCHOLOGICAL COUNSELING CENTER IN UNIVERSITY

Jing Tan^a^, Weijia Zhu^b^

^*a*^ *College of Arts and Design, Mianyang Normal University, Mianyang, China,* ^*b*^ *School of Design, Zhijiang College of Zhejiang University of Technology, Shaoxing, China.*

**Objectives:** This study investigated the relationship between environmental color, relaxation response and positive emotions in a sample of clients from the psychological counseling center in university.

**Methods:** In this paper, means and standard deviations are used to describe the results of the positive emotion experiment. Then Spearman correlation analysis and linear regression analysis are used to test the effect of relaxation on positive emotion, and the mediating effect of environmental color is examined.

**Results:** In the healing environment of the psychological counseling center in university, the relaxation response has a positive impact on positive emotions at the psychological level. The better the relaxation effect of the environmental color, the higher the positive emotions.

**Conclusions:** The results of this study illustrate the importance of environmental color in psychological counseling center in university. It suggests that experiencing relaxation through environmental colors can evoke positive emotions, which may contribute to psychological healing. It also indicates that the relationship between color and the characteristics of the interior space used should be considered in the construction of healing environments.

